# Supplementary material for: Heat flows enrich prebiotic building blocks and enhance their reactivity
Source: Nature. 2024 Apr 3;628(8006):110–6. doi: 10.1038/s41586-024-07193-7 (PMC10990939; doi:10.1038/s41586-024-07193-7)
Supplement: Supplementary file 1 — This file contains one Supplementary Discussion (stability of enrichment against fluctuations and for small temperature gradients), 15 Supplementary Figures (further heat maps and errors, further results in experimental network, details of simulation and modelling and information on detection), 66 Supplementary Tables (abbreviations, calibration and raw data) and exemplary calibration data. [file 41586_2024_7193_MOESM1_ESM.pdf]

nature portfolio

https://doi.org/10.1038/s41586-024-07193-7

Supplementary information

Heat flows enrich prebiotic building blocks and enhance their reactivity

In the format provided by the authors and unedited

Supplementary Discussions 5

Supplementary Discussion 1*: Stability of the thermogravitational accumulation against temporal fluctuations of the temperature gradient and behaviour in the limit of small temperature gradients.* 5

Supplementary Figures 7

Supplementary Figure 1*: Enrichment of nucleobases, nucleosides, 5'-nucleotides and 2',3'-cyclic nucleotides after 6 h.* 7

Supplementary Figure 2*: Error maps for Figure 3.* 8

Supplementary Figure 3*: Error maps for Extended Data Fig. 5.* 9

Supplementary Figure 4*: Separation of amino acids in experimental network* 10

Supplementary Figure 5*: Individual experiments and per-chamber depiction of network experiments* 11

Supplementary Figure 6*: Maximal pairwise separation for various species.* 13

Supplementary Figure 7*: Concentration profiles in heat flux cell and fit for Soret coefficient.* 14

Supplementary Figure 8*: Influence of diffusion constant on determination of Soret coefficient for different exemplary species.* 15

Supplementary Figure 9*: Errors in Soret coefficients for network models.* 16

Supplementary Figure 10*: Example distribution of flow rates for a network.* 17

Supplementary Figure 11*: Example of enrichment behavior in a single heat-flow chamber as used in the network simulation.* 18

Supplementary Figure 12*: Stability of thermogravitational accumulation against fluctuations of the applied temperature gradients.* 19

Supplementary Figure 13*: TMP hydrolysis under reaction conditions* 20

Supplementary Figure 14*: Separation of molecules in LC methods* 21

Supplementary Figure 15*: Reproducibility of amino acid detection in LC method described in Methods.* 22

Supplementary Tables 23

Supplementary Table 1*: List of abbreviations used.* 23

Supplementary Table 2*: Enrichment against mean concentration of set of molecules.* 25

Supplementary Table 3*: List of coefficients of determination, slopes and standard deviations of linear fits of LC and IC methods* 27

Supplementary Table 4*: List of coefficients of determination, slopes and standard deviations of linear fits of LC methods for amino acids.* 28

Supplementary Table 5*: Raw data Fig. 2a – 2-aminoazoles in water, 170 µm, 18 h, 18 K.* 30

Supplementary Table 6*: Raw data Fig. 2b and Extended Data Fig. 3 – AA in water, 170 µm, 18 h, pH 7.4.* 31

Supplementary Table 7*: Raw data Fig. 2b and Extended Data Fig. 3 – AA in water, 170 µm, 18 h, pH 7.4 – enrichments in top fraction* 35

Supplementary Table 8*: Raw data Fig. 2b and Extended Data Fig. 3 – AA in water, 170 µm, 18 h, pH 7.4 – enrichments in bottom fraction* 38

Supplementary Table 9*: Raw data Fig. 3a and Extended Data Fig. 5a – nucleobases in water, 170µm, 18h* 41

Supplementary Table 10*: Raw data Fig. 3b – adenosine and AMPs in water, 170µm, 18h* 42

Supplementary Table 11*: Raw data Fig. 3c – Ribonucleosides in water, 170µm, 18h* 43

Supplementary Table 12*: Raw data Fig. 3d – 5'-Ribonucleotides in water, 170µm, 18h* 44

Supplementary Table 13*: Raw data Fig. 3e – Deoxyribonucleotides in water, 170µm, 18h* 45

Supplementary Table 14*: Raw data Fig. 4b-c – Experimental network, raw data* 46

Supplementary Table 15*: Raw data Fig. 4b-c – Experimental network, concentrations (µM))* 49

Supplementary Table 16*: Raw data Fig. 4b-c – Experimental network, normalization against total concentration of species in chamber network (c_0) per experiment* 52

Supplementary Table 17*: Raw data Fig. 5 – Time series of Glycine dimerization* 55

Supplementary Table 18*: Raw data Fig. 5 – Variation of TMP concentration* 56

Supplementary Table 19*: Raw data Supplementary Figure 13 – TMP degradation* 57

Supplementary Table 20*: Raw data Fig. 5 – Glycine dimerization in heat flow chamber* 58

Supplementary Table 21*: Raw data Extended Data Fig. 2a – 2-aminoazoles in water, 170 µm, 18 h, 10 K.* 60

Supplementary Table 22*: Raw data Extended Data Fig. 2a – 2-aminoazoles in water, 170 µm, 18 h, 5 K.* 61

Supplementary Table 23*: Raw data Extended Data Fig. 2b and 2f – mixture of non-proteogenic and proteogenic AA in water, 170 µm, 18 h, 4 fractions* 62

Supplementary Table 24*: Raw data Extended Data Fig. 2f – mixture of non-proteogenic and proteogenic AA in water, 170 µm, 18 h – enrichments in top fraction, 4 fractions* 65

Supplementary Table 25*: Raw data Extended Data Fig. 2f – mixture of non-proteogenic and proteogenic AA in water, 170 µm, 18 h – enrichments in bottom fraction, 4 fractions* 67

Supplementary Table 26*: Raw data Extended Data Fig. 2b and 2g – mixture of non-proteogenic and proteogenic AA in water, 170 µm, 18 h, 12 fractions* 69

Supplementary Table 27*: Raw data Extended Data Fig. 2g – mixture of non-proteogenic and proteogenic AA in water, 170 µm, 18 h – enrichments in top fraction, 12 fractions* 73

Supplementary Table 28*: Raw data Extended Data Fig. 2g – mixture of non-proteogenic and proteogenic AA in water, 170 µm, 18 h – enrichments in bottom fraction, 12 fractions* 75

Supplementary Table 29*: Raw data Extended Data Fig. 3 – AA in water, 170 µm, 18 h, pH 5.1.* .. 77

Supplementary Table 30*: Raw data Extended Data Fig. 3 – AA in water, 170 µm, 18 h, pH 5.1 – enrichments in top fraction* 80

Supplementary Table 31*: Raw data Extended Data Fig. 3 – AA in water, 170 µm, 18 h, pH 5.1 – enrichments in bottom fraction* 82

Supplementary Table 32*: Raw data Extended Data Fig. 3 – AA in water, 170 µm, 18 h, pH 9.9* ... 84

Supplementary Table 33*: Raw data Extended Data Fig. 3 – AA in water, 170 µm, 18 h, pH 9.9 – enrichments in top fraction* 87

Supplementary Table 34*: Raw data Extended Data Fig. 3 – AA in water, 170 µm, 18 h, pH 9.9 – enrichments in bottom fraction* 89

***Supplementary Table 35**: Raw data Extended Data Fig. 4 – AA in water, 170 µm, 18 h, 1 mM NaCl, pH 7.4* 91

***Supplementary Table 36**: Raw data Extended Data Fig. 4 – AA in water, 170 µm, 18 h, 1 mM NaCl, pH 7.4 – enrichments in top fraction* 93

***Supplementary Table 37**: Raw data Extended Data Fig. 4 – AA in water, 170 µm, 18 h, 1 mM NaCl, pH 7.4 – enrichments in bottom fraction* 95

***Supplementary Table 38**: Raw data Extended Data Fig. 4 – AA in water, 170 µm, 18 h, 10 mM NaCl, pH 7.4* 97

***Supplementary Table 39**: Raw data Extended Data Fig. 4 – AA in water, 170 µm, 18 h, 10 mM NaCl, pH 7.4 – enrichments in top fraction* 99

***Supplementary Table 40**: Raw data Extended Data Fig. 4 – AA in water, 170 µm, 18 h, 10 mM NaCl, pH 7.4 – enrichments in bottom fraction* 101

***Supplementary Table 41**: Raw data Extended Data Fig. 4 – AA in water, 170 µm, 18 h, 100 mM NaCl, pH 7.4* 103

***Supplementary Table 42**: Raw data Extended Data Fig. 4 – AA in water, 170 µm, 18 h, 100 mM NaCl, pH 7.4 – enrichments in top fraction* 105

***Supplementary Table 43**: Raw data Extended Data Fig. 4 – AA in water, 170 µm, 18 h, 100 mM NaCl, pH 7.4 – enrichments in bottom fraction* 107

***Supplementary Table 44**: Raw data Extended Data Fig. 5b – nucleobases in 10 % formamide, 170µm, 18h* 109

***Supplementary Table 45**: Raw data Extended Data Fig. 5c – nucleobases in 100 mM phosphate buffer, 170µm, 18h* 110

***Supplementary Table 46**: Raw data Extended Data Fig. 5d – nucleobases in 10 % methanol, 170µm, 18h* 111

***Supplementary Table 47**: Raw data Extended Data Fig. 5e – nucleobases in water, 127 µm, 18h* 113

***Supplementary Table 48**: Raw data Extended Data Fig. 5f – nucleobases in water, 150 µm, 18h* 114

***Supplementary Table 49**: Raw data Extended Data Fig. 5g – nucleobases in water, 200 µm, 18h* 115

***Supplementary Table 50**: Raw data Extended Data Fig. 5h – nucleobases in water, 170 µm, 18h, pH 3* 116

***Supplementary Table 51**: Raw data Extended Data Fig. 5i – nucleobases in water, 170 µm, 18h, pH 4* 117

***Supplementary Table 52**: Raw data Extended Data Fig. 5j – nucleobases in water, 170 µm, 18h, pH 5* 118

***Supplementary Table 53**: Raw data Extended Data Fig. 5k – nucleobases in water, 170 µm, 18h, pH 7* 119

***Supplementary Table 54**: Raw data Extended Data Fig. 5l – nucleobases in water, 170 µm, 18h, pH 10* 120

***Supplementary Table 55**: Raw data Extended Data Fig. 5m – nucleobases in water, 170 µm, 18h, pH 11* 121

***Supplementary Table 56**: Raw data Extended Data Fig. 5n – 2',3'-Ribonucleotides in water, 170µm, 18h* 122

***Supplementary Table 57**: Raw data Extended Data Fig. 5o – 3',5'-Ribonucleotides in water, 170µm, 18h* 123

***Supplementary Table 58**: Raw data Extended Data Fig. 5p – 3',5'-Ribonucleotides in 10 % formamide, 170µm, 18h* 124

***Supplementary Table 59**: Raw data Extended Data Fig. 5q – cytidine and CMPs in water, 170µm, 18h* 125

***Supplementary Table 60**: Raw data Extended Data Fig. 5r – Deoxy- vs 5'-Ribonucleotides in water, 170µm, 18h* 126

***Supplementary Table 61**: Raw data Extended Data Fig. 5s-u – Chirality of amino acids and nucleoside in water, 170µm, 18h* 128

***Supplementary Table 62**: Raw data Supplementary Fig. 1a – nucleobases in water, 170µm, 6h* . 129

***Supplementary Table 63**: Raw data Supplementary Fig. 1b – Ribonucleosides in water, 170µm, 6h* 130

***Supplementary Table 64**: Raw data Supplementary Fig. 1c – 5'-Ribonucleotides in water, 170µm, 6h* 131

***Supplementary Table 65**: Raw data Supplementary Fig. 1d – 2',3'-Ribonucleotides in water, 170µm, 6h* 132

***Supplementary Table 66**: Raw data Supplementary Fig. 15* 133

Supplementary references 134

Calibration data amino acids (Fig. 2) 135

Calibration data amino acids (Fig. 4 – 0 K and 16 K) 155

Calibration data dimerization of glycine (Fig. 5) 166

Supplementary Discussions

***Supplementary Discussion 1**: Stability of the thermogravitational accumulation against temporal fluctuations of the temperature gradient and behaviour in the limit of small temperature gradients.*

While the stability of thermogravitational accumulation to changes in chamber geometry[41] as well as spatial fluctuations in heat flux[42] has already been demonstrated, we here explore the stability of the process to temporal fluctuations in heat flux.

Using finite element simulations (Comsol 5.4), we simulate a 2-dimensional closed heat flow chamber with a height of 50 mm and a width of 0.17 mm. To show the temporal stability to heat flux fluctuations, we simulate a proxy species with a diffusion coefficient of D = 800e-12 m^2/s and a Soret coefficient of S_T = 5.7e-3 1/K, which is in the range of the compounds studied in this work. The simulated differential equations correspond to those given in the method section (Determination of thermophoretic strength) but are now solved simultaneously to account for the time variance of the applied temperature difference. The cold side is kept at a constant 30 °C, while the warm side is varied as shown in Supplementary Fig. 12. The simulation is run for 14 h.

The results depicted in Supplementary Fig. 12 for constant temperature differences of $\Delta T = 10$ K and $\Delta T = 20$ K show, as expected, a non-linear increase of the strength of the thermogravitational accumulation with ΔT at the point of maximum accumulation (cold, lower corner). If instead a variable temperature difference is applied, here exemplarily with a sinusoidal function of amplitude 10 K (thus varying between 0 and 20 K) and periodicity (Supplementary Fig. 12: black solid line), the average accumulation characteristic is the same as for a constant temperature difference of 12 K (Supplementary Fig. 12: orange).

The time-averaged temperature difference gives a value of 10 K. The deviation from the accumulation with a constant temperature difference of 10 K (Supplementary Fig. 12: blue) is due to the nonlinear dependence of the thermogravitational accumulation on the temperature difference. Temperature differences above 10 K therefore contribute disproportionately more to the accumulation compared to those below 10 K. Despite the very strong temporal fluctuations of the heat flux, the accumulation process under consideration shows to be extremely stable.

The exact determination of the temperature gradients occurring in natural systems, for example in fissures in basaltic glass, is difficult. Therefore, a discussion of the possible enrichments by heat flux driven chambers at very small temperature differences as well as the natural conditions, which also make larger temperature gradients appear realistic, is relevant. As shown in the main text in Figure 4f, even in small networks with very moderate temperature gradients of only 2 K, an enhancement of e.g. I vs N of (3.5 ± 0.2)-fold can be achieved.

Figure 4f shows that these values can be further boosted by the size of the system, but our calculations here are limited by the measurement accuracy of the Soret coefficients involved. Due to the accumulating error of the heat flux chambers connected in series, a calculation of systems N>30 is no longer reliably possible. However, we expect here a continuation of the behavior shown in Figure 4f and thus a compensation of the smaller temperature differences discussed here.

Even larger temperature gradients are likely to be realistic within natural systems. Varying across orders of magnitude in fracture size and associated widely varying flow velocities, fast flows can serve as effective heat sources and sinks for narrower adjacent channels[49,55], implementing a wide range of possible temperature differences in which the effects shown can take place. Also, it should be noted here that the temperature gradients shown here, as discussed above, need not act all the time and could also fluctuate over the local dimension (for a stability analysis over local fluctuations, see reference 42). In summary, the systems discussed here may well be considered realistic and thus may have made a relevant contribution to prebiotic chemistry.

Supplementary Figures

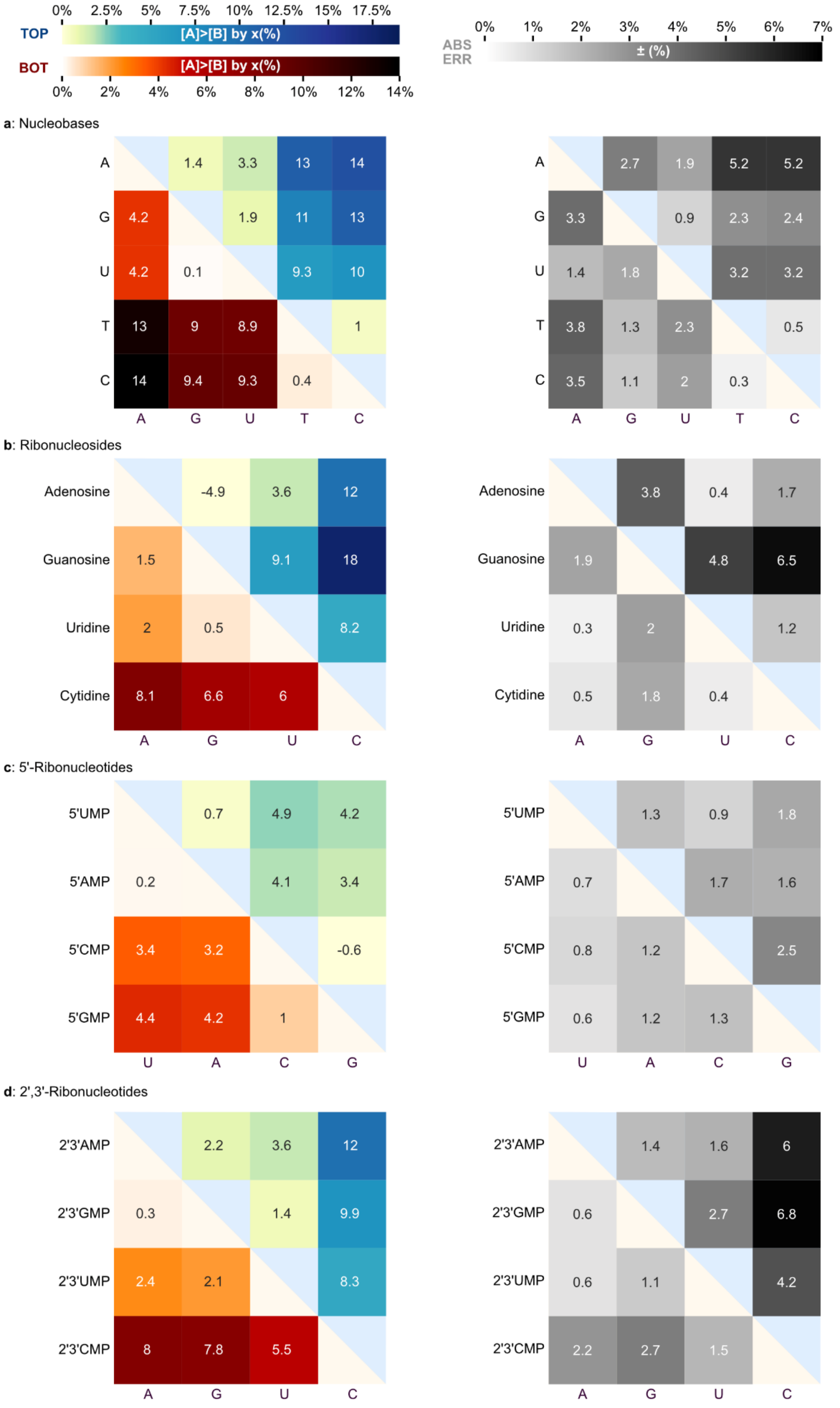

***Supplementary Figure 1**: Enrichment of nucleobases, nucleosides, 5'-nucleotides and 2',3'-cyclic nucleotides after 6 h.*
The tendencies are the same as after 18 h (Fig. 3a/c/d, main text and Extended Data Fig. 5n) though at lowered amplitudes. Error maps show s.d. (3 repeats, for raw data see Supplementary Tables 5-65).

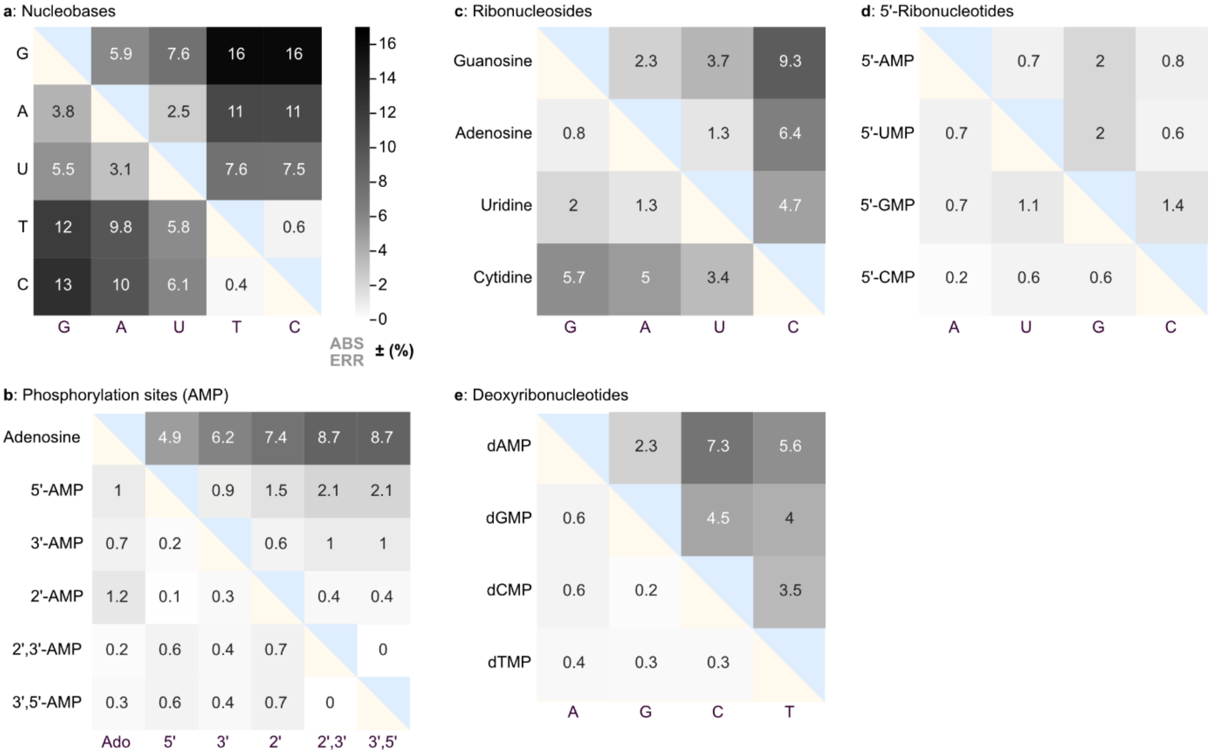

Supplementary Figure 2*: Error maps for Figure 3.*
Error maps show s.d. (3 repeats, for raw data see Supplementary Tables 5-65).

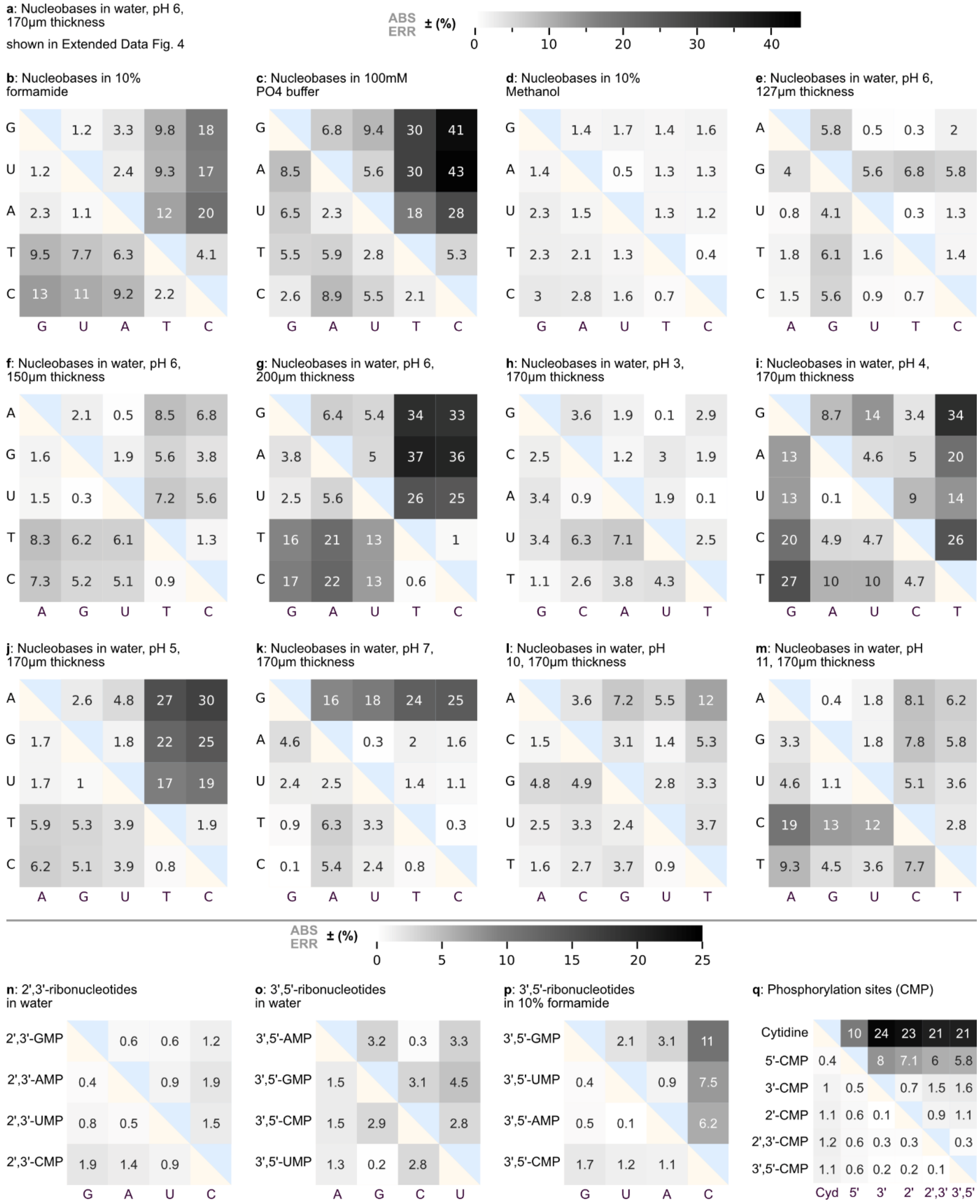

***Supplementary Figure 3**: Error maps for Extended Data Fig. 5.*
Error maps show s.d. (3 repeats, for raw data see Supplementary Tables 5-65).

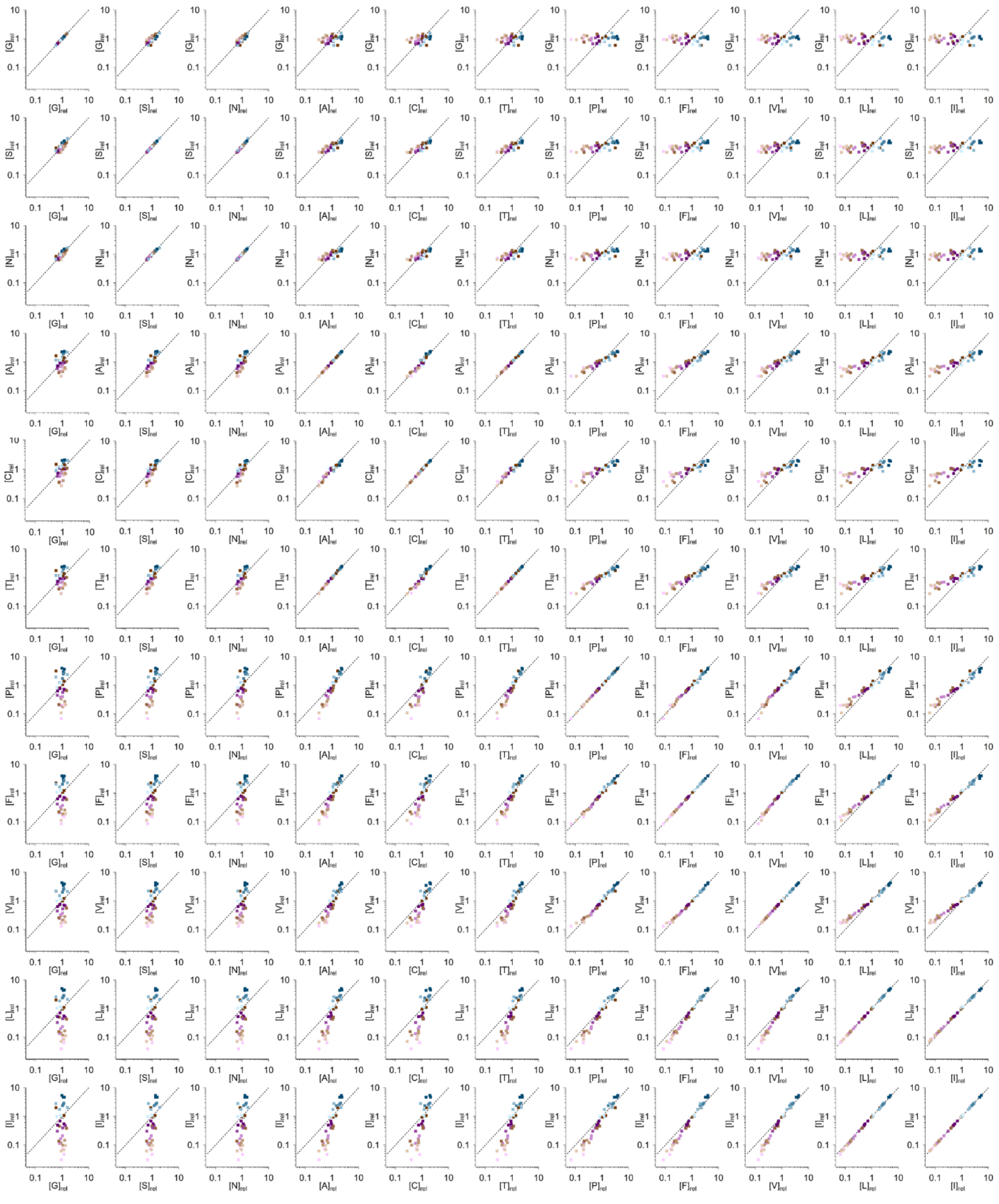

***Supplementary Figure 4**: Separation of amino acids in experimental network*
Experimental setup of a small network of three interconnected chambers with an volume-inflow of 1 nl/s of the same AA-mixture used in Figure 4 and ΔT=16 K. After 60 h, the chamber contents from 3 repeats were frozen and divided into individual parts according to the colour gradations shown in Supplementary Figure 5 and measured by HPLC. In addition to examples shown in the main text Fig. 4 and Extended Data Fig. 6, we show various combinations of pairs. For instance, thermophoretically different AAs glycine (G) and isoleucine (I) separate readily while mass-identical leucine (L) and I only show minor concentration differences in our experimental system.

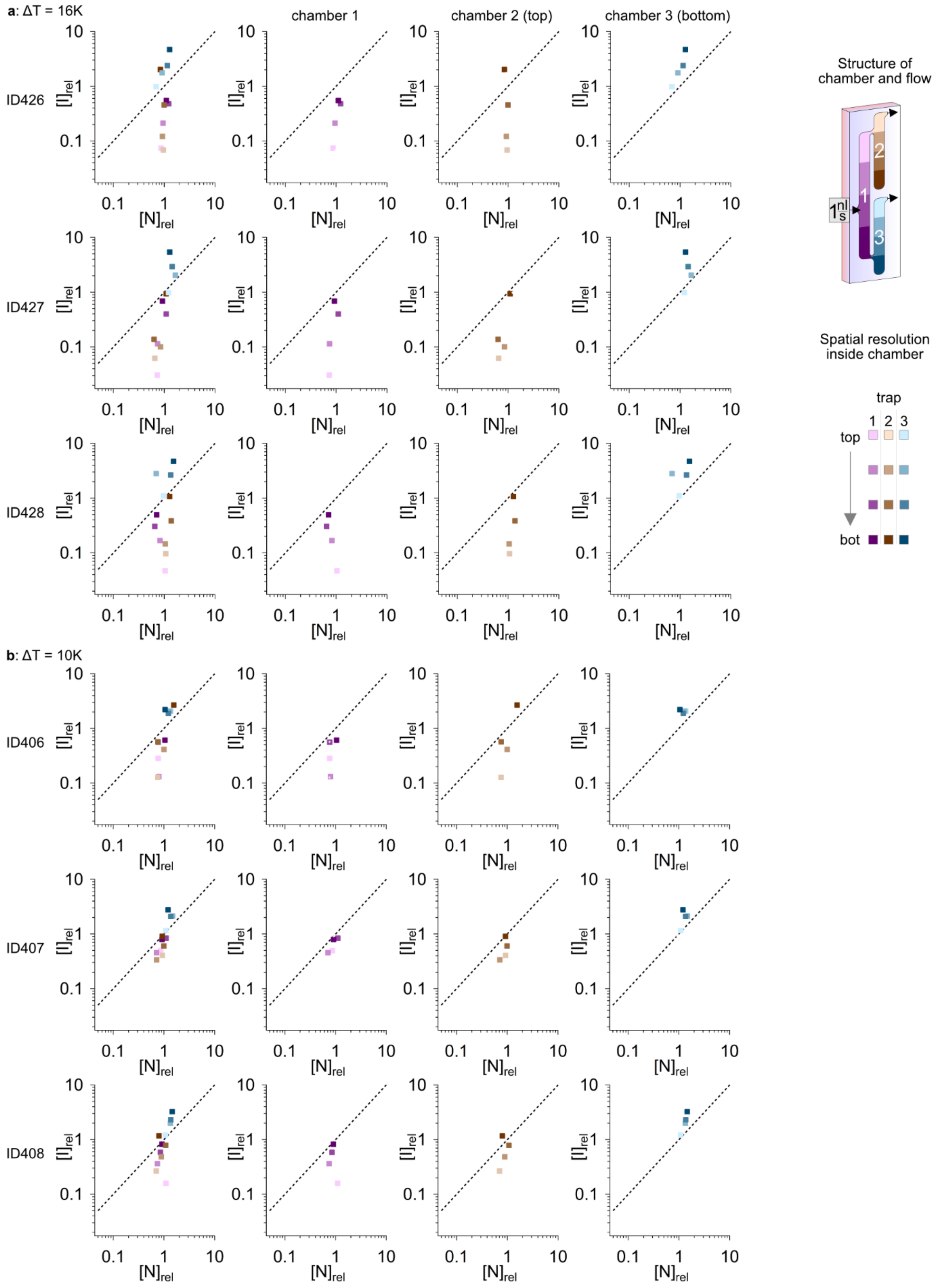

***Supplementary Figure 5**: Individual experiments and per-chamber depiction of network experiments*

Experimental setup of a small network of three interconnected chambers with an volume-inflow of 1 nl/s of the same AA-mixture used in Figure 4 with a ΔT of ***(a)*** *16K and* ***(b)*** *10K. After 60 h, the chamber contents from 3 repeats at each temperature gradient were frozen and*

divided into individual parts according to the colour gradations shown on the right side. For the AA pair I vs N, the concentrations per repeat and per individual chambers are shown.

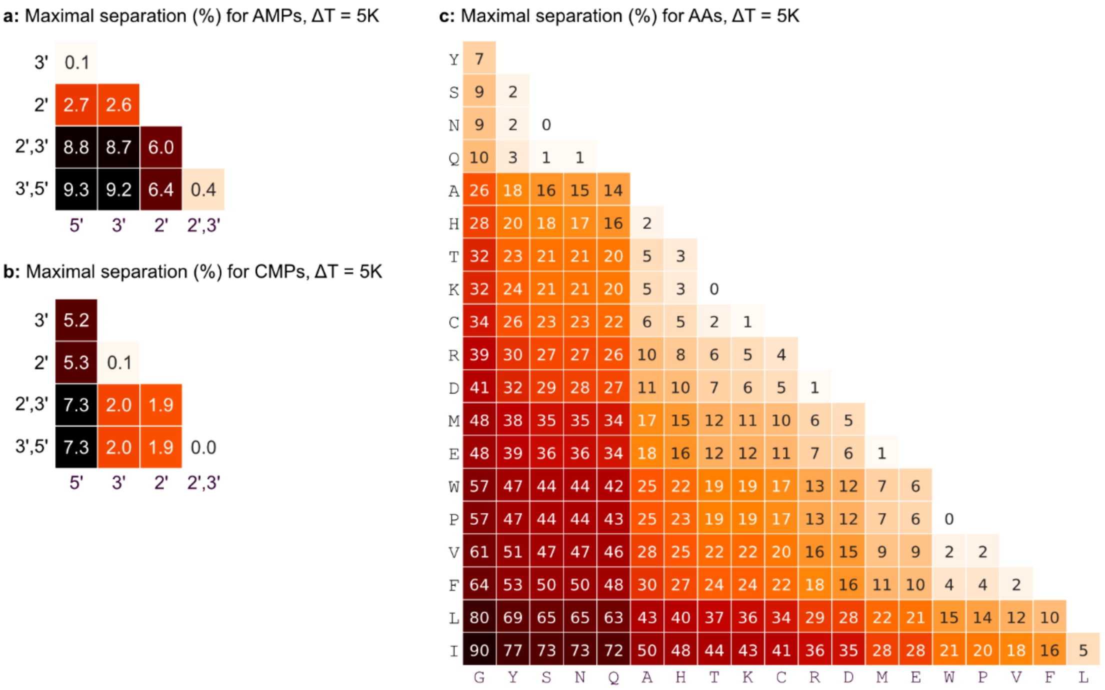

***Supplementary Figure 6**: Maximal pairwise separation for various species.*
As shown in Extended Data Fig. 2, the experimental restriction to average over 25 % of the heat flux chamber results in lowered enrichment values. The maximal pairwise separation values shown here are calculated by numerically finding the steady state solution for a set temperature gradient of 5 K, fixing the top of a chamber to 1 and taking the value from the lowest mm of the chamber. ***(a/b)*** *shows the maximal pairwise separation for adenosine and cytidine monophosphates, showing separation of up to 5 % between species of identical mass.* ***(c)*** *Similarly, for amino acids, up to 90 % separation are feasible.*

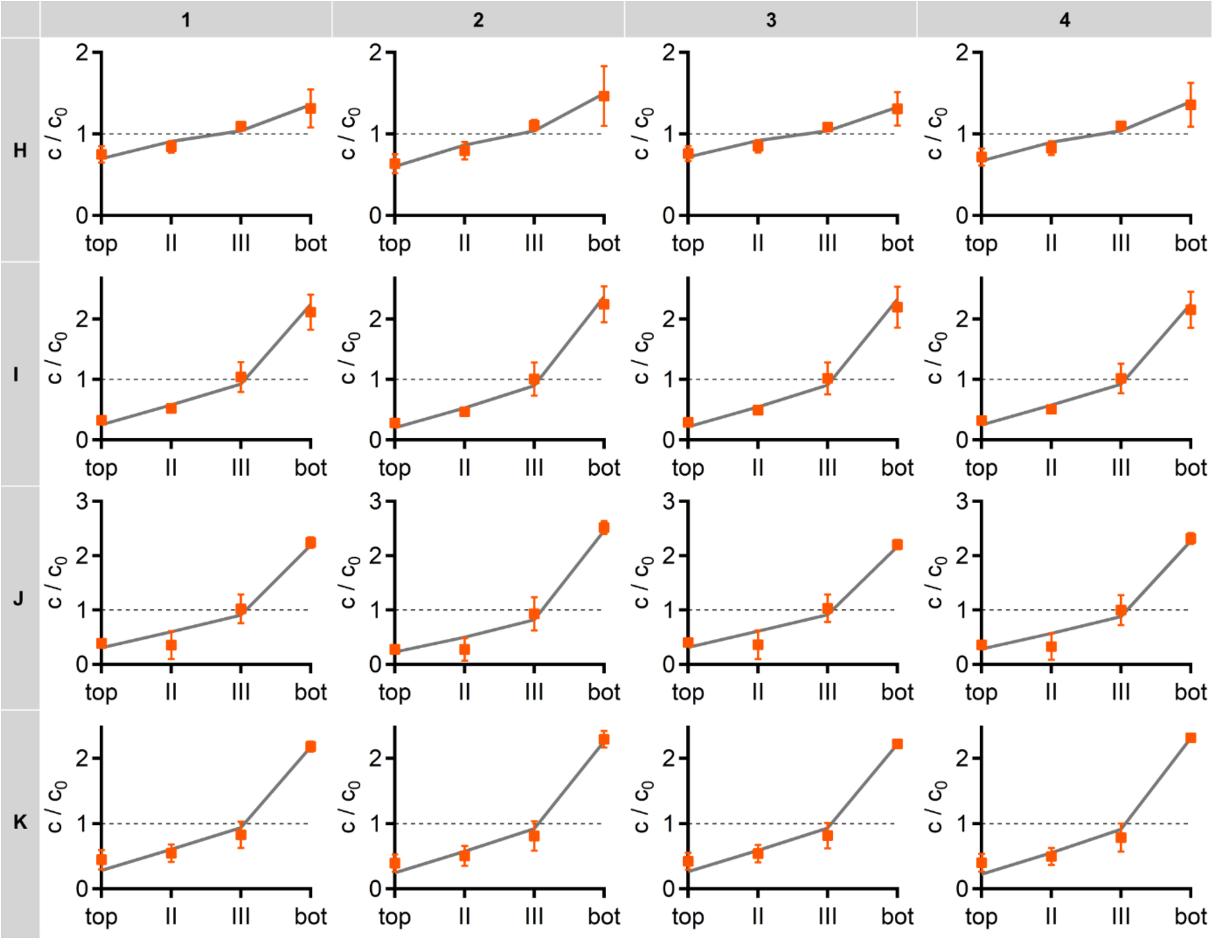

***Supplementary Figure 7**: Concentration profiles in heat flux cell and fit for Soret coefficient. In addition to data shown in Extended Data Fig. 8 (errors=s.d., 3 repeats). **H1**: Adenosine, **H2**: Cytidine, **H3**: Guanosine, **H4**: Uridine, **I1**: 5'-AMP, **I2**: 5'-CMP, **I3**: 5'-GMP, **I4**: 5'-UMP, **J1**: 2',3'-AMP, **J2**: 2',3'-CMP, **J3**: 2',3'-GMP, **J4**: 2',3'-UMP, **K1**: 3',5'-AMP, **K2**: 3',5'-CMP, **K3**: 3',5'-GMP, **K4**: 3',5'-UMP.*

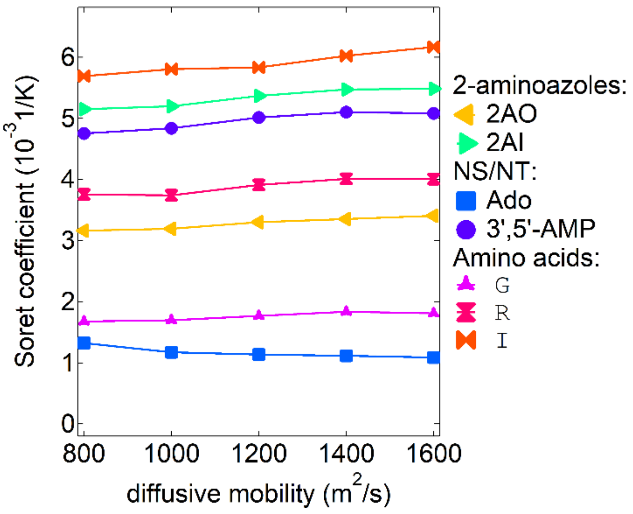

***Supplementary Figure 8**: Influence of diffusion constant on determination of Soret coefficient for different exemplary species.*
For details on determination of the Soret coefficient, see Methods.

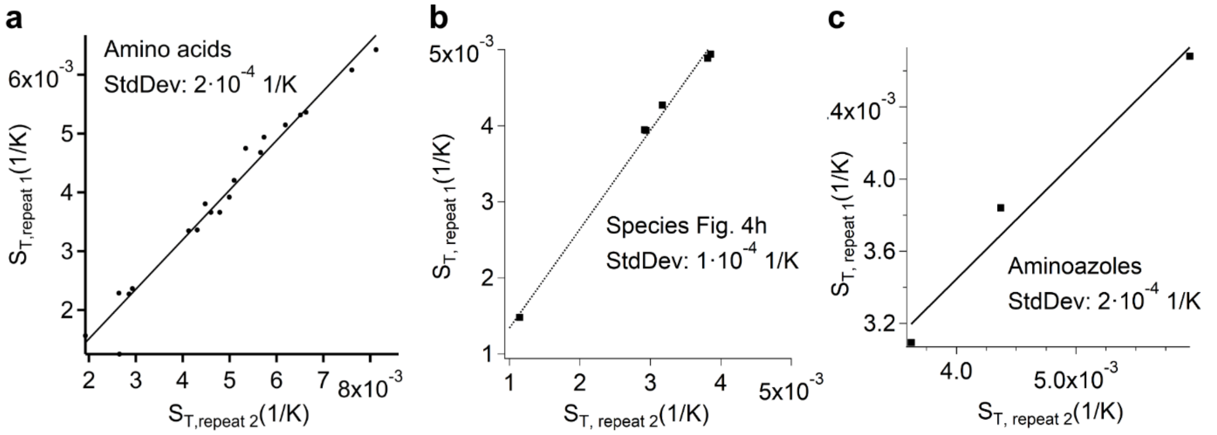

Supplementary Figure 9*: Errors in Soret coefficients for network models.*
To separate random from systematic errors, measurements of Soret coefficients for each of 2 repeats are shown in ***a-c****. Systematic errors here are smaller differences in the temperature gradients between different measurements. These deviations affect the Soret coefficients of all species present in the respective mixtures equally, so they lead to a slope different from 1 in these correlation plots. The random error, e.g. by the integral determination of the peaks, then results from the standard deviation of the linear fits of these correlations (shown here as solid lines).*

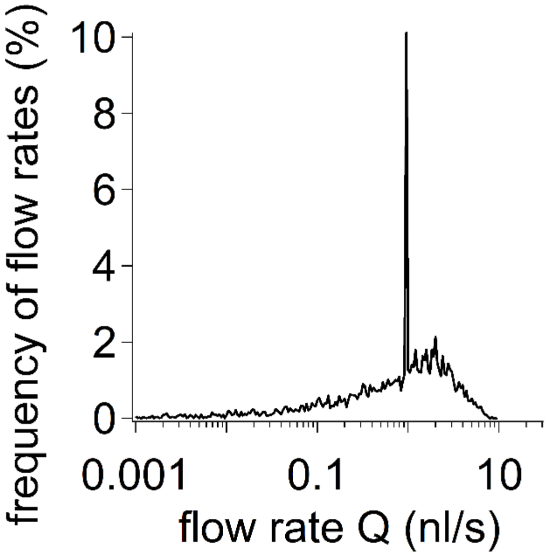

Supplementary Figure 10*: Example distribution of flow rates for a network.*
Example for system of 20 by 20 heat flux traps over 30 iterations. The input flow rate was set to 1 nl/s, which is why it is the most common. The maximum flow rates were limited to 10 nl/s, the smallest to $1 \cdot 10^{-3}$ *nl/s.*

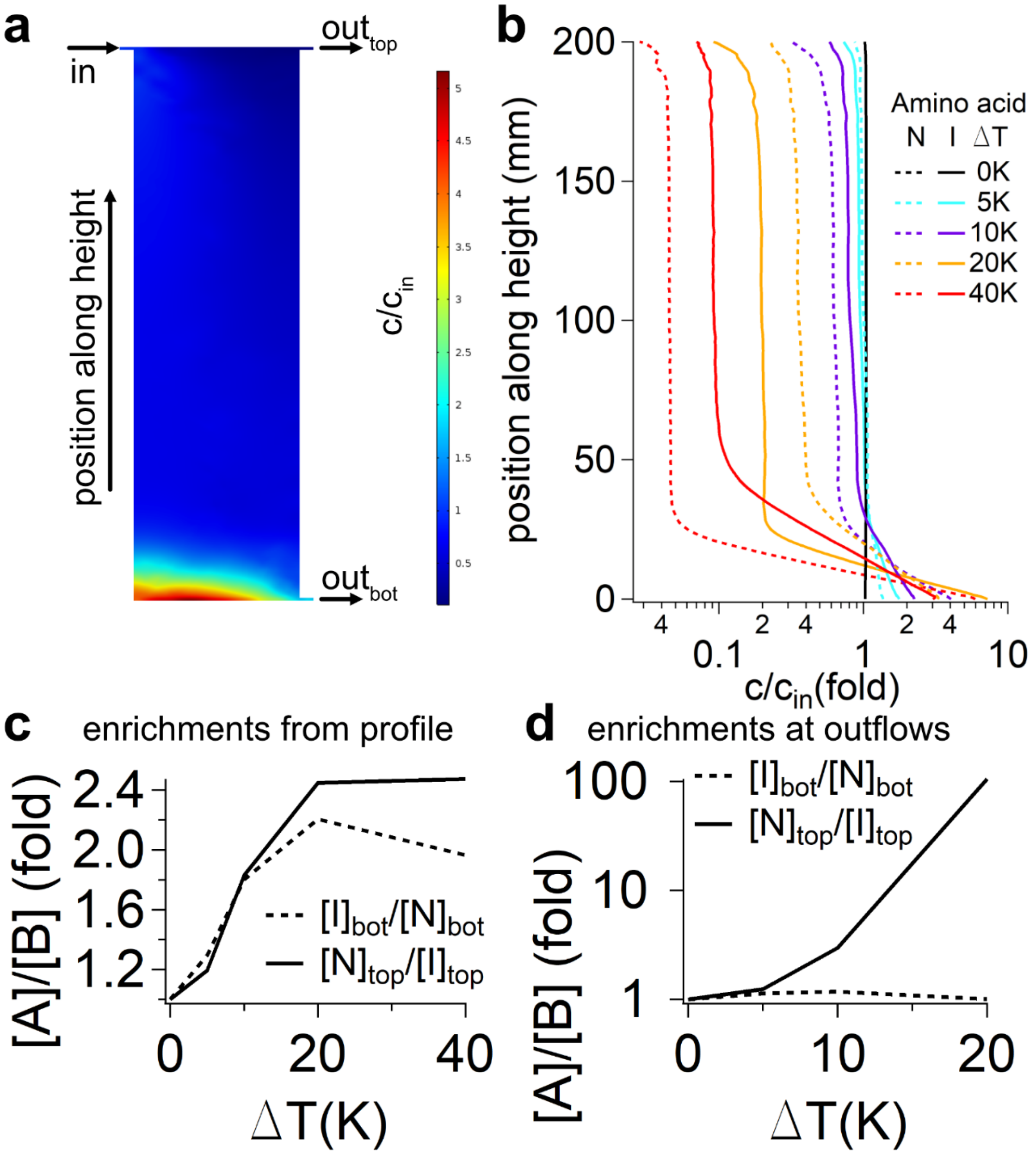

***Supplementary Figure 11**: Example of enrichment behavior in a single heat-flow chamber as used in the network simulation.*

*As in main text Figures 4 and 5 ($\Delta T = 10\ K, v_{in} = 1\ \frac{nl}{s}, v_{out,bot} = 0.5\ \frac{nl}{s}$, height=200 mm, width=60mm, thickness=0.17mm, amino acids: I and N). **(a)** Concentration of AA I within the complete heat flow chamber. The concentration distribution is distorted by the fluid flow toward to top and bottom outlet. **(b)** Projection of concentrations of AAs I (solid) and N (dashed) relative to the inflow concentration (c_{in}) along the height axis of the chamber for different temperature differences ΔT. **(c)** Increasing temperature differences lead to larger concentration ratios between AAs I and N at the top position of the chamber (at 200mm) compared to the bottom section of the chamber (at 0 mm). **(d)** Concentration ratios between the AAs I and N at the top and bottom outlet positions. The outward fluid flow toward the bottom and top change the concentration ratios to even more extreme values. This effect leads to the observed dependence on the temperature difference in a larger network and scales with the network size.*

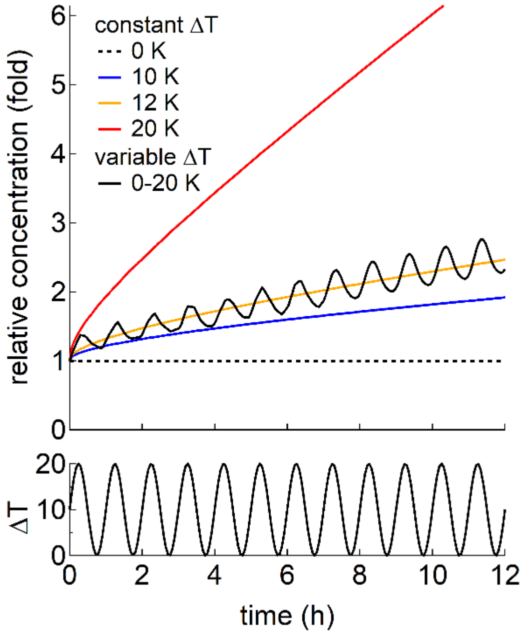

Supplementary Figure 12*: Stability of thermogravitational accumulation against fluctuations of the applied temperature gradients.*

Relative concentration increase at the bottom/cold spot of the heat flow chamber is plotted versus the accumulation time. Plots that show scenarios with constant temperature difference (blue, orange, red, dashed black) show the exponential increase in accumulation efficiency. The solid black plot shows the scenario with a varying temperature difference (sine function with 10 K amplitude and 1 h periodicity).

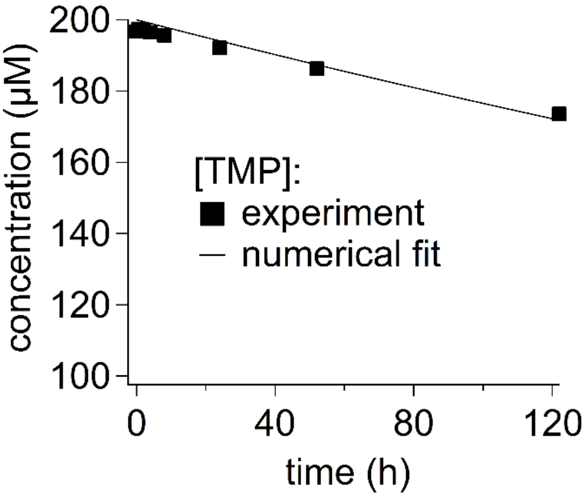

Supplementary Figure 13*: TMP hydrolysis under reaction conditions*
Time series at 90 °C and at an initial concentration of 200 µM and pH 10.5 (as indicated in Methods) over a period of 120 h (errors=s.d., 3 repeats). The numerical fit corresponds to the solution of Eq. 18. The reaction rate was determined to be $k_4 = 3.5 \cdot 10^{-7}\ (1/s)(\pm 19\%)$.

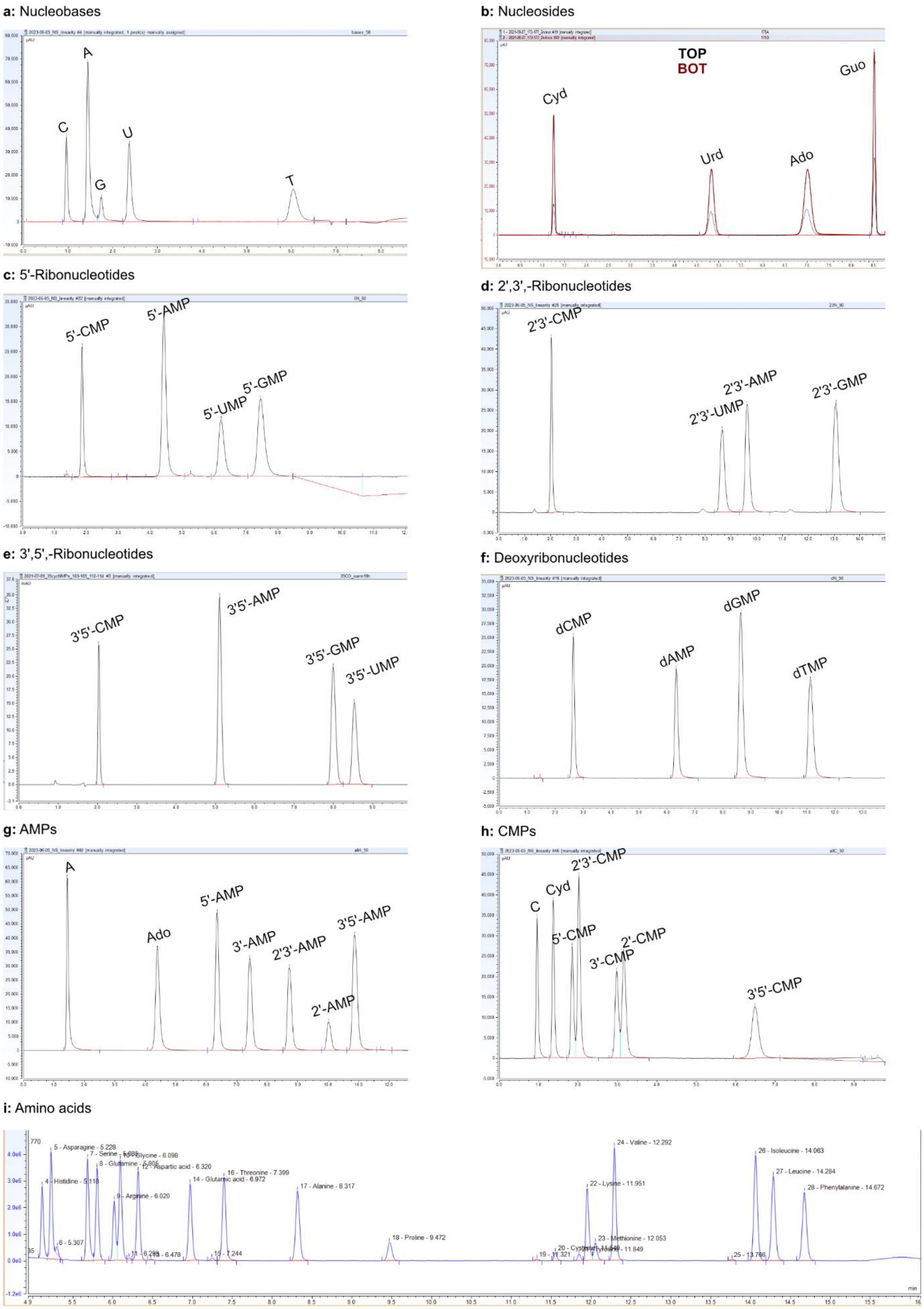

***Supplementary Figure 14**: Separation of molecules in LC methods*
In (b) we show exemplary chromatograms for the extracted top (black) and bottom (red) fractions.

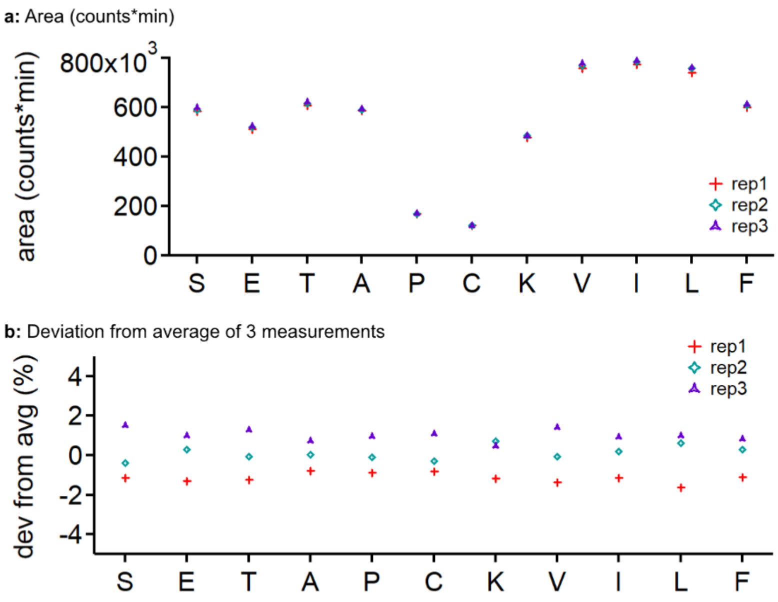

Supplementary Figure 15*: Reproducibility of amino acid detection in LC method described in Methods.*

We separated a sample of amino acids into 3 parts and prepared each individually according to the pre-column derivatization introduced in Methods to check for potential errors. For a selection of amino acids, we show ***(a)*** *the measured peak integral and* ***(b)*** *the deviation from the average peak integral of the 3 measurements.*

Supplementary Tables

Supplementary Table 1: *List of abbreviations used.*

**Abbreviation**	**Full name**
2AO	2-aminooxazole
2AI	2-aminoimidazole
2AT	2-aminothiazole
A	Adenine
C	Cytosine
G	Guanine
T	Thymine
U	Uracil
Ado	D-Adenosine
Cyd / D-Cyd	D-Cytidine (dextrorotary)
L-Cyd	L-Cytidine (levorotary)
Guo	D-Guanosine
Urd	D-Uridine
2’,3’-AMP	2’,3’-cyclic Adenosine monophosphate
2’,3’-CMP	2’,3’-cyclic Cytidine monophosphate
2’,3’-GMP	2’,3’-cyclic Guanosine monophosphate
2’,3’-UMP	2’,3’-cyclic Uridine monophosphate
2’-AMP	Adenosine 2’-monophosphate
2’-CMP	Cytidine 2’-monophosphate
3’,5’-AMP	3’,5’-cyclic Adenosine monophosphate
3’,5’-CMP	3’,5’-cyclic Cytidine monophosphate
3’,5’-GMP	3’,5’-cyclic Guanosine monophosphate
3’,5’-UMP	3’,5’-cyclic Uridine monophosphate
3’-AMP	Adenosine 3’-monophosphate
3’-CMP	Cytidine 3’-monophosphate
5’-AMP	Adenosine 5’-monophosphate
5’-CMP	Cytidine 5’-monophosphate
5’-GMP	Guanosine 5’-monophosphate
5’-UMP	Uridine 5’-monophosphate
dAMP	2’-Deoxyadenosine 5’-monophosphate
dCMP	2’-Deoxcytidine 5’-monophosphate
dGMP	2’-Deoxyguanosine 5’-monophosphate
dTMP	Thymidine 5’-monophosphate
PO_4	Orthophosphate (PO_4)
diPO_4	Pyrophosphate (P_2O_7)
TMP	Trimetaphosphate (P_3O_9)
triPO_4	Triphosphate (P_3O_{10})
**Extended Data Fig. 2 and Supplementary Tables 23-28**	
Ans	Anserine
Arg	Arginine
Asp	Aspartic acid
Car	Carnosine
Cit	Citrulline
Cre	Creatinine

Cys	Cysteine
Cth	Cystathionine
ETA	Ethanolamine
Glu	Glutamic acid
AABA	Homoalanine
Hcy	Homocysteine
His	Histidine
Hyl	Hydroxylysine
Hyp	Hydroxyproline
Leu-Ile	Leucine + Isoleucine
Lys	Lysine
Met	Methionine
3-MH	Methylhistidine
Orn	Ornithine
Phe	Phenylalanine
Pro	Proline
Sar	Sarcosine
Ser	Serine
Tau	Taurine
Thr	Threonine
Tyr	Tyrosine
Val	Valine

***Supplementary Table 2**: Enrichment against mean concentration of set of molecules. When not stated, conditions are: 18 h in water. Starting from a mean concentration of $\bar{c}_0$, at the top (j=top), the average concentration $\bar{c}_{top} = \frac{1}{S}\sum_i \bar{c}_{i,top}$ (see Methods, Eq. 3) of all species $i: 1,2,..,S$ decreases as the solutes are enriched by thermophoretic accumulation at the bottom. The average bottom concentration $\bar{c}_{bot} = \frac{1}{S}\sum_i c_{i,bot}$ increases accordingly (see Methods, Eq. 3). Errors=s.d., 3 repeats.*

2-aminoazoles, different temperature gradients							
Setting	fraction j	$\overline{c_j/c_0}$	2AI	2AO	2AT		
5K	top	0.79	(-6.3±0.4)%	(6.9±1.2)%	(-0.5±1.6)%		
	bot	1.26	(2.8±1.2)%	(-3.1±1.5)%	(0.3±2.7)%		
10K	top	0.65	(-9.2±8.1)%	(10.9±6.2)%	(-1.7±4.1)%		
	bot	1.51	(5.5±3.8)%	(-6.8±2.5)%	(1.2±1.3)%		
18K	top	0.29	(-43±14)%	(29.5±0.9)%	(14±13)%		
	bot	2.08	(16.5±4.0)%	(-11.9±0.8)%	(-4.7±4.6)%		
Nucleobases, nucleosides, nucleotides							
Setting	fraction j	$\overline{c_j/c_0}$	A	C	G	T	U
NB	top	0.69	(16.3±3.5)%	(-20.4±4.4)%	(18.1±7.2)%	(-19.7±4.6)%	(5.7±1.4)%
	bot	1.46	(-11.6±3.6)%	(14.7±4.7)%	(-12.4±5.0)%	(14.3±4.4)%	(-5.1±1.0)%
rNS	top	0.72	(5.0±0.9)%	(-11.5±3.9)%	(6.5±3.3)%		(0.1±0.4)%
	bot	1.36	(-3.3±1.4)%	(7.0±3.3)%	(-3.5±1.9)%		(-0.2±0.1)%
5'-NMP	top	0.30	(6.1±0.7)%	(-7.3±0.1)%	(-4.2±1.2)%		(5.3±0.7)%
	bot	2.18	(-2.9±0.3)%	(3.1±0.1)%	(0.8±0.6)%		(-1.0±0.5)%
dNMP	top	0.19	(11.1±3.2)%	(-7.6±3.0)%	(5.1±1.2)%	(-8.6±2.1)%	
	bot	2.73	(-3.1±0.4)%	(2.1±0.2)%	(-1.8±0.2)%	(2.8±0.1)%	
2',3'-NMP	top	0.36	(8.9±0.6)%	(-22.4±0.7)%	(13.0±0.0)%		(0.5±0.5)%
	bot	2.32	(-3.4±0.4)%	(8.5±1.0)%	(-5.0±0.7)%		(-0.1±0.1)%
3',5'-NMP	top	0.41	(6.9±1.2)%	(-5.3±0.8)%	(1.9±2.4)%		(-3.5±2.3)%
	bot	2.27	(-3.2±0.3)%	(1.7±1.8)%	(-1.4±1.1)%		(2.9±1.0)%
3',5'-NMP 10% Form.	top	0.16	(2.6±0.5)%	(-23.1±3.5)%	(14.5±2.9)%		(6.0±0.7)%
	bot	3.14	(-0.5±0.1)%	(4.5±1.0)%	(-2.7±0.6)%		(-1.2±0.3)%
Nucleobases in various pH & buffers							
Setting	fraction j	$\overline{c_j/c_0}$	A	C	G	T	U
pH3	top	0.65	(8.8±0.6)%	(0.4±1.8)%	(20.6±1.4)%	(-31.0±0.3)%	(1.2±1.3)%
	bot	1.34	(-10.9±2.3)%	(-13.9±1.5)%	(-16.8±0.5)%	(34.9±0.1)%	(6.7±3.5)%
pH4	top	0.80	(3.8±0.5)%	(0.6±5.4)%	(16.9±9.7)%	(-20±12)%	(-1.8±3.8)%
	bot	1.27	(-3.3±1.1)%	(8.0±3.5)%	(-16±10)%	(13.4±8.7)%	(-2.1±1.0)%
pH5	top	0.47	(26.6±7.2)%	(-30.7±6.9)%	(25.0±4.0)%	(-30.3±6.0)%	(9.4±1.7)%
	bot	1.59	(-15.1±2.1)%	(17.9±2.3)%	(-13.6±1.7)%	(17.4±2.3)%	(-6.6±1.0)%
pH7	top	0.68	(16.3±3.7)%	(-20.3±3.4)%	(17.2±14.6)%	(-17.3±3.8)%	(4.0±3.6)%
	bot	1.29	(-8.4±3.1)%	(9.8±1.2)%	(-8.4±1.0)%	(9.1±2.0)%	(-2.2±1.1)%
pH10	top	0.48	(33.1±4.5)%	(-10.0±1.0)%	(10.7±2.9)%	(-27.1±2.5)%	(-6.7±0.4)%

	bot	1.97	(-20.0±1.0)%	(-4.6±2.2)%	(-4.0±2.6)%	(19.6±0.5)%	(9.1±0.7)%
pH11	top	0.79	(17.7±2.5)%	(-10.1±3.6)%	(10.0±2.4)%	(-14.2±2.2)%	(-3.4±0.6)%
	bot	1.25	(-21.2±4.1)%	(14.7±8.7)%	(-11.3±2.4)%	(16.2±2.0)%	(1.6±2.9)%
127µm	top	0.80	(9.3±1.8)%	(-12.5±0.7)%	(10.4±4.6)%	(-11.1±1.2)%	(3.9±1.2)%
	bot	1.22	(-7.1±0.5)%	(9.2±1.2)%	(-6.9±3.4)%	(8.1±1.8)%	(-3.3±0.3)%
150µm	top	0.65	(17.2±2.6)%	(-22.0±1.8)%	(19.0±0.4)%	(-20.9±2.8)%	(6.7±1.9)%
	bot	1.49	(-11.9±2.9)%	(14.3±2.7)%	(-10.0±1.6)%	(13.5±3.6)%	(-5.9±1.8)%
200µm	top	0.54	(27.0±9.8)%	(-32.9±8.7)%	(30.6±6.7)%	(-31.5±9.6)%	(6.8±3.9)%
	bot	1.38	(-13.9±7.4)%	(17.1±8.0)%	(-14.0±4.9)%	(15.6±7.6)%	(-4.9±3.5)%
10% Formamide	top	0.26	(7.2±4.8)%	(-29.6±5.6)%	(27.7±2.1)%	(-15.0±4.0)%	(9.7±2.7)%
	bot	2.29	(-5.0±2.3)%	(17.4±6.0)%	(-13.0±4.0)%	(8.4±3.4)%	(-7.7±3.2)%
100mM PO4	top	0.40	(34.2±7.9)%	(-33.2±9.2)%	(9±10)%	(-29.1±6.6)%	(19.1±2.5)%
	bot	1.81	(-14.3±3.7)%	(21.9±2.5)%	(-16.2±3.2)%	(17.6±0.5)%	(-9.0±1.9)%
10% Methanol	top	0.94	(1.4±0.7)%	(-2.0±0.7)%	(1.9±1.1)%	(-1.5±0.7)%	(0.2±0.8)%
	bot	1.10	(-1.6±1.3)%	(2.4±1.5)%	(-2.4±1.5)%	(1.9±0.8)%	(-0.3±0.8)%
Nucleobases, nucleotides after 6 hours							
Setting	fraction *j*	$\overline{c_j/c_0}$	A	C	G	T	U
NB	top	0.84	(6.0±2.7)%	(-7.1±1.9)%	(4.6±0.4)%	(-6.1±1.9)%	(2.6±1.0)%
	bot	1.21	(-6.6±2.0)%	(6.3±0.9)%	(-2.8±1.1)%	(5.9±1.2)%	(-2.7±0.9)%
rNS	top	0.70	(2.3±0.7)%	(-8.7±2.0)%	(7.7±3.6)%		(-1.3±1.0)%
	bot	1.30	(-2.8±0.5)%	(5.1±0.4)%	(-1.4±1.4)%		(-0.9±0.6)%
5'-NMP	top	0.65	(1.7±0.8)%	(-2.4±1.2)%	(-1.7±1.4)%		(2.4±0.6)%
	bot	1.56	(-1.8±0.7)%	(1.4±0.7)%	(2.3±0.7)%		(-2.0±0.1)%
2',3'-NMPs	top	0.57	(4.3±1.5)%	(-6.9±3.7)%	(2.0±2.5)%		(0.6±0.4)%
	bot	1.67	(-2.6±0.6)%	(5.2±1.5)%	(-2.3±1.0)%		(-0.3±0.1)%

Supplementary Table 3*: List of coefficients of determination, slopes and standard deviations of linear fits of LC and IC methods*

The coefficient of determination is calculated as $R^2 = 1 - \frac{\sum_{i=1}^{n}(y_i - f(x_i))^2}{\sum_{i=1}^{n}(y_i - \bar{y})^2}$ *with* y_i *the measured values,* $f(x_i)$ *the predicted value from the fitted slope,* $\bar{y}$ *the mean value of the measured slope and i=1…n the individual calibration measurements. The values are used for measurements of nucleobases, nucleosides, nucleotides, 2-aminoazoles and trimetaphosphate as described in Methods.*

Set of molecules	Species	R^2 (linear fit)	Slope (linear fit)	Error (linear fit)
Nucleobases (µAU*s/µM)	A	0.999946	6817.31	25.10
	C	0.999991	2729.11	4.00
	G	0.999861	1295.07	7.64
	U	0.999958	4331.60	13.97
	T	0.999992	3820.38	5.38
Nucleosides (µAU*s/µM)	A	0.999969	8478.40	23.59
	C	0.999966	3545.42	10.37
	G	0.999954	7947.92	26.81
	U	0.999960	5435.43	17.15
5'-Ribonucleotides (µAU*s/µM)	A	0.999963	6266.74	19.04
	C	0.999958	2773.21	8.99
	G	0.999969	5044.92	14.06
	U	0.999975	3047.74	7.68
2',3'-Ribonucleotides (µAU*s/µM)	A	0.999984	6285.21	12.51
	C	0.999974	4551.70	11.50
	G	0.999976	7937.32	19.31
	U	0.999981	5272.32	11.57
3',5'-Ribonucleotides (µAU*s/µM)	A	0.999971	8257.57	25.88
	C	0.999980	3428.16	8.93
	G	0.998556	5910.68	129.79
	U	0.997852	6211.76	166.41
Deoxyribo-nucleotides (µAU*s/µM)	A	0.999981	3402.14	7.41
	C	0.999981	3171.54	6.87
	G	0.999956	6302.40	20.87
	T	0.999968	4478.07	12.57
AMPs (µAU*s/µM)	2'-AMP	0.999980	2050.85	4.64
	3'-AMP	0.999981	6633.92	14.35
CMPs (µAU*s/µM)	2'-CMP	0.999934	3878.25	18.24
	3'-CMP	0.999957	2745.52	10.42
2AZ (nAU*min/µM)	2AI	0.975984	8492.38	769.13
	2AO	0.994395	8922.18	386.75
	2AT	0.999316	2222.47	33.57

***Supplementary Table 4**: List of coefficients of determination, slopes and standard deviations of linear fits of LC methods for amino acids.*
Exemplary sets of calibrations are shown on pages 135-165. ED refers to Extended Data; S refers to the Supplementary Information. In each category, F references to figures, T to tables. The calibration used for the analysis of Glycine dimerization is partially shown here and partially at the end of the Supplementary Information. R^2 is defined as in Supplementary Table 3.

for measurements:			Species	R^2	slope (linear fit) counts*min/µM, W / Y: mAU*min/µM Gly / GlyGly: µAU*min/mM	range of calibration (µM)	
Fig.	Ext.	Suppl.				min	max
2	EDF3 EDF8 EDT1	SF7 ST6 ST29 ST32	A	0.984	4753	5	75
			C	0.984	415	5	75
			D	0.973	4625	5	75
			E	0.982	4335	5	75
			F	0.992	5432	5	75
			G	0.989	5555	5	75
			H	0.991	3299	5	75
			I	0.989	6911	5	75
			K	0.980	3632	5	75
			L	0.989	5928	5	75
			M	0.993	1106	5	75
			N	0.988	5076	5	75
			P	0.989	1452	5	75
			Q	0.990	4727	5	75
			R	0.991	3134	5	75
			S	0.990	4926	5	75
			T	0.991	5476	5	75
			V	0.982	6529	5	75
			W	0.987	0.0076	5	75
			Y	0.991	0.0080	5	75
4 (0K 16K)	EDF6	SF4 SF5 ST14 ST15	A	0.985	11963	2	500
			C	0.977	1507	2	500
			F	0.986	15084	2	500
			G	0.987	15002	2	500
			I	0.993	17736	2	500
			L	0.987	18440	2	500
			N	0.995	13338	2	500
			P	0.987	3898	2	500
			S	0.992	14064	2	500
			T	0.987	16080	2	500
			V	0.992	16030	2	500
4 (10K)		SF5 ST14 ST15	A	0.997	18781	2	500
			C	0.978	3558	2	500
			F	0.997	19950	2	500
			I	0.997	26016	2	500
			L	0.998	23086	2	500
			N	0.998	17121	2	500

			P	0.998	5922	2	500
			T	0.997	20305	2	500
			V	0.992	23707	2	500
	EDF4	ST35<br>ST38<br>ST41	A	0.969	14386	5	60
			C	0.975	1385	5	60
			D	0.966	11099	5	60
			E	0.970	10808	5	60
			F	0.977	15219	5	60
			H	0.956	8977	5	60
			I	0.976	20888	5	60
			K	0.975	10569	5	60
			L	0.951	18271	5	60
			N	0.979	11051	5	60
			P	0.976	4190	5	60
			Q	0.974	11513	5	60
			S	0.948	14032	5	60
			T	0.972	15502	5	60
			V	0.974	19218	5	60
5c left		ST18	Gly	0.995	617.4	500	20000
			GlyGly	0.981	341.3	2.5	10000
5b<br>5c right		ST17<br>ST20	Gly	0.998	see pages 166-167 (in counts*min vs mM)		
			GlyGly	0.992			

***Supplementary Table 5**: Raw data Fig. 2a – 2-aminoazoles in water, 170 µm, 18 h, 18 K.*
Per repeat (k), fraction ($j \in \{top, II, III, bot\}$) and species, we analyse the HPLC data to obtain "measured values". Using the calibration shown in Supplementary Table 3, we calculate the corresponding "concentration (µM)" $[A]_{j,k,HPLC}$. Per species and repeat, we calculate an average total concentration over all fraction (c_0) which we use to normalize our data ("$[A]_{j,k}$ concentration (x c_0)") to be able to compare runs and species with different concentrations. Per fraction, we can thus determine a mean concentration $\bar{c}_{j,k}$. Against this mean concentration, we calculate the "ratio species vs mean" (as introduced in Eq. 4 in the Methods). The averages and errors of those are shown in Supplementary Table 2. Furthermore, we directly compare species against species (right-most columns) for the top and bottom fraction. The average and standard deviations of those are shown in the heat maps in Fig. 2-3, Extended Data Fig. 2-5 and Supplementary Fig. 1-3.

repeat k	species	measured values (mAU*min)				concentration (µM) $[A]_{j,k,HPLC}$ calibration acc. to Supplementary Table 3				c_0 (µM)	Normalization (Eq. 1) $[A]_{j,k}$ concentration (x c_0)				Ratio species vs mean (Eq. 4) $[A]_{j,k}/\bar{c}_{j,k} - 1$ (%)		Ratio species against species (shown in heat maps), Eq. 2 $[A]_{j,k}/[B]_{j,k} - 1$ (%)							
		top	II	III	bot	top	II	III	bot		top	II	III	bot	top	bot	top part (blue shade)				bot part (red shade)			
1	2AO	0.143	0.278	0.329	0.647	15.98	31.17	36.92	72.5	39.14	0.41	0.8	0.94	1.85	29.08	-10.72	2AO	0	1.61	194.19	2AO	0	0.15	-26.56
	2AT	0.031	0.051	0.082	0.141	13.81	23.13	36.99	63.58	34.38	0.4	0.67	1.08	1.85	27.04	-10.85	2AT	-1.58	0	189.52	2AT	-0.15	0	-26.67
	2AI	0.050	0.204	0.279	0.910	5.9	24.02	32.9	107.2	42.51	0.14	0.57	0.77	2.52	-56.12	21.57	2AI	-66.01	-65.46	0	2AI	36.16	36.37	0
	mean concentration per fraction $\bar{c}_{j,k}$										0.32	0.68	0.93	2.07				2AO	2AT	2AI		2AO	2AT	2AI
2	2AO	0.179	0.309	0.494	0.752	20.03	34.62	55.42	84.34	48.6	0.41	0.71	1.14	1.74	28.76	-12.19	2AO	0	34.73	70.15	2AO	0	-12.45	-21.53
	2AT	0.027	0.050	0.102	0.176	12.19	22.41	45.85	79.01	39.87	0.31	0.56	1.15	1.98	-4.43	0.29	2AT	-25.77	0	26.3	2AT	14.22	0	-10.38
	2AI	0.125	0.234	0.563	1.141	14.71	27.58	66.33	134.3	60.73	0.24	0.45	1.09	2.21	-24.33	11.9	2AI	-41.23	-20.82	0	2AI	27.44	11.58	0
	mean concentration per fraction $\bar{c}_{j,k}$										0.32	0.58	1.13	1.98				2AO	2AT	2AI		2AO	2AT	2AI
3	2AO	0.150	0.284	0.550	0.910	16.81	31.8	61.64	102.03	53.07	0.32	0.6	1.16	1.92	30.77	-12.68	2AO	0	9.92	160.23	2AO	0	-9.52	-24.84
	2AT	0.030	0.049	0.115	0.220	13.41	22.05	51.79	98.85	46.52	0.29	0.47	1.11	2.12	18.97	-3.49	2AT	-9.02	0	136.74	2AT	10.52	0	-16.93
	2AI	0.070	0.162	0.595	1.467	8.22	19.08	70.07	172.7	67.52	0.12	0.28	1.04	2.56	-49.75	16.18	2AI	-61.57	-57.76	0	2AI	33.05	20.38	0
	mean concentration per fraction $\bar{c}_{j,k}$										0.24	0.45	1.1	2.2				2AO	2AT	2AI		2AO	2AT	2AI

***Supplementary Table 6**: Raw data Fig. 2b and Extended Data Fig. 3 – AA in water, 170 µm, 18 h, pH 7.4.*
Samples were injected 2-3 times and the mean concentration was used to calculate the normalized concentrations and enrichments (following tables). Calibrations are shown in Supplementary Table 4 and at the end of the Supplementary Information (pages 135-154).

repeat k	species	measured values (counts*min) for W and Y: mAU*min				measured values (counts*min) for W and Y: mAU*min				measured values (counts*min) for W and Y: mAU*min				concentration (µM) $[A]_{j,k,HPLC}$ calibration acc. to Supplementary Table 4				c_0 (µM)	Normalization (Eq. 1) concentration (x c_0)				Ratio species vs mean (Eq. 4) $[A]_{j,k}/\bar{c}_{j,k} - 1$ (%)	
		top	II	III	bot	top	II	III	bot	top	II	III	bot	top	II	III	bot		top	II	III	bot	top	bot
1	H	34236	65431	113293	132221	36382	67925	118823	138230					10.70	20.21	35.18	40.98	26.77	0.40	0.75	1.31	1.53	11.3	-3.7
	N	68489	105096	169019	166371	71260	110150	178744	173497					13.77	21.20	34.26	33.48	25.68	0.54	0.83	1.33	1.30	49.3	-18.0
	S	73581	114278	178598	181273	76036	120354	187252	190450					15.19	23.81	37.13	37.73	28.46	0.53	0.84	1.30	1.33	48.5	-16.7
	Q	63435	97669	157164	154095	64696	102286	164591	161260					13.55	21.15	34.03	33.36	25.52	0.53	0.83	1.33	1.31	47.8	-17.8
	R	30462	63237	109290	141583	31657	65929	113552	148480					9.91	20.60	35.55	46.27	28.08	0.35	0.73	1.27	1.65	-1.8	3.6
	G	91090	135703	185619	182435	95079	141649	195603	192302					16.76	24.96	34.31	33.73	27.44	0.61	0.91	1.25	1.23	70.0	-22.7
	D	35610	62979	163833	172103	37590	65133	172021	182014					7.91	13.85	36.31	38.28	24.09	0.33	0.57	1.51	1.59	-8.5	-0.1
	E	29726	57542	155133	170993	30598	61041	164472	178843					6.96	13.68	36.86	40.35	24.46	0.28	0.56	1.51	1.65	-20.8	3.7
	T	54663	99848	192941	214999	53579	104756	203098	224493					9.88	18.68	36.16	40.13	26.21	0.38	0.71	1.38	1.53	5.0	-3.7
	A	47345	83438	170334	175737	50012	87447	178964	183333					10.24	17.98	36.75	37.77	25.69	0.40	0.70	1.43	1.47	11.0	-7.5
	P	8710	20337	50488	64711	9326	21305	52630	66842					6.21	14.34	35.50	45.29	25.33	0.25	0.57	1.40	1.79	-31.8	12.4
	C	4076	8385	15561	17618	4181	8545	16678	19315					9.95	20.41	38.86	44.52	28.44	0.35	0.72	1.37	1.57	-2.5	-1.6
	K	31694	56463	134067	129117	32450	58361	138858	134901					8.83	15.81	37.57	36.34	24.64	0.36	0.64	1.52	1.48	-0.2	-7.2
	M	6888	16098	38357	45872	7019	16598	40168	48109					6.29	14.79	35.51	42.50	24.77	0.25	0.60	1.43	1.72	-29.3	7.9
	V	49349	103411	246673	292075	44521	88469	226343	297284					7.19	14.70	36.23	45.14	25.81	0.28	0.57	1.40	1.75	-22.5	10.0
	I	29670	79746	234895	330117	30791	84457	247654	346622					4.37	11.88	34.91	48.96	25.03	0.17	0.47	1.39	1.96	-51.3	23.0
	L	28663	73764	203375	278285	29959	77718	214416	289835					4.94	12.78	35.24	47.92	25.22	0.20	0.51	1.40	1.90	-45.4	19.5
	F	35543	84698	186591	262090	37403	88338	196470	274163					6.71	15.93	35.26	49.36	26.82	0.25	0.59	1.31	1.84	-30.3	15.7
	Y	0.1188	0.1912	0.2877	0.3170	0.1274	0.1990	0.3057	0.3325					15.33	24.30	36.95	40.44	29.26	0.52	0.83	1.26	1.38	45.9	-13.1
	W	0.0387	0.1114	0.2711	0.3642	0.0408	0.1147	0.2789	0.3730					5.25	14.94	36.34	48.71	26.31	0.20	0.57	1.38	1.85	-44.4	16.4

	mean concentration per fraction $\bar{c}_{j,k}$																		0.36	0.68	1.38	1.59		
	H	43629	60589	93184	194818	25819	43891	90408	207775	46676	64625	98510	205958	11.73	17.08	28.50	61.48	29.70	0.40	0.58	0.96	2.07	11.4	-7.1
	N	90482	115634	153551	248928	62984	91687	156481	254336	97421	122929	161592	262087	16.48	21.69	30.97	50.26	29.85	0.55	0.73	1.04	1.68	55.7	-24.5
	S	95317	123204	164412	254838	90422	102615	163231	260112	102092	131070	172767	268052	19.48	24.15	33.86	52.98	32.62	0.60	0.74	1.04	1.62	68.4	-27.1
	Q	83358	107017	144161	234753	55562	82727	145056	237905	88579	114626	150951	247805	16.04	21.46	31.04	50.81	29.84	0.54	0.72	1.04	1.70	51.7	-23.6
	R	35737	51001	86741	203339	18669	34963	84069	222142	38662	55318	89470	214384	9.90	15.02	27.68	68.05	30.16	0.33	0.50	0.92	2.26	-7.4	1.2
	G	123544	146465	179840	239970	114254	128383	180146	248600	132831	154857	188421	252725	22.24	25.78	32.91	44.48	31.35	0.71	0.82	1.05	1.42	100.1	-36.4
	D	46298	73032	129731	308275	26408	44469	113589	291130	49836	77235	134498	324495	8.83	14.03	27.23	66.58	29.17	0.30	0.48	0.93	2.28	-14.6	2.4
	E	38821	62346	115078	313900	19239	34829	97313	285283	40848	64954	120228	326894	7.61	12.47	25.58	71.21	29.22	0.26	0.43	0.88	2.44	-26.6	9.3
	T	70161	98107	158385	349426	39464	66395	150411	351760	72379	101213	166505	369338	11.08	16.17	28.93	65.16	30.34	0.37	0.53	0.95	2.15	3.0	-3.7
	A	67293	92053	145650	295295	43869	65056	137537	284783	71008	97650	153592	310232	12.78	17.87	30.63	62.44	30.93	0.41	0.58	0.99	2.02	16.5	-9.4
2	P	12134	18734	35990	111473	5475	9982	31678	106404	13131	18853	37702	117014	7.05	10.92	24.18	76.86	29.75	0.24	0.37	0.81	2.58	-33.1	15.9
	C	5710	7225	12602	28045	2544	4845	12659	31132	5540	7922	12610	28322	11.09	16.07	30.44	70.32	31.98	0.35	0.50	0.95	2.20	-2.2	-1.4
	K	48300	67309	112484	243019	31150	48685	108965	234861	50704	70400	117969	257243	11.94	17.10	31.15	67.46	31.91	0.37	0.54	0.98	2.11	5.6	-5.2
	M	12164	15570	29897	77878	6872	10858	27181	77624	12180	17351	33883	80715	9.41	13.20	27.43	71.22	30.31	0.31	0.44	0.90	2.35	-12.4	5.4
	V	50087	74047	178470	538090	29094	54198	120561	468598	47095	74663	173715	581482	6.45	10.36	24.14	81.09	30.51	0.21	0.34	0.79	2.66	-40.4	19.2
	I	39149	64450	142840	594453	15019	29241	115029	541826	41558	68166	150649	627295	4.62	7.81	19.70	85.06	29.30	0.16	0.27	0.67	2.90	-55.5	30.2
	L	37666	60526	130280	496892	15492	29806	107533	459164	40127	62812	136081	523036	5.25	8.61	21.02	83.17	29.51	0.18	0.29	0.71	2.82	-49.9	26.4
	F	42452	65479	130634	414791	17710	35356	114510	417078	44966	69669	135717	437900	6.45	10.46	23.37	77.92	29.55	0.22	0.35	0.79	2.64	-38.4	18.3
	Y																							
	W	0.0731	0.1049	0.2152	0.5432	0.0250	0.0662	0.1840	0.5677	0.0668	0.1049	0.2136	0.5636	7.27	12.15	26.99	73.76	30.04	0.24	0.40	0.90	2.46	-31.8	10.1
	mean concentration per fraction $\bar{c}_{j,k}$																		0.35	0.51	0.91	2.23		
	H	35350	73935	103728	175591	36558	77148	110076	186128					10.90	22.90	32.40	54.82	30.25	0.36	0.76	1.07	1.81	11.2	-8.2
	N	81941	132925	167352	222500	85843	139006	177123	236965					16.53	26.79	33.93	45.26	30.63	0.54	0.87	1.11	1.48	66.5	-25.2
3	S	93402	136689	192163	246511	97023	142205	202762	259661					19.33	28.31	40.08	51.37	34.77	0.56	0.81	1.15	1.48	71.5	-25.2
	Q	73063	123660	156793	211947	75909	127758	165093	221778					15.76	26.59	34.05	45.88	30.57	0.52	0.87	1.11	1.50	59.1	-24.0
	R	27317	65857	95518	181800	28193	68498	100065	190266					8.85	21.43	31.20	59.35	30.21	0.29	0.71	1.03	1.96	-9.5	-0.5

	G	121545	154734	196642	221589	124914	161870	206731	233403					22.18	28.50	36.31	40.95	31.98	0.69	0.89	1.14	1.28	114.0	-35.1
	D	42114	103188	154527	311047	42916	107627	161062	326898					9.19	22.79	34.11	68.96	33.76	0.27	0.67	1.01	2.04	-16.0	3.4
	E	30518	88417	140549	305997	30976	89566	149182	321422					7.09	20.53	33.42	72.37	33.35	0.21	0.62	1.00	2.17	-34.4	9.9
	T	56282	122256	178663	321170	56449	127759	188812	334837					10.29	22.83	33.55	59.90	31.64	0.33	0.72	1.06	1.89	0.4	-4.1
	A	59467	115234	167029	281046	60598	120031	176522	294781					12.63	24.75	36.14	60.58	33.53	0.38	0.74	1.08	1.81	16.3	-8.5
	P	9099	25360	40619	102215	9667	26562	42898	106486					6.46	17.87	28.75	71.84	31.23	0.21	0.57	0.92	2.30	-36.2	16.5
	C	4219	10235	14776	27015	4420	10921	15432	26913					10.42	25.50	36.42	65.01	34.34	0.30	0.74	1.06	1.89	-6.4	-4.1
	K	37249	87710	124982	233825	37523	90725	132188	243587					10.29	24.56	35.40	65.72	33.99	0.30	0.72	1.04	1.93	-6.6	-2.1
	M	7032	19225	29882	69113	7263	20065	32168	70894					6.47	17.77	28.06	63.32	28.90	0.22	0.61	0.97	2.19	-31.0	11.0
	V	49902	113462	173147	475252	33435	107393	189191	483698					6.38	16.91	27.75	73.44	31.12	0.21	0.54	0.89	2.36	-36.7	19.5
	I	22592	90859	163044	536241	24144	94571	170034	563344					3.38	13.42	24.10	79.55	30.11	0.11	0.45	0.80	2.64	-65.3	33.8
	L	23437	83940	146347	448525	23688	88143	152199	468736					3.97	14.51	25.18	77.37	30.26	0.13	0.48	0.83	2.56	-59.5	29.5
	F	28822	89481	143122	368553	28586	91937	150325	386964					5.28	16.70	27.01	69.55	29.64	0.18	0.56	0.91	2.35	-45.0	18.9
	Y	0.1420	0.2239	0.2962	0.4419	0.1475	0.2360	0.3183	0.4660					18.03	28.64	38.26	56.54	35.37	0.51	0.81	1.08	1.60	57.3	-19.0
	W	0.0378	0.1355	0.2259	0.4963	0.0342	0.1349	0.2123	0.4979					4.76	17.87	28.96	65.69	29.32	0.16	0.61	0.99	2.24	-49.9	13.5
	mean concentration per fraction $\bar{c}_{j,k}$																		0.32	0.69	1.01	1.97		
	H	45825	85648	88616	148568	47842	89163	93376	157360					14.19	26.49	27.58	46.36	28.66	0.50	0.92	0.96	1.62	13.4	-4.8
	N	74937	131331	122000	180905	79025	138552	129201	191844					15.17	26.59	24.75	36.72	25.80	0.59	1.03	0.96	1.42	34.6	-16.3
	S	77666	132596	124999	191592	81677	139553	131414	203252					16.17	27.62	26.02	40.07	27.47	0.59	1.01	0.95	1.46	34.8	-14.2
	Q	68950	124182	114137	171366	73007	128812	119140	179753					15.02	26.76	24.68	37.14	25.90	0.58	1.03	0.95	1.43	32.8	-15.6
	R	42921	84426	95049	170742	44641	89001	100092	179842					13.97	27.66	31.13	55.92	32.17	0.43	0.86	0.97	1.74	-0.6	2.3
4	G	98021	144752	141205	193846	104081	150061	148111	204671					18.19	26.54	26.04	35.87	26.66	0.68	1.00	0.98	1.35	56.3	-20.8
	D	43266	109459	88802	181863	45505	114368	94100	191598					9.60	24.20	19.77	40.37	23.48	0.41	1.03	0.84	1.72	-6.4	1.2
	E	38122	104017	85082	181290	39488	107962	89152	191252					8.95	24.45	20.10	42.97	24.12	0.37	1.01	0.83	1.78	-15.0	4.8
	T	66431	141188	132638	233667	69393	148258	139691	244864					12.40	26.43	24.87	43.69	26.85	0.46	0.98	0.93	1.63	5.8	-4.2
	A	54449	116983	105478	181257	57956	123308	110571	190832					11.83	25.28	22.73	39.14	24.74	0.48	1.02	0.92	1.58	9.4	-6.9
	P	13645	34311	33444	69074	14044	35694	35403	72416					9.53	24.10	23.70	48.71	26.51	0.36	0.91	0.89	1.84	-17.7	8.1

	C	5453	12645	12359	21289	5702	12977	12485	22114					13.45	30.89	29.95	52.32	31.65	0.42	0.98	0.95	1.65	-2.7	-2.7
	K	32266	87705	70471	134611	33523	90963	73529	140568					9.06	24.59	19.82	37.88	22.84	0.40	1.08	0.87	1.66	-9.2	-2.4
	M	10610	25518	25436	50758	10880	27832	27958	52680					9.72	24.13	24.15	46.78	26.19	0.37	0.92	0.92	1.79	-15.0	5.1
	V	57791	188823	144903	324768	55011	155562	151191	330095					8.64	26.38	22.68	50.15	26.96	0.32	0.98	0.84	1.86	-26.6	9.5
	I	50777	150472	146717	368152	53103	158181	154632	389284					7.52	22.33	21.80	54.80	26.61	0.28	0.84	0.82	2.06	-35.3	21.2
	L	46307	132160	128911	308729	48924	138774	135242	325708					8.03	22.85	22.28	53.51	26.67	0.30	0.86	0.84	2.01	-31.0	18.1
	F	57999	136276	145757	313618	60901	142057	152601	328707					10.94	25.62	27.46	59.13	30.79	0.36	0.83	0.89	1.92	-18.6	13.0
	Y	0.1457	0.2411	0.2609	0.4034	0.1480	0.2561	0.2756	0.4251					18.29	30.96	33.41	51.60	33.56	0.54	0.92	1.00	1.54	24.8	-9.5
	W	0.0633	0.1845	0.2065	0.4332	0.0689	0.1974	0.2205	0.4531					8.74	25.23	28.21	58.56	30.19	0.29	0.84	0.93	1.94	-33.7	14.2
	mean concentration per fraction $\bar{c}_{j,k}$																		0.44	0.95	0.91	1.70		

***Supplementary Table 7**: Raw data Fig. 2b and Extended Data Fig. 3 – AA in water, 170 µm, 18 h, pH 7.4 – enrichments in top fraction Shown in the top right triangle.*

repeat		species	Ratio species against species (shown in heat maps), Eq. 2 $[A]_{j,k}/[B]_{j,k} - 1$ (%)																			
1	A	H	0.0	-25.4	-25.1	-24.7	13.3	-34.5	21.7	40.6	6.0	0.3	63.1	14.2	11.6	57.5	43.5	128.8	103.9	59.7	-23.7	100.3
		N	34.1	0.0	0.5	1.0	51.9	-12.2	63.2	88.5	42.2	34.5	118.7	53.2	49.6	111.2	92.5	206.8	173.5	114.1	2.3	168.6
		S	33.4	-0.5	0.0	0.5	51.2	-12.6	62.4	87.6	41.5	33.8	117.7	52.4	48.9	110.1	91.5	205.3	172.1	113.1	1.8	167.2
		Q	32.8	-1.0	-0.5	0.0	50.5	-13.0	61.6	86.7	40.8	33.2	116.6	51.7	48.2	109.2	90.7	203.9	170.8	112.1	1.3	166.0
		R	-11.7	-34.2	-33.9	-33.5	0.0	-42.2	7.4	24.1	-6.4	-11.5	44.0	0.8	-1.5	39.0	26.7	101.9	80.0	40.9	-32.7	76.8
		G	52.7	13.9	14.5	15.0	73.1	0.0	85.9	114.7	62.0	53.1	149.1	74.5	70.4	140.5	119.2	249.4	211.5	143.9	16.5	205.9
		D	-17.8	-38.7	-38.4	-38.1	-6.9	-46.2	0.0	15.5	-12.9	-17.6	34.0	-6.1	-8.3	29.4	18.0	88.0	67.6	31.2	-37.3	64.6
		E	-28.9	-46.9	-46.7	-46.4	-19.4	-53.4	-13.4	0.0	-24.6	-28.7	16.0	-18.7	-20.6	12.0	2.1	62.8	45.1	13.6	-45.7	42.5
		T	-5.7	-29.7	-29.3	-29.0	6.8	-38.3	14.8	32.6	0.0	-5.4	53.8	7.7	5.2	48.5	35.4	115.8	92.3	50.6	-28.0	88.9
		A	-0.3	-25.6	-25.3	-24.9	13.0	-34.7	21.4	40.2	5.8	0.0	62.7	13.9	11.3	57.1	43.2	128.2	103.4	59.2	-23.9	99.8
		P	-38.7	-54.3	-54.1	-53.8	-30.5	-59.9	-25.4	-13.8	-35.0	-38.5	0.0	-30.0	-31.6	-3.5	-12.0	40.3	25.0	-2.1	-53.2	22.8
		C	-12.4	-34.7	-34.4	-34.1	-0.8	-42.7	6.6	23.1	-7.2	-12.2	42.8	0.0	-2.3	37.9	25.7	100.3	78.5	39.8	-33.2	75.4
		K	-10.4	-33.2	-32.8	-32.5	1.6	-41.3	9.1	26.0	-4.9	-10.1	46.2	2.4	0.0	41.2	28.7	105.1	82.8	43.1	-31.6	79.5
		M	-36.5	-52.6	-52.4	-52.2	-28.1	-58.4	-22.7	-10.7	-32.7	-36.3	3.6	-27.5	-29.2	0.0	-8.8	45.3	29.5	1.4	-51.5	27.2
		V	-30.3	-48.1	-47.8	-47.5	-21.1	-54.4	-15.2	-2.1	-26.1	-30.2	13.6	-20.4	-22.3	9.7	0.0	59.4	42.1	11.2	-46.8	39.5
		I	-56.3	-67.4	-67.2	-67.1	-50.5	-71.4	-46.8	-38.6	-53.7	-56.2	-28.7	-50.1	-51.2	-31.2	-37.3	0.0	-10.9	-30.2	-66.6	-12.5
		L	-51.0	-63.4	-63.2	-63.1	-44.4	-67.9	-40.3	-31.1	-48.0	-50.8	-20.0	-44.0	-45.3	-22.8	-29.6	12.2	0.0	-21.7	-62.6	-1.8
		F	-37.4	-53.3	-53.1	-52.8	-29.0	-59.0	-23.8	-12.0	-33.6	-37.2	2.2	-28.5	-30.1	-1.4	-10.1	43.3	27.7	0.0	-52.2	25.4
		Y	31.1	-2.3	-1.8	-1.3	48.5	-14.2	59.5	84.2	39.0	31.4	113.8	49.7	46.2	106.4	88.1	199.8	167.2	109.3	0.0	162.5
		W	-50.1	-62.8	-62.6	-62.4	-43.4	-67.3	-39.2	-29.8	-47.1	-49.9	-18.6	-43.0	-44.3	-21.4	-28.3	14.2	1.8	-20.3	-61.9	0.0
			H	N	S	Q	R	G	D	E	T	A	P	C	K	M	V	I	L	F	Y	W
			B																			
2	A	H	0.0	-28.4	-33.8	-26.5	20.4	-44.3	30.5	51.7	8.2	-4.4	66.6	13.9	5.5	27.2	86.9	150.7	122.3	80.9		63.3
		N	39.7	0.0	-7.6	2.7	68.2	-22.2	82.3	112.0	51.1	33.6	132.8	59.2	47.5	77.8	161.2	250.2	210.6	152.8		128.2
		S	51.2	8.2	0.0	11.1	82.0	-15.8	97.2	129.4	63.5	44.6	151.8	72.2	59.5	92.3	182.5	278.9	236.0	173.5		146.9
		Q	36.1	-2.6	-10.0	0.0	63.9	-24.2	77.6	106.5	47.2	30.2	126.8	55.1	43.7	73.2	154.4	241.2	202.5	146.3		122.3
		R	-16.9	-40.6	-45.0	-39.0	0.0	-53.7	8.4	26.0	-10.1	-20.6	38.4	-5.3	-12.3	5.7	55.3	108.2	84.6	50.3		35.7
		G	79.6	28.5	18.8	31.9	116.2	0.0	134.3	172.5	94.2	71.7	199.1	104.6	89.5	128.5	235.6	350.1	299.1	224.9		193.3
		D	-23.4	-45.1	-49.3	-43.7	-7.7	-57.3	0.0	16.3	-17.1	-26.7	27.7	-12.7	-19.1	-2.5	43.3	92.1	70.3	38.7		25.2
		E	-34.1	-52.8	-56.4	-51.6	-20.7	-63.3	-14.0	0.0	-28.7	-37.0	9.8	-24.9	-30.4	-16.2	23.2	65.2	46.5	19.2		7.6
		T	-7.6	-33.8	-38.8	-32.1	11.3	-48.5	20.6	40.3	0.0	-11.6	54.0	5.3	-2.4	17.6	72.8	131.7	105.5	67.3		51.0
		A	4.6	-25.2	-30.8	-23.2	25.9	-41.8	36.4	58.7	13.1	0.0	74.2	19.2	10.4	33.0	95.5	162.1	132.4	89.2		70.8
		P	-40.0	-57.0	-60.3	-55.9	-27.7	-66.6	-21.7	-8.9	-35.1	-42.6	0.0	-31.6	-36.6	-23.6	12.2	50.5	33.4	8.6		-2.0
		C	-12.2	-37.2	-41.9	-35.5	5.6	-51.1	14.5	33.2	-5.1	-16.1	46.2	0.0	-7.4	11.7	64.0	120.0	95.0	58.8		43.3
		K	-5.3	-32.2	-37.3	-30.4	14.1	-47.2	23.6	43.8	2.5	-9.4	57.8	8.0	0.0	20.5	77.1	137.5	110.6	71.4		54.7
		M	-21.4	-43.8	-48.0	-42.3	-5.4	-56.2	2.5	19.3	-15.0	-24.8	30.9	-10.4	-17.0	0.0	46.9	97.0	74.7	42.2		28.4

		V	-46.5	-61.7	-64.6	-60.7	-35.6	-70.2	-30.2	-18.8	-42.1	-48.8	-10.9	-39.0	-43.5	-31.9	0.0	34.1	18.9	-3.2		-12.6
		I	-60.1	-71.4	-73.6	-70.7	-52.0	-77.8	-47.9	-39.5	-56.8	-61.8	-33.5	-54.5	-57.9	-49.2	-25.4	0.0	-11.3	-27.8		-34.8
		L	-55.0	-67.8	-70.2	-66.9	-45.8	-74.9	-41.3	-31.7	-51.3	-57.0	-25.0	-48.7	-52.5	-42.8	-15.9	12.8	0.0	-18.6		-26.5
		F	-44.7	-60.4	-63.4	-59.4	-33.5	-69.2	-27.9	-16.1	-40.2	-47.2	-7.9	-37.0	-41.7	-29.7	3.3	38.5	22.8	0.0		-9.7
		Y																				
		W	-38.8	-56.2	-59.5	-55.0	-26.3	-65.9	-20.1	-7.1	-33.8	-41.4	2.0	-30.2	-35.4	-22.1	14.5	53.5	36.1	10.8		0.0
			H	N	S	Q	R	G	D	E	T	A	P	C	K	M	V	I	L	F	Y	W
			**B**																			
3	**A**	H	0.0	-33.3	-35.2	-30.1	22.9	-48.1	32.3	69.4	10.7	-4.4	74.1	18.8	19.0	61.0	75.6	220.8	174.2	102.0	-29.3	122.0
		N	49.8	0.0	-2.9	4.7	84.1	-22.2	98.2	153.8	65.9	43.2	160.9	77.9	78.2	141.3	163.1	380.6	310.8	202.6	5.9	232.5
		S	54.3	3.0	0.0	7.8	89.6	-19.9	104.2	161.4	70.9	47.5	168.7	83.2	83.6	148.5	171.0	395.0	323.1	211.7	9.0	242.5
		Q	43.1	-4.5	-7.3	0.0	75.9	-25.7	89.3	142.4	58.5	36.8	149.2	69.9	70.2	130.5	151.4	359.1	292.4	189.1	1.1	217.6
		R	-18.6	-45.7	-47.3	-43.1	0.0	-57.7	7.7	37.8	-9.9	-22.2	41.7	-3.4	-3.2	31.0	42.9	161.0	123.1	64.4	-42.5	80.6
		G	92.5	28.5	24.8	34.5	136.6	0.0	154.8	226.1	113.2	84.1	235.3	128.7	129.1	210.1	238.2	517.6	428.0	288.9	36.1	327.3
		D	-24.4	-49.6	-51.0	-47.2	-7.1	-60.7	0.0	28.0	-16.3	-27.7	31.6	-10.3	-10.1	21.7	32.7	142.4	107.2	52.7	-46.6	67.7
		E	-41.0	-60.6	-61.7	-58.7	-27.4	-69.3	-21.9	0.0	-34.6	-43.6	2.8	-29.9	-29.8	-4.9	3.7	89.4	61.9	19.3	-58.3	31.0
		T	-9.7	-39.7	-41.5	-36.9	11.0	-53.1	19.5	53.0	0.0	-13.7	57.3	7.2	7.4	45.4	58.6	189.7	147.6	82.4	-36.2	100.4
		A	4.6	-30.2	-32.2	-26.9	28.5	-45.7	38.4	77.2	15.8	0.0	82.1	24.2	24.4	68.4	83.7	235.5	186.8	111.3	-26.1	132.1
		P	-42.6	-61.7	-62.8	-59.9	-29.4	-70.2	-24.0	-2.7	-36.4	-45.1	0.0	-31.8	-31.7	-7.5	0.9	84.2	57.5	16.0	-59.4	27.5
		C	-15.8	-43.8	-45.4	-41.2	3.5	-56.3	11.4	42.6	-6.8	-19.5	46.6	0.0	0.2	35.6	47.9	170.1	130.9	70.1	-40.5	86.9
		K	-15.9	-43.9	-45.5	-41.3	3.3	-56.3	11.2	42.4	-6.9	-19.6	46.4	-0.2	0.0	35.4	47.6	169.6	130.5	69.8	-40.6	86.6
		M	-37.9	-58.6	-59.8	-56.6	-23.7	-67.7	-17.8	5.2	-31.2	-40.6	8.1	-26.3	-26.1	0.0	9.1	99.2	70.3	25.4	-56.1	37.8
		V	-43.1	-62.0	-63.1	-60.2	-30.0	-70.4	-24.7	-3.6	-37.0	-45.6	-0.9	-32.4	-32.3	-8.3	0.0	82.6	56.1	15.0	-59.8	26.4
		I	-68.8	-79.2	-79.8	-78.2	-61.7	-83.8	-58.8	-47.2	-65.5	-70.2	-45.7	-63.0	-62.9	-49.8	-45.2	0.0	-14.5	-37.0	-78.0	-30.8
		L	-63.5	-75.7	-76.4	-74.5	-55.2	-81.1	-51.7	-38.2	-59.6	-65.1	-36.5	-56.7	-56.6	-41.3	-35.9	17.0	0.0	-26.3	-74.2	-19.1
		F	-50.5	-67.0	-67.9	-65.4	-39.2	-74.3	-34.5	-16.2	-45.2	-52.7	-13.8	-41.2	-41.1	-20.3	-13.0	58.8	35.7	0.0	-65.0	9.9
		Y	41.5	-5.5	-8.3	-1.1	73.9	-26.5	87.2	139.7	56.7	35.3	146.4	68.0	68.3	127.9	148.6	353.9	288.0	185.9	0.0	214.1
		W	-54.9	-69.9	-70.8	-68.5	-44.6	-76.6	-40.4	-23.7	-50.1	-56.9	-21.5	-46.5	-46.4	-27.4	-20.9	44.5	23.5	-9.0	-68.2	0.0
			H	N	S	Q	R	G	D	E	T	A	P	C	K	M	V	I	L	F	Y	W
			**B**																			
4	**A**	H	0.0	-15.7	-15.9	-14.6	14.1	-27.4	21.2	33.4	7.2	3.6	37.8	16.6	24.9	33.5	54.6	75.4	64.5	39.3	-9.1	71.2
		N	18.7	0.0	-0.2	1.4	35.4	-13.9	43.8	58.3	27.2	23.0	63.5	38.3	48.2	58.4	83.4	108.1	95.1	65.3	7.9	103.1
		S	18.8	0.2	0.0	1.5	35.6	-13.7	44.1	58.6	27.4	23.2	63.7	38.6	48.5	58.7	83.7	108.4	95.4	65.6	8.0	103.4
		Q	17.1	-1.4	-1.5	0.0	33.5	-15.0	41.9	56.2	25.5	21.3	61.3	36.5	46.2	56.3	80.9	105.3	92.5	63.1	6.4	100.4
		R	-12.3	-26.1	-26.2	-25.1	0.0	-36.4	6.2	17.0	-6.0	-9.2	20.7	2.2	9.5	17.0	35.5	53.7	44.2	22.1	-20.3	50.0
		G	37.8	16.1	15.9	17.7	57.2	0.0	67.0	83.8	47.7	42.8	89.8	60.6	72.1	83.9	112.9	141.6	126.6	92.0	25.2	135.8
		D	-17.5	-30.5	-30.6	-29.5	-5.9	-40.1	0.0	10.1	-11.5	-14.5	13.6	-3.8	3.1	10.1	27.5	44.7	35.7	15.0	-25.0	41.2
		E	-25.1	-36.8	-36.9	-36.0	-14.5	-45.6	-9.2	0.0	-19.6	-22.3	3.2	-12.6	-6.4	0.0	15.8	31.4	23.2	4.4	-31.9	28.3
		T	-6.7	-21.4	-21.5	-20.3	6.4	-32.3	13.0	24.4	0.0	-3.3	28.5	8.7	16.5	24.5	44.2	63.6	53.4	29.9	-15.2	59.6
		A	-3.5	-18.7	-18.8	-17.6	10.1	-30.0	17.0	28.8	3.5	0.0	32.9	12.5	20.5	28.8	49.1	69.2	58.7	34.4	-12.3	65.1
		P	-27.4	-38.8	-38.9	-38.0	-17.2	-47.3	-12.0	-3.1	-22.2	-24.8	0.0	-15.4	-9.3	-3.1	12.2	27.3	19.4	1.2	-34.0	24.3
		C	-14.2	-27.7	-27.8	-26.7	-2.1	-37.7	4.0	14.5	-8.0	-11.1	18.2	0.0	7.1	14.5	32.6	50.4	41.1	19.5	-22.0	46.8

		K	-19.9	-32.5	-32.6	-31.6	-8.7	-41.9	-3.0	6.8	-14.2	-17.0	10.3	-6.7	0.0	6.9	23.8	40.4	31.7	11.6	-27.2	37.0
		M	-25.1	-36.9	-37.0	-36.0	-14.5	-45.6	-9.2	0.0	-19.7	-22.4	3.2	-12.7	-6.4	0.0	15.8	31.4	23.2	4.4	-31.9	28.2
		V	-35.3	-45.5	-45.6	-44.7	-26.2	-53.0	-21.6	-13.7	-30.6	-33.0	-10.9	-24.6	-19.2	-13.6	0.0	13.5	6.4	-9.9	-41.2	10.7
		I	-43.0	-52.0	-52.0	-51.3	-35.0	-58.6	-30.9	-23.9	-38.9	-40.9	-21.5	-33.5	-28.8	-23.9	-11.9	0.0	-6.2	-20.6	-48.2	-2.4
		L	-39.2	-48.8	-48.8	-48.1	-30.6	-55.9	-26.3	-18.9	-34.8	-37.0	-16.2	-29.1	-24.0	-18.8	-6.0	6.6	0.0	-15.3	-44.7	4.1
		F	-28.2	-39.5	-39.6	-38.7	-18.1	-47.9	-13.0	-4.2	-23.0	-25.6	-1.1	-16.3	-10.4	-4.2	10.9	25.9	18.0	0.0	-34.8	22.8
		Y	10.0	-7.3	-7.4	-6.0	25.5	-20.1	33.4	46.8	18.0	14.0	51.6	28.3	37.4	46.9	70.1	93.0	80.9	53.3	0.0	88.3
		W	-41.6	-50.8	-50.8	-50.1	-33.3	-57.6	-29.2	-22.0	-37.4	-39.4	-19.5	-31.9	-27.0	-22.0	-9.7	2.5	-3.9	-18.6	-46.9	0.0
			H	N	S	Q	R	G	D	E	T	A	P	C	K	M	V	I	L	F	Y	W
			**B**																			

Supplementary Table 8*: Raw data Fig. 2b and Extended Data Fig. 3 – AA in water, 170 µm, 18 h, pH 7.4 – enrichments in bottom fraction Shown in the bottom left triangle.*

**repeat**		**species**	Ratio species against species (shown in heat maps), Eq. 2 $[A]_{j,k}/[B]_{j,k} - 1$ (%)																			
1	**A**	H	0.0	17.4	15.5	17.2	-7.1	24.6	-3.7	-7.2	0.0	4.1	-14.4	-2.2	3.8	-10.8	-12.4	-21.7	-19.4	-16.8	10.8	-17.3
		N	-14.8	0.0	-1.6	-0.2	-20.9	6.1	-18.0	-21.0	-14.8	-11.3	-27.1	-16.7	-11.6	-24.0	-25.4	-33.3	-31.4	-29.2	-5.7	-29.6
		S	-13.4	1.7	0.0	1.4	-19.6	7.8	-16.6	-19.6	-13.4	-9.9	-25.9	-15.3	-10.1	-22.7	-24.2	-32.2	-30.2	-28.0	-4.1	-28.4
		Q	-14.6	0.2	-1.4	0.0	-20.7	6.3	-17.8	-20.8	-14.6	-11.1	-26.9	-16.5	-11.4	-23.8	-25.3	-33.2	-31.2	-29.0	-5.5	-29.4
		R	7.6	26.4	24.3	26.1	0.0	34.0	3.7	-0.1	7.6	12.0	-7.8	5.2	11.7	-4.0	-5.8	-15.8	-13.3	-10.5	19.2	-11.0
		G	-19.7	-5.7	-7.3	-5.9	-25.4	0.0	-22.7	-25.5	-19.7	-16.4	-31.2	-21.5	-16.7	-28.4	-29.7	-37.2	-35.3	-33.2	-11.1	-33.6
		D	3.8	21.9	19.9	21.6	-3.5	29.3	0.0	-3.7	3.8	8.1	-11.1	1.5	7.7	-7.4	-9.1	-18.7	-16.4	-13.7	15.0	-14.2
		E	7.7	26.5	24.5	26.2	0.1	34.2	3.8	0.0	7.8	12.2	-7.7	5.4	11.8	-3.9	-5.7	-15.7	-13.2	-10.4	19.3	-10.9
		T	0.0	17.4	15.5	17.1	-7.1	24.5	-3.7	-7.2	0.0	4.1	-14.4	-2.2	3.8	-10.8	-12.5	-21.7	-19.4	-16.8	10.7	-17.3
		A	-3.9	12.8	11.0	12.5	-10.7	19.6	-7.5	-10.8	-3.9	0.0	-17.7	-6.1	-0.3	-14.3	-15.9	-24.8	-22.6	-20.1	6.4	-20.6
		P	16.8	37.1	34.9	36.8	8.5	45.4	12.5	8.4	16.8	21.6	0.0	14.2	21.2	4.2	2.2	-8.6	-5.9	-2.9	29.3	-3.4
		C	2.3	20.1	18.1	19.8	-5.0	27.4	-1.5	-5.1	2.3	6.5	-12.4	0.0	6.1	-8.7	-10.5	-20.0	-17.6	-14.9	13.3	-15.4
		K	-3.7	13.1	11.3	12.9	-10.5	20.0	-7.2	-10.6	-3.6	0.3	-17.5	-5.8	0.0	-14.0	-15.6	-24.6	-22.4	-19.9	6.7	-20.3
		M	12.1	31.6	29.4	31.3	4.1	39.6	8.0	4.0	12.1	16.7	-4.0	9.6	16.3	0.0	-1.9	-12.3	-9.7	-6.8	24.1	-7.3
		V	14.2	34.1	31.9	33.8	6.1	42.3	10.0	6.0	14.2	18.9	-2.2	11.7	18.5	1.9	0.0	-10.6	-8.0	-5.0	26.5	-5.5
		I	27.7	50.0	47.6	49.7	18.7	59.1	23.1	18.6	27.8	33.0	9.4	24.9	32.6	14.0	11.9	0.0	2.9	6.3	41.5	5.7
		L	24.1	45.7	43.4	45.4	15.3	54.6	19.6	15.2	24.1	29.2	6.3	21.4	28.8	10.7	8.7	-2.9	0.0	3.2	37.4	2.6
		F	20.2	41.2	38.9	40.8	11.7	49.8	15.8	11.6	20.2	25.2	3.0	17.6	24.8	7.3	5.3	-5.9	-3.1	0.0	33.2	-0.6
		Y	-9.7	6.0	4.3	5.8	-16.1	12.5	-13.0	-16.2	-9.7	-6.0	-22.7	-11.7	-6.3	-19.4	-20.9	-29.3	-27.2	-24.9	0.0	-25.3
		W	20.9	42.0	39.7	41.7	12.4	50.6	16.5	12.2	20.9	25.9	3.6	18.2	25.5	7.9	5.9	-5.4	-2.6	0.6	33.9	0.0
			H	N	S	Q	R	G	D	E	T	A	P	C	K	M	V	I	L	F	Y	W
			**B**																			
2	**A**	H	0.0	22.9	27.4	21.6	-8.2	45.9	-9.3	-15.1	-3.6	2.5	-19.9	-5.9	-2.1	-11.9	-22.1	-28.7	-26.5	-21.5		-15.7
		N	-18.7	0.0	3.7	-1.1	-25.4	18.7	-26.2	-30.9	-21.6	-16.6	-34.8	-23.4	-20.3	-28.3	-36.6	-42.0	-40.2	-36.1		-31.4
		S	-21.5	-3.5	0.0	-4.6	-28.0	14.5	-28.8	-33.4	-24.4	-19.5	-37.1	-26.1	-23.2	-30.9	-38.9	-44.1	-42.4	-38.4		-33.8
		Q	-17.7	1.1	4.8	0.0	-24.5	20.0	-25.4	-30.1	-20.7	-15.7	-34.1	-22.6	-19.4	-27.5	-35.9	-41.4	-39.6	-35.4		-30.6
		R	9.0	34.0	38.9	32.5	0.0	59.0	-1.2	-7.4	5.0	11.8	-12.7	2.6	6.7	-4.0	-15.1	-22.3	-19.9	-14.4		-8.1
		G	-31.5	-15.7	-12.7	-16.7	-37.1	0.0	-37.8	-41.8	-34.0	-29.7	-45.1	-35.5	-32.9	-39.6	-46.6	-51.1	-49.7	-46.2		-42.2
		D	10.3	35.6	40.5	34.1	1.2	60.9	0.0	-6.4	6.3	13.1	-11.6	3.8	8.0	-2.8	-14.1	-21.4	-19.0	-13.4		-7.0
		E	17.7	44.8	50.1	43.2	8.0	71.8	6.8	0.0	13.5	20.7	-5.6	10.8	15.3	3.7	-8.3	-16.0	-13.5	-7.6		-0.7
		T	3.8	27.6	32.2	26.2	-4.8	51.4	-5.9	-11.9	0.0	6.4	-16.8	-2.3	1.6	-8.6	-19.2	-26.0	-23.8	-18.5		-12.5
		A	-2.5	19.9	24.3	18.6	-10.5	42.3	-11.6	-17.2	-6.0	0.0	-21.8	-8.2	-4.5	-14.1	-24.0	-30.5	-28.4	-23.4		-17.8
		P	24.8	53.4	59.0	51.7	14.5	82.1	13.2	6.0	20.3	28.0	0.0	17.5	22.2	10.0	-2.8	-11.0	-8.3	-2.0		5.2
		C	6.2	30.6	35.4	29.2	-2.5	55.0	-3.7	-9.8	2.4	8.9	-14.9	0.0	4.0	-6.4	-17.3	-24.3	-22.0	-16.6		-10.4
		K	2.1	25.5	30.1	24.1	-6.3	49.0	-7.4	-13.3	-1.6	4.7	-18.2	-3.9	0.0	-10.0	-20.5	-27.2	-25.0	-19.8		-13.9
		M	13.5	39.5	44.6	38.0	4.1	65.6	2.9	-3.6	9.4	16.4	-9.0	6.8	11.1	0.0	-11.6	-19.1	-16.6	-10.9		-4.3

		V	28.4	57.8	63.6	56.1	17.8	87.3	16.4	9.0	23.7	31.7	2.9	20.9	25.7	13.1	0.0	-8.5	-5.7	0.8		8.3
		I	40.3	72.4	78.7	70.5	28.7	104.6	27.2	19.1	35.2	43.8	12.4	32.0	37.4	23.6	9.2	0.0	3.0	10.1		18.3
		L	36.1	67.4	73.5	65.5	24.9	98.6	23.5	15.6	31.2	39.6	9.1	28.1	33.3	19.9	6.0	-2.9	0.0	6.9		14.8
		F	27.4	56.6	62.3	54.9	16.9	85.9	15.5	8.2	22.8	30.6	2.1	19.9	24.7	12.2	-0.8	-9.2	-6.4	0.0		7.4
		Y																				
		W	18.6	45.8	51.1	44.2	8.8	73.0	7.6	0.7	14.3	21.6	-5.0	11.6	16.1	4.5	-7.6	-15.4	-12.9	-6.9		0.0
			H	N	S	Q	R	G	D	E	T	A	P	C	K	M	V	I	L	F	Y	W
			**B**																			
		H	0.0	22.6	22.6	20.7	-7.8	41.5	-11.3	-16.5	-4.3	0.3	-21.2	-4.3	-6.3	-17.3	-23.2	-31.4	-29.1	-22.8	13.3	-19.1
		N	-18.4	0.0	0.0	-1.5	-24.8	15.4	-27.6	-31.9	-21.9	-18.2	-35.8	-21.9	-23.6	-32.5	-37.4	-44.1	-42.2	-37.0	-7.6	-34.0
		S	-18.5	0.0	0.0	-1.6	-24.8	15.4	-27.7	-31.9	-22.0	-18.2	-35.8	-22.0	-23.6	-32.6	-37.4	-44.1	-42.2	-37.0	-7.6	-34.1
		Q	-17.2	1.6	1.6	0.0	-23.6	17.2	-26.5	-30.8	-20.7	-16.9	-34.8	-20.7	-22.4	-31.5	-36.4	-43.2	-41.3	-36.0	-6.1	-33.0
		R	8.4	32.9	33.0	30.9	0.0	53.4	-3.8	-9.5	3.8	8.7	-14.6	3.8	1.6	-10.3	-16.7	-25.6	-23.2	-16.3	22.9	-12.3
		G	-29.3	-13.4	-13.3	-14.7	-34.8	0.0	-37.3	-41.0	-32.4	-29.1	-44.3	-32.4	-33.8	-41.6	-45.7	-51.5	-49.9	-45.4	-19.9	-42.9
		D	12.7	38.2	38.2	36.1	4.0	59.5	0.0	-5.9	7.9	13.0	-11.2	7.9	5.6	-6.8	-13.4	-22.7	-20.1	-13.0	27.8	-8.8
		E	19.8	46.8	46.9	44.6	10.4	69.5	6.2	0.0	14.6	20.1	-5.7	14.6	12.2	-1.0	-8.1	-17.9	-15.1	-7.5	35.7	-3.2
		T	4.5	28.1	28.1	26.1	-3.7	47.8	-7.3	-12.8	0.0	4.8	-17.7	0.0	-2.1	-13.6	-19.8	-28.4	-26.0	-19.3	18.4	-15.5
		A	-0.3	22.3	22.3	20.4	-8.0	41.1	-11.5	-16.7	-4.5	0.0	-21.4	-4.6	-6.5	-17.5	-23.4	-31.6	-29.3	-23.0	13.0	-19.4
3	**A**	P	27.0	55.7	55.7	53.3	17.1	79.7	12.6	6.0	21.5	27.3	0.0	21.5	19.0	5.0	-2.5	-12.9	-10.0	-2.0	43.9	2.7
		C	4.5	28.1	28.2	26.2	-3.6	47.9	-7.3	-12.7	0.0	4.8	-17.7	0.0	-2.1	-13.6	-19.8	-28.3	-25.9	-19.3	18.4	-15.5
		K	6.7	30.8	30.9	28.8	-1.6	51.0	-5.3	-10.9	2.1	7.0	-16.0	2.1	0.0	-11.8	-18.1	-26.8	-24.4	-17.6	20.9	-13.7
		M	20.9	48.2	48.3	46.0	11.5	71.1	7.3	1.0	15.7	21.2	-4.8	15.7	13.3	0.0	-7.2	-17.1	-14.3	-6.6	37.0	-2.2
		V	30.2	59.7	59.7	57.2	20.1	84.3	15.5	8.8	24.7	30.6	2.6	24.6	22.1	7.7	0.0	-10.7	-7.7	0.6	47.6	5.3
		I	45.8	78.8	78.8	76.0	34.5	106.3	29.4	21.8	39.6	46.2	14.8	39.5	36.7	20.6	12.0	0.0	3.3	12.6	65.3	17.9
		L	41.1	73.0	73.1	70.4	30.1	99.7	25.2	17.8	35.1	41.5	11.1	35.0	32.3	16.7	8.3	-3.2	0.0	9.0	59.9	14.1
		F	29.5	58.8	58.8	56.4	19.4	83.3	14.9	8.2	24.0	29.9	2.0	23.9	21.4	7.1	-0.6	-11.2	-8.2	0.0	46.8	4.7
		Y	-11.8	8.2	8.2	6.5	-18.6	24.9	-21.7	-26.3	-15.5	-11.5	-30.5	-15.6	-17.3	-27.0	-32.3	-39.5	-37.5	-31.9	0.0	-28.7
		W	23.7	51.6	51.7	49.3	14.0	75.0	9.7	3.3	18.4	24.0	-2.6	18.3	15.9	2.3	-5.0	-15.2	-12.4	-4.5	40.2	0.0
			H	N	S	Q	R	G	D	E	T	A	P	C	K	M	V	I	L	F	Y	W
			**B**																			
		H	0.0	13.7	10.9	12.8	-6.9	20.2	-5.9	-9.2	-0.6	2.3	-11.9	-2.1	-2.5	-9.4	-13.0	-21.4	-19.4	-15.8	5.2	-16.6
		N	-12.0	0.0	-2.4	-0.8	-18.1	5.8	-17.2	-20.1	-12.6	-10.0	-22.6	-13.9	-14.2	-20.3	-23.5	-30.9	-29.1	-25.9	-7.4	-26.7
		S	-9.8	2.5	0.0	1.7	-16.1	8.4	-15.1	-18.1	-10.4	-7.8	-20.6	-11.8	-12.1	-18.3	-21.6	-29.2	-27.3	-24.0	-5.1	-24.8
		Q	-11.4	0.8	-1.7	0.0	-17.5	6.6	-16.6	-19.5	-11.9	-9.3	-21.9	-13.2	-13.5	-19.7	-22.9	-30.4	-28.5	-25.3	-6.7	-26.1
		R	7.4	22.2	19.2	21.2	0.0	29.2	1.1	-2.4	6.8	9.9	-5.4	5.2	4.8	-2.7	-6.6	-15.6	-13.4	-9.5	13.1	-10.4
4	**A**	G	-16.8	-5.4	-7.8	-6.2	-22.6	0.0	-21.7	-24.5	-17.3	-14.9	-26.8	-18.6	-18.9	-24.7	-27.7	-34.7	-32.9	-29.9	-12.5	-30.6
		D	6.3	20.8	17.9	19.9	-1.1	27.8	0.0	-3.5	5.6	8.7	-6.4	4.0	3.6	-3.7	-7.6	-16.5	-14.3	-10.5	11.8	-11.4
		E	10.1	25.2	22.1	24.2	2.5	32.4	3.6	0.0	9.5	12.6	-3.0	7.8	7.4	-0.2	-4.2	-13.5	-11.2	-7.2	15.9	-8.2
		T	0.6	14.4	11.6	13.5	-6.4	21.0	-5.3	-8.7	0.0	2.9	-11.4	-1.5	-1.9	-8.9	-12.5	-21.0	-18.9	-15.3	5.9	-16.1
		A	-2.2	11.2	8.5	10.3	-9.0	17.6	-8.0	-11.2	-2.8	0.0	-13.9	-4.3	-4.6	-11.4	-15.0	-23.2	-21.2	-17.6	2.9	-18.5
		P	13.6	29.1	26.0	28.1	5.7	36.6	6.9	3.1	12.9	16.1	0.0	11.1	10.8	2.9	-1.2	-10.8	-8.4	-4.3	19.5	-5.3
		C	2.2	16.2	13.3	15.3	-4.9	22.9	-3.8	-7.2	1.6	4.5	-10.0	0.0	-0.3	-7.4	-11.1	-19.7	-17.6	-13.9	7.5	-14.8

		K	2.5	16.6	13.7	15.7	-4.6	23.3	-3.5	-6.9	1.9	4.8	-9.7	0.3	0.0	-7.1	-10.8	-19.5	-17.3	-13.6	7.9	-14.5
		M	10.4	25.5	22.4	24.5	2.7	32.7	3.9	0.2	9.7	12.9	-2.8	8.0	7.7	0.0	-4.0	-13.3	-11.0	-7.0	16.2	-7.9
		V	15.0	30.7	27.5	29.7	7.0	38.3	8.2	4.4	14.3	17.6	1.2	12.5	12.2	4.2	0.0	-9.7	-7.3	-3.1	21.0	-4.1
		I	27.3	44.7	41.2	43.6	18.5	53.0	19.8	15.6	26.5	30.2	12.1	24.6	24.2	15.3	10.7	0.0	2.6	7.2	34.0	6.1
		L	24.0	41.0	37.6	39.9	15.4	49.1	16.7	12.6	23.3	26.8	9.2	21.4	21.0	12.4	7.9	-2.6	0.0	4.5	30.5	3.4
		F	18.7	35.0	31.7	33.9	10.5	42.7	11.7	7.8	18.0	21.4	4.5	16.2	15.8	7.5	3.2	-6.7	-4.3	0.0	24.9	-1.0
		Y	-5.0	8.0	5.4	7.2	-11.6	14.3	-10.6	-13.7	-5.5	-2.8	-16.3	-7.0	-7.3	-13.9	-17.4	-25.3	-23.4	-20.0	0.0	-20.8
		W	19.9	36.3	33.0	35.3	11.6	44.2	12.9	8.9	19.2	22.6	5.6	17.4	17.0	8.6	4.3	-5.8	-3.3	1.0	26.2	0.0
			H	N	S	Q	R	G	D	E	T	A	P	C	K	M	V	I	L	F	Y	W
			**B**																			

***Supplementary Table 9**: Raw data Fig. 3a and Extended Data Fig. 5a – nucleobases in water, 170µm, 18h*

repeat k	species	measured values (µAU*min)				concentration (µM) $[A]_{j,k,HPLC}$ calibration acc. to Supplementary Table 3				c_0 (µM)	Normalization (Eq. 1) concentration (x c_0)				Ratio species vs mean (Eq. 4) $[A]_{j,k}/\bar{c}_{j,k} - 1$ (%)		Ratio species against species (shown in heat maps), Eq. 2 $[A]_{j,k}/[B]_{j,k} - 1$ (%)											
		top	II	III	bot	top	II	III	bot		top	II	III	bot	top	bot	top part (blue shade)						bot part (red shade)					
1	C	663.99	928.45	1173.02	2148.73	14.6	20.41	25.79	47.24	27.01	0.54	0.76	0.95	1.75	-22.82	18.78	C	0	-32.92	-39	-25.79	-0.04	C	0	37.07	45.68	25.74	0.17
	A	2384.36	2718.93	2957.58	3775.75	20.99	23.93	26.03	33.23	26.04	0.81	0.92	1	1.28	15.07	-13.35	A	49.09	0	-9.05	10.63	49.03	A	-27.05	0	6.28	-8.27	-26.92
	G	669.47	707.5	738.41	907.17	31.02	32.78	34.21	42.03	35.01	0.89	0.94	0.98	1.2	26.52	-18.47	G	63.93	9.95	0	21.65	63.86	G	-31.36	-5.91	0	-13.69	-31.24
	U	1385.64	1682.96	1895.22	2646.42	19.19	23.31	26.25	36.66	26.35	0.73	0.88	1	1.39	4.01	-5.54	U	34.75	-9.61	-17.8	0	34.7	U	-20.47	9.01	15.86	0	-20.34
	T	966.27	1361.15	1700.63	3120.4	15.18	21.38	26.71	49.01	28.07	0.54	0.76	0.95	1.75	-22.79	18.58	T	0.04	-32.9	-38.97	-25.76	0	T	-0.17	36.84	45.44	25.53	0
	mean concentration per fraction $\bar{c}_{j,k}$										0.7	0.85	0.98	1.47				C	A	G	U	T		C	A	G	U	T
2	C	522.74	745.92	881.15	2074.36	11.49	16.4	19.37	45.61	23.22	0.49	0.71	0.83	1.96	-24.12	17.28	C	0	-37.29	-36.18	-29.29	-1.28	C	0	37.79	34.03	24.83	0.95
	A	2105.14	2464.35	2296.27	3801.61	18.53	21.69	20.21	33.46	23.47	0.79	0.92	0.86	1.43	21.01	-14.89	A	59.47	0	1.77	12.75	57.44	A	-27.43	0	-2.73	-9.41	-26.74
	G	517.24	607.52	565.33	977.31	23.96	28.15	26.19	45.28	30.89	0.78	0.91	0.85	1.47	18.91	-12.5	G	56.7	-1.74	0	10.79	54.7	G	-25.39	2.81	0	-6.86	-24.68
	U	1185.18	1466.37	1456.3	2663.82	16.42	20.31	20.17	36.9	23.45	0.7	0.87	0.86	1.57	7.33	-6.05	U	41.43	-11.31	-9.74	0	39.63	U	-19.89	10.38	7.36	0	-19.13
	T	776.13	1108.52	1295.27	3011.8	12.19	17.41	20.34	47.3	24.31	0.5	0.72	0.84	1.95	-23.13	16.17	T	1.29	-36.48	-35.36	-28.38	0	T	-0.95	36.49	32.76	23.65	0
	mean concentration per fraction $\bar{c}_{j,k}$										0.65	0.82	0.85	1.67				C	A	G	U	T		C	A	G	U	T
3	C	682.94	996.45	1284.95	1480.68	15.01	21.91	28.25	32.55	24.43	0.61	0.9	1.16	1.33	-14.3	8.19	C	0	-24.05	-21.36	-18.89	-1.29	C	0	15.83	15.3	12.3	-0.06
	A	2221.35	2653.78	2948.17	3157.98	19.55	23.36	25.95	27.79	24.16	0.81	0.97	1.07	1.15	12.84	-6.6	A	31.66	0	3.54	6.79	29.96	A	-13.67	0	-0.46	-3.05	-13.72
	G	490.41	604.4	690.17	725.23	22.72	28	31.98	33.6	29.07	0.78	0.96	1.1	1.16	8.98	-6.17	G	27.16	-3.42	0	3.14	25.52	G	-13.27	0.46	0	-2.6	-13.32
	U	1323.38	1667.43	1923.32	2072.46	18.33	23.1	26.64	28.71	24.19	0.76	0.95	1.1	1.19	5.66	-3.66	U	23.29	-6.36	-3.04	0	21.7	U	-10.95	3.15	2.67	0	-11
	T	1007.24	1445.85	1861.29	2156.92	15.82	22.71	29.23	33.88	25.41	0.62	0.89	1.15	1.33	-13.18	8.25	T	1.31	-23.06	-20.33	-17.83	0	T	0.06	15.9	15.37	12.36	0
	mean concentration per fraction $\bar{c}_{j,k}$										0.72	0.93	1.12	1.23				C	A	G	U	T		C	A	G	U	T

***Supplementary Table 10**: Raw data Fig. 3b – adenosine and AMPs in water, 170µm, 18h*
Due to technical problems, for the third replicate the temperature gradient was not applied for the entire duration of the experiment, which is why we do not take it into account for the calculation of the heat flow-driven enrichment.

repeat k	species	measured values (µAU*min)				concentration (µM) $[A]_{j,k,HPLC}$ calibration acc. to Supplementary Table 3				c_0 (µM)
		top	II	III	bot	top	II	III	bot	
1	Ado	3745.69	4798.81	4859.08	5498.03	26.51	33.96	34.39	38.91	33.44
	5'	2233.92	3330.98	3991.21	6700.22	21.39	31.89	38.21	64.15	38.91
	2'	534.36	846.23	1037.77	1814.44	15.63	24.76	30.36	53.08	30.96
	3'	1247.72	1917.85	2285.14	3838.79	11.28	17.35	20.67	34.72	21
	2',3'	1800.09	2826.62	3660.37	7304.36	17.18	26.98	34.94	69.73	37.21
	3'.5'	2531.16	3981.59	5167.52	10411.38	18.39	28.93	37.55	75.65	40.13
	mean concentration per fraction $\bar{c}_{j,k}$									
2	Ado	3862.07	3931.11	4175.67	5972.89	27.33	27.82	29.55	42.27	31.74
	5'	2392.86	2568.85	2834.82	7140.5	22.91	24.6	27.14	68.37	35.75
	2'	583.69	636.98	712.66	1921.77	17.08	18.64	20.85	56.22	28.2
	3'	1372.67	1484.13	1643.05	4111.9	12.41	13.42	14.86	37.19	19.47
	2',3'	1999.27	2169.7	2455.97	7709.24	19.09	20.71	23.45	73.59	34.21
	3'.5'	2807.7	3048.19	3451.08	10975.17	20.4	22.15	25.08	79.75	36.84
	mean concentration per fraction $\bar{c}_{j,k}$									

repeat k	species	Normalization (Eq. 1) concentration (x c_0)				Ratio species vs mean (Eq. 4) $[A]_{j,k}/\bar{c}_{j,k}-1$ (%)	
		top	II	III	bot	top	bot
1	Ado	0.79	1.02	1.03	1.16	33.99	-26.22
	5'	0.55	0.82	0.98	1.65	-7.09	4.54
	2'	0.5	0.8	0.98	1.71	-14.65	8.73
	3'	0.54	0.83	0.98	1.65	-9.19	4.82
	2',3'	0.46	0.73	0.94	1.87	-21.94	18.83
	3'.5'	0.46	0.72	0.94	1.89	-22.53	19.54
	mean concentration per fraction $\bar{c}_{j,k}$	0.55	0.82	0.98	1.66		
2	Ado	0.86	0.88	0.93	1.33	26.66	-26.65
	5'	0.64	0.69	0.76	1.91	-5.74	5.33
	2'	0.61	0.66	0.74	1.99	-10.91	9.84
	3'	0.64	0.69	0.76	1.91	-6.21	5.21
	2',3'	0.56	0.61	0.69	2.15	-17.93	18.51
	3'.5'	0.55	0.6	0.68	2.16	-18.55	19.24
	mean concentration per fraction $\bar{c}_{j,k}$	0.64	0.69	0.76	1.91		

Ratio species against species (shown in heat maps), Eq. 2 $[A]_{j,k}/[B]_{j,k}-1$ (%)

repeat k	species	top part (blue shade)						
1	Ado	Ado	0	44.21	56.97	47.54	71.64	72.96
	5'	5'	-30.66	0	8.85	2.31	19.02	19.94
	2'	2'	-36.3	-8.13	0	-6.01	9.34	10.18
	3'	3'	-32.22	-2.26	6.39	0	16.34	17.23
	2',3'	2',3'	-41.74	-15.98	-8.55	-14.04	0	0.77
	3'.5'	3'.5'	-42.18	-16.62	-9.24	-14.7	-0.76	0
	mean concentration per fraction $\bar{c}_{j,k}$		Ado	5'	2'	3'	2',3'	3'.5'
2	Ado	Ado	0	34.37	42.17	35.05	54.33	55.5
	5'	5'	-25.58	0	5.8	0.5	14.86	15.72
	2'	2'	-29.66	-5.49	0	-5.01	8.56	9.37
	3'	3'	-25.95	-0.5	5.27	0	14.28	15.14
	2',3'	2',3'	-35.2	-12.93	-7.88	-12.5	0	0.75
	3'.5'	3'.5'	-35.69	-13.59	-8.57	-13.15	-0.75	0
	mean concentration per fraction $\bar{c}_{j,k}$		Ado	5'	2'	3'	2',3'	3'.5'

repeat k	species	bot part (red shade)						
1	Ado	Ado	0	-29.43	-32.14	-29.61	-37.91	-38.28
	5'	5'	41.7	0	-3.85	-0.26	-12.02	-12.54
	2'	2'	47.37	4	0	3.73	-8.5	-9.04
	3'	3'	42.07	0.26	-3.6	0	-11.79	-12.32
	2',3'	2',3'	61.06	13.67	9.29	13.37	0	-0.59
	3'.5'	3'.5'	62.02	14.34	9.94	14.05	0.6	0
	mean concentration per fraction $\bar{c}_{j,k}$		Ado	5'	2'	3'	2',3'	3'.5'
2	Ado	Ado	0	-30.36	-33.22	-30.28	-38.1	-38.48
	5'	5'	43.6	0	-4.1	0.12	-11.12	-11.66
	2'	2'	49.74	4.28	0	4.4	-7.31	-7.88
	3'	3'	43.43	-0.12	-4.22	0	-11.22	-11.76
	2',3'	2',3'	61.55	12.51	7.89	12.64	0	-0.61
	3'.5'	3'.5'	62.55	13.2	8.55	13.33	0.61	0
	mean concentration per fraction $\bar{c}_{j,k}$		Ado	5'	2'	3'	2',3'	3'.5'

Supplementary Table 11*: Raw data Fig. 3c – Ribonucleosides in water, 170µm, 18h*

repeat k	species	measured values (µAU*s)				concentration (µM) $[A]_{j,k,HPLC}$ calibration acc. to Supplementary Table 3				c_0 (µM)	Normalization (Eq. 1) concentration (x c_0)				Ratio species vs mean (Eq. 4) $[A]_{j,k}/\bar{c}_{j,k} - 1$ (%)		Ratio species against species (shown in heat maps), Eq. 2 $[A]_{j,k}/[B]_{j,k} - 1$ (%)									
		top	II	III	bot	top	II	III	bot		top	II	III	bot	top	bot	top part (blue shade)					bot part (red shade)				
1	C	42856	57126	87630	160202	12.09	16.11	24.72	45.19	24.53	0.49	0.66	1.01	1.84	-16.9	11.65	C	0	-16.51	-21.79	-25.22	C	0	12.04	17.68	19
	U	79997	97939	141268	222829	14.72	18.02	25.99	41	24.93	0.59	0.72	1.04	1.64	-0.47	-0.35	U	19.78	0	-6.32	-10.43	U	-10.75	0	5.03	6.21
	A	131148	156721	218736	325815	15.47	18.48	25.8	38.43	24.55	0.63	0.75	1.05	1.57	6.25	-5.12	A	27.87	6.75	0	-4.39	A	-15.02	-4.79	0	1.12
	G	115509	133861	180304	271329	14.53	16.84	22.69	34.14	22.05	0.66	0.76	1.03	1.55	11.12	-6.18	G	33.73	11.65	4.59	0	G	-15.97	-5.85	-1.11	0
	mean concentration per fraction $\bar{c}_{j,k}$										0.59	0.72	1.03	1.65				C	U	A	G		C	U	A	G
2	C	57422	74318	97539	110893	16.2	20.96	27.51	31.28	23.99	0.68	0.87	1.15	1.3	-9.75	5.01	C	0	-10.06	-13.77	-13.83	C	0	5.32	7.94	7.15
	U	100453	118881	150246	165666	18.48	21.87	27.64	30.48	24.62	0.75	0.89	1.12	1.24	0.34	-0.3	U	11.18	0	-4.13	-4.2	U	-5.05	0	2.48	1.73
	A	161163	184416	229067	248644	19.01	21.75	27.02	29.33	24.28	0.78	0.9	1.11	1.21	4.66	-2.72	A	15.97	4.3	0	-0.08	A	-7.36	-2.42	0	-0.73
	G	138500	158232	195151	215097	17.43	19.91	24.55	27.06	22.24	0.78	0.9	1.1	1.22	4.74	-2	G	16.05	4.38	0.08	0	G	-6.67	-1.7	0.74	0
	mean concentration per fraction $\bar{c}_{j,k}$										0.75	0.89	1.12	1.24				C	U	A	G		C	U	A	G
3	C	61659	71020	96802	102497	17.39	20.03	27.3	28.91	23.41	0.74	0.86	1.17	1.23	-7.93	4.22	C	0	-8.23	-11.54	-11.04	C	0	4.26	6.3	6.61
	U	106884	115185	149675	156395	19.66	21.19	27.54	28.77	24.29	0.81	0.87	1.13	1.18	0.33	-0.04	U	8.96	0	-3.62	-3.07	U	-4.09	0	1.95	2.25
	A	171403	179162	228648	237098	20.22	21.13	26.97	27.96	24.07	0.84	0.88	1.12	1.16	4.09	-1.95	A	13.05	3.75	0	0.56	A	-5.93	-1.92	0	0.29
	G	146640	155741	196546	203394	18.45	19.6	24.73	25.59	22.09	0.84	0.89	1.12	1.16	3.51	-2.24	G	12.42	3.17	-0.56	0	G	-6.2	-2.2	-0.29	0
	mean concentration per fraction $\bar{c}_{j,k}$										0.81	0.87	1.13	1.18				C	U	A	G		C	U	A	G

***Supplementary Table 12**: Raw data Fig. 3d – 5’-Ribonucleotides in water, 170µm, 18h*

repeat k	species	measured values (µAU*min)				concentration (µM) $[A]_{j,k,HPLC}$ calibration acc. to Supplementary Table 3				c_0 (µM)	Normalization (Eq. 1) concentration (x c_0)				Ratio species vs mean (Eq. 4) $[A]_{j,k}/\bar{c}_{j,k}-1$ (%)		Ratio species against species (shown in heat maps), Eq. 2 $[A]_{j,k}/[B]_{j,k}-1$ (%)									
		top	II	III	bot	top	II	III	bot		top	II	III	bot	top	bot	top part (blue shade)					bot part (red shade)				
1	C	311.46	448.32	1079.66	2466.9	6.74	9.7	23.36	53.37	23.29	0.29	0.42	1	2.29	-7.38	2.99	C	0	-13.42	-11.9	-2.81	C	0	6.37	3.47	2.31
	A	846.94	1193.24	2638.36	5460.1	8.11	11.42	25.26	52.28	24.27	0.33	0.47	1.04	2.15	6.97	-3.18	A	15.5	0	1.76	12.25	A	-5.99	0	-2.73	-3.81
	U	372.11	510.79	1140.31	2509.58	7.33	10.06	22.45	49.41	22.31	0.33	0.45	1.01	2.21	5.12	-0.46	U	13.5	-1.73	0	10.31	U	-3.35	2.81	0	-1.12
	G	482.99	721.47	1651.98	3633.94	5.74	8.58	19.65	43.22	19.3	0.3	0.44	1.02	2.24	-4.71	0.66	G	2.89	-10.92	-9.35	0	G	-2.26	3.96	1.13	0
	mean concentration per fraction $\bar{c}_{j,k}$										0.31	0.45	1.02	2.22				C	A	U	G		C	A	U	G
2	C	383.2	538.23	1426.77	2146.23	8.29	11.64	30.87	46.43	24.31	0.34	0.48	1.27	1.91	-7.33	3.23	C	0	-11.96	-11.37	-4.96	C	0	5.92	4.1	3.07
	A	1020.27	1397.84	3367.33	4749.7	9.77	13.38	32.24	45.48	25.22	0.39	0.53	1.28	1.8	5.26	-2.54	A	13.59	0	0.67	7.96	A	-5.59	0	-1.72	-2.69
	U	458.55	617.08	1504.48	2186.51	9.03	12.15	29.62	43.05	23.46	0.38	0.52	1.26	1.83	4.56	-0.84	U	12.83	-0.67	0	7.24	U	-3.94	1.75	0	-1
	G	601.16	841.33	2153.96	3104.92	7.15	10.01	25.62	36.93	19.93	0.36	0.5	1.29	1.85	-2.5	0.16	G	5.21	-7.37	-6.75	0	G	-2.97	2.77	1.01	0
	mean concentration per fraction $\bar{c}_{j,k}$										0.37	0.51	1.27	1.85				C	A	U	G		C	A	U	G
3	C	239.91	550.05	820.73	2802.29	5.19	11.9	17.76	60.63	23.87	0.22	0.5	0.74	2.54	-7.07	3.14	C	0	-12.5	-12.48	-1.85	C	0	6.19	4.99	1.61
	A	649.78	1464.37	2089.86	6253.99	6.22	14.02	20.01	59.88	25.03	0.25	0.56	0.8	2.39	6.21	-2.87	A	14.29	0	0.02	12.18	A	-5.83	0	-1.13	-4.31
	U	296.19	646.46	941.68	2883.92	5.83	12.73	18.54	56.77	23.47	0.25	0.54	0.79	2.42	6.18	-1.77	U	14.26	-0.02	0	12.15	U	-4.75	1.14	0	-3.22
	G	375.24	889.94	1275.76	4233.75	4.46	10.58	15.17	50.35	20.14	0.22	0.53	0.75	2.5	-5.32	1.5	G	1.88	-10.85	-10.83	0	G	-1.59	4.5	3.33	0
	mean concentration per fraction $\bar{c}_{j,k}$										0.23	0.53	0.77	2.46				C	A	U	G		C	A	U	G

***Supplementary Table 13**: Raw data Fig. 3e – Deoxyribonucleotides in water, 170µm, 18h*

repeat k	species	measured values (µAU*min)				concentration (µM) $[A]_{j,k,HPLC}$ calibration acc. to Supplementary Table 3				c_0 (µM)	Normalization (Eq. 1) concentration (x c_0)				Ratio species vs mean (Eq. 4) $[A]_{j,k}/\bar{c}_{j,k}-1$ (%)		Ratio species against species (shown in heat maps), Eq. 2 $[A]_{j,k}/[B]_{j,k}-1$ (%)									
		top	II	III	bot	top	II	III	bot		top	II	III	bot	top	bot	top part (blue shade)					bot part (red shade)				
1	C	397.27	584.32	813.84	4692.53	7.52	11.05	15.4	88.77	30.69	0.24	0.36	0.5	2.89	-4.26	1.89	C	0	-11.39	-7.49	3.26	C	0	5	3.81	-0.99
	A	479.93	706.15	975.11	4783.78	8.46	12.45	17.2	84.37	30.62	0.28	0.41	0.56	2.76	8.05	-2.96	A	12.86	0	4.4	16.54	A	-4.76	0	-1.14	-5.71
	G	823.52	1246.19	1703.29	8668.96	7.84	11.86	16.22	82.53	29.61	0.26	0.4	0.55	2.79	3.49	-1.84	G	8.1	-4.22	0	11.62	G	-3.67	1.15	0	-4.62
	T	529.37	779.82	1096.56	6521.43	7.09	10.45	14.69	87.38	29.9	0.24	0.35	0.49	2.92	-7.28	2.91	T	-3.16	-14.19	-10.41	0	T	1	6.05	4.85	0
	mean concentration per fraction $\bar{c}_{j,k}$										0.26	0.38	0.53	2.84				C	A	G	T		C	A	G	T
2	C	185.64	508.5	1537.77	4445.32	3.51	9.62	29.09	84.1	31.58	0.11	0.3	0.92	2.66	-12.09	2.37	C	0	-23.34	-16.33	-4.83	C	0	6.1	3.96	-0.31
	A	256.15	640.58	1734.58	4431.7	4.52	11.3	30.59	78.16	31.14	0.15	0.36	0.98	2.51	14.67	-3.52	A	30.44	0	9.14	24.14	A	-5.75	0	-2.02	-6.04
	G	415.02	1077.38	2998.93	7998.59	3.95	10.26	28.55	76.15	29.73	0.13	0.35	0.96	2.56	5.06	-1.53	G	19.52	-8.38	0	13.74	G	-3.81	2.06	0	-4.1
	T	266.83	698.93	2068.55	6099.3	3.58	9.36	27.72	81.72	30.59	0.12	0.31	0.91	2.67	-7.63	2.68	T	5.08	-19.45	-12.08	0	T	0.31	6.43	4.28	0
	mean concentration per fraction $\bar{c}_{j,k}$										0.13	0.33	0.94	2.6				C	A	G	T		C	A	G	T
3	C	382.19	571.29	805.98	4714.74	7.23	10.81	15.25	89.19	30.62	0.24	0.35	0.5	2.91	-5.61	1.98	C	0	-12.51	-9.99	1.63	C	0	4.65	4.24	-0.74
	A	467.95	689.43	951.67	4825.79	8.25	12.16	16.78	85.11	30.58	0.27	0.4	0.55	2.78	7.89	-2.55	A	14.31	0	2.89	16.17	A	-4.44	0	-0.39	-5.15
	G	816.07	1237.11	1696.87	8693.16	7.77	11.78	16.15	82.76	29.62	0.26	0.4	0.55	2.79	4.86	-2.17	G	11.1	-2.81	0	12.91	G	-4.07	0.4	0	-4.78
	T	520.02	776.52	1087.61	6568.59	6.97	10.4	14.57	88.01	29.99	0.23	0.35	0.49	2.93	-7.13	2.74	T	-1.61	-13.92	-11.43	0	T	0.75	5.43	5.02	0
	mean concentration per fraction $\bar{c}_{j,k}$										0.25	0.37	0.52	2.86				C	A	G	T		C	A	G	T
4	C	166.63	492.33	1542.28	4503.33	3.15	9.31	29.18	85.19	31.71	0.1	0.29	0.92	2.69	-8.55	2.28	C	0	-19.65	-14.46	4.15	C	0	5.76	4.15	-0.51
	A	216.14	607.39	1726.22	4437.74	3.81	10.71	30.44	78.26	30.81	0.12	0.35	0.99	2.54	13.82	-3.29	A	24.46	0	6.46	29.62	A	-5.45	0	-1.52	-5.93
	G	358.92	1036.52	2990.87	7967.02	3.42	9.87	28.47	75.85	29.4	0.12	0.34	0.97	2.58	6.91	-1.79	G	16.91	-6.07	0	21.75	G	-3.98	1.55	0	-4.47
	T	215.96	662.45	2061.8	6109.74	2.89	8.88	27.63	81.86	30.31	0.1	0.29	0.91	2.7	-12.19	2.8	T	-3.98	-22.85	-17.87	0	T	0.51	6.3	4.68	0
	mean concentration per fraction $\bar{c}_{j,k}$										0.11	0.32	0.95	2.63				C	A	G	T		C	A	G	T

***Supplementary Table 14**: Raw data Fig. 4b-c – Experimental network, raw data*
After recovery in fractions, we pre-column derivatized the samples and recorded the chromatograms in the fluorescence channel.
The sample designation corresponds to the scheme '#id#_#chamber#_#fraction# of #totalFractions#', where #id# is the sample ID, #chamber# is the chamber number as shown in Figure 4a, #fraction# is the fraction number inside each chamber as shown in Figure 4a, and #totalFractions# is the total number of all fractions in the chamber.

dT	repeat	part	[N] (counts*min)	[S] (counts*min)	[G] (counts*min)	[T] (counts*min)	[A] (counts*min)	[P] (counts*min)	[C] (counts*min)	[V] (counts*min)	[I] (counts*min)	[L] (counts*min)	[F] (counts*min)
16.2	1	426_1_1 of 4	386057	390545	447855	296107	268060	36270	37194	165200	86755	102568	118872
16.2	1	426_1_2 of 4	420592	425577	439650	410337	365227	72446	48966	348217	248051	272722	268441
16.2	1	426_1_3 of 4	543682	551062	564611	580260	504453	125414	64069	626755	560455	578038	479063
16.2	1	426_1_4 of 4	489651	502696	501468	557997	485488	130563	59605	662280	638148	644313	509763
16.2	1	426_2_1 of 4	424517	426962	493041	322202	297955	36680	40458	198093	79708	98558	125302
16.2	1	426_2_2 of 4	409130	410934	389395	401430	360047	60568	45493	284374	141244	164799	198089
16.2	1	426_2_3 of 4	440892	460104	336199	687084	577043	148571	66710	675674	531442	583427	558549
16.2	1	426_2_4 of 4	374875	409883	250848	1038859	840339	574867	90600	2034305	2352434	2318265	1634601
16.2	1	426_3_1 of 4	306484	313259	268868	474214	398265	151375	66601	935018	1149305	1199211	788346
16.2	1	426_3_2 of 4	402010	416796	341176	649771	551818	225421	73846	1435239	2049713	2141321	1196869
16.2	1	426_3_3 of 4	508612	546603	440907	880019	749505	314895	78774	1853411	2773988	2759113	1480086
16.2	1	426_3_4 of 4	564675	613123	465040	1166703	996510	528393	87520	3250451	5458502	5016061	2338004
16.2	2	427_1_1 of 4	364139	350458	515004	167405	163999	12947	21881	139530	39328	46719	62827
16.2	2	427_1_2 of 4	369075	360537	440941	261604	241090	38645	32073	257877	146773	158395	153512
16.2	2	427_1_3 of 4	545416	549829	584706	530090	465496	107300	63783	563806	512603	526271	431141
16.2	2	427_1_4 of 4	459455	471755	461977	541281	464349	146611	60595	803797	885687	840095	567383
16.2	2	427_2_1 of 4	325474	322344	502583	161624	162398	20802	15376	180416	79539	85508	82993
16.2	2	427_2_2 of 4	422213	412597	585221	247127	232061	34037	22989	230402	129092	140829	138753
16.2	2	427_2_3 of 4	314764	322276	414719	233103	217403	40489	20147	273158	176969	185896	163244
16.2	2	427_2_4 of 4	545157	572529	581549	679090	588584	199526	56811	1061621	1205985	1143048	776106
16.2	2	427_3_1 of 4	605243	638724	590778	820788	704628	242101	74243	1216493	1256573	1254077	906721
16.2	2	427_3_2 of 4	825395	995216	868281	1294407	1123040	448685	111267	2278408	2623105	2529371	1695863
16.2	2	427_3_3 of 4	718783	772179	640355	1275168	1067895	520852	110972	2832286	3767652	3441098	2071985
16.2	2	427_3_4 of 4	638684	698600	538723	1397922	1148661	748321	111235	4572469	6950136	5915358	2960126
16.2	3	428_1_1 of 4	599466	600615	700248	410592	384435	38156	53982	186198	60489	78932	114888
16.2	3	428_1_2 of 4	474217	487674	520166	462320	409507	74410	48280	343657	216600	244822	252438
16.2	3	428_1_3 of 4	374134	377966	392137	399811	351043	86107	38610	469319	392622	399853	322379
16.2	3	428_1_4 of 4	407899	421993	404778	502100	432397	122199	49615	662383	639266	639491	491229
16.2	3	428_2_1 of 4	615344	611861	798217	369549	335273	40468	45531	191411	123953	138119	149039
16.2	3	428_2_2 of 4	603090	610203	744848	418593	381161	52368	48962	295287	188010	204034	200645
16.2	3	428_2_3 of 4	788182	801372	871516	692928	625363	124728	72600	585185	495473	521763	464474
16.2	3	428_2_4 of 4	734969	770151	702589	940036	841864	268428	95577	1287144	1383074	1339855	958924

16.2	3	428_3_1 of 4	551060	576864	511823	781146	674594	225784	78861	1233332	1412651	1421117	975243
16.2	3	428_3_2 of 4	399233	432416	346139	808124	695063	375996	59129	2323165	3612057	3155797	1606449
16.2	3	428_3_3 of 4	772294	812925	650814	1325134	1126790	476698	117400	2602380	3412954	3266290	2017916
16.2	3	428_3_4 of 4	876530	928230	715837	1683877	1397961	700837	140324	4115048	6024215	5495404	3066482
0	1	436_1_1 of 4	372672	323976	310101	370744	148599	52599	52922	277665	306083	307367	446996
0	1	436_1_2 of 4	263333	213093	195795	247777	85111	31936	40357	165715	179132	180113	333918
0	1	436_1_3 of 4	286968	235075	223182	273917	98450	36160	40518	192279	210817	207933	353187
0	1	436_1_4 of 4	315923	258256	239896	301362	104402	39883	46950	199120	215734	213658	392491
0	1	436_2_1 of 4	323293	265432	253327	307666	106715	40856	46503	208341	225965	225589	400680
0	1	436_2_2 of 4	306688	250011	239885	292636	108496	40447	41148	210458	231000	230458	373282
0	1	436_2_3 of 4	317517	258978	236746	303910	100767	37992	45293	199673	218677	217641	406682
0	1	436_2_4 of 4	342886	284485	270422	333569	120868	45167	48902	233024	255819	255353	418016
0	1	436_3_1 of 4	317991	264071	251433	305431	112689	42304	45263	216126	236489	237384	388715
0	1	436_3_2 of 4	308219	259149	244823	294979	113718	42068	44017	218584	239036	239099	366024
0	1	436_3_3 of 4	324094	264704	253420	307918	109878	42425	47272	214678	237358	237170	408869
0	1	436_3_4 of 4	323747	268593	256792	312363	114806	42789	46069	221745	241969	242200	395922
0	2	437_1_1 of 4	516070	528121	549846	573313	497105	136645	55231	708634	686261	682061	526698
0	2	437_1_2 of 4	523464	536660	556955	579703	511427	139496	57013	734052	696888	693118	533340
0	2	437_1_3 of 4	496072	504269	522211	553161	466016	130485	53868	674218	651528	642475	498559
0	2	437_1_4 of 4	487106	495030	515774	538851	450252	126934	53488	651159	633433	624415	486570
0	2	437_2_1 of 4	512456	524452	543735	567743	482409	136691	56122	691237	675555	670068	526401
0	2	437_2_2 of 4	530787	543879	567863	586586	515433	143793	58086	719057	701476	692389	531592
0	2	437_2_3 of 4	547914	560457	582130	606885	495866	144651	60385	709216	710060	705212	567366
0	2	437_2_4 of 4	437017	440261	450299	475488	371335	108839	49641	552301	536522	529856	458598
0	2	437_3_1 of 4	504008	517301	537748	560433	488523	133251	54713	710984	672428	667907	512269
0	2	437_3_2 of 4	508494	516824	536915	562803	453969	131551	56727	663191	648630	640181	512174
0	2	437_3_3 of 4	496981	509263	522400	552392	481576	131890	56599	709920	681875	679364	522457
0	2	437_3_4 of 4	529358	540972	561292	584334	508711	139150	57381	728576	700132	695567	535303
0	3	438_1_1 of 4	642056	671060	714761	723730	593229	175002	61793	792573	862741	853148	648638
0	3	438_1_2 of 4	469947	480290	500434	520576	429490	124401	52966	584037	623245	616334	472485
0	3	438_1_3 of 4	458701	462542	475481	500960	346239	109223	50704	519584	564072	559013	461951
0	3	438_1_4 of 4	446126	449112	459836	483283	320846	104075	49321	492872	533461	530754	448278
0	3	438_2_1 of 4	461625	467317	482703	505390	351500	112070	46978	523553	569428	564119	465950
0	3	438_2_2 of 4	444672	450925	465225	484395	391163	115976	47549	541996	573887	568074	442976
0	3	438_2_3 of 4	477413	491291	511301	530296	441876	127443	52576	598827	634091	630741	486054
0	3	438_2_4 of 4	475794	491798	502858	532189	454853	127272	54338	622637	650646	647316	504297
0	3	438_3_1 of 4	513516	528646	544710	579799	487327	137582	55852	647101	686363	677088	516111
0	3	438_3_2 of 4	553898	571475	593775	622611	538086	150215	62779	706289	745302	735899	562520
0	3	438_3_3 of 4	539123	554315	573595	601322	527618	144475	59441	701956	721372	714356	545357
0	3	438_3_4 of 4	577245	596126	613084	642695	566565	156759	64403	782875	773367	765426	576825
10.4	1	406_1_1 of 4	418024			349318	342384	74422	63257	344233	261566	243084	246405
10.4	1	406_1_2 of 4	433848			315012	317945	50846	68157	173821	121210	128520	167179

10.4	1	406_1_3 of 4	418137			458698	426601	118392	83995	566311	518335	463487	414675
10.4	1	406_1_4 of 4	571427			587802	545800	139218	110691	566131	564380	523550	489744
10.4	1	406_2_1 of 4	412407			263792	281100	46313	45890	210075	117984	117763	141260
10.4	1	406_2_2 of 4	546804			512908	491863	114563	92253	433651	381421	364325	378104
10.4	1	406_2_3 of 4	414746			475039	427174	125028	87502	513294	526201	483846	448397
10.4	1	406_2_4 of 4	855003			1282750	1128903	444194	232204	2078961	2476525	2123126	1666738
10.4	1	406_3_1 of 4	757316			1046167	918746	349857	201935	1678901	1919196	1668481	1302750
10.4	1	406_3_2 of 4	720884			998893	891776	343064	185325	1609374	1921281	1671277	1246038
10.4	1	406_3_3 of 4	670081			927426	832235	312327	174523	1480357	1744591	1490445	1141678
10.4	1	406_3_4 of 4	574319			868303	754675	319502	167348	1625632	2043176	1693117	1215611
10.4	2	407_1_1 of 4	467144			474589	444474	120878	97275	488363	512512	472264	431782
10.4	2	407_1_2 of 4	383867			410870	381600	108882	86045	442907	472477	433470	383414
10.4	2	407_1_3 of 4	598913			681528	630001	188761	130680	797553	868439	785257	667897
10.4	2	407_1_4 of 4	493803			581044	528493	166405	124825	722340	822270	734769	610839
10.4	2	407_2_1 of 4	504693			471479	444124	107072	91381	421544	420365	394913	381731
10.4	2	407_2_2 of 4	385882			370922	353844	87753	72027	353088	348045	335180	315018
10.4	2	407_2_3 of 4	538463			546254	514121	135858	107977	576745	627706	591684	542104
10.4	2	407_2_4 of 4	501469			607757	562492	183884	112999	837428	938422	829154	676045
10.4	2	407_3_1 of 4	589342			764410	691617	241249	142136	1055258	1197975	1049458	853487
10.4	2	407_3_2 of 4	801286			1148726	1012995	395639	221645	1859624	2196057	1914301	1446095
10.4	2	407_3_3 of 4	736986			1071687	913569	377451	217863	1751908	2166862	1821732	1385496
10.4	2	407_3_4 of 4	652315			1047126	917516	426625	216511	2201960	2874289	2352583	1601250
10.4	3	408_1_1 of 4	552084			284888	243643	45601	45733	155514	128590	129402	160323
10.4	3	408_1_2 of 4	376283			349473	331738	77032	67086	367640	294267	279027	280968
10.4	3	408_1_3 of 4	428469			444098	408609	111027	84547	457503	473249	435860	405737
10.4	3	408_1_4 of 4	456216			525814	447823	139503	102618	604345	663922	597855	539944
10.4	3	408_2_1 of 4	355032			271204	269824	57779	45714	236053	216135	206495	205411
10.4	3	408_2_2 of 4	447839			389727	376142	94882	65260	384683	392716	366239	336981
10.4	3	408_2_3 of 4	543954			533674	498586	139487	85905	605940	638358	589854	513585
10.4	3	408_2_4 of 4	401882			507421	460614	165772	81252	805956	946780	830934	614344
10.4	3	408_3_1 of 4	537992			667220	614842	202156	114854	919924	983975	913444	714654
10.4	3	408_3_2 of 4	674797			918396	795979	304122	157181	1371028	1610876	1385181	1090385
10.4	3	408_3_3 of 4	685488			944806	825732	333227	165620	1558088	1869797	1579267	1201335
10.4	3	408_3_4 of 4	732679			1109491	980919	425955	207745	2064843	2631999	2185245	1512471

***Supplementary Table 15**: Raw data Fig. 4b-c – Experimental network, concentrations (µM))*
From data shown in Supplementary Table 14, concentrations are calculated according to calibrations for amino acids.
The sample designation corresponds to the scheme '#id#_#chamber#_#fraction# of #totalFractions#', where #id# is the sample ID, #chamber# is the chamber number as shown in Figure 4a, #fraction# is the fraction number inside each chamber as shown in Figure 4a, and #totalFractions# is the total number of all fractions in the chamber. Calibrations are shown in Supplementary Table 4 and at the end of the Supplementary Information (pages 155-165)

dT	rep eat	part	[N] (µM)	[S] (µM)	[G] (µM)	[T] (µM)	[A] (µM)	[P] (µM)	[C] (µM)	[V] (µM)	[I] (µM)	[L] (µM)	[F] (µM)
16.2	1	426_1_1 of 4	28.95	27.77	29.85	18.41	22.41	9.31	24.68	10.31	4.89	5.56	7.88
16.2	1	426_1_2 of 4	31.53	30.26	29.31	25.52	30.53	18.59	32.49	21.72	13.99	14.79	17.80
16.2	1	426_1_3 of 4	40.76	39.18	37.64	36.08	42.17	32.18	42.51	39.10	31.60	31.35	31.76
16.2	1	426_1_4 of 4	36.71	35.74	33.43	34.70	40.58	33.50	39.55	41.31	35.98	34.94	33.79
16.2	1	426_2_1 of 4	31.83	30.36	32.87	20.04	24.91	9.41	26.84	12.36	4.49	5.34	8.31
16.2	1	426_2_2 of 4	30.68	29.22	25.96	24.96	30.10	15.54	30.18	17.74	7.96	8.94	13.13
16.2	1	426_2_3 of 4	33.06	32.71	22.41	42.73	48.23	38.12	44.26	42.15	29.96	31.64	37.03
16.2	1	426_2_4 of 4	28.11	29.14	16.72	64.60	70.24	147.48	60.11	126.90	132.64	125.72	108.37
16.2	1	426_3_1 of 4	22.98	22.27	17.92	29.49	33.29	38.84	44.19	58.33	64.80	65.03	52.26
16.2	1	426_3_2 of 4	30.14	29.63	22.74	40.41	46.13	57.83	48.99	89.53	115.57	116.13	79.35
16.2	1	426_3_3 of 4	38.13	38.86	29.39	54.73	62.65	80.79	52.26	115.62	156.40	149.63	98.12
16.2	1	426_3_4 of 4	42.34	43.59	31.00	72.55	83.30	135.56	58.07	202.77	307.76	272.03	155.00
16.2	2	427_1_1 of 4	27.30	24.92	34.33	10.41	13.71	3.32	14.52	8.70	2.22	2.53	4.17
16.2	2	427_1_2 of 4	27.67	25.63	29.39	16.27	20.15	9.91	21.28	16.09	8.28	8.59	10.18
16.2	2	427_1_3 of 4	40.89	39.09	38.98	32.97	38.91	27.53	42.32	35.17	28.90	28.54	28.58
16.2	2	427_1_4 of 4	34.45	33.54	30.79	33.66	38.81	37.61	40.20	50.14	49.94	45.56	37.61
16.2	2	427_2_1 of 4	24.40	22.92	33.50	10.05	13.57	5.34	10.20	11.25	4.48	4.64	5.50
16.2	2	427_2_2 of 4	31.66	29.34	39.01	15.37	19.40	8.73	15.25	14.37	7.28	7.64	9.20
16.2	2	427_2_3 of 4	23.60	22.91	27.64	14.50	18.17	10.39	13.37	17.04	9.98	10.08	10.82
16.2	2	427_2_4 of 4	40.87	40.71	38.76	42.23	49.20	51.19	37.69	66.23	68.00	61.99	51.45
16.2	2	427_3_1 of 4	45.38	45.41	39.38	51.04	58.90	62.11	49.26	75.89	70.85	68.01	60.11
16.2	2	427_3_2 of 4	61.89	70.76	57.88	80.50	93.87	115.11	73.82	142.13	147.90	137.17	112.43
16.2	2	427_3_3 of 4	53.89	54.90	42.68	79.30	89.26	133.63	73.63	176.68	212.43	186.61	137.36
16.2	2	427_3_4 of 4	47.89	49.67	35.91	86.93	96.02	191.98	73.80	285.24	391.87	320.79	196.24
16.2	3	428_1_1 of 4	44.95	42.70	46.68	25.53	32.13	9.79	35.82	11.62	3.41	4.28	7.62
16.2	3	428_1_2 of 4	35.56	34.67	34.67	28.75	34.23	19.09	32.03	21.44	12.21	13.28	16.74
16.2	3	428_1_3 of 4	28.05	26.87	26.14	24.86	29.34	22.09	25.62	29.28	22.14	21.68	21.37
16.2	3	428_1_4 of 4	30.58	30.00	26.98	31.22	36.14	31.35	32.92	41.32	36.04	34.68	32.57
16.2	3	428_2_1 of 4	46.14	43.50	53.21	22.98	28.03	10.38	30.21	11.94	6.99	7.49	9.88
16.2	3	428_2_2 of 4	45.22	43.39	49.65	26.03	31.86	13.44	32.48	18.42	10.60	11.06	13.30
16.2	3	428_2_3 of 4	59.10	56.98	58.09	43.09	52.27	32.00	48.17	36.50	27.94	28.30	30.79
16.2	3	428_2_4 of 4	55.11	54.76	46.83	58.46	70.37	68.87	63.41	80.29	77.98	72.66	63.57

16.2	3	428_3_1 of 4	41.32	41.02	34.12	48.58	56.39	57.93	52.32	76.94	79.65	77.07	64.65
16.2	3	428_3_2 of 4	29.93	30.75	23.07	50.26	58.10	96.46	39.23	144.92	203.66	171.14	106.50
16.2	3	428_3_3 of 4	57.90	57.80	43.38	82.41	94.19	122.30	77.89	162.34	192.43	177.13	133.78
16.2	3	428_3_4 of 4	65.72	66.00	47.72	104.72	116.85	179.80	93.10	256.70	339.66	298.02	203.29
0	1	436_1_1 of 4	27.94	23.04	20.67	23.06	12.42	13.49	35.11	17.32	17.26	16.67	29.63
0	1	436_1_2 of 4	19.74	15.15	13.05	15.41	7.11	8.19	26.78	10.34	10.10	9.77	22.14
0	1	436_1_3 of 4	21.52	16.71	14.88	17.03	8.23	9.28	26.88	11.99	11.89	11.28	23.41
0	1	436_1_4 of 4	23.69	18.36	15.99	18.74	8.73	10.23	31.15	12.42	12.16	11.59	26.02
0	1	436_2_1 of 4	24.24	18.87	16.89	19.13	8.92	10.48	30.85	13.00	12.74	12.23	26.56
0	1	436_2_2 of 4	22.99	17.78	15.99	18.20	9.07	10.38	27.30	13.13	13.02	12.50	24.75
0	1	436_2_3 of 4	23.81	18.41	15.78	18.90	8.42	9.75	30.05	12.46	12.33	11.80	26.96
0	1	436_2_4 of 4	25.71	20.23	18.03	20.74	10.10	11.59	32.44	14.54	14.42	13.85	27.71
0	1	436_3_1 of 4	23.84	18.78	16.76	18.99	9.42	10.85	30.03	13.48	13.33	12.87	25.77
0	1	436_3_2 of 4	23.11	18.43	16.32	18.34	9.51	10.79	29.20	13.64	13.48	12.97	24.27
0	1	436_3_3 of 4	24.30	18.82	16.89	19.15	9.18	10.88	31.36	13.39	13.38	12.86	27.11
0	1	436_3_4 of 4	24.27	19.10	17.12	19.43	9.60	10.98	30.56	13.83	13.64	13.13	26.25
0	2	437_1_1 of 4	38.69	37.55	36.65	35.65	41.55	35.06	36.64	44.21	38.69	36.99	34.92
0	2	437_1_2 of 4	39.25	38.16	37.13	36.05	42.75	35.79	37.83	45.79	39.29	37.59	35.36
0	2	437_1_3 of 4	37.19	35.85	34.81	34.40	38.95	33.48	35.74	42.06	36.73	34.84	33.05
0	2	437_1_4 of 4	36.52	35.20	34.38	33.51	37.64	32.57	35.49	40.62	35.71	33.86	32.26
0	2	437_2_1 of 4	38.42	37.29	36.24	35.31	40.32	35.07	37.23	43.12	38.09	36.34	34.90
0	2	437_2_2 of 4	39.80	38.67	37.85	36.48	43.08	36.89	38.54	44.86	39.55	37.55	35.24
0	2	437_2_3 of 4	41.08	39.85	38.80	37.74	41.45	37.11	40.06	44.24	40.04	38.24	37.61
0	2	437_2_4 of 4	32.77	31.30	30.02	29.57	31.04	27.92	32.93	34.45	30.25	28.73	30.40
0	2	437_3_1 of 4	37.79	36.78	35.85	34.85	40.84	34.19	36.30	44.35	37.91	36.22	33.96
0	2	437_3_2 of 4	38.13	36.75	35.79	35.00	37.95	33.75	37.64	41.37	36.57	34.72	33.95
0	2	437_3_3 of 4	37.26	36.21	34.82	34.35	40.25	33.84	37.55	44.29	38.45	36.84	34.64
0	2	437_3_4 of 4	39.69	38.46	37.41	36.34	42.52	35.70	38.07	45.45	39.48	37.72	35.49
0	3	438_1_1 of 4	48.14	47.71	47.64	45.01	49.59	44.90	41.00	49.44	48.64	46.27	43.00
0	3	438_1_2 of 4	35.24	34.15	33.36	32.37	35.90	31.92	35.14	36.43	35.14	33.42	31.32
0	3	438_1_3 of 4	34.39	32.89	31.69	31.15	28.94	28.02	33.64	32.41	31.80	30.32	30.63
0	3	438_1_4 of 4	33.45	31.93	30.65	30.05	26.82	26.70	32.72	30.75	30.08	28.78	29.72
0	3	438_2_1 of 4	34.61	33.23	32.18	31.43	29.38	28.75	31.17	32.66	32.11	30.59	30.89
0	3	438_2_2 of 4	33.34	32.06	31.01	30.12	32.70	29.75	31.55	33.81	32.36	30.81	29.37
0	3	438_2_3 of 4	35.79	34.93	34.08	32.98	36.94	32.70	34.88	37.36	35.75	34.21	32.22
0	3	438_2_4 of 4	35.67	34.97	33.52	33.10	38.02	32.65	36.05	38.84	36.69	35.10	33.43
0	3	438_3_1 of 4	38.50	37.59	36.31	36.06	40.74	35.30	37.06	40.37	38.70	36.72	34.22
0	3	438_3_2 of 4	41.53	40.63	39.58	38.72	44.98	38.54	41.65	44.06	42.02	39.91	37.29
0	3	438_3_3 of 4	40.42	39.41	38.23	37.39	44.10	37.07	39.44	43.79	40.67	38.74	36.15
0	3	438_3_4 of 4	43.28	42.39	40.87	39.97	47.36	40.22	42.73	48.84	43.60	41.51	38.24
10.4	1	406_1_1 of 4	24.42			17.20	18.23	12.57	17.78	14.52	10.05	10.53	12.35
10.4	1	406_1_2 of 4	25.34			15.51	16.93	8.59	19.16	7.33	4.66	5.57	8.38

10.4	1	406_1_3 of 4	24.42			22.59	22.71	19.99	23.61	23.89	19.92	20.08	20.79
10.4	1	406_1_4 of 4	33.38			28.95	29.06	23.51	31.11	23.88	21.69	22.68	24.55
10.4	1	406_2_1 of 4	24.09			12.99	14.97	7.82	12.90	8.86	4.54	5.10	7.08
10.4	1	406_2_2 of 4	31.94			25.26	26.19	19.34	25.93	18.29	14.66	15.78	18.95
10.4	1	406_2_3 of 4	24.22			23.40	22.74	21.11	24.60	21.65	20.23	20.96	22.48
10.4	1	406_2_4 of 4	49.94			63.18	60.11	75.00	65.27	87.69	95.19	91.96	83.55
10.4	1	406_3_1 of 4	44.23			51.52	48.92	59.07	56.76	70.82	73.77	72.27	65.30
10.4	1	406_3_2 of 4	42.11			49.20	47.48	57.93	52.09	67.89	73.85	72.39	62.46
10.4	1	406_3_3 of 4	39.14			45.68	44.31	52.74	49.06	62.44	67.06	64.56	57.23
10.4	1	406_3_4 of 4	33.54			42.76	40.18	53.95	47.04	68.57	78.54	73.34	60.93
10.4	2	407_1_1 of 4	27.28			23.37	23.67	20.41	27.34	20.60	19.70	20.46	21.64
10.4	2	407_1_2 of 4	22.42			20.24	20.32	18.38	24.19	18.68	18.16	18.78	19.22
10.4	2	407_1_3 of 4	34.98			33.57	33.54	31.87	36.73	33.64	33.38	34.01	33.48
10.4	2	407_1_4 of 4	28.84			28.62	28.14	28.10	35.09	30.47	31.61	31.83	30.62
10.4	2	407_2_1 of 4	29.48			23.22	23.65	18.08	25.69	17.78	16.16	17.11	19.13
10.4	2	407_2_2 of 4	22.54			18.27	18.84	14.82	20.25	14.89	13.38	14.52	15.79
10.4	2	407_2_3 of 4	31.45			26.90	27.37	22.94	30.35	24.33	24.13	25.63	27.17
10.4	2	407_2_4 of 4	29.29			29.93	29.95	31.05	31.76	35.32	36.07	35.92	33.89
10.4	2	407_3_1 of 4	34.42			37.65	36.83	40.73	39.95	44.51	46.05	45.46	42.78
10.4	2	407_3_2 of 4	46.80			56.57	53.94	66.80	62.30	78.44	84.41	82.92	72.49
10.4	2	407_3_3 of 4	43.05			52.78	48.64	63.73	61.24	73.90	83.29	78.91	69.45
10.4	2	407_3_4 of 4	38.10			51.57	48.85	72.04	60.86	92.88	110.48	101.90	80.27
10.4	3	408_1_1 of 4	32.25			14.03	12.97	7.70	12.86	6.56	4.94	5.61	8.04
10.4	3	408_1_2 of 4	21.98			17.21	17.66	13.01	18.86	15.51	11.31	12.09	14.08
10.4	3	408_1_3 of 4	25.03			21.87	21.76	18.75	23.76	19.30	18.19	18.88	20.34
10.4	3	408_1_4 of 4	26.65			25.90	23.84	23.56	28.84	25.49	25.52	25.90	27.07
10.4	3	408_2_1 of 4	20.74			13.36	14.37	9.76	12.85	9.96	8.31	8.94	10.30
10.4	3	408_2_2 of 4	26.16			19.19	20.03	16.02	18.34	16.23	15.10	15.86	16.89
10.4	3	408_2_3 of 4	31.77			26.28	26.55	23.55	24.15	25.56	24.54	25.55	25.74
10.4	3	408_2_4 of 4	23.47			24.99	24.53	27.99	22.84	34.00	36.39	35.99	30.79
10.4	3	408_3_1 of 4	31.42			32.86	32.74	34.13	32.28	38.80	37.82	39.57	35.82
10.4	3	408_3_2 of 4	39.41			45.23	42.38	51.35	44.18	57.83	61.92	60.00	54.66
10.4	3	408_3_3 of 4	40.04			46.53	43.97	56.27	46.55	65.72	71.87	68.41	60.22
10.4	3	408_3_4 of 4	42.79			54.64	52.23	71.92	58.39	87.10	101.17	94.66	75.81

***Supplementary Table 16**: Raw data Fig. 4b-c – Experimental network, normalization against total concentration of species in chamber network (c_0) per experiment*

For comparison between different species in the network, we normalized each concentration from Supplementary Table 15 against the average concentration of this species in the entire network (thus of all 3 chambers as shown in Supplementary Table 15), similar to the normalization done to data shown in heat maps in Fig. 2-3 and Extended Data Figures. The different chamber volumes of chambers and thus of individual fractions is taken into account (chamber 1 has a volume of 24.7 µl, chambers 2 and 3 have a volume of 14.9 µl).

The sample designation corresponds to the scheme '#id#_#chamber#_#fraction# of #totalFractions#', where #id# is the sample ID, #chamber# is the chamber number as shown in Figure 4a, #fraction# is the fraction number inside each chamber as shown in Figure 4a, and #totalFractions# is the total number of all fractions in the chamber.

dT	rep eat	part	[N] (xc_0)	[S] (xc_0)	[G] (xc_0)	[T] (xc_0)	[A] (xc_0)	[P] (xc_0)	[C] (xc_0)	[V] (xc_0)	[I] (xc_0)	[L] (xc_0)	[F] (xc_0)
16.2	1	426_1_1 of 4	0.871	0.853	1.053	0.499	0.526	0.201	0.606	0.177	0.074	0.089	0.164
16.2	1	426_1_2 of 4	0.949	0.930	1.033	0.692	0.716	0.401	0.798	0.373	0.213	0.236	0.371
16.2	1	426_1_3 of 4	1.227	1.204	1.327	0.978	0.989	0.694	1.044	0.672	0.480	0.500	0.661
16.2	1	426_1_4 of 4	1.105	1.098	1.179	0.941	0.952	0.722	0.971	0.710	0.547	0.557	0.704
16.2	1	426_2_1 of 4	0.958	0.933	1.159	0.543	0.584	0.203	0.659	0.212	0.068	0.085	0.173
16.2	1	426_2_2 of 4	0.924	0.898	0.915	0.677	0.706	0.335	0.741	0.305	0.121	0.142	0.273
16.2	1	426_2_3 of 4	0.995	1.005	0.790	1.159	1.132	0.822	1.087	0.724	0.455	0.504	0.771
16.2	1	426_2_4 of 4	0.846	0.895	0.590	1.752	1.648	3.180	1.476	2.180	2.016	2.004	2.257
16.2	1	426_3_1 of 4	0.692	0.684	0.632	0.800	0.781	0.837	1.085	1.002	0.985	1.037	1.088
16.2	1	426_3_2 of 4	0.907	0.911	0.802	1.096	1.082	1.247	1.203	1.538	1.757	1.851	1.652
16.2	1	426_3_3 of 4	1.148	1.194	1.036	1.484	1.470	1.742	1.284	1.987	2.377	2.386	2.043
16.2	1	426_3_4 of 4	1.275	1.339	1.093	1.967	1.954	2.923	1.426	3.484	4.678	4.337	3.228
16.2	2	427_1_1 of 4	0.732	0.674	0.937	0.285	0.322	0.069	0.391	0.131	0.031	0.040	0.085
16.2	2	427_1_2 of 4	0.742	0.694	0.802	0.445	0.473	0.205	0.573	0.242	0.114	0.134	0.208
16.2	2	427_1_3 of 4	1.097	1.058	1.064	0.902	0.913	0.569	1.140	0.530	0.399	0.445	0.584
16.2	2	427_1_4 of 4	0.924	0.907	0.841	0.921	0.911	0.777	1.083	0.756	0.689	0.711	0.768
16.2	2	427_2_1 of 4	0.654	0.620	0.914	0.275	0.319	0.110	0.275	0.170	0.062	0.072	0.112
16.2	2	427_2_2 of 4	0.849	0.794	1.065	0.421	0.455	0.180	0.411	0.217	0.100	0.119	0.188
16.2	2	427_2_3 of 4	0.633	0.620	0.755	0.397	0.427	0.215	0.360	0.257	0.138	0.157	0.221
16.2	2	427_2_4 of 4	1.096	1.101	1.058	1.156	1.155	1.058	1.015	0.998	0.938	0.967	1.051
16.2	2	427_3_1 of 4	1.217	1.229	1.075	1.397	1.383	1.283	1.327	1.143	0.978	1.061	1.228
16.2	2	427_3_2 of 4	1.660	1.914	1.580	2.204	2.204	2.378	1.989	2.142	2.041	2.140	2.296
16.2	2	427_3_3 of 4	1.445	1.485	1.165	2.171	2.096	2.761	1.984	2.662	2.931	2.911	2.805
16.2	2	427_3_4 of 4	1.284	1.344	0.980	2.380	2.254	3.967	1.988	4.298	5.407	5.005	4.008
16.2	3	428_1_1 of 4	1.042	1.013	1.180	0.603	0.647	0.200	0.811	0.177	0.047	0.065	0.148
16.2	3	428_1_2 of 4	0.824	0.823	0.876	0.679	0.689	0.389	0.725	0.327	0.168	0.201	0.324
16.2	3	428_1_3 of 4	0.650	0.638	0.661	0.587	0.591	0.451	0.580	0.446	0.305	0.329	0.414
16.2	3	428_1_4 of 4	0.709	0.712	0.682	0.738	0.728	0.639	0.745	0.630	0.497	0.526	0.631

16.2	3	428_2_1 of 4	1.070	1.032	1.345	0.543	0.564	0.212	0.684	0.182	0.096	0.114	0.191
16.2	3	428_2_2 of 4	1.048	1.029	1.255	0.615	0.642	0.274	0.735	0.281	0.146	0.168	0.258
16.2	3	428_2_3 of 4	1.370	1.352	1.468	1.018	1.053	0.653	1.091	0.557	0.385	0.429	0.597
16.2	3	428_2_4 of 4	1.278	1.299	1.184	1.381	1.417	1.405	1.436	1.224	1.076	1.102	1.232
16.2	3	428_3_1 of 4	0.958	0.973	0.862	1.148	1.136	1.181	1.185	1.173	1.099	1.168	1.252
16.2	3	428_3_2 of 4	0.694	0.729	0.583	1.187	1.170	1.967	0.888	2.210	2.809	2.595	2.063
16.2	3	428_3_3 of 4	1.343	1.371	1.096	1.947	1.897	2.494	1.764	2.475	2.654	2.686	2.592
16.2	3	428_3_4 of 4	1.524	1.566	1.206	2.474	2.353	3.667	2.108	3.914	4.685	4.519	3.938
0	1	436_1_1 of 4	1.181	1.240	1.256	1.222	1.349	1.282	1.166	1.308	1.318	1.326	1.150
0	1	436_1_2 of 4	0.834	0.815	0.793	0.817	0.773	0.778	0.889	0.781	0.771	0.777	0.859
0	1	436_1_3 of 4	0.909	0.900	0.904	0.903	0.894	0.881	0.893	0.906	0.908	0.897	0.908
0	1	436_1_4 of 4	1.001	0.988	0.971	0.994	0.948	0.972	1.034	0.938	0.929	0.922	1.009
0	1	436_2_1 of 4	1.024	1.016	1.026	1.014	0.969	0.996	1.025	0.981	0.973	0.973	1.031
0	1	436_2_2 of 4	0.972	0.957	0.971	0.965	0.985	0.986	0.907	0.991	0.995	0.994	0.960
0	1	436_2_3 of 4	1.006	0.991	0.959	1.002	0.915	0.926	0.998	0.940	0.942	0.939	1.046
0	1	436_2_4 of 4	1.086	1.089	1.095	1.100	1.097	1.101	1.077	1.098	1.102	1.101	1.075
0	1	436_3_1 of 4	1.007	1.010	1.018	1.007	1.023	1.031	0.997	1.018	1.018	1.024	1.000
0	1	436_3_2 of 4	0.976	0.992	0.991	0.973	1.032	1.025	0.970	1.030	1.029	1.031	0.941
0	1	436_3_3 of 4	1.027	1.013	1.026	1.015	0.998	1.034	1.041	1.011	1.022	1.023	1.052
0	1	436_3_4 of 4	1.026	1.028	1.040	1.030	1.042	1.043	1.015	1.044	1.042	1.045	1.018
0	2	437_1_1 of 4	1.018	1.020	1.024	1.021	1.041	1.023	0.993	1.029	1.030	1.033	1.020
0	2	437_1_2 of 4	1.032	1.037	1.037	1.032	1.071	1.044	1.025	1.066	1.046	1.050	1.033
0	2	437_1_3 of 4	0.978	0.974	0.972	0.985	0.976	0.977	0.969	0.979	0.978	0.973	0.965
0	2	437_1_4 of 4	0.960	0.956	0.960	0.959	0.943	0.950	0.962	0.946	0.951	0.946	0.942
0	2	437_2_1 of 4	1.010	1.013	1.012	1.011	1.010	1.023	1.009	1.004	1.014	1.015	1.019
0	2	437_2_2 of 4	1.047	1.050	1.057	1.044	1.079	1.077	1.044	1.044	1.053	1.049	1.029
0	2	437_2_3 of 4	1.080	1.082	1.084	1.080	1.038	1.083	1.086	1.030	1.066	1.068	1.099
0	2	437_2_4 of 4	0.862	0.850	0.838	0.847	0.777	0.815	0.893	0.802	0.805	0.802	0.888
0	2	437_3_1 of 4	0.994	0.999	1.001	0.998	1.023	0.998	0.984	1.033	1.009	1.012	0.992
0	2	437_3_2 of 4	1.003	0.998	1.000	1.002	0.950	0.985	1.020	0.963	0.973	0.970	0.992
0	2	437_3_3 of 4	0.980	0.984	0.973	0.983	1.008	0.987	1.018	1.031	1.023	1.029	1.012
0	2	437_3_4 of 4	1.044	1.045	1.045	1.040	1.065	1.042	1.032	1.058	1.051	1.053	1.036
0	3	438_1_1 of 4	1.272	1.297	1.332	1.292	1.323	1.332	1.130	1.276	1.310	1.308	1.271
0	3	438_1_2 of 4	0.931	0.928	0.932	0.930	0.958	0.947	0.969	0.941	0.946	0.945	0.926
0	3	438_1_3 of 4	0.909	0.894	0.886	0.895	0.772	0.832	0.927	0.837	0.856	0.857	0.905
0	3	438_1_4 of 4	0.884	0.868	0.857	0.863	0.716	0.792	0.902	0.794	0.810	0.814	0.878
0	3	438_2_1 of 4	0.914	0.903	0.899	0.903	0.784	0.853	0.859	0.843	0.864	0.865	0.913
0	3	438_2_2 of 4	0.881	0.871	0.867	0.865	0.872	0.883	0.870	0.873	0.871	0.871	0.868
0	3	438_2_3 of 4	0.946	0.949	0.953	0.947	0.986	0.970	0.962	0.964	0.963	0.967	0.952
0	3	438_2_4 of 4	0.942	0.950	0.937	0.950	1.014	0.969	0.994	1.003	0.988	0.992	0.988
0	3	438_3_1 of 4	1.017	1.022	1.015	1.035	1.087	1.047	1.022	1.042	1.042	1.038	1.011
0	3	438_3_2 of 4	1.097	1.104	1.106	1.112	1.200	1.144	1.148	1.137	1.132	1.128	1.102

0	3	438_3_3 of 4	1.068	1.071	1.069	1.074	1.177	1.100	1.087	1.130	1.095	1.095	1.069
0	3	438_3_4 of 4	1.143	1.152	1.142	1.148	1.264	1.193	1.178	1.261	1.174	1.173	1.130
10.4	1	406_1_1 of 4	0.764			0.555	0.594	0.405	0.536	0.407	0.282	0.300	0.371
10.4	1	406_1_2 of 4	0.793			0.500	0.552	0.277	0.577	0.206	0.131	0.159	0.252
10.4	1	406_1_3 of 4	0.764			0.729	0.740	0.644	0.711	0.670	0.559	0.572	0.624
10.4	1	406_1_4 of 4	1.045			0.934	0.947	0.758	0.938	0.670	0.609	0.646	0.737
10.4	1	406_2_1 of 4	0.754			0.419	0.488	0.252	0.389	0.249	0.127	0.145	0.213
10.4	1	406_2_2 of 4	1.000			0.815	0.854	0.623	0.781	0.513	0.412	0.449	0.569
10.4	1	406_2_3 of 4	0.758			0.755	0.741	0.680	0.741	0.607	0.568	0.597	0.675
10.4	1	406_2_4 of 4	1.563			2.038	1.959	2.417	1.967	2.460	2.673	2.619	2.508
10.4	1	406_3_1 of 4	1.384			1.662	1.594	1.904	1.711	1.987	2.072	2.058	1.960
10.4	1	406_3_2 of 4	1.318			1.587	1.547	1.867	1.570	1.905	2.074	2.062	1.875
10.4	1	406_3_3 of 4	1.225			1.473	1.444	1.700	1.478	1.752	1.883	1.839	1.718
10.4	1	406_3_4 of 4	1.050			1.379	1.310	1.739	1.418	1.924	2.205	2.089	1.829
10.4	2	407_1_1 of 4	0.862			0.724	0.748	0.605	0.745	0.545	0.493	0.519	0.592
10.4	2	407_1_2 of 4	0.708			0.627	0.642	0.545	0.659	0.494	0.455	0.477	0.526
10.4	2	407_1_3 of 4	1.105			1.040	1.060	0.944	1.001	0.890	0.836	0.863	0.916
10.4	2	407_1_4 of 4	0.911			0.887	0.889	0.832	0.956	0.806	0.791	0.808	0.838
10.4	2	407_2_1 of 4	0.931			0.719	0.747	0.536	0.700	0.470	0.405	0.434	0.523
10.4	2	407_2_2 of 4	0.712			0.566	0.595	0.439	0.552	0.394	0.335	0.369	0.432
10.4	2	407_2_3 of 4	0.993			0.834	0.865	0.680	0.827	0.643	0.604	0.651	0.743
10.4	2	407_2_4 of 4	0.925			0.927	0.946	0.920	0.866	0.934	0.903	0.912	0.927
10.4	2	407_3_1 of 4	1.087			1.166	1.163	1.207	1.089	1.177	1.153	1.154	1.170
10.4	2	407_3_2 of 4	1.478			1.753	1.704	1.979	1.698	2.074	2.114	2.105	1.983
10.4	2	407_3_3 of 4	1.359			1.635	1.536	1.888	1.669	1.954	2.085	2.003	1.900
10.4	2	407_3_4 of 4	1.203			1.598	1.543	2.134	1.659	2.456	2.766	2.587	2.196
10.4	3	408_1_1 of 4	1.094			0.521	0.495	0.285	0.471	0.215	0.158	0.181	0.276
10.4	3	408_1_2 of 4	0.746			0.639	0.675	0.481	0.691	0.509	0.363	0.391	0.484
10.4	3	408_1_3 of 4	0.849			0.812	0.831	0.694	0.871	0.633	0.583	0.611	0.700
10.4	3	408_1_4 of 4	0.904			0.962	0.911	0.872	1.057	0.836	0.818	0.838	0.931
10.4	3	408_2_1 of 4	0.703			0.496	0.549	0.361	0.471	0.327	0.266	0.289	0.354
10.4	3	408_2_2 of 4	0.887			0.713	0.765	0.593	0.672	0.532	0.484	0.513	0.581
10.4	3	408_2_3 of 4	1.078			0.976	1.014	0.872	0.885	0.839	0.787	0.826	0.885
10.4	3	408_2_4 of 4	0.796			0.928	0.937	1.036	0.837	1.115	1.167	1.164	1.059
10.4	3	408_3_1 of 4	1.066			1.220	1.250	1.263	1.183	1.273	1.213	1.280	1.232
10.4	3	408_3_2 of 4	1.337			1.680	1.619	1.900	1.619	1.898	1.985	1.940	1.880
10.4	3	408_3_3 of 4	1.358			1.728	1.679	2.082	1.706	2.156	2.304	2.212	2.071
10.4	3	408_3_4 of 4	1.452			2.029	1.995	2.662	2.140	2.858	3.243	3.061	2.608

Supplementary Table 17*: Raw data Fig. 5 – Time series of Glycine dimerization*
Initial conditions: $[Gly]_{init}$ = 100 mM, $[TMP]_{init}$ = 100 mM, pH_{init} 10.5 and 90 °C. For calibration see Supplementary Table 4 and pages 166-167.

time (h)	raw data (counts*sec)		concentrations (µM)		yield (%)
	Gly	GlyGly	Gly (G)	GlyGly (GG)	GG*2/(G+GG*2)
0	6312720340	9040218	94.43	0.02	0.04
0	6967202216	13981321	104.61	0.04	0.07
0	6166118414	17641697	92.15	0.05	0.10
0.5	6510501172	1047133617	97.50	3.88	7.37
0.5	6687743434	1146289185	100.26	4.25	7.82
0.5	6348718171	1055393674	94.99	3.91	7.60
1	6459441944	1174264345	96.71	4.36	8.27
1	6727673481	1249481314	100.88	4.64	8.43
1	6369976662	1149068656	95.32	4.26	8.21
2	6325995632	1402091140	94.63	5.23	9.94
2	6822661875	1326929661	102.36	4.94	8.80
2	6221412993	1309351882	93.01	4.87	9.48
4	6468439883	1421298682	96.85	5.30	9.86
4	6936828985	1388467887	104.13	5.17	9.04
4	6367673724	1276750915	95.28	4.75	9.06
8	6597753318	1340206864	98.86	4.99	9.17
8	6974769029	1385874327	104.72	5.16	8.98
8	6531154308	1292143800	97.82	4.81	8.95
24	7338413265	1178712620	110.39	4.38	7.34
24	6688639544	1135167978	100.27	4.21	7.75
24	6470860460	1131697184	96.88	4.20	7.97
52	7623634441	1115505084	114.83	4.14	6.72
52	6896860726	1163193182	103.51	4.32	7.70
122	7589395271	1000804052	114.30	3.70	6.08
122	7579834133	973440155	114.15	3.60	5.93

***Supplementary Table 18**: Raw data Fig. 5 – Variation of TMP concentration*
Initial conditions: specified concentrations of Gly ($[Gly]_{init}$) and TMP ($[TMP]_{init}$), pH_{init} 10.5 and 90 °C for 16 h. Samples with $[Gly]_{init}$ = 100 mM were diluted 10-fold before injection into LC-MS. For calibration see Supplementary Table 4.

$[Gly]_{init}$ (mM)	$[TMP]_{init}$ (mM)	raw data (µAU*min)		concentrations (mM)		yield (%)
		Gly	GlyGly	Gly (G)	GlyGly (GG)	GG*2/(G+GG*2)
100	0.10	5183.78	0.51	83.96	0.02	0.04
100	0.10	6349.56	0.64	102.84	0.02	0.04
100	0.25	5228.90	0.17	84.69	0.00	0.01
100	0.25	5656.49	0.72	91.61	0.02	0.05
100	0.63	4946.96	0.45	80.12	0.01	0.03
100	1.58	5300.33	6.95	85.84	0.20	0.47
100	1.58	5544.85	8.36	89.81	0.24	0.54
100	3.98	5142.59	24.36	83.29	0.71	1.69
100	3.98	5833.27	28.92	94.48	0.85	1.76
100	10.00	5659.15	97.54	91.66	2.86	5.87
100	25.12	4560.18	152.80	73.86	4.48	10.81
100	25.12	4389.42	158.79	71.09	4.65	11.57
100	63.10	4274.35	171.14	69.23	5.01	12.65
100	63.10	4384.69	179.96	71.02	5.27	12.93
100	158.49	4705.16	194.13	76.21	5.69	12.99
100	158.49	4696.34	200.91	76.06	5.89	13.40
10	0.10	6391.10	0.00	10.35	0.000	0.00
10	0.25	7752.41	0.09	12.56	0.000	0.00
10	0.25	7660.96	0.14	12.41	0.000	0.01
10	0.63	7186.75	0.17	11.64	0.000	0.01
10	0.63	6996.91	0.51	11.33	0.001	0.03
10	1.58	6979.76	3.16	11.31	0.009	0.16
10	1.58	6366.92	1.30	10.31	0.004	0.07
10	3.98	6931.43	9.97	11.23	0.029	0.52
10	3.98	7530.53	11.98	12.20	0.035	0.57
10	10.00	7593.52	41.46	12.30	0.121	1.94
10	25.12	6670.56	36.91	10.80	0.108	1.96
10	25.12	7298.20	37.74	11.82	0.111	1.84
10	63.10	6359.76	45.12	10.30	0.132	2.50
10	63.10	6478.81	44.51	10.49	0.130	2.43
10	158.49	6686.47	48.37	10.83	0.142	2.55
10	158.49	6558.22	45.76	10.62	0.134	2.46

***Supplementary Table 19**: Raw data Supplementary Figure 13 – TMP degradation*
Initial conditions: $[TMP]_{init}$ = 200µM (without Gly), pH_{init} 10.5, 90 °C for experimental time. To account for evaporation, we normalized the TMP concentration according to $[TMP]_{normalized} = \frac{[TMP]}{\frac{1}{3}*[PO_4]+\frac{2}{3}*[diPO_4]+[TMP]+[triPO_4]}$.

time (h)	PO_4 (µM)	$diPO_4$ (µM)	TMP (µM)	$triPO_4$ (µM)	$TMP_{normalized}$ (µM)
0	1.50	3.23	210.42	1.06	196.53
0	1.47	3.07	212.20	1.06	196.67
0	1.12	3.09	211.05	0.90	196.89
0.5	1.21	2.94	213.77	0.17	197.66
0.5	1.48	3.24	212.96	0.26	197.30
0.5	1.62	3.14	214.45	0.19	197.40
1	1.31	3.12	211.60	0.49	197.20
1	1.31	3.28	213.31	0.50	197.11
2	1.61	3.26	213.30	0.62	196.93
2	1.61	3.19	216.74	0.69	196.95
2	1.49	3.18	215.33	0.69	196.98
4	2.04	3.26	214.20	1.00	196.46
4	1.80	3.29	214.03	1.04	196.48
4	1.53	3.35	216.28	1.05	196.55
8	2.23	3.58	220.59	1.87	195.57
8	2.41	3.55	217.29	1.55	195.75
8	2.32	3.52	217.53	1.81	195.57
24	8.93	5.56	223.53	2.53	192.09
24	9.19	5.38	223.81	2.29	192.31
24	9.07	5.54	222.65	2.66	191.92
52	24.31	7.67	227.31	2.75	186.87
52	35.11	6.15	247.31	3.62	185.44
52	26.87	7.54	225.93	2.30	186.56

***Supplementary Table 20**: Raw data Fig. 5 – Glycine dimerization in heat flow chamber*
Initial conditions: specified concentration of Gly ($[Gly]_{init}$), $[TMP]_{init}$ = 1 mM, pH_{init} = 10.5, dT = 10 K between 75 °C and 85 °C, dT = 0 K (isothermal) at 85 °C, both for 16 h. Due to technical problems, for the third replicate with $[Gly]_{init}$ = 10 mM, the temperature gradient was not applied for the entire duration of the experiment, which is why we do not take it into account for the calculation of the heat flow-driven enrichment. For calibration see Supplementary Table 4 and pages 166-167.

$[Gly]_{init}$ (mM)	dT (K)	repeat	part in heat flow chamber	raw data (counts*sec)		concentrations (µM)		yield (%)
				Gly	GlyGly	Gly (G)	GlyGly (GG)	GG*2/(G+GG*2)
1	10	1	top	802185500	2985868	0.60	0.00	0.18
1	10	1	II	1176008707	5690031	0.88	0.00	0.24
1	10	1	III	1644844965	14388030	1.23	0.00	0.43
1	10	1	bot	1810375377	15005115	1.36	0.00	0.41
1	10	2	top	1328739417	27717693	1.00	0.01	1.02
1	10	2	II	1260566309	19816549	0.94	0.00	0.77
1	10	2	III	1432268457	29163998	1.07	0.01	1.00
1	10	2	bot	2326716092	83442136	1.74	0.02	1.75
1	10	3	top	1135748152	9942536	0.85	0.00	0.43
1	10	3	II	1366989643	11820745	1.02	0.00	0.43
1	10	3	III	1527888876	10097632	1.15	0.00	0.33
1	10	3	bot	1841297149	14902900	1.38	0.00	0.40
1	0	1	top	1467102500	0	1.10	0.00	0.00
1	0	1	II	1517817237	0	1.14	0.00	0.00
1	0	1	III	1367185730	0	1.02	0.00	0.00
1	0	1	bot	1592836350	0	1.19	0.00	0.00
1	0	2	top	1629955701	0	1.22	0.00	0.00
1	0	2	II	1347055154	0	1.01	0.00	0.00
1	0	2	III	1716629737	0	1.29	0.00	0.00
1	0	2	bot	1474382134	0	1.11	0.00	0.00
1	0	3	top	1610370106	0	1.21	0.00	0.00
1	0	3	II	1473755296	0	1.10	0.00	0.00
1	0	3	III	1561244091	0	1.17	0.00	0.00
1	0	3	bot	1393863214	0	1.04	0.00	0.00
10	0	1	full	1238282812	0	9.28	0.00	0.00
10	0	2	full	1400958908	0	10.50	0.00	0.00
10	10	1	top	1014256241	70605118	10.14	0.17	3.33
10	10	1	II	827227886	60344712	8.27	0.15	3.49

10	10	1	III	871205665	62290152	8.71	0.15	3.42
10	10	1	IV	874632626	65897201	8.74	0.16	3.60
10	10	1	bot	1289953582	110420339	12.89	0.27	4.07
10	10	2	top	892728415	26482216	8.92	0.07	1.45
10	10	2	II	1031353717	28558232	10.31	0.07	1.35
10	10	2	III	1040394989	28412444	10.40	0.07	1.33
10	10	2	IV	959940215	26743552	9.59	0.07	1.36
10	10	2	bot	1041392787	69652955	10.41	0.17	3.21

Supplementary Table 21*: Raw data Extended Data Fig. 2a – 2-aminoazoles in water, 170 µm, 18 h, 10 K.*

repeat k	species	measured values (mAU*min)				concentration (µM) $[A]_{j,k,HPLC}$ calibration acc. to Supplementary Table 3				c_0 (µM)	Normalization (Eq. 1) concentration (x c_0)				Ratio species vs mean (Eq. 4) $[A]_{j,k}/\bar{c}_{j,k} - 1$ (%)		Ratio species against species (shown in heat maps), Eq. 2 $[A]_{j,k}/[B]_{j,k} - 1$ (%)							
		top	II	III	bot	top	II	III	bot		top	II	III	bot	top	bot	top part (blue shade)				bot part (red shade)			
1	2AO	0.259	0.330	0.382	0.656	28.97	37	42.86	73.52	45.59	0.64	0.81	0.94	1.61	12.17	-5.9	2AO	0	20.85	18.07	2AO	0	-6.98	-10.15
	2AT	0.050	0.083	0.081	0.163	22.27	37.17	36.54	73.43	42.35	0.53	0.88	0.86	1.73	-7.18	1.17	2AT	-17.25	0	-2.3	2AT	7.51	0	-3.4
	2AI	0.268	0.405	0.423	0.892	31.5	47.71	49.82	105.04	58.52	0.54	0.82	0.85	1.79	-4.99	4.73	2AI	-15.3	2.36	0	2AI	11.3	3.52	0
	mean concentration per fraction $\bar{c}_{j,k}$										0.57	0.83	0.88	1.71				2AO	2AT	2AI		2AO	2AT	2AI
2	2AO	0.287	0.442	0.464	0.568	32.14	49.58	51.95	63.68	49.34	0.65	1	1.05	1.29	17.78	-10.2	2AO	0	14.68	48.13	2AO	0	-9.86	-18.79
	2AT	0.058	0.098	0.107	0.147	26.19	44.14	48.05	66.01	46.1	0.57	0.96	1.04	1.43	2.7	-0.37	2AT	-12.8	0	29.17	2AT	10.94	0	-9.9
	2AI	0.249	0.508	0.608	0.900	29.32	59.85	71.55	105.95	66.67	0.44	0.9	1.07	1.59	-20.49	10.57	2AI	-32.49	-22.58	0	2AI	23.13	10.99	0
	mean concentration per fraction $\bar{c}_{j,k}$										0.55	0.95	1.06	1.44				2AO	2AT	2AI		2AO	2AT	2AI
3	2AO	0.348	0.376	0.385	0.550	39.05	42.11	43.14	61.64	46.49	0.84	0.91	0.93	1.33	2.68	-4.2	2AO	0	3.37	4.78	2AO	0	-6.89	-5.43
	2AT	0.075	0.073	0.089	0.131	33.66	32.94	40.09	58.99	41.42	0.81	0.8	0.97	1.42	-0.67	2.89	2AT	-3.26	0	1.36	2AT	7.4	0	1.57
	2AI	0.387	0.415	0.453	0.677	45.61	48.88	53.29	79.77	56.89	0.8	0.86	0.94	1.4	-2.01	1.3	2AI	-4.56	-1.34	0	2AI	5.74	-1.55	0
	mean concentration per fraction $\bar{c}_{j,k}$										0.82	0.85	0.94	1.38				2AO	2AT	2AI		2AO	2AT	2AI

Supplementary Table 22*: Raw data Extended Data Fig. 2a – 2-aminoazoles in water, 170 µm, 18 h, 5 K.*

repeat k	species	measured values (mAU*min)				concentration (µM) $[A]_{j,k,HPLC}$ calibration acc. to Supplementary Table 3				c_0 (µM)	Normalization (Eq. 1) concentration (x c_0)				Ratio species vs mean (Eq. 4) $[A]_{j,k}/\bar{c}_{j,k} - 1$ (%)		Ratio species against species (shown in heat maps), Eq. 2 $[A]_{j,k}/[B]_{j,k} - 1$ (%)							
		top	II	III	bot	top	II	III	bot		top	II	III	bot	top	bot	top part (blue shade)				bot part (red shade)			
1	2AO	0.349	0.400	0.440	0.543	39.17	44.81	49.34	60.85	48.54	0.81	0.92	1.02	1.25	5.14	-1.15	2AO	0	3.44	12.78	2AO	0	1.59	-4.8
	2AT	0.083	0.099	0.113	0.132	37.53	44.64	50.89	59.35	48.1	0.78	0.93	1.06	1.23	1.64	-2.7	2AT	-3.32	0	9.04	2AT	-1.57	0	-6.3
	2AI	0.392	0.528	0.550	0.722	46.18	62.19	64.82	84.99	64.55	0.72	0.96	1	1.32	-6.78	3.84	2AI	-11.34	-8.29	0	2AI	5.05	6.72	0
	mean concentration per fraction $\bar{c}_{j,k}$										0.77	0.94	1.03	1.27				2AO	2AT	2AI		2AO	2AT	2AI
2	2AO	0.356	0.399	0.418	0.498	39.96	44.74	46.86	55.8	46.84	0.85	0.96	1	1.19	7.72	-4.85	2AO	0	9.28	14.97	2AO	0	-8.31	-5.85
	2AT	0.066	0.080	0.082	0.110	29.65	35.86	37.08	49.36	37.99	0.78	0.94	0.98	1.3	-1.42	3.78	2AT	-8.49	0	5.2	2AT	9.07	0	2.69
	2AI	0.368	0.496	0.492	0.628	43.33	58.45	57.92	73.9	58.4	0.74	1	0.99	1.27	-6.3	1.07	2AI	-13.02	-4.95	0	2AI	6.21	-2.62	0
	mean concentration per fraction $\bar{c}_{j,k}$										0.79	0.97	0.99	1.25				2AO	2AT	2AI		2AO	2AT	2AI
3	2AO	0.363	0.369	0.427	0.500	40.74	41.41	47.89	56.01	46.51	0.88	0.89	1.03	1.2	7.77	-3.29	2AO	0	9.8	14.55	2AO	0	-3.23	-6.42
	2AT	0.071	0.081	0.093	0.111	31.99	36.54	41.98	49.9	40.1	0.8	0.91	1.05	1.24	-1.85	-0.06	2AT	-8.92	0	4.33	2AT	3.34	0	-3.3
	2AI	0.370	0.448	0.496	0.624	43.63	52.78	58.41	73.42	57.06	0.76	0.92	1.02	1.29	-5.92	3.35	2AI	-12.7	-4.15	0	2AI	6.87	3.41	0
	mean concentration per fraction $\bar{c}_{j,k}$										0.81	0.91	1.03	1.25				2AO	2AT	2AI		2AO	2AT	2AI

Supplementary Table 23*: Raw data Extended Data Fig. 2b and 2f – mixture of non-proteogenic and proteogenic AA in water, 170 µm, 18 h, 4 fractions*

Due to technical problems, for the third replicate the temperature gradient was not applied for the entire duration of the experiment, which is why we do not take it into account for the calculation of the heat flow-driven enrichment.

repeat k	species	measured values (counts*s)				Normalization (Eq. 1) concentration (x c_0)				Ratio species vs mean (Eq. 4) $[A]_{j,k}/\bar{c}_{j,k} - 1$ (%)	
		top	II	III	bot	top	II	III	bot	top	bot
1	Anserine	5661160	14186861	60439034	65311397	0.156	0.39	1.66	1.794	-56.5	15.4
	Arginine	22016377	44274642	153608231	155034889	0.235	0.472	1.639	1.654	-34.3	6.3
	Aspartic acid	122990130	163986536	247094698	235028807	0.64	0.853	1.285	1.222	78.9	-21.4
	Carnosine	9979306	20266295	77231792	82007108	0.211	0.428	1.63	1.731	-41.1	11.3
	Citrulline	117867559	182397721	388083850	381490630	0.441	0.682	1.451	1.426	23.3	-8.3
	Creatinine	125283026	248456331	843952194	849050372	0.242	0.481	1.633	1.643	-32.2	5.6
	Cysteine	4710370	6994902	15602677	23191288	0.373	0.554	1.236	1.837	4.4	18.1
	Cystathionine	35545955	43974432	70715740	62230923	0.669	0.828	1.331	1.172	87.2	-24.7
	Ethanolamine	87566767	153629632	317147701	404955825	0.364	0.638	1.317	1.682	1.7	8.1
	Glutamic acid	160632652	217561571	328811326	317341023	0.627	0.85	1.284	1.239	75.4	-20.3
	Histidine	20491013	34802647	118210601	120636746	0.279	0.473	1.608	1.641	-22.1	5.5
	Homoalanine	355664255	690039385	1955777936	2002729555	0.284	0.552	1.563	1.601	-20.5	2.9
	Homocysteine	62472985	114750157	272380609	286470087	0.339	0.624	1.48	1.557	-5.0	0.1
	Hydroxylysine	24311276	43065626	113774832	110488207	0.333	0.591	1.56	1.515	-6.7	-2.6
	Hydroxyproline	164413172	269219013	575721834	574819884	0.415	0.68	1.454	1.451	16.1	-6.7
	Leu_Isoleu	99494415	257334460	1343860840	1633860520	0.119	0.309	1.612	1.96	-66.6	26.0
	Lysine	16948349	36206710	121302436	120876224	0.23	0.49	1.643	1.637	-35.8	5.3

	Methionine	126216509	298994224	1040431634	937315258	0.21	0.498	1.732	1.56	-41.2	0.3
	Methylhistidine	20519352	57726006	260313528	262878946	0.136	0.384	1.731	1.748	-61.8	12.4
	Ornithine	12608661	19037405	61509756	64691271	0.32	0.482	1.559	1.639	-10.6	5.4
	Phenylalanine	99228691	337584752	1440076029	1605091125	0.114	0.388	1.654	1.844	-68.1	18.5
	Proline	167959106	428909981	1597435540	1599932831	0.177	0.452	1.684	1.687	-50.5	8.4
	Sarcosine	650059441	829292609	1578532943	1444372035	0.578	0.737	1.402	1.283	61.5	-17.5
	Serine	99389885	117112651	172709916	160896091	0.723	0.852	1.256	1.17	102.1	-24.8
	Taurine	33370199	34786476	37685341	33323306	0.959	1	1.083	0.958	168.3	-38.4
	Threonine	91610345	158030941	364403735	365615740	0.374	0.645	1.488	1.493	4.6	-4.0
	Tyrosine	49212648	75668073	191695027	224976919	0.363	0.559	1.416	1.662	1.7	6.8
	Valine	92196100	342808167	1647533619	1613378040	0.1	0.371	1.783	1.746	-72.1	12.3
	mean concentration per fraction $\bar{c}_{j,k}$					0.358	0.581	1.506	1.555		
2	Anserine	6404480	17474518	29985336	95846174	0.171	0.467	0.801	2.561	-58.6	29.0
	Arginine	27485822	62783931	92282637	232426941	0.265	0.605	0.89	2.24	-35.9	12.9
	Aspartic acid	157002104	204312918	221924813	277877904	0.729	0.949	1.031	1.291	76.5	-35.0
	Carnosine	11741861	26339407	41923872	114312753	0.242	0.542	0.863	2.353	-41.5	18.5
	Citrulline	154169887	239605330	298801812	599488263	0.477	0.742	0.925	1.856	15.5	-6.5
	Creatinine	129730202	294549847	527843949	1250163592	0.236	0.535	0.959	2.271	-43.0	14.4
	Cysteine	7921540	7911174	11069908	36479208	0.5	0.499	0.699	2.302	21.0	16.0
	Cystathionine	35166140	50681402	60723148	149900037	0.474	0.684	0.819	2.022	14.9	1.9
	Ethanolamine	147123146	229662500	332703427	462782208	0.502	0.784	1.135	1.579	21.5	-20.5
	Glutamic acid	204632384	268784402	295605805	390310787	0.706	0.927	1.02	1.347	70.9	-32.2
	Histidine	25312291	47300797	81114355	185782898	0.298	0.557	0.956	2.189	-27.8	10.3
	Homoalanine	503607574	904394665	1275829921	2579914641	0.383	0.687	0.97	1.961	-7.4	-1.2

	Homocysteine	82088666	144483585	190180275	364897015	0.42	0.739	0.973	1.867	1.7	-5.9
	Hydroxylysine	31864014	58235170	77978043	148297841	0.403	0.736	0.986	1.875	-2.5	-5.6
	Hydroxyproline	220873325	349506064	435052426	761219465	0.5	0.791	0.985	1.724	21.1	-13.2
	Leu_Isoleu	287471992	340885858	525749398	2051963832	0.359	0.425	0.656	2.56	-13.2	29.0
	Lysine	21651126	48636521	76771640	189021936	0.258	0.579	0.914	2.25	-37.6	13.3
	Methionine	187575370	368927282	547472674	1163121898	0.331	0.651	0.966	2.052	-19.9	3.4
	Methylhistidine	24673653	80214831	135818473	441428608	0.145	0.47	0.796	2.589	-65.0	30.4
	Ornithine	11595276	25018027	38274068	83651840	0.293	0.631	0.966	2.111	-29.2	6.3
	Phenylalanine	173291206	384952919	697423704	1775194935	0.229	0.508	0.92	2.343	-44.6	18.0
	Proline	252436278	549671066	897858212	2014442830	0.272	0.592	0.967	2.169	-34.2	9.3
	Sarcosine	592809379	993915119	1255838065	1799193814	0.511	0.856	1.082	1.55	23.7	-21.9
	Serine	127757429	132936014	162560332	263150074	0.745	0.775	0.947	1.533	80.2	-22.8
	Taurine	35760987	38003994	37791502	39113960	0.949	1.009	1.003	1.038	129.8	-47.7
	Threonine	129423556	197809706	267677576	571881802	0.444	0.678	0.918	1.961	7.4	-1.2
	Tyrosine	74143329	110102016	161703385	222968677	0.521	0.774	1.137	1.568	26.2	-21.0
	Valine	166247816	379689086	726367545	1958382529	0.206	0.47	0.899	2.425	-50.2	22.1
	mean concentration per fraction $\bar{c}_{j,k}$					0.413	0.667	0.935	1.985		

***Supplementary Table 24**: Raw data Extended Data Fig. 2f – mixture of non-proteogenic and proteogenic AA in water, 170 µm, 18 h – enrichments in top fraction, 4 fractions*

**repeat**		**species**	Ratio species against species (shown in heat maps), Eq. 2 $[A]_{j,k}/[B]_{j,k} - 1$ (%)																											
1	**A**	Ans	0.0	-33.8	-75.7	-26.2	-64.7	-35.9	-58.3	-76.8	-57.2	-75.2	-44.2	-45.3	-54.2	-53.4	-62.5	30.3	-32.2	-26.0	14.0	-51.3	36.4	-12.2	-73.1	-78.5	-83.8	-58.4	-57.2	55.9
		Arg	51.0	0.0	-63.3	11.5	-46.7	-3.1	-37.0	-64.9	-35.4	-62.6	-15.7	-17.4	-30.8	-29.6	-43.4	96.8	2.3	11.8	72.1	-26.5	106.1	32.7	-59.3	-67.5	-75.5	-37.2	-35.4	135.4
		Asp	311.3	172.3	0.0	203.6	45.1	163.8	71.4	-4.4	75.9	2.0	129.6	125.0	88.4	91.8	54.1	436.0	178.7	204.5	368.7	100.2	461.1	261.3	10.8	-11.5	-33.3	71.0	76.0	541.1
		Car	35.5	-10.3	-67.1	0.0	-52.2	-13.1	-43.5	-68.5	-42.1	-66.4	-24.4	-25.9	-37.9	-36.8	-49.3	76.5	-8.2	0.3	54.4	-34.1	84.8	19.0	-63.5	-70.9	-78.0	-43.7	-42.0	111.1
		Cit	183.4	87.6	-31.1	109.2	0.0	81.7	18.1	-34.1	21.2	-29.7	58.1	55.0	29.8	32.2	6.2	269.2	92.0	109.8	222.9	37.9	286.6	148.9	-23.7	-39.0	-54.1	17.8	21.2	341.7
		Cre	55.9	3.2	-62.1	15.1	-45.0	0.0	-35.0	-63.8	-33.3	-61.3	-13.0	-14.7	-28.6	-27.3	-41.6	103.2	5.6	15.4	77.7	-24.1	112.7	36.9	-58.0	-66.4	-74.7	-35.2	-33.3	143.0
		Cys	139.9	58.8	-41.7	77.1	-15.3	53.9	0.0	-44.2	2.6	-40.5	33.9	31.2	9.9	11.9	-10.1	212.6	62.5	77.6	173.4	16.8	227.3	110.7	-35.4	-48.4	-61.1	-0.3	2.6	273.9
		Cth	330.3	184.9	4.6	217.7	51.9	176.0	79.4	0.0	84.0	6.7	140.2	135.4	97.1	100.7	61.2	460.7	191.5	218.5	390.4	109.4	487.1	277.9	15.9	-7.4	-30.2	78.9	84.1	570.7
		ETA	133.8	54.8	-43.2	72.6	-17.5	50.0	-2.5	-45.7	0.0	-42.0	30.5	27.9	7.1	9.0	-12.4	204.7	58.4	73.1	166.4	13.8	219.0	105.4	-37.0	-49.7	-62.1	-2.8	0.0	264.4
		Glu	303.3	167.1	-1.9	197.8	42.3	158.7	68.1	-6.3	72.5	0.0	125.1	120.6	84.8	88.1	51.1	425.6	173.3	198.6	359.6	96.3	450.3	254.2	8.6	-13.2	-34.6	67.7	72.6	528.6
		AABA	79.2	18.6	-56.4	32.3	-36.8	14.9	-25.3	-58.4	-23.4	-55.6	0.0	-2.0	-17.9	-16.4	-32.9	133.5	21.4	32.6	104.2	-12.8	144.5	57.4	-51.8	-61.4	-70.9	-25.5	-23.3	179.3
		Hcy	82.8	21.0	-55.6	35.0	-35.5	17.2	-23.8	-57.5	-21.8	-54.7	2.0	0.0	-16.3	-14.7	-31.5	138.2	23.8	35.3	108.3	-11.0	149.4	60.6	-50.8	-60.7	-70.4	-24.0	-21.8	184.9
		His	118.3	44.5	-46.9	61.2	-23.0	40.0	-9.0	-49.3	-6.6	-45.9	21.8	19.4	0.0	1.8	-18.2	184.5	47.9	61.6	148.8	6.3	197.8	91.7	-41.2	-53.0	-64.6	-9.2	-6.6	240.2
		Hyl	114.4	42.0	-47.9	58.3	-24.3	37.5	-10.6	-50.2	-8.3	-46.8	19.7	17.3	-1.8	0.0	-19.7	179.4	45.3	58.7	144.3	4.4	192.5	88.3	-42.3	-53.9	-65.2	-10.9	-8.3	234.2
		Hyp	166.9	76.7	-35.1	97.1	-5.8	71.2	11.3	-38.0	14.2	-33.8	49.0	46.0	22.3	24.5	0.0	247.8	80.9	97.6	204.2	29.9	264.2	134.5	-28.1	-42.6	-56.7	11.0	14.2	316.0
		Leu-Ile	-23.3	-49.2	-81.3	-43.3	-72.9	-50.8	-68.0	-82.2	-67.2	-81.0	-57.2	-58.0	-64.8	-64.2	-71.3	0.0	-48.0	-43.2	-12.5	-62.6	4.7	-32.6	-79.3	-83.5	-87.6	-68.1	-67.2	19.6
		Lys	47.6	-2.3	-64.1	9.0	-47.9	-5.3	-38.5	-65.7	-36.9	-63.4	-17.6	-19.3	-32.4	-31.2	-44.7	92.3	0.0	9.3	68.2	-28.2	101.4	29.6	-60.3	-68.2	-76.1	-38.6	-36.8	130.1
		Met	35.1	-10.6	-67.2	-0.3	-52.3	-13.4	-43.7	-68.6	-42.2	-66.5	-24.6	-26.1	-38.1	-37.0	-49.4	76.0	-8.5	0.0	54.0	-34.2	84.3	18.7	-63.6	-70.9	-78.1	-43.8	-42.2	110.6
		3-MH	-12.3	-41.9	-78.7	-35.2	-69.0	-43.7	-63.4	-79.6	-62.5	-78.2	-51.0	-52.0	-59.8	-59.1	-67.1	14.3	-40.5	-35.0	0.0	-57.3	19.7	-22.9	-76.4	-81.1	-85.8	-63.5	-62.5	36.8
		Orn	105.4	36.0	-50.0	51.7	-27.5	31.8	-14.4	-52.3	-12.1	-49.1	14.7	12.4	-5.9	-4.2	-23.0	167.7	39.2	52.1	134.1	0.0	180.3	80.4	-44.7	-55.8	-66.7	-14.6	-12.1	220.2
		Phe	-26.7	-51.5	-82.2	-45.9	-74.1	-53.0	-69.4	-83.0	-68.7	-81.8	-59.1	-59.9	-66.4	-65.8	-72.5	-4.5	-50.3	-45.7	-16.5	-64.3	0.0	-35.6	-80.3	-84.2	-88.1	-69.5	-68.6	14.2
		Pro	13.8	-24.6	-72.3	-15.9	-59.8	-27.0	-52.5	-73.5	-51.3	-71.8	-36.5	-37.7	-47.8	-46.9	-57.3	48.4	-22.9	-15.7	29.8	-44.6	55.3	0.0	-69.3	-75.5	-81.5	-52.7	-51.3	77.5
		Sar	271.3	145.9	-9.7	174.2	31.1	138.2	54.8	-13.7	58.8	-7.9	107.3	103.2	70.1	73.2	39.1	383.9	151.6	174.9	323.2	80.8	406.7	226.2	0.0	-20.1	-39.8	54.4	58.9	478.8
		Ser	364.7	207.7	13.0	243.1	64.0	198.0	93.7	8.0	98.8	15.2	159.4	154.2	112.9	116.7	74.1	505.5	214.8	244.0	429.6	126.2	534.0	308.1	25.1	0.0	-24.7	93.2	98.8	624.3
		Tau	516.7	308.4	49.9	355.3	117.6	295.6	157.1	43.3	163.8	52.9	244.2	237.4	182.5	187.7	131.0	703.6	317.8	356.5	602.8	200.2	741.4	441.7	66.1	32.7	0.0	156.4	163.9	861.3
		Thr	140.5	59.2	-41.5	77.6	-15.1	54.3	0.3	-44.1	2.9	-40.4	34.2	31.6	10.2	12.2	-9.9	213.4	63.0	78.0	174.1	17.1	228.1	111.2	-35.2	-48.2	-61.0	0.0	2.9	274.9
		Tyr	133.7	54.8	-43.2	72.5	-17.5	49.9	-2.6	-45.7	0.0	-42.1	30.4	27.9	7.1	9.0	-12.4	204.6	58.4	73.0	166.4	13.8	218.9	105.3	-37.1	-49.7	-62.1	-2.8	0.0	264.3
		Val	-35.8	-57.5	-84.4	-52.6	-77.4	-58.8	-73.3	-85.1	-72.6	-84.1	-64.2	-64.9	-70.6	-70.1	-76.0	-16.4	-56.5	-52.5	-26.9	-68.8	-12.5	-43.6	-82.7	-86.2	-89.6	-73.3	-72.5	0.0
			Ans	Arg	Asp	Car	Cit	Cre	Cys	Cth	ETA	Glu	AABA	Hcy	His	Hyl	Hyp	Leu-Ile	Lys	Met	3-MH	Orn	Phe	Pro	Sar	Ser	Tau	Thr	Tyr	Val
			**B**																											
2	**A**	Ans	0.0	-35.4	-76.5	-29.2	-64.1	-27.4	-65.8	-63.9	-65.9	-75.8	-42.6	-55.3	-59.3	-57.5	-65.8	-52.3	-33.6	-48.3	18.3	-41.5	-25.2	-37.1	-66.5	-77.0	-82.0	-61.4	-67.2	-16.9
		Arg	54.8	0.0	-63.7	9.6	-44.5	12.4	-47.0	-44.2	-47.2	-62.5	-11.2	-30.8	-36.9	-34.2	-47.0	-26.1	2.8	-19.9	83.1	-9.4	15.8	-2.5	-48.1	-64.4	-72.1	-40.3	-49.2	28.7
		Asp	326.2	175.3	0.0	201.7	52.8	209.5	45.9	53.7	45.3	3.3	144.5	90.6	73.6	81.0	45.8	103.3	183.0	120.4	404.1	149.3	218.9	168.3	42.8	-2.0	-23.2	64.4	39.9	254.3
		Car	41.3	-8.8	-66.9	0.0	-49.4	2.6	-51.7	-49.1	-51.9	-65.8	-19.0	-36.8	-42.5	-40.0	-51.7	-32.6	-6.2	-27.0	67.1	-17.4	5.7	-11.1	-52.7	-67.5	-74.5	-45.5	-53.6	17.4

Cit	178.9	80.1	-34.6	97.5	0.0	102.6	-4.5	0.6	-4.9	-32.4	60.0	24.7	13.6	18.5	-4.6	33.1	85.2	44.2	229.9	63.1	108.7	75.6	-6.6	-35.9	-49.7	7.6	-8.4	131.9
Cre	37.7	-11.1	-67.7	-2.5	-50.6	0.0	-52.9	-50.3	-53.1	-66.6	-21.0	-38.4	-43.9	-41.5	-52.9	-34.3	-8.6	-28.8	62.9	-19.5	3.0	-13.3	-53.9	-68.4	-75.2	-46.9	-54.8	14.5
Cys	192.2	88.7	-31.5	106.8	4.7	112.2	0.0	5.4	-0.4	-29.2	67.6	30.6	19.0	24.1	0.0	39.4	94.0	51.1	245.5	70.9	118.6	83.9	-2.1	-32.9	-47.3	12.7	-4.1	142.9
Cth	177.3	79.1	-34.9	96.3	-0.6	101.4	-5.1	0.0	-5.5	-32.8	59.1	24.0	12.9	17.8	-5.1	32.3	84.1	43.4	227.9	62.2	107.5	74.5	-7.1	-36.3	-50.0	6.9	-9.0	130.5
ETA	193.4	89.5	-31.2	107.7	5.2	113.1	0.4	5.8	0.0	-28.9	68.3	31.2	19.5	24.6	0.4	40.0	94.8	51.7	247.0	71.6	119.5	84.7	-1.7	-32.6	-47.1	13.1	-3.7	143.9
Glu	312.6	166.5	-3.2	192.1	47.9	199.6	41.2	48.8	40.6	0.0	136.7	84.5	68.1	75.3	41.2	96.9	174.0	113.3	388.0	141.3	208.7	159.7	38.2	-5.2	-25.6	59.1	35.4	243.0
AABA	74.3	12.6	-59.1	23.4	-37.5	26.6	-40.3	-37.1	-40.6	-57.8	0.0	-22.1	-29.0	-26.0	-40.4	-16.9	15.7	-9.9	106.1	1.9	30.4	9.7	-41.6	-59.9	-68.6	-32.8	-42.8	44.9
Hcy	123.6	44.4	-47.5	58.3	-19.8	62.4	-23.4	-19.3	-23.8	-45.8	28.3	0.0	-8.9	-5.0	-23.5	6.7	48.5	15.6	164.5	30.8	67.3	40.8	-25.1	-48.6	-59.7	-13.7	-26.6	85.9
His	145.5	58.6	-42.4	73.8	-12.0	78.3	-16.0	-11.5	-16.3	-40.5	40.9	9.8	0.0	4.3	-16.0	17.1	63.0	26.9	190.3	43.6	83.7	54.5	-17.8	-43.6	-55.8	-5.3	-19.4	104.1
Hyl	135.4	52.1	-44.8	66.7	-15.6	71.0	-19.4	-15.1	-19.8	-42.9	35.1	5.3	-4.1	0.0	-19.4	12.3	56.3	21.7	178.4	37.7	76.2	48.2	-21.1	-45.9	-57.6	-9.2	-22.7	95.7
Hyp	192.3	88.8	-31.4	106.9	4.8	112.2	0.0	5.4	-0.4	-29.2	67.7	30.7	19.0	24.1	0.0	39.4	94.1	51.1	245.6	70.9	118.7	84.0	-2.1	-32.8	-47.3	12.7	-4.1	143.0
Leu-Ile	109.6	35.4	-50.8	48.4	-24.9	52.2	-28.3	-24.4	-28.6	-49.2	20.3	-6.3	-14.6	-11.0	-28.3	0.0	39.2	8.4	147.9	22.6	56.8	31.9	-29.8	-51.8	-62.2	-19.2	-31.2	74.2
Lys	50.6	-2.7	-64.7	6.6	-46.0	9.4	-48.5	-45.7	-48.7	-63.5	-13.6	-32.7	-38.7	-36.0	-48.5	-28.2	0.0	-22.1	78.1	-11.9	12.7	-5.2	-49.6	-65.4	-72.9	-41.9	-50.6	25.2
Met	93.4	24.9	-54.6	36.9	-30.7	40.5	-33.8	-30.2	-34.1	-53.1	11.0	-13.5	-21.2	-17.9	-33.8	-7.7	28.4	0.0	128.7	13.1	44.7	21.7	-35.2	-55.5	-65.1	-25.4	-36.5	60.8
3-MH	-15.4	-45.4	-80.2	-40.1	-69.7	-38.6	-71.1	-69.5	-71.2	-79.5	-51.5	-62.2	-65.6	-64.1	-71.1	-59.7	-43.9	-56.3	0.0	-50.5	-36.7	-46.8	-71.7	-80.6	-84.8	-67.4	-72.2	-29.7
Orn	71.0	10.4	-59.9	21.0	-38.7	24.2	-41.5	-38.3	-41.7	-58.6	-1.9	-23.6	-30.4	-27.4	-41.5	-18.4	13.5	-11.6	102.2	0.0	27.9	7.6	-42.7	-60.7	-69.2	-34.1	-43.9	42.1
Phe	33.7	-13.7	-68.6	-5.4	-52.1	-2.9	-54.3	-51.8	-54.4	-67.6	-23.3	-40.2	-45.6	-43.2	-54.3	-36.2	-11.2	-30.9	58.1	-21.8	0.0	-15.9	-55.2	-69.3	-75.9	-48.5	-56.1	11.1
Pro	58.9	2.6	-62.7	12.5	-43.0	15.4	-45.6	-42.7	-45.8	-61.5	-8.8	-29.0	-35.3	-32.5	-45.6	-24.2	5.5	-17.9	87.9	-7.1	18.9	0.0	-46.8	-63.5	-71.4	-38.7	-47.9	32.1
Sar	198.5	92.8	-30.0	111.4	7.0	116.8	2.2	7.7	1.8	-27.6	71.3	33.5	21.6	26.8	2.2	42.4	98.2	54.4	253.1	74.6	123.4	87.9	0.0	-31.4	-46.2	15.1	-2.0	148.2
Ser	335.1	181.0	2.1	208.0	56.0	216.0	48.9	56.9	48.3	5.4	149.6	94.5	77.2	84.8	48.9	107.6	188.9	125.0	414.6	154.5	225.5	173.9	45.7	0.0	-21.6	67.8	42.8	261.7
Tau	454.8	258.3	30.2	292.8	98.9	302.9	89.9	100.1	89.1	34.5	218.3	148.1	126.0	135.7	89.8	164.7	268.4	186.9	556.2	224.5	315.1	249.2	85.8	27.5	0.0	114.0	82.1	361.2
Thr	159.3	67.5	-39.2	83.6	-7.0	88.3	-11.2	-6.5	-11.6	-37.2	48.8	15.9	5.6	10.1	-11.3	23.7	72.2	34.1	206.7	51.7	94.0	63.2	-13.1	-40.4	-53.3	0.0	-14.9	115.6
Tyr	204.6	96.8	-28.5	115.7	9.2	121.2	4.3	9.9	3.8	-26.2	74.8	36.2	24.1	29.4	4.2	45.3	102.3	57.5	260.3	78.2	127.9	91.8	2.0	-30.0	-45.1	17.5	0.0	153.3
Val	20.3	-22.3	-71.8	-14.8	-56.9	-12.6	-58.8	-56.6	-59.0	-70.8	-31.0	-46.2	-51.0	-48.9	-58.8	-42.6	-20.1	-37.8	42.3	-29.6	-10.0	-24.3	-59.7	-72.4	-78.3	-53.6	-60.5	0.0
	Ans	Arg	Asp	Car	Cit	Cre	Cys	Cth	ETA	Glu	AABA	Hcy	His	Hyl	Hyp	Leu-Ile	Lys	Met	3-MH	Orn	Phe	Pro	Sar	Ser	Tau	Thr	Tyr	Val
	**B**																											

Supplementary Table 25*: Raw data Extended Data Fig. 2f – mixture of non-proteogenic and proteogenic AA in water, 170 µm, 18 h – enrichments in bottom fraction, 4 fractions*

**repeat**		**species**	Ratio species against species (shown in heat maps), Eq. 2 $[A]_{j,k}/[B]_{j,k}-1$ (%)																											
1	**A**	Ans	0.0	8.5	46.8	3.6	25.8	9.2	-2.3	53.2	6.7	44.8	9.4	12.1	15.3	18.4	23.6	-8.5	9.6	15.0	2.6	9.5	-2.7	6.4	39.8	53.4	87.3	20.2	8.0	2.8
		Arg	-7.8	0.0	35.3	-4.5	16.0	0.7	-10.0	41.2	-1.6	33.5	0.8	3.3	6.2	9.1	14.0	-15.6	1.0	6.0	-5.4	0.9	-10.3	-1.9	28.9	41.4	72.7	10.8	-0.5	-5.3
		Asp	-31.9	-26.1	0.0	-29.4	-14.3	-25.6	-33.5	4.3	-27.3	-1.4	-25.5	-23.6	-21.5	-19.3	-15.8	-37.6	-25.3	-21.7	-30.1	-25.4	-33.7	-27.5	-4.7	4.5	27.6	-18.1	-26.4	-30.0
		Car	-3.5	4.7	41.6	0.0	21.4	5.3	-5.8	47.8	3.0	39.7	5.5	8.1	11.2	14.2	19.3	-11.7	5.7	11.0	-1.0	5.6	-6.1	2.6	34.9	48.0	80.7	16.0	4.2	-0.9
		Cit	-20.5	-13.8	16.7	-17.6	0.0	-13.2	-22.4	21.7	-15.2	15.1	-13.1	-10.9	-8.4	-5.9	-1.7	-27.2	-12.9	-8.6	-18.4	-13.0	-22.6	-15.4	11.2	21.9	48.9	-4.5	-14.2	-18.3
		Cre	-8.4	-0.6	34.4	-5.1	15.2	0.0	-10.5	40.3	-2.3	32.6	0.2	2.7	5.6	8.4	13.2	-16.2	0.4	5.3	-6.0	0.2	-10.9	-2.6	28.1	40.5	71.6	10.1	-1.1	-5.9
		Cys	2.4	11.1	50.3	6.1	28.8	11.8	0.0	56.8	9.2	48.2	12.0	14.8	18.0	21.2	26.6	-6.3	12.2	17.7	5.1	12.1	-0.4	8.9	43.2	57.0	91.8	23.1	10.5	5.2
		Cth	-34.7	-29.2	-4.2	-32.3	-17.9	-28.7	-36.2	0.0	-30.3	-5.5	-28.6	-26.8	-24.7	-22.7	-19.3	-40.2	-28.4	-24.9	-33.0	-28.5	-36.5	-30.5	-8.7	0.1	22.3	-21.5	-29.5	-32.9
		ETA	-6.3	1.7	37.6	-2.9	17.9	2.3	-8.5	43.5	0.0	35.7	2.5	5.0	8.0	11.0	15.9	-14.2	2.7	7.8	-3.8	2.6	-8.8	-0.3	31.0	43.7	75.6	12.6	1.2	-3.7
		Glu	-30.9	-25.1	1.4	-28.4	-13.1	-24.6	-32.5	5.8	-26.3	0.0	-24.5	-22.6	-20.4	-18.2	-14.6	-36.8	-24.3	-20.6	-29.1	-24.4	-32.8	-26.5	-3.4	5.9	29.4	-17.0	-25.4	-29.0
		AABA	-8.6	-0.8	34.2	-5.2	15.0	-0.2	-10.7	40.0	-2.4	32.4	0.0	2.5	5.4	8.3	13.0	-16.3	0.2	5.1	-6.2	0.1	-11.0	-2.7	27.8	40.2	71.3	9.9	-1.3	-6.0
		Hcy	-10.8	-3.2	31.0	-7.5	12.2	-2.6	-12.9	36.6	-4.8	29.2	-2.4	0.0	2.8	5.6	10.3	-18.3	-2.2	2.6	-8.4	-2.3	-13.2	-5.1	24.7	36.8	67.1	7.2	-3.7	-8.3
		His	-13.2	-5.9	27.4	-10.1	9.1	-5.3	-15.3	32.9	-7.4	25.6	-5.1	-2.8	0.0	2.7	7.3	-20.6	-4.9	-0.2	-11.0	-5.0	-15.6	-7.7	21.3	33.1	62.5	4.3	-6.3	-10.8
		Hyl	-15.5	-8.4	24.0	-12.5	6.2	-7.8	-17.5	29.3	-9.9	22.3	-7.6	-5.3	-2.7	0.0	4.4	-22.7	-7.4	-2.9	-13.3	-7.6	-17.8	-10.2	18.1	29.5	58.2	1.5	-8.8	-13.2
		Hyp	-19.1	-12.2	18.7	-16.2	1.8	-11.7	-21.0	23.9	-13.7	17.1	-11.5	-9.3	-6.8	-4.2	0.0	-25.9	-11.3	-7.0	-17.0	-11.5	-21.3	-14.0	13.1	24.1	51.5	-2.8	-12.7	-16.9
		Leu-Ile	9.2	18.5	60.3	13.2	37.4	19.3	6.7	67.3	16.6	58.2	19.5	22.4	25.9	29.3	35.0	0.0	19.7	25.6	12.1	19.6	6.3	16.2	52.7	67.5	104.6	31.3	17.9	12.2
		Lys	-8.8	-1.0	33.9	-5.4	14.8	-0.4	-10.9	39.7	-2.6	32.1	-0.2	2.3	5.2	8.0	12.8	-16.5	0.0	4.9	-6.4	-0.1	-11.2	-2.9	27.6	39.9	70.9	9.7	-1.5	-6.2
		Met	-13.0	-5.7	27.6	-9.9	9.4	-5.1	-15.1	33.2	-7.2	25.9	-4.9	-2.5	0.2	3.0	7.5	-20.4	-4.7	0.0	-10.8	-4.8	-15.4	-7.5	21.6	33.4	62.9	4.5	-6.1	-10.6
		3-MH	-2.6	5.7	43.0	1.0	22.6	6.4	-4.8	49.2	4.0	41.1	6.6	9.2	12.3	15.4	20.5	-10.8	6.8	12.1	0.0	6.6	-5.2	3.7	36.2	49.4	82.5	17.1	5.2	0.1
		Orn	-8.6	-0.9	34.1	-5.3	14.9	-0.2	-10.8	39.9	-2.5	32.3	-0.1	2.4	5.3	8.2	12.9	-16.4	0.1	5.1	-6.2	0.0	-11.1	-2.8	27.8	40.1	71.2	9.8	-1.3	-6.1
		Phe	2.8	11.5	50.8	6.5	29.3	12.2	0.4	57.4	9.7	48.8	12.4	15.2	18.4	21.7	27.0	-5.9	12.6	18.2	5.5	12.5	0.0	9.3	43.7	57.6	92.5	23.5	11.0	5.6
		Pro	-6.0	2.0	38.0	-2.6	18.3	2.6	-8.2	44.0	0.3	36.1	2.8	5.4	8.3	11.3	16.2	-13.9	3.0	8.1	-3.5	2.9	-8.5	0.0	31.4	44.2	76.1	13.0	1.5	-3.4
		Sar	-28.5	-22.4	5.0	-25.9	-10.0	-21.9	-30.1	9.5	-23.7	3.6	-21.8	-19.8	-17.6	-15.3	-11.6	-34.5	-21.6	-17.8	-26.6	-21.7	-30.4	-23.9	0.0	9.7	34.0	-14.0	-22.8	-26.5
		Ser	-34.8	-29.3	-4.3	-32.4	-18.0	-28.8	-36.3	-0.1	-30.4	-5.6	-28.7	-26.9	-24.8	-22.8	-19.4	-40.3	-28.5	-25.0	-33.1	-28.6	-36.6	-30.6	-8.8	0.0	22.1	-21.6	-29.6	-33.0
		Tau	-46.6	-42.1	-21.6	-44.7	-32.8	-41.7	-47.9	-18.2	-43.0	-22.7	-41.6	-40.2	-38.5	-36.8	-34.0	-51.1	-41.5	-38.6	-45.2	-41.6	-48.1	-43.2	-25.4	-18.1	0.0	-35.8	-42.4	-45.1
		Thr	-16.8	-9.7	22.1	-13.8	4.7	-9.2	-18.7	27.4	-11.2	20.5	-9.0	-6.7	-4.1	-1.5	2.9	-23.8	-8.8	-4.3	-14.6	-8.9	-19.0	-11.5	16.3	27.6	55.9	0.0	-10.2	-14.5
		Tyr	-7.4	0.5	35.9	-4.0	16.5	1.1	-9.5	41.8	-1.2	34.1	1.3	3.8	6.7	9.7	14.5	-15.2	1.5	6.5	-5.0	1.4	-9.9	-1.5	29.5	42.0	73.5	11.3	0.0	-4.8
		Val	-2.7	5.6	42.8	0.9	22.4	6.3	-4.9	49.0	3.8	40.9	6.4	9.1	12.2	15.2	20.3	-10.9	6.7	11.9	-0.1	6.5	-5.3	3.5	36.1	49.3	82.3	17.0	5.1	0.0
			Ans	Arg	Asp	Car	Cit	Cre	Cys	Cth	ETA	Glu	AABA	Hcy	His	Hyl	Hyp	Leu-Ile	Lys	Met	3-MH	Orn	Phe	Pro	Sar	Ser	Tau	Thr	Tyr	Val
			**B**																											
2	**A**	Ans	0.0	14.3	98.4	8.8	38.0	12.8	11.2	26.6	62.2	90.2	17.0	30.6	37.1	36.6	48.6	0.0	13.8	24.8	-1.1	21.3	9.3	18.0	65.2	67.0	146.6	30.6	63.4	5.6
		Arg	-12.5	0.0	73.6	-4.8	20.7	-1.3	-2.7	10.8	41.9	66.4	2.4	14.3	20.0	19.5	30.0	-12.5	-0.4	9.2	-13.4	6.2	-4.4	3.3	44.5	46.1	115.8	14.3	42.9	-7.6
		Asp	-49.6	-42.4	0.0	-45.1	-30.5	-43.2	-43.9	-36.2	-18.3	-4.2	-41.0	-34.2	-30.9	-31.2	-25.1	-49.6	-42.6	-37.1	-50.1	-38.8	-44.9	-40.5	-16.7	-15.8	24.3	-34.2	-17.7	-46.8
		Car	-8.1	5.0	82.3	0.0	26.8	3.6	2.2	16.3	49.0	74.7	7.5	20.0	26.0	25.5	36.5	-8.1	4.6	14.7	-9.1	11.5	0.4	8.5	51.8	53.4	126.6	20.0	50.1	-3.0

Cit	-27.5	-17.2	43.8	-21.1	0.0	-18.3	-19.4	-8.2	17.5	37.8	-15.2	-5.3	-0.6	-1.0	7.7	-27.5	-17.5	-9.6	-28.3	-12.1	-20.8	-14.4	19.7	21.0	78.7	-5.3	18.4	-23.5
Cre	-11.3	1.4	75.9	-3.5	22.3	0.0	-1.4	12.3	43.8	68.6	3.7	15.8	21.6	21.1	31.7	-11.3	0.9	10.6	-12.3	7.6	-3.1	4.7	46.5	48.1	118.7	15.8	44.8	-6.4
Cys	-10.1	2.8	78.4	-2.2	24.0	1.4	0.0	13.8	45.8	71.0	5.2	17.4	23.3	22.8	33.6	-10.1	2.3	12.2	-11.1	9.1	-1.7	6.1	48.5	50.1	121.7	17.4	46.9	-5.1
Cth	-21.0	-9.7	56.7	-14.1	9.0	-10.9	-12.2	0.0	28.1	50.2	-7.6	3.2	8.3	7.9	17.3	-21.0	-10.1	-1.4	-21.9	-4.2	-13.7	-6.8	30.4	31.9	94.8	3.2	29.0	-16.6
ETA	-38.3	-29.5	22.3	-32.9	-14.9	-30.5	-31.4	-21.9	0.0	17.3	-27.9	-19.5	-15.4	-15.8	-8.4	-38.3	-29.8	-23.1	-39.0	-25.2	-32.6	-27.2	1.8	3.0	52.1	-19.5	0.7	-34.9
Glu	-47.4	-39.9	4.3	-42.8	-27.4	-40.7	-41.5	-33.4	-14.7	0.0	-38.5	-31.3	-27.9	-28.2	-21.9	-47.4	-40.1	-34.4	-48.0	-36.2	-42.5	-37.9	-13.1	-12.2	29.7	-31.3	-14.1	-44.5
AABA	-14.5	-2.3	69.6	-7.0	17.9	-3.6	-4.9	8.2	38.6	62.5	0.0	11.6	17.2	16.7	27.0	-14.5	-2.7	6.7	-15.4	3.7	-6.6	0.9	41.2	42.7	110.8	11.6	39.6	-9.7
Hcy	-23.4	-12.5	51.9	-16.7	5.6	-13.7	-14.8	-3.1	24.2	45.6	-10.4	0.0	5.0	4.6	13.8	-23.4	-12.9	-4.5	-24.3	-7.1	-16.3	-9.6	26.4	27.8	88.8	0.0	25.1	-19.1
His	-27.1	-16.7	44.7	-20.6	0.6	-17.8	-18.9	-7.7	18.3	38.7	-14.7	-4.8	0.0	-0.4	8.3	-27.1	-17.0	-9.0	-27.9	-11.5	-20.3	-13.9	20.4	21.8	79.8	-4.8	19.1	-23.0
Hyl	-26.8	-16.3	45.3	-20.3	1.0	-17.4	-18.6	-7.3	18.7	39.2	-14.3	-4.4	0.4	0.0	8.8	-26.8	-16.7	-8.6	-27.6	-11.2	-20.0	-13.6	20.9	22.3	80.6	-4.4	19.6	-22.7
Hyp	-32.7	-23.1	33.5	-26.8	-7.1	-24.1	-25.1	-14.8	9.1	28.0	-21.3	-12.1	-7.7	-8.1	0.0	-32.7	-23.4	-16.0	-33.4	-18.3	-26.4	-20.6	11.2	12.4	66.0	-12.1	9.9	-28.9
Leu-Ile	0.0	14.3	98.3	8.8	37.9	12.7	11.2	26.6	62.1	90.1	17.0	30.6	37.1	36.5	48.5	0.0	13.8	24.8	-1.1	21.3	9.3	18.0	65.1	66.9	146.5	30.6	63.3	5.6
Lys	-12.1	0.4	74.3	-4.4	21.2	-0.9	-2.3	11.2	42.5	67.1	2.8	14.8	20.5	20.0	30.5	-12.1	0.0	9.6	-13.1	6.6	-4.0	3.7	45.1	46.7	116.7	14.8	43.5	-7.2
Met	-19.9	-8.4	59.0	-12.8	10.6	-9.6	-10.9	1.5	30.0	52.4	-6.2	4.7	9.9	9.5	19.1	-19.8	-8.8	0.0	-20.7	-2.8	-12.4	-5.4	32.4	33.8	97.6	4.7	30.9	-15.4
3-MH	1.1	15.5	100.5	10.0	39.5	14.0	12.4	28.0	63.9	92.2	18.3	32.0	38.6	38.1	50.2	1.1	15.1	26.1	0.0	22.6	10.5	19.3	67.0	68.8	149.3	32.0	65.1	6.8
Orn	-17.6	-5.8	63.5	-10.3	13.7	-7.1	-8.3	4.4	33.7	56.7	-3.6	7.7	13.0	12.6	22.5	-17.6	-6.2	2.8	-18.5	0.0	-9.9	-2.7	36.1	37.6	103.3	7.7	34.6	-13.0
Phe	-8.5	4.6	81.5	-0.4	26.2	3.2	1.8	15.8	48.4	74.0	7.0	19.5	25.5	25.0	35.9	-8.5	4.1	14.2	-9.5	11.0	0.0	8.0	51.1	52.8	125.6	19.5	49.4	-3.4
Pro	-15.3	-3.2	68.1	-7.8	16.9	-4.5	-5.8	7.3	37.4	61.1	-0.9	10.7	16.2	15.7	25.9	-15.3	-3.6	5.7	-16.2	2.8	-7.4	0.0	39.9	41.5	108.9	10.7	38.4	-10.5
Sar	-39.5	-30.8	20.1	-34.1	-16.5	-31.7	-32.7	-23.3	-1.8	15.1	-29.2	-20.9	-17.0	-17.3	-10.0	-39.4	-31.1	-24.4	-40.1	-26.5	-33.8	-28.5	0.0	1.1	49.3	-20.9	-1.1	-36.1
Ser	-40.1	-31.6	18.8	-34.8	-17.4	-32.5	-33.4	-24.2	-2.9	13.9	-29.9	-21.8	-17.9	-18.2	-11.0	-40.1	-31.8	-25.3	-40.8	-27.3	-34.5	-29.3	-1.1	0.0	47.7	-21.8	-2.2	-36.8
Tau	-59.5	-53.7	-19.6	-55.9	-44.0	-54.3	-54.9	-48.7	-34.2	-22.9	-52.6	-47.0	-44.4	-44.6	-39.8	-59.4	-53.8	-49.4	-59.9	-50.8	-55.7	-52.1	-33.0	-32.3	0.0	-47.0	-33.8	-57.2
Thr	-23.4	-12.5	51.9	-16.7	5.6	-13.7	-14.8	-3.1	24.2	45.6	-10.4	0.0	5.0	4.6	13.8	-23.4	-12.9	-4.5	-24.3	-7.1	-16.3	-9.6	26.4	27.8	88.8	0.0	25.1	-19.1
Tyr	-38.8	-30.0	21.5	-33.4	-15.5	-31.0	-31.9	-22.5	-0.7	16.4	-28.4	-20.0	-16.0	-16.4	-9.0	-38.8	-30.3	-23.6	-39.4	-25.7	-33.1	-27.7	1.1	2.2	51.0	-20.0	0.0	-35.3
Val	-5.3	8.2	87.9	3.0	30.6	6.8	5.3	19.9	53.6	80.1	10.8	23.7	29.9	29.3	40.7	-5.3	7.8	18.2	-6.3	14.9	3.5	11.8	56.4	58.1	133.5	23.7	54.7	0.0
	Ans	Arg	Asp	Car	Cit	Cre	Cys	Cth	ETA	Glu	AABA	Hcy	His	Hyl	Hyp	Leu-Ile	Lys	Met	3-MH	Orn	Phe	Pro	Sar	Ser	Tau	Thr	Tyr	Val
	**B**																											

Supplementary Table 26*: Raw data Extended Data Fig. 2b and 2g – mixture of non-proteogenic and proteogenic AA in water, 170 µm, 18 h, 12 fractions*

Due to technical problems, for the third replicate the temperature gradient was not applied for the entire duration of the experiment, which is why we do not take it into account for the calculation of the heat flow-driven enrichment.

repeat k	species	measured values (counts*s)												Normalization (Eq. 1) concentration (x c_0)												Ratio species vs mean (Eq. 4) $[A]_{j,k}/\bar{c}_{j,k} - 1$ (%)	
		top	II	III	IV	V	VI	VII	VIII	IX	X	XI	bot	top	II	III	IV	V	VI	VII	VIII	IX	X	XI	bot	top	bot
1	Anserine	9551624	11476674	20504399	17406186	33276686	36216805	18337629	33447667	45194536	46363381	41819528	60292811	0.307	0.368	0.658	0.559	1.068	1.162	0.589	1.074	1.451	1.488	1.342	1.935	-43.6	23.0
	Arginine	37038842	41705006	65839601	52231992	99672725	101961405	55470935	90159454	114588825	119540792	105059990	144486888	0.432	0.487	0.769	0.61	1.164	1.19	0.648	1.053	1.338	1.396	1.227	1.687	-20.4	7.3
	Aspartic acid	150461595	145953487	170605713	117229953	194716385	211639048	103373805	175504822	209882556	223280106	194498068	217398980	0.854	0.828	0.968	0.665	1.105	1.201	0.587	0.996	1.191	1.267	1.104	1.234	57.1	-21.6
	Carnosine	15644775	18652569	29092985	24723017	44303215	47827978	25806768	42895189	53984135	57904881	51951686	66464678	0.392	0.467	0.728	0.619	1.109	1.198	0.646	1.074	1.352	1.45	1.301	1.664	-27.9	5.8
	Citrulline	173661434	168366961	238625378	161742041	314645374	339873622	135081281	273051855	348954715	389212633	328228345	408038849	0.635	0.616	0.873	0.592	1.151	1.244	0.494	0.999	1.277	1.424	1.201	1.493	16.9	-5.1
	Creatinine	230369539	277953525	392167356	298823747	592303027	602632106	298170629	524586207	677118933	749752914	649604378	815272598	0.453	0.546	0.77	0.587	1.164	1.184	0.586	1.03	1.33	1.473	1.276	1.602	-16.7	1.8
	Cysteine	5652566	7090409	9230826	12091505	12926997	16294059	10771118	15849161	16791375	21575517	20445803	20997074	0.4	0.501	0.653	0.855	0.914	1.152	0.762	1.121	1.187	1.526	1.446	1.485	-26.5	-5.6
	Cystathionine	38681187	33522526	45695891	69787202	66514483	71644463	70231078	94328912	82241554	84416868	100225127	104276119	0.539	0.467	0.636	0.972	0.926	0.998	0.978	1.314	1.145	1.176	1.396	1.452	-0.9	-7.7
	Ethanolamine	164469490	192266299	236816469	114752068	280894417	282549134	87294932	167741773	292317598	328833204	257014079	330615813	0.721	0.843	1.039	0.503	1.232	1.239	0.383	0.736	1.282	1.442	1.127	1.45	32.7	-7.8
	Glutamic acid	198724559	190575946	236973157	148872538	266613316	282228759	116294873	216440818	270558073	297281109	247777403	281092701	0.866	0.831	1.033	0.649	1.162	1.23	0.507	0.943	1.179	1.296	1.08	1.225	59.3	-22.1
	Histidine	25641672	36451326	42626390	41036569	75111937	80504779	42706984	71497676	85977437	102075918	91176825	114348725	0.38	0.541	0.632	0.609	1.114	1.194	0.633	1.06	1.275	1.514	1.352	1.696	-30.0	7.8
	Homoalanine	755590904	767010728	1134009322	738195938	1476550145	1555872721	638809096	1215709760	1634329667	1815173852	1521871437	1856091262	0.6	0.609	0.901	0.586	1.173	1.236	0.507	0.966	1.298	1.442	1.209	1.474	10.4	-6.3

	Homocysteine	107383361	100618218	148100980	92329779	197606820	215170011	80382959	161502245	226800017	251477796	204447899	262188746	0.629	0.59	0.868	0.541	1.158	1.261	0.471	0.946	1.329	1.473	1.198	1.536	15.8	-2.3
	Hydroxylysine	36695089	41766457	58600097	44801626	79788674	82210006	44579469	72482933	89718354	89764760	83205037	102985535	0.533	0.606	0.851	0.65	1.158	1.193	0.647	1.052	1.302	1.303	1.208	1.495	-2.0	-4.9
	Hydroxyproline	230999543	238703170	333174485	193952237	417164113	451025836	163397508	342517945	460111924	507947833	410214193	523280794	0.649	0.67	0.936	0.545	1.172	1.267	0.459	0.962	1.292	1.427	1.152	1.47	19.4	-6.6
	Leu_Isoleu	180445360	244097275	508153164	476621534	929786342	958435937	500169818	597497855	961799774	1107059132	821609039	1371678757	0.25	0.338	0.704	0.661	1.289	1.328	0.693	0.828	1.333	1.534	1.139	1.901	-54.0	20.9
	Lysine	29643124	36173761	49271978	45236600	80343795	82023569	43937721	76245385	93803659	96038572	88334757	121097695	0.422	0.515	0.702	0.645	1.145	1.169	0.626	1.086	1.337	1.368	1.259	1.726	-22.3	9.7
	Methionine	358496252	464716840	682513745	254006117	950430520	950216706	198335078	583075489	980967726	1114155233	829652046	1196169208	0.502	0.651	0.956	0.356	1.332	1.332	0.278	0.817	1.375	1.561	1.163	1.676	-7.6	6.6
	Methylhistidine	42020989	49976385	92619620	83740795	166369085	177175651	92373755	161779992	207132319	218050483	207334928	285004146	0.283	0.336	0.623	0.563	1.119	1.192	0.621	1.088	1.394	1.467	1.395	1.918	-48.0	21.9
	Ornithine	15404717	19062340	27512897	26649293	39677308	41491457	32993105	48843889	55829498	64160906	64762243	77419630	0.36	0.445	0.643	0.622	0.927	0.969	0.771	1.141	1.304	1.498	1.513	1.808	-33.8	15.0
	Phenylalanine	336556629	287560302	574396922	297946928	986507147	965862384	234996973	827994575	1096406449	1143064458	879418361	1397286412	0.447	0.382	0.763	0.396	1.311	1.284	0.312	1.101	1.457	1.519	1.169	1.857	-17.7	18.1
	Proline	357262104	427040821	678478008	363839681	980093234	1106185813	332724317	822915982	1206054122	1324371177	1067626639	1507861005	0.421	0.504	0.8	0.429	1.156	1.305	0.392	0.971	1.422	1.562	1.259	1.778	-22.5	13.1
	Sarcosine	806392049	1213048885	896549891	809037826	1148113197	1257312600	573200599	1033648453	1467129349	1410783361	1199808131	1682524265	0.717	1.078	0.797	0.719	1.021	1.118	0.51	0.919	1.304	1.254	1.067	1.496	31.9	-4.9
	Serine	109047496	97004270	113121655	119126251	147163553	165209739	95910066	155608204	172945632	175340449	175049668	192371302	0.762	0.678	0.79	0.832	1.028	1.154	0.67	1.087	1.208	1.225	1.223	1.344	40.1	-14.6
	Taurine	34191182	31754909	30339757	25137711	32156573	33624300	22602410	30761195	33145325	32752528	31723658	32758041	1.106	1.027	0.981	0.813	1.04	1.088	0.731	0.995	1.072	1.06	1.026	1.06	103.5	-32.6
	Threonine	145124895	146617765	215176462	157133707	288580720	311839743	138055972	261027744	323288627	361777712	306733010	398295813	0.57	0.576	0.846	0.617	1.134	1.225	0.543	1.026	1.27	1.422	1.205	1.565	4.9	-0.5
	Tyrosine	69871890	89906246	117420227	136264091	150470029	151957203	143755320	97907781	130406254	142044129	96282784	145076418	0.57	0.733	0.958	1.111	1.227	1.239	1.172	0.799	1.064	1.158	0.785	1.183	4.8	-24.8
	Valine	404369545	409396709	774224716	351429457	1139021352	1310641642	294854542	857446047	1422733809	1598292795	1253505842	1761974587	0.419	0.424	0.802	0.364	1.181	1.358	0.306	0.889	1.475	1.657	1.299	1.826	-22.9	16.1
	mean concentration per fraction $\bar{c}_{j,k}$													0.544	0.595	0.81	0.631	1.131	1.204	0.59	1.003	1.294	1.407	1.219	1.573		

	Anserine	5893436	1777635	9596519	13909351	18794163	19577075	26882871	30097109	37436524	69540104	52022308	147620957	0.163	0.049	0.266	0.385	0.521	0.542	0.745	0.834	1.037	1.927	1.441	4.09	-61.0	41.8
	Arginine	25741098	11335947	35719213	46155927	60948433	64666717	82674249	86448152	105611664	170505033	132032070	340149154	0.266	0.117	0.369	0.477	0.629	0.668	0.854	0.893	1.091	1.761	1.364	3.513	-36.5	21.8
	Aspartic acid	147546584	106701558	163036101	178345913	197359335	179184281	204582259	202414229	220824221	242868700	183494277	265522772	0.773	0.559	0.854	0.934	1.033	0.938	1.071	1.06	1.156	1.272	0.961	1.39	84.5	-51.8
	Carnosine	10174932	4249089	15601240	20588795	27410854	29828470	38043835	40944048	50620354	87610291	68707469	176735955	0.214	0.089	0.328	0.433	0.577	0.627	0.8	0.861	1.065	1.843	1.445	3.717	-48.9	28.9
	Citrulline	142974732	89426104	182313969	209250303	240376389	219355034	264932597	294003491	318710530	473035129	302688186	662135142	0.505	0.316	0.644	0.739	0.849	0.774	0.935	1.038	1.125	1.67	1.069	2.337	20.6	-18.9
	Creatinine	122005096	70672858	192085920	260952070	318718485	344955660	441053782	463539577	602413924	932107917	666217544	1622906987	0.242	0.14	0.382	0.519	0.633	0.686	0.877	0.921	1.197	1.853	1.324	3.226	-42.1	11.9
	Cysteine	12533952	4234829	6624887	7978894	7828999	7598811	8410429	14761395	13676168	44761720	41093117	83682503	0.594	0.201	0.314	0.378	0.371	0.36	0.399	0.7	0.648	2.122	1.948	3.966	41.9	37.6
	Cystathionine	30376600	21268142	38856151	45237709	52692460	44996264	49716072	62523546	56772302	91871419	143764449	173099233	0.449	0.315	0.575	0.669	0.779	0.666	0.735	0.925	0.84	1.359	2.127	2.561	7.3	-11.2
2	Ethanolamine	121534573	68890741	155405513	184120215	184568132	225930186	277769657	264785313	317953343	367746591	179564244	450712563	0.521	0.295	0.666	0.789	0.791	0.969	1.191	1.135	1.363	1.577	0.77	1.932	24.5	-33.0
	Glutamic acid	183904806	123680201	216144436	233468222	254968650	234721976	271283286	276216961	296319840	344542801	222700359	374125343	0.728	0.489	0.855	0.924	1.009	0.929	1.074	1.093	1.173	1.364	0.881	1.481	73.9	-48.6
	Histidine	20253569	6631954	29314690	40269780	54073589	46983977	61486271	64445691	76838166	122096109	102073587	255194569	0.276	0.09	0.4	0.549	0.738	0.641	0.839	0.879	1.048	1.666	1.392	3.481	-34.0	20.7
	Homoalanine	513953252	262957208	653701456	813335613	973878964	988096037	1240897824	1260991271	1554986085	2245710458	1482575476	3367520246	0.402	0.205	0.511	0.635	0.761	0.772	0.97	0.985	1.215	1.755	1.158	2.631	-4.1	-8.7
	Homocysteine	75698129	37687986	99653395	131137551	148550490	137347115	180080518	172615333	220271367	285211728	192877532	419534976	0.432	0.215	0.569	0.749	0.849	0.785	1.029	0.986	1.258	1.629	1.102	2.397	3.3	-16.9
	Hydroxylysine	29683061	15956427	39002669	48513812	58876283	60975485	71459882	70753528	88179215	123330844	95390605	200953000	0.394	0.212	0.518	0.645	0.782	0.81	0.95	0.94	1.172	1.639	1.268	2.67	-5.8	-7.4
	Hydroxyproline	197070504	119636578	244120200	280493687	324665431	301087459	370017367	381137680	445431628	620790747	364640876	834635021	0.527	0.32	0.653	0.751	0.869	0.806	0.99	1.02	1.192	1.661	0.976	2.234	26.0	-22.5
	Leu_Isoleu	153838470	56132001	142707886	207160412	307441860	323693601	426589577	496346266	677186961	1343288635	854065230	2795564738	0.237	0.087	0.22	0.319	0.474	0.499	0.658	0.765	1.044	2.071	1.317	4.31	-43.4	49.5
	Lysine	19591776	9095432	28539068	37779401	48674691	50638843	61116952	64672301	77038366	132042954	106476530	261158509	0.262	0.122	0.382	0.506	0.651	0.678	0.818	0.865	1.031	1.767	1.425	3.494	-37.4	21.2

	Methionine	249203204	111580491	278773772	368279317	453407789	489613755	628478788	626060033	774924568	1056498126	584427379	1723075822	0.407	0.182	0.455	0.602	0.741	0.8	1.027	1.023	1.266	1.726	0.955	2.815	-2.7	-2.4
	Methylhistidine	24430869	5759026	40597265	57272418	74747568	82520511	106654073	120439279	148073347	293619597	227559903	639696507	0.161	0.038	0.267	0.377	0.492	0.544	0.703	0.794	0.976	1.934	1.499	4.215	-61.6	46.2
	Ornithine	13084140	5768966	17874367	20617564	28267141	29484986	39460709	43181724	61882255	99850173	81471766	194748577	0.247	0.109	0.337	0.389	0.534	0.557	0.745	0.815	1.168	1.885	1.538	3.676	-41.0	27.5
	Phenylalanine	168490779	58052434	249384789	329162778	370053402	445837566	585782730	574601163	792030462	1326223362	672500736	2462725353	0.252	0.087	0.372	0.492	0.553	0.666	0.875	0.858	1.183	1.981	1.004	3.678	-39.9	27.6
	Proline	236017583	112343752	341578303	445419052	534546922	533631456	736430931	719891376	990504230	1486432235	856352946	2669210657	0.293	0.14	0.424	0.553	0.664	0.663	0.915	0.894	1.23	1.846	1.064	3.315	-30.0	15.0
	Sarcosine	587604443	406777273	882992259	883417416	955085385	910015892	958659058	1129219561	1173964895	1504850937	1131223383	2290601338	0.55	0.381	0.827	0.827	0.894	0.852	0.898	1.057	1.099	1.409	1.059	2.145	31.4	-25.6
	Serine	98585505	83941834	116063762	123895698	130329649	113525873	122472672	144839060	138143767	193648287	197692394	277376038	0.68	0.579	0.8	0.854	0.899	0.783	0.844	0.999	0.952	1.335	1.363	1.912	62.4	-33.7
	Taurine	29813267	26140438	31144296	30924166	31346749	27242771	29494363	30303265	29546115	31307557	28939314	33941522	0.993	0.871	1.038	1.03	1.044	0.908	0.983	1.01	0.984	1.043	0.964	1.131	137.3	-60.8
	Threonine	114850460	66719547	150577075	171032760	198682380	181557707	224398039	257083132	277219339	455860466	321038776	697175909	0.442	0.257	0.58	0.659	0.765	0.699	0.864	0.99	1.068	1.755	1.236	2.685	5.6	-6.9
	Tyrosine	61105445	37034865	81402758	88729635	104904749	119317513	138044245	121642939	166391366	192820409	131774055	258525361	0.488	0.296	0.65	0.709	0.838	0.953	1.103	0.972	1.33	1.541	1.053	2.066	16.6	-28.3
	Valine	191102105	67784424	260291684	364159224	480303569	546412590	790657861	775506822	1048789881	1813030567	856413117	3156497299	0.222	0.079	0.302	0.422	0.557	0.633	0.917	0.899	1.216	2.102	0.993	3.659	-47.1	26.9
	mean concentration per fraction $\bar{c}_{j,k}$													0.419	0.244	0.52	0.618	0.725	0.722	0.886	0.936	1.112	1.696	1.239	2.883		

***Supplementary Table 27**: Raw data Extended Data Fig. 2g – mixture of non-proteogenic and proteogenic AA in water, 170 µm, 18 h – enrichments in top fraction, 12 fractions*

**repeat**		**species**	Ratio species against species (shown in heat maps), Eq. 2 $[A]_{j,k}/[B]_{j,k} - 1$ (%)																											
1	**A**	Ans	0.0	-29.1	-64.1	-21.7	-51.8	-32.3	-23.3	-43.1	-57.5	-64.6	-19.4	-48.9	-51.3	-42.5	-52.8	22.6	-27.4	-39.0	8.4	-14.8	-31.5	-27.2	-57.2	-59.8	-72.3	-46.2	-46.2	-26.9
		Arg	41.1	0.0	-49.4	10.4	-31.9	-4.4	8.2	-19.7	-40.1	-50.1	13.7	-27.9	-31.3	-18.8	-33.3	72.9	2.4	-13.9	53.0	20.2	-3.3	2.6	-39.7	-43.2	-60.9	-24.2	-24.1	3.2
		Asp	178.5	97.4	0.0	118.0	34.4	88.7	113.6	58.5	18.4	-1.4	124.5	42.3	35.7	60.3	31.6	241.4	102.2	70.0	202.0	137.3	90.9	102.6	19.1	12.1	-22.8	49.7	49.8	103.7
		Car	27.8	-9.4	-54.1	0.0	-38.4	-13.4	-2.0	-27.3	-45.7	-54.8	3.0	-34.7	-37.7	-26.5	-39.6	56.6	-7.3	-22.0	38.6	8.9	-12.4	-7.0	-45.4	-48.6	-64.6	-31.3	-31.3	-6.5
		Cit	107.3	46.9	-25.6	62.2	0.0	40.4	59.0	17.9	-11.9	-26.6	67.1	5.9	1.0	19.3	-2.1	154.1	50.4	26.5	124.8	76.6	42.0	50.8	-11.4	-16.6	-42.5	11.4	11.5	51.6
		Cre	47.6	4.6	-47.0	15.5	-28.8	0.0	13.2	-16.0	-37.3	-47.7	19.0	-24.6	-28.1	-15.1	-30.3	80.9	7.1	-9.9	60.1	25.8	1.2	7.4	-36.9	-40.6	-59.1	-20.6	-20.6	8.0
		Cys	30.4	-7.6	-53.2	2.0	-37.1	-11.7	0.0	-25.8	-44.6	-53.9	5.1	-33.4	-36.5	-25.0	-38.4	59.8	-5.4	-20.4	41.4	11.1	-10.7	-5.1	-44.3	-47.5	-63.9	-29.9	-29.9	-4.6
		Cth	75.7	24.6	-36.9	37.5	-15.2	19.1	34.8	0.0	-25.3	-37.8	41.7	-10.2	-14.4	1.1	-17.0	115.4	27.5	7.2	90.6	49.7	20.4	27.9	-24.9	-29.3	-51.3	-5.5	-5.5	28.5
		ETA	135.3	66.8	-15.5	84.2	13.5	59.4	80.5	33.9	0.0	-16.7	89.7	20.2	14.7	35.4	11.2	188.5	70.8	43.6	155.2	100.5	61.3	71.2	0.6	-5.3	-34.8	26.5	26.6	72.1
		Glu	182.5	100.3	1.4	121.1	36.3	91.4	116.7	60.8	20.0	0.0	127.8	44.3	37.6	62.6	33.5	246.3	105.0	72.4	206.3	140.7	93.6	105.5	20.8	13.7	-21.7	51.9	52.0	106.6
		AABA	24.0	-12.1	-55.5	-2.9	-40.2	-16.0	-4.9	-29.4	-47.3	-56.1	0.0	-36.6	-39.6	-28.6	-41.4	52.0	-10.0	-24.3	34.5	5.7	-15.0	-9.8	-47.0	-50.1	-65.6	-33.3	-33.3	-9.3
		Hcy	95.8	38.8	-29.7	53.2	-5.6	32.6	50.1	11.4	-16.8	-30.7	57.8	0.0	-4.6	12.7	-7.5	139.9	42.1	19.4	112.3	66.8	34.1	42.4	-16.3	-21.2	-45.7	5.2	5.3	43.2
		His	105.2	45.5	-26.3	60.6	-1.0	39.0	57.4	16.8	-12.8	-27.4	65.5	4.8	0.0	18.1	-3.0	151.6	49.0	25.2	122.6	74.9	40.7	49.3	-12.2	-17.4	-43.1	10.3	10.4	50.1
		Hyl	73.8	23.2	-37.6	36.0	-16.2	17.7	33.3	-1.1	-26.2	-38.5	40.1	-11.2	-15.3	0.0	-17.9	113.0	26.1	6.0	88.4	48.1	19.1	26.4	-25.7	-30.1	-51.8	-6.6	-6.5	27.1
		Hyp	111.6	50.0	-24.0	65.6	2.1	43.4	62.3	20.4	-10.1	-25.1	70.6	8.1	3.1	21.8	0.0	159.4	53.6	29.1	129.5	80.3	45.0	54.0	-9.5	-14.8	-41.3	13.8	13.9	54.8
		Leu-Ile	-18.4	-42.2	-70.7	-36.2	-60.6	-44.7	-37.4	-53.6	-65.3	-71.1	-34.2	-58.3	-60.2	-53.0	-61.4	0.0	-40.8	-50.2	-11.5	-30.5	-44.1	-40.6	-65.1	-67.2	-77.4	-56.1	-56.1	-40.3
		Lys	37.8	-2.3	-50.5	7.8	-33.5	-6.7	5.7	-21.6	-41.5	-51.2	11.1	-29.6	-32.9	-20.7	-34.9	68.9	0.0	-15.9	49.4	17.4	-5.6	0.2	-41.1	-44.5	-61.8	-25.9	-25.9	0.8
		Met	63.9	16.2	-41.2	28.3	-20.9	11.0	25.7	-6.7	-30.4	-42.0	32.1	-16.3	-20.2	-5.7	-22.6	100.9	18.9	0.0	77.7	39.6	12.3	19.2	-29.9	-34.0	-54.6	-11.9	-11.8	19.9
		3-MH	-7.8	-34.6	-66.9	-27.8	-55.5	-37.5	-29.3	-47.5	-60.8	-67.4	-25.7	-52.9	-55.1	-46.9	-56.4	13.0	-33.1	-43.7	0.0	-21.4	-36.8	-32.9	-60.6	-62.9	-74.4	-50.4	-50.4	-32.5
		Orn	17.4	-16.8	-57.9	-8.2	-43.4	-20.5	-10.0	-33.2	-50.1	-58.5	-5.4	-40.0	-42.8	-32.5	-44.5	43.8	-14.8	-28.4	27.3	0.0	-19.6	-14.6	-49.8	-52.8	-67.5	-36.9	-36.9	-14.2
		Phe	45.9	3.4	-47.6	14.2	-29.6	-1.1	11.9	-17.0	-38.0	-48.3	17.6	-25.5	-28.9	-16.0	-31.1	78.9	5.9	-11.0	58.2	24.3	0.0	6.2	-37.6	-41.3	-59.6	-21.6	-21.5	6.7
		Pro	37.4	-2.6	-50.7	7.6	-33.7	-6.9	5.4	-21.8	-41.6	-51.3	10.8	-29.8	-33.0	-20.9	-35.1	68.5	-0.2	-16.1	49.0	17.1	-5.8	0.0	-41.2	-44.7	-61.9	-26.1	-26.1	0.5
		Sar	133.9	65.8	-16.0	83.0	12.8	58.4	79.4	33.1	-0.6	-17.2	88.5	19.5	13.9	34.6	10.5	186.6	69.7	42.7	153.6	99.3	60.3	70.1	0.0	-5.9	-35.2	25.7	25.8	71.1
		Ser	148.5	76.1	-10.8	94.5	19.9	68.3	90.6	41.4	5.6	-12.0	100.3	26.9	21.1	43.0	17.4	204.5	80.3	51.6	169.4	111.7	70.3	80.8	6.3	0.0	-31.1	33.6	33.7	81.7
		Tau	260.8	155.8	29.5	182.4	74.1	144.4	176.7	105.3	53.3	27.7	190.9	84.3	75.8	107.6	70.5	342.2	161.9	120.2	291.2	207.4	147.2	162.5	54.3	45.2	0.0	93.9	94.1	163.9
		Thr	86.0	31.9	-33.2	45.6	-10.3	26.0	42.7	5.9	-21.0	-34.2	50.0	-5.0	-9.4	7.1	-12.1	128.0	35.0	13.5	101.7	58.5	27.5	35.3	-20.5	-25.1	-48.4	0.0	0.1	36.1
		Tyr	85.9	31.8	-33.3	45.5	-10.3	25.9	42.6	5.8	-21.0	-34.2	49.9	-5.0	-9.4	7.0	-12.2	127.8	34.9	13.4	101.6	58.4	27.4	35.2	-20.5	-25.2	-48.5	-0.1	0.0	36.0
		Val	36.7	-3.1	-50.9	7.0	-34.0	-7.4	4.9	-22.2	-41.9	-51.6	10.2	-30.2	-33.4	-21.3	-35.4	67.6	-0.8	-16.6	48.2	16.5	-6.3	-0.5	-41.5	-45.0	-62.1	-26.5	-26.5	0.0
			Ans	Arg	Asp	Car	Cit	Cre	Cys	Cth	ETA	Glu	AABA	Hcy	His	Hyl	Hyp	Leu-Ile	Lys	Met	3-MH	Orn	Phe	Pro	Sar	Ser	Tau	Thr	Tyr	Val
			**B**																											
2	**A**	Ans	0.0	-38.6	-78.9	-23.7	-67.7	-32.7	-72.5	-63.7	-68.7	-77.6	-40.9	-59.3	-62.2	-58.6	-69.0	-31.2	-37.7	-59.9	1.4	-33.9	-35.1	-44.3	-70.3	-76.0	-83.6	-63.1	-66.6	-26.3
		Arg	62.8	0.0	-65.6	24.2	-47.3	9.6	-55.3	-40.8	-49.0	-63.5	-3.8	-33.8	-38.5	-32.6	-49.6	12.1	1.4	-34.7	65.2	7.6	5.6	-9.3	-51.7	-60.9	-73.2	-39.9	-45.6	20.0
		Asp	373.2	190.6	0.0	261.0	53.1	218.6	30.0	71.9	48.3	6.1	179.6	92.4	78.7	95.9	46.5	225.7	194.7	89.7	380.0	212.8	207.0	163.6	40.4	13.7	-22.2	74.7	58.2	248.7
		Car	31.1	-19.5	-72.3	0.0	-57.6	-11.7	-64.0	-52.4	-58.9	-70.6	-22.5	-46.7	-50.5	-45.7	-59.4	-9.8	-18.4	-47.4	33.0	-13.4	-15.0	-27.0	-61.1	-68.5	-78.5	-51.6	-56.2	-3.4

Cit	209.1	89.9	-34.7	135.8	0.0	108.1	-15.0	12.3	-3.1	-30.7	82.7	25.7	16.7	28.0	-4.3	112.8	92.5	24.0	213.6	104.4	100.6	72.2	-8.3	-25.7	-49.2	14.1	3.4	127.8
Cre	48.5	-8.8	-68.6	13.3	-52.0	0.0	-59.2	-46.0	-53.5	-66.7	-12.2	-39.6	-43.9	-38.5	-54.0	2.2	-7.5	-40.4	50.7	-1.8	-3.6	-17.3	-55.9	-64.3	-75.6	-45.2	-50.3	9.5
Cys	263.8	123.5	-23.1	177.6	17.7	145.0	0.0	32.2	14.0	-18.4	115.0	47.9	37.4	50.6	12.6	150.5	126.6	45.9	269.1	140.5	136.1	102.7	8.0	-12.6	-40.2	34.3	21.7	168.1
Cth	175.2	69.0	-41.8	110.0	-11.0	85.3	-24.4	0.0	-13.8	-38.3	62.6	11.9	3.9	13.9	-14.8	89.5	71.4	10.4	179.2	81.9	78.6	53.3	-18.3	-33.9	-54.8	1.6	-8.0	102.8
ETA	219.1	96.0	-32.6	143.5	3.2	114.9	-12.3	16.0	0.0	-28.4	88.6	29.8	20.5	32.1	-1.2	119.7	98.8	28.0	223.7	111.0	107.1	77.8	-5.3	-23.3	-47.5	17.8	6.7	135.2
Glu	345.8	173.8	-5.8	240.1	44.2	200.2	22.5	62.0	39.7	0.0	163.4	81.3	68.3	84.5	38.0	206.9	177.6	78.8	352.2	194.7	189.2	148.3	32.3	7.1	-26.7	64.6	49.1	228.5
AABA	69.2	3.9	-64.2	29.1	-45.3	13.9	-53.5	-38.5	-47.0	-62.0	0.0	-31.2	-36.1	-30.0	-47.6	16.5	5.4	-32.1	71.7	11.9	9.8	-5.7	-49.8	-59.4	-72.2	-37.5	-43.4	24.7
Hcy	145.9	51.1	-48.0	87.6	-20.4	65.6	-32.4	-10.6	-22.9	-44.8	45.3	0.0	-7.1	1.8	-23.9	69.3	53.2	-1.4	149.5	62.6	59.6	37.0	-27.0	-40.9	-59.6	-9.2	-17.8	81.3
His	164.8	62.7	-44.0	102.1	-14.3	78.3	-27.2	-3.8	-17.0	-40.6	56.5	7.7	0.0	9.6	-18.0	82.3	65.0	6.2	168.7	75.1	71.8	47.5	-21.4	-36.4	-56.5	-2.2	-11.4	95.2
Hyl	141.6	48.4	-48.9	84.3	-21.9	62.7	-33.6	-12.2	-24.3	-45.8	42.8	-1.8	-8.8	0.0	-25.2	66.3	50.5	-3.1	145.0	59.7	56.7	34.6	-28.3	-42.0	-60.3	-10.8	-19.2	78.0
Hyp	223.0	98.4	-31.7	146.4	4.5	117.5	-11.2	17.4	1.2	-27.5	90.9	31.3	22.0	33.7	0.0	122.4	101.2	29.5	227.7	113.5	109.6	79.9	-4.1	-22.4	-46.9	19.3	8.0	138.1
Leu-Ile	45.3	-10.8	-69.3	10.8	-53.0	-2.2	-60.1	-47.2	-54.5	-67.4	-14.2	-40.9	-45.2	-39.9	-55.0	0.0	-9.5	-41.8	47.3	-4.0	-5.8	-19.1	-56.9	-65.1	-76.1	-46.4	-51.4	7.0
Lys	60.6	-1.4	-66.1	22.5	-48.1	8.1	-55.9	-41.7	-49.7	-64.0	-5.1	-34.7	-39.4	-33.5	-50.3	10.5	0.0	-35.6	62.9	6.1	4.2	-10.6	-52.4	-61.4	-73.6	-40.7	-46.3	18.3
Met	149.4	53.2	-47.3	90.3	-19.3	67.9	-31.5	-9.4	-21.9	-44.1	47.4	1.4	-5.8	3.2	-22.8	71.7	55.3	0.0	153.0	64.9	61.8	38.9	-26.0	-40.1	-59.0	-7.9	-16.6	83.8
3-MH	-1.4	-39.5	-79.2	-24.8	-68.1	-33.6	-72.9	-64.2	-69.1	-77.9	-41.7	-59.9	-62.8	-59.2	-69.5	-32.1	-38.6	-60.5	0.0	-34.8	-36.0	-45.1	-70.7	-76.3	-83.8	-63.6	-67.0	-27.3
Orn	51.3	-7.1	-68.0	15.4	-51.1	1.9	-58.4	-45.0	-52.6	-66.1	-10.6	-38.5	-42.9	-37.4	-53.2	4.1	-5.8	-39.3	53.4	0.0	-1.8	-15.7	-55.1	-63.7	-75.1	-44.2	-49.4	11.5
Phe	54.1	-5.3	-67.4	17.6	-50.1	3.8	-57.6	-44.0	-51.7	-65.4	-8.9	-37.3	-41.8	-36.2	-52.3	6.1	-4.0	-38.2	56.3	1.9	0.0	-14.2	-54.3	-63.0	-74.7	-43.1	-48.5	13.6
Pro	79.5	10.3	-62.1	37.0	-41.9	20.9	-50.7	-34.8	-43.7	-59.7	6.1	-27.0	-32.2	-25.7	-44.4	23.6	11.8	-28.0	82.1	18.7	16.5	0.0	-46.7	-56.9	-70.5	-33.7	-40.0	32.3
Sar	237.0	107.0	-28.8	157.1	9.0	126.9	-7.4	22.5	5.6	-24.4	99.2	37.0	27.3	39.5	4.3	132.0	109.9	35.1	241.9	122.8	118.7	87.7	0.0	-19.0	-44.6	24.4	12.7	148.4
Ser	316.3	155.7	-12.0	217.6	34.7	180.3	14.4	51.3	30.4	-6.6	146.0	69.3	57.2	72.3	28.9	186.6	159.3	66.9	322.3	175.2	170.1	131.9	23.5	0.0	-31.6	53.7	39.2	206.8
Tau	508.4	273.7	28.6	364.2	96.8	309.7	67.2	121.1	90.6	36.5	259.5	147.4	129.7	151.9	88.3	318.9	278.9	144.0	517.2	302.2	294.8	238.9	80.5	46.2	0.0	124.6	103.4	348.4
Thr	170.9	66.4	-42.8	106.7	-12.4	82.4	-25.6	-1.6	-15.1	-39.2	60.1	10.1	2.3	12.1	-16.1	86.5	68.7	8.6	174.8	79.1	75.8	50.9	-19.6	-34.9	-55.5	0.0	-9.4	99.6
Tyr	199.1	83.7	-36.8	128.2	-3.3	101.4	-17.8	8.7	-6.3	-32.9	76.7	21.6	12.9	23.8	-7.4	105.9	86.3	19.9	203.4	97.7	94.0	66.6	-11.3	-28.2	-50.8	10.4	0.0	120.4
Val	35.7	-16.7	-71.3	3.5	-56.1	-8.6	-62.7	-50.7	-57.5	-69.6	-19.8	-44.8	-48.8	-43.8	-58.0	-6.6	-15.5	-45.6	37.6	-10.3	-12.0	-24.4	-59.7	-67.4	-77.7	-49.9	-54.6	0.0
	Ans	Arg	Asp	Car	Cit	Cre	Cys	Cth	ETA	Glu	AABA	Hcy	His	Hyl	Hyp	Leu-Ile	Lys	Met	3-MH	Orn	Phe	Pro	Sar	Ser	Tau	Thr	Tyr	Val
	**B**																											

Supplementary Table 28*: Raw data Extended Data Fig. 2g – mixture of non-proteogenic and proteogenic AA in water, 170 µm, 18 h – enrichments in bottom fraction, 12 fractions*

repeat		species	Ratio species against species (shown in heat maps), Eq. 2 $[A]_{j,k}/[B]_{j,k} - 1$ (%)																											
1	A	Ans	0.0	14.7	56.9	16.3	29.6	20.8	30.3	33.2	33.4	58.0	14.1	31.3	26.0	29.4	31.7	1.8	12.1	15.4	0.9	7.0	4.2	8.8	29.4	44.0	82.6	23.6	63.5	6.0
		Arg	-12.8	0.0	36.7	1.4	13.0	5.3	13.6	16.2	16.3	37.7	-0.5	14.4	9.8	12.8	14.8	-11.3	-2.2	0.6	-12.0	-6.7	-9.2	-5.1	12.8	25.5	59.2	7.8	42.6	-7.6
		Asp	-36.2	-26.9	0.0	-25.9	-17.4	-23.0	-16.9	-15.1	-14.9	0.7	-27.2	-16.3	-19.7	-17.5	-16.1	-35.1	-28.5	-26.4	-35.7	-31.8	-33.6	-30.6	-17.5	-8.2	16.4	-21.2	4.3	-32.4
		Car	-14.0	-1.4	34.9	0.0	11.5	3.9	12.1	14.6	14.7	35.8	-1.9	12.9	8.3	11.3	13.2	-12.5	-3.6	-0.7	-13.2	-8.0	-10.4	-6.4	11.3	23.8	57.0	6.3	40.7	-8.9
		Cit	-22.8	-11.5	21.0	-10.3	0.0	-6.8	0.6	2.8	2.9	21.9	-12.0	1.3	-2.8	-0.1	1.6	-21.5	-13.5	-10.9	-22.1	-17.4	-19.6	-16.0	-0.2	11.1	40.9	-4.6	26.2	-18.2
		Cre	-17.2	-5.1	29.8	-3.8	7.3	0.0	7.9	10.3	10.4	30.7	-5.6	8.6	4.2	7.1	9.0	-15.8	-7.2	-4.5	-16.5	-11.4	-13.8	-9.9	7.1	19.2	51.1	2.3	35.4	-12.3
		Cys	-23.3	-12.0	20.3	-10.8	-0.6	-7.3	0.0	2.2	2.4	21.2	-12.5	0.7	-3.4	-0.7	1.0	-21.9	-14.0	-11.4	-22.6	-17.9	-20.1	-16.5	-0.8	10.5	40.1	-5.1	25.5	-18.7
		Cth	-24.9	-13.9	17.7	-12.7	-2.7	-9.3	-2.2	0.0	0.1	18.6	-14.4	-1.5	-5.5	-2.9	-1.2	-23.6	-15.8	-13.4	-24.3	-19.7	-21.8	-18.3	-2.9	8.1	37.1	-7.2	22.7	-20.5
		ETA	-25.1	-14.0	17.6	-12.9	-2.9	-9.4	-2.3	-0.1	0.0	18.4	-14.5	-1.6	-5.6	-3.0	-1.3	-23.7	-16.0	-13.5	-24.4	-19.8	-21.9	-18.5	-3.0	7.9	36.9	-7.3	22.6	-20.6
		Glu	-36.7	-27.4	-0.7	-26.4	-18.0	-23.5	-17.5	-15.7	-15.5	0.0	-27.8	-16.9	-20.3	-18.1	-16.6	-35.6	-29.0	-26.9	-36.1	-32.2	-34.0	-31.1	-18.1	-8.8	15.6	-21.7	3.5	-32.9
		AABA	-12.4	0.5	37.5	1.9	13.6	5.9	14.2	16.8	16.9	38.4	0.0	15.0	10.4	13.4	15.4	-10.8	-1.7	1.2	-11.6	-6.2	-8.7	-4.6	13.4	26.2	60.0	8.3	43.3	-7.1
		Hcy	-23.8	-12.6	19.5	-11.4	-1.3	-8.0	-0.7	1.5	1.6	20.3	-13.1	0.0	-4.0	-1.4	0.3	-22.5	-14.6	-12.1	-23.1	-18.5	-20.6	-17.1	-1.5	9.7	39.1	-5.8	24.6	-19.3
		His	-20.6	-8.9	24.5	-7.7	2.9	-4.1	3.5	5.8	5.9	25.4	-9.4	4.2	0.0	2.8	4.5	-19.2	-11.0	-8.4	-19.9	-15.0	-17.3	-13.6	2.7	14.3	45.0	-1.8	29.8	-15.9
		Hyl	-22.7	-11.4	21.2	-10.2	0.1	-6.6	0.7	2.9	3.1	22.0	-11.8	1.4	-2.7	0.0	1.7	-21.4	-13.4	-10.8	-22.0	-17.3	-19.5	-15.9	-0.1	11.3	41.1	-4.5	26.4	-18.1
		Hyp	-24.1	-12.9	19.1	-11.7	-1.6	-8.2	-1.0	1.2	1.3	20.0	-13.3	-0.3	-4.3	-1.7	0.0	-22.7	-14.8	-12.3	-23.4	-18.7	-20.9	-17.4	-1.7	9.4	38.7	-6.1	24.2	-19.5
		Leu-Ile	-1.7	12.7	54.1	14.2	27.3	18.7	28.1	30.9	31.1	55.2	12.1	29.0	23.8	27.2	29.4	0.0	10.2	13.4	-0.8	5.2	2.4	6.9	27.1	41.5	79.4	21.5	60.7	4.1
		Lys	-10.8	2.3	39.9	3.7	15.6	7.7	16.2	18.8	19.0	40.9	1.8	17.1	12.3	15.4	17.4	-9.2	0.0	2.9	-10.0	-4.6	-7.1	-3.0	15.4	28.4	62.8	10.2	45.8	-5.5
		Met	-13.4	-0.6	35.9	0.7	12.3	4.7	12.9	15.4	15.6	36.8	-1.1	13.7	9.1	12.1	14.1	-11.8	-2.9	0.0	-12.6	-7.3	-9.7	-5.7	12.1	24.7	58.2	7.1	41.7	-8.2
		3-MH	-0.9	13.7	55.4	15.2	28.4	19.7	29.2	32.0	32.2	56.5	13.1	30.1	24.8	28.3	30.5	0.9	11.1	14.4	0.0	6.0	3.2	7.8	28.2	42.7	80.9	22.5	62.1	5.0
		Orn	-6.6	7.2	46.6	8.6	21.1	12.9	21.8	24.5	24.7	47.6	6.6	22.7	17.7	20.9	23.0	-4.9	4.8	7.9	-5.7	0.0	-2.6	1.7	20.9	34.6	70.6	15.5	52.8	-1.0
		Phe	-4.0	10.1	50.5	11.6	24.4	16.0	25.1	27.9	28.1	51.6	9.5	26.0	20.9	24.2	26.4	-2.3	7.6	10.8	-3.1	2.7	0.0	4.4	24.2	38.2	75.3	18.7	57.0	1.7
		Pro	-8.1	5.4	44.1	6.9	19.1	11.0	19.8	22.4	22.6	45.2	4.9	20.6	15.8	19.0	21.0	-6.5	3.1	6.1	-7.3	-1.6	-4.2	0.0	18.9	32.3	67.8	13.6	50.3	-2.6
		Sar	-22.7	-11.3	21.2	-10.1	0.2	-6.6	0.8	3.0	3.1	22.1	-11.8	1.5	-2.6	0.1	1.8	-21.3	-13.3	-10.8	-22.0	-17.3	-19.5	-15.9	0.0	11.3	41.2	-4.4	26.4	-18.1
		Ser	-30.6	-20.3	8.9	-19.3	-10.0	-16.1	-9.5	-7.5	-7.3	9.7	-20.8	-8.8	-12.5	-10.1	-8.6	-29.3	-22.1	-19.8	-29.9	-25.7	-27.6	-24.4	-10.2	0.0	26.8	-14.1	13.6	-26.4
		Tau	-45.2	-37.2	-14.1	-36.3	-29.0	-33.8	-28.6	-27.0	-26.9	-13.5	-37.5	-28.1	-31.0	-29.1	-27.9	-44.3	-38.6	-36.8	-44.7	-41.4	-42.9	-40.4	-29.2	-21.1	0.0	-32.3	-10.4	-42.0
		Thr	-19.1	-7.2	26.9	-6.0	4.8	-2.3	5.4	7.8	7.9	27.8	-7.7	6.2	1.9	4.7	6.5	-17.7	-9.3	-6.6	-18.4	-13.4	-15.7	-12.0	4.6	16.5	47.7	0.0	32.3	-14.3
		Tyr	-38.9	-29.9	-4.1	-28.9	-20.8	-26.1	-20.3	-18.5	-18.4	-3.4	-30.2	-19.7	-23.0	-20.9	-19.5	-37.8	-31.4	-29.4	-38.3	-34.6	-36.3	-33.5	-20.9	-11.9	11.7	-24.4	0.0	-35.2
		Val	-5.6	8.3	48.0	9.7	22.3	14.0	23.0	25.7	25.9	49.1	7.7	23.9	18.9	22.1	24.3	-3.9	5.8	8.9	-4.8	1.0	-1.7	2.7	22.1	35.9	72.3	16.7	54.3	0.0
			Ans	Arg	Asp	Car	Cit	Cre	Cys	Cth	ETA	Glu	AABA	Hcy	His	Hyl	Hyp	Leu-Ile	Lys	Met	3-MH	Orn	Phe	Pro	Sar	Ser	Tau	Thr	Tyr	Val
			B																											
2	A	Ans	0.0	16.4	194.2	10.0	75.0	26.8	3.1	59.7	111.6	176.2	17.5	55.4	70.6	53.2	83.1	-5.1	17.0	45.3	-3.0	11.2	11.2	23.4	90.7	113.9	261.6	52.3	98.0	11.8
		Arg	-14.1	0.0	152.7	-5.5	50.3	8.9	-11.4	37.2	81.8	137.2	0.9	33.5	46.6	31.6	57.3	-18.5	0.5	24.8	-16.7	-4.4	-4.5	6.0	63.8	83.7	210.6	30.8	70.0	-4.0
		Asp	-66.0	-60.4	0.0	-62.6	-40.5	-56.9	-64.9	-45.7	-28.1	-6.1	-60.1	-47.2	-42.0	-47.9	-37.8	-67.7	-60.2	-50.6	-67.0	-62.2	-62.2	-58.1	-35.2	-27.3	22.9	-48.2	-32.7	-62.0
		Car	-9.1	5.8	167.4	0.0	59.0	15.2	-6.3	45.2	92.4	151.1	6.8	41.3	55.1	39.2	66.4	-13.7	6.4	32.0	-11.8	1.1	1.1	12.1	73.3	94.4	228.7	38.5	79.9	1.6

Cit	-42.8	-33.5	68.1	-37.1	0.0	-27.5	-41.1	-8.7	21.0	57.9	-32.9	-11.2	-2.5	-12.5	4.6	-45.8	-33.1	-17.0	-44.5	-36.4	-36.4	-29.5	9.0	22.2	106.7	-12.9	13.1	-36.1
Cre	-21.1	-8.2	132.0	-13.2	38.0	0.0	-18.7	26.0	66.9	117.8	-7.3	22.6	34.6	20.8	44.4	-25.2	-7.7	14.6	-23.5	-12.3	-12.3	-2.7	50.4	68.7	185.2	20.1	56.1	-11.9
Cys	-3.0	12.9	185.3	6.7	69.7	23.0	0.0	54.9	105.3	167.9	13.9	50.7	65.5	48.5	77.6	-8.0	13.5	40.9	-5.9	7.9	7.8	19.6	84.9	107.4	250.7	47.7	92.0	8.4
Cth	-37.4	-27.1	84.2	-31.1	9.6	-20.6	-35.4	0.0	32.5	72.9	-26.4	-2.7	6.8	-4.1	14.6	-40.6	-26.7	-9.0	-39.2	-30.3	-30.4	-22.8	19.4	33.9	126.4	-4.6	24.0	-30.0
ETA	-52.8	-45.0	39.0	-48.0	-17.3	-40.1	-51.3	-24.5	0.0	30.5	-44.5	-26.6	-19.4	-27.6	-13.5	-55.2	-44.7	-31.4	-54.2	-47.4	-47.5	-41.7	-9.9	1.0	70.9	-28.0	-6.5	-47.2
Glu	-63.8	-57.8	6.5	-60.2	-36.7	-54.1	-62.7	-42.2	-23.4	0.0	-57.5	-43.7	-38.2	-44.5	-33.7	-65.6	-57.6	-47.4	-64.9	-59.7	-59.7	-55.3	-31.0	-22.6	30.9	-44.8	-28.3	-59.5
AABA	-14.9	-0.9	150.4	-6.4	48.9	7.9	-12.2	35.9	80.2	135.1	0.0	32.3	45.3	30.4	55.8	-19.2	-0.4	23.7	-17.4	-5.3	-5.4	5.0	62.3	82.0	207.8	29.7	68.5	-4.9
Hcy	-35.7	-25.1	89.3	-29.2	12.6	-18.4	-33.7	2.7	36.2	77.7	-24.4	0.0	9.8	-1.5	17.8	-38.9	-24.7	-6.5	-37.6	-28.4	-28.5	-20.6	22.7	37.6	132.7	-2.0	27.4	-28.1
His	-41.4	-31.8	72.4	-35.5	2.5	-25.7	-39.6	-6.4	24.0	61.9	-31.2	-8.9	0.0	-10.2	7.3	-44.4	-31.4	-14.9	-43.1	-34.8	-34.8	-27.7	11.7	25.3	111.9	-10.7	16.0	-34.5
Hyl	-34.7	-24.0	92.1	-28.2	14.2	-17.2	-32.7	4.3	38.2	80.3	-23.3	1.5	11.4	0.0	19.5	-38.0	-23.6	-5.2	-36.6	-27.4	-27.4	-19.4	24.5	39.6	136.1	-0.5	29.3	-27.0
Hyp	-45.4	-36.4	60.7	-39.9	-4.4	-30.7	-43.7	-12.8	15.6	50.9	-35.8	-15.1	-6.8	-16.3	0.0	-48.2	-36.1	-20.7	-47.0	-39.2	-39.3	-32.6	4.1	16.8	97.5	-16.8	8.1	-39.0
Leu-Ile	5.4	22.7	210.0	15.9	84.4	33.6	8.7	68.3	123.0	191.1	23.8	63.8	79.8	61.4	92.9	0.0	23.3	53.1	2.3	17.2	17.2	30.0	100.9	125.4	281.1	60.5	108.6	17.8
Lys	-14.6	-0.5	151.4	-6.0	49.5	8.3	-11.9	36.5	80.8	136.0	0.4	32.8	45.8	30.9	56.4	-18.9	0.0	24.1	-17.1	-4.9	-5.0	5.4	62.9	82.7	209.0	30.2	69.2	-4.5
Met	-31.2	-19.9	102.5	-24.3	20.4	-12.7	-29.0	9.9	45.7	90.1	-19.1	7.0	17.5	5.4	26.0	-34.7	-19.4	0.0	-33.2	-23.4	-23.5	-15.1	31.3	47.2	148.9	4.9	36.3	-23.1
3-MH	3.1	20.0	203.2	13.4	80.3	30.7	6.3	64.6	118.1	184.6	21.1	60.2	75.9	57.8	88.7	-2.2	20.6	49.7	0.0	14.6	14.6	27.1	96.5	120.4	272.7	57.0	104.0	15.2
Orn	-10.1	4.7	164.4	-1.1	57.3	14.0	-7.3	43.6	90.3	148.3	5.6	39.7	53.4	37.7	64.6	-14.7	5.2	30.6	-12.8	0.0	0.0	10.9	71.4	92.2	225.1	36.9	78.0	0.5
Phe	-10.1	4.7	164.6	-1.1	57.4	14.0	-7.3	43.6	90.3	148.4	5.7	39.8	53.5	37.7	64.7	-14.7	5.3	30.6	-12.7	0.0	0.0	11.0	71.5	92.3	225.2	37.0	78.0	0.5
Pro	-18.9	-5.6	138.4	-10.8	41.8	2.8	-16.4	29.5	71.6	123.9	-4.8	26.0	38.3	24.1	48.4	-23.1	-5.1	17.7	-21.3	-9.8	-9.9	0.0	54.5	73.3	193.1	23.5	60.5	-9.4
Sar	-47.6	-38.9	54.3	-42.3	-8.2	-33.5	-45.9	-16.2	11.0	44.9	-38.4	-18.5	-10.5	-19.7	-4.0	-50.2	-38.6	-23.8	-49.1	-41.7	-41.7	-35.3	0.0	12.2	89.7	-20.1	3.8	-41.4
Ser	-53.2	-45.6	37.6	-48.6	-18.2	-40.7	-51.8	-25.3	-1.0	29.2	-45.1	-27.3	-20.2	-28.4	-14.4	-55.6	-45.3	-32.1	-54.6	-48.0	-48.0	-42.3	-10.8	0.0	69.1	-28.8	-7.4	-47.7
Tau	-72.3	-67.8	-18.7	-69.6	-51.6	-64.9	-71.5	-55.8	-41.5	-23.6	-67.5	-57.0	-52.8	-57.6	-49.4	-73.8	-67.6	-59.8	-73.2	-69.2	-69.3	-65.9	-47.3	-40.9	0.0	-57.9	-45.3	-69.1
Thr	-34.4	-23.6	93.1	-27.8	14.9	-16.8	-32.3	4.8	38.9	81.3	-22.9	2.0	12.0	0.5	20.2	-37.7	-23.2	-4.6	-36.3	-27.0	-27.0	-19.0	25.2	40.4	137.4	0.0	30.0	-26.6
Tyr	-49.5	-41.2	48.6	-44.4	-11.6	-36.0	-47.9	-19.3	6.9	39.5	-40.7	-21.5	-13.8	-22.6	-7.5	-52.1	-40.9	-26.6	-51.0	-43.8	-43.8	-37.7	-3.7	8.0	82.7	-23.1	0.0	-43.5
Val	-10.5	4.2	163.2	-1.6	56.6	13.4	-7.7	42.9	89.4	147.1	5.1	39.1	52.7	37.0	63.8	-15.1	4.7	30.0	-13.2	-0.5	-0.5	10.4	70.6	91.4	223.6	36.3	77.1	0.0
	Ans	Arg	Asp	Car	Cit	Cre	Cys	Cth	ETA	Glu	AABA	Hcy	His	Hyl	Hyp	Leu-Ile	Lys	Met	3-MH	Orn	Phe	Pro	Sar	Ser	Tau	Thr	Tyr	Val
	**B**																											

Supplementary Table 29*: Raw data Extended Data Fig. 3 – AA in water, 170 µm, 18 h, pH 5.1.*

repeat k	species	measured values (counts*min) for W and Y: mAU*min				measured values (counts*min) for W and Y: mAU*min								concentration (µM) $[A]_{j,k,HPLC}$ calibration acc. to Supplementary Table 4				c_0 (µM)	Normalization (Eq. 1) concentration (x c_0)				Ratio species vs mean (Eq. 4) $[A]_{j,k}/\bar{c}_{j,k}-1$ (%)	
		top	II	III	bot	top	II	III	bot					top	II	III	bot		top	II	III	bot	top	bot
	H	18545	39408	65767	147913	20000	40360	68447	154043					5.84	12.09	20.34	45.76	21.01	0.28	0.58	0.97	2.18	8.8	-7.1
	N	51531	78771	116081	182033	55031	80006	121813	188797					10.50	15.64	23.43	36.53	21.53	0.49	0.73	1.09	1.70	90.7	-27.6
	S	49847	73116	114811	187230	52126	75195	120171	194522					10.35	15.05	23.85	38.75	22.00	0.47	0.68	1.08	1.76	84.0	-24.9
	Q	46514	69925	106850	170929	47987	72085	111472	177674					10.00	15.02	23.09	36.87	21.25	0.47	0.71	1.09	1.74	84.0	-26.0
	R	14681	38761	64098	164343	15811	40109	64714	169066					4.86	12.58	20.55	53.18	22.79	0.21	0.55	0.90	2.33	-16.5	-0.5
	G	84352	108670	133428	194205	87818	112784	140736	201153					15.50	19.93	24.68	35.59	23.92	0.65	0.83	1.03	1.49	153.3	-36.6
	D	12378	16639	68104	166027	12792	18225	72660	172665					2.72	3.77	15.22	36.61	14.58	0.19	0.26	1.04	2.51	-27.0	7.1
	E	9593	15522	61833	165731	10452	15584	64425	170704					2.31	3.59	14.56	38.81	14.82	0.16	0.24	0.98	2.62	-39.0	11.7
	T	24741	49761	100145	221722	26220	51492	104429	232704					4.65	9.25	18.68	41.49	18.52	0.25	0.50	1.01	2.24	-1.7	-4.4
	A	24631	35994	86521	179845	25409	37525	90429	185712					5.26	7.73	18.62	38.46	17.52	0.30	0.44	1.06	2.20	17.5	-6.4
1	P	2857	6713	17871	60777	2817	6927	18726	62720					1.95	4.70	12.60	42.51	15.44	0.13	0.30	0.82	2.75	-50.5	17.4
	C	1761	3664	6792	20444	1926	4012	7250	21634					4.45	9.25	16.93	50.73	20.34	0.22	0.45	0.83	2.49	-14.5	6.4
	K	15885	22234	63453	124871	16042	23353	66771	129334					4.39	6.28	17.93	34.99	15.90	0.28	0.39	1.13	2.20	8.1	-6.1
	M	3728	8775	18316	51112	3619	9326	18770	51678					3.32	8.19	16.77	46.49	18.69	0.18	0.44	0.90	2.49	-30.5	6.1
	V	13938	58133	86295	248674	10856	48709	72214	258459					1.90	8.18	12.14	38.84	15.27	0.12	0.54	0.80	2.54	-51.3	8.5
	I	6224	16647	55420	268875	6462	17765	57229	280683					0.92	2.49	8.15	39.76	12.83	0.07	0.19	0.64	3.10	-72.0	32.2
	L	6285	16957	53062	235890	6861	17881	55658	246001					1.11	2.94	9.17	40.65	13.47	0.08	0.22	0.68	3.02	-67.8	28.7
	F	10346	30555	70232	266225	10233	30257	72720	277102					1.89	5.60	13.16	50.01	17.67	0.11	0.32	0.74	2.83	-58.1	20.8
	Y	0.0763	0.1327	0.1942	0.3758	0.0746	0.1323	0.2052	0.3953					9.40	16.50	24.87	48.01	24.70	0.38	0.67	1.01	1.94	48.8	-17.1
	W	0.0126	0.0482	0.1185	0.3879	0.0122	0.0493	0.1167	0.4052					1.64	6.44	15.54	52.40	19.01	0.09	0.34	0.82	2.76	-66.3	17.6
	mean concentration per fraction $\bar{c}_{j,k}$																		0.26	0.47	0.93	2.34		

2	H	34285	67453	117500	170007	35663	70837	121852	174403					10.60	20.96	36.27	52.19	30.01	0.35	0.70	1.21	1.74	8.0	-7.8
	N	86859	143662	184623	205455	89588	136952	175794	209707					17.38	27.64	35.50	40.90	30.36	0.57	0.91	1.17	1.35	75.0	-28.6
	S	85294	129218	167609	210107	89614	134498	175974	216443					17.75	26.77	34.87	43.29	30.67	0.58	0.87	1.14	1.41	76.9	-25.2
	Q	77320	121113	158657	194225	80537	126619	167261	199336					16.70	26.20	34.47	41.63	29.75	0.56	0.88	1.16	1.40	71.6	-25.8
	R	27331	59009	115767	182316	29056	61699	118912	184874					8.99	19.26	37.44	58.57	31.06	0.29	0.62	1.21	1.89	-11.5	0.0
	G	125026	163567	180125	196571	129480	171727	190485	205679					22.91	30.18	33.36	36.21	30.66	0.75	0.98	1.09	1.18	128.4	-37.4
	D	36768	67800	131427	244802	38295	71164	138058	254111					8.11	15.02	29.13	53.93	26.55	0.31	0.57	1.10	2.03	-6.6	7.7
	E	28810	60510	130789	242979	29088	62447	136842	249848					6.68	14.18	30.87	56.84	27.14	0.25	0.52	1.14	2.09	-24.8	11.0
	T	52364	106614	187587	281897	54097	111270	195844	291237					9.72	19.89	35.01	52.33	29.24	0.33	0.68	1.20	1.79	1.6	-5.1
	A	54045	97431	156783	230354	55816	100782	163309	237999					11.56	20.85	33.67	49.27	28.84	0.40	0.72	1.17	1.71	22.5	-9.4
	P	7445	18901	45970	86578	8060	19952	48172	89319					5.34	13.38	32.41	60.55	27.92	0.19	0.48	1.16	2.17	-41.6	15.0
	C	3035	6507	13355	23771	3084	6863	14011	24710					7.38	16.12	32.99	58.44	28.73	0.26	0.56	1.15	2.03	-21.5	7.8
	K	37399	73137	123146	175651	38747	70755	125519	178843					10.48	19.81	34.23	48.80	28.33	0.37	0.70	1.21	1.72	13.1	-8.7
	M	8743	18457	41216	64192	8525	19108	42082	66908					7.81	16.99	37.67	59.29	30.44	0.26	0.56	1.24	1.95	-21.6	3.3
	V	28429	225816	189762	420295	26592	81268	192062	404877					4.21	23.52	29.24	63.20	30.04	0.14	0.78	0.97	2.10	-57.1	11.5
	I	17337	53985	194205	460461	18558	55679	202339	475909					2.60	7.93	28.69	67.74	26.74	0.10	0.30	1.07	2.53	-70.3	34.3
	L	17610	52072	173563	387889	18512	54239	179910	400513					3.05	8.97	29.81	66.50	27.08	0.11	0.33	1.10	2.46	-65.6	30.2
	F	22615	63471	177428	362751	23774	64250	183183	373589					4.27	11.76	33.19	67.78	29.25	0.15	0.40	1.13	2.32	-55.4	22.8
	Y	0.1137	0.1899	0.2942	0.3982	0.1128	0.196	0.2981	0.4166					14.11	24.03	36.89	50.74	31.44	0.45	0.76	1.17	1.61	37.2	-14.4
	W	0.0279	0.0931	0.2529	0.4754	0.0312	0.0977	0.2631	0.5016					3.90	12.60	34.09	64.56	28.79	0.14	0.44	1.18	2.24	-58.5	18.9
	mean concentration per fraction $\bar{c}_{j,k}$																		0.33	0.64	1.15	1.89		
3	H	41315	69842	104906	167749	43152	73890	107842	175221					12.80	21.78	32.24	51.97	29.70	0.43	0.73	1.09	1.75	7.7	-8.3
	N	93246	130411	149665	206780	96038	137980	153403	215862					18.65	26.44	29.85	41.63	29.14	0.64	0.91	1.02	1.43	59.8	-25.2
	S	91403	132542	148454	208062	95550	139126	151499	218120					18.97	27.57	30.44	43.25	30.06	0.63	0.92	1.01	1.44	57.7	-24.6
	Q	84036	121636	139512	194565	88100	127146	142560	202102					18.21	26.32	29.84	41.96	29.08	0.63	0.90	1.03	1.44	56.4	-24.4
	R	33868	62810	108852	169406	34614	65807	111147	174727					10.92	20.52	35.09	54.90	30.36	0.36	0.68	1.16	1.81	-10.1	-5.3
	G	129461	160204	170642	200048	135683	167805	174816	211306					23.87	29.52	31.09	37.03	30.38	0.79	0.97	1.02	1.22	96.2	-36.2

	D	46744	92424	101010	219393	49017	97225	103649	232268					10.35	20.50	22.12	48.82	25.45	0.41	0.81	0.87	1.92	1.6	0.5
	E	39119	83254	102902	217087	40595	87017	103576	229146					9.19	19.64	23.82	51.47	26.03	0.35	0.75	0.91	1.98	-11.8	3.6
	T	60781	113184	157881	288297	64569	117881	161213	300297					11.45	21.10	29.14	53.74	28.86	0.40	0.73	1.01	1.86	-0.9	-2.5
	A	61868	107344	123710	234669	64420	111990	125045	242302					13.29	23.07	26.17	50.18	28.18	0.47	0.82	0.93	1.78	17.8	-6.7
	P	9576	21671	37811	91220	10054	22726	38023	95477					6.76	15.28	26.11	64.27	28.10	0.24	0.54	0.93	2.29	-39.9	19.8
	C	2778	6276	12060	21720	2940	6550	12550	23208					6.89	15.46	29.67	54.16	26.55	0.26	0.58	1.12	2.04	-35.1	6.9
	K	45515	82199	85095	171841	47481	86160	86471	178664					12.80	23.17	23.62	48.25	26.96	0.47	0.86	0.88	1.79	18.6	-6.3
	M	10463	21318	33352	65321	10911	22047	34258	69152					9.67	19.61	30.58	60.82	30.17	0.32	0.65	1.01	2.02	-20.0	5.6
	V	60301	92615	151285	401063	38373	97547	148925	417360					7.56	14.56	22.99	62.68	26.95	0.28	0.54	0.85	2.33	-30.0	21.8
	I	28318	75119	148160	474224	29304	77532	153087	493525					4.17	11.04	21.79	70.01	26.76	0.16	0.41	0.81	2.62	-61.1	37.1
	L	27858	70111	134470	397121	28535	72880	138331	413616					4.76	12.06	23.01	68.38	27.05	0.18	0.45	0.85	2.53	-56.1	32.4
	F	34437	77671	152260	346105	34331	79242	157348	360004					6.33	14.44	28.50	65.00	28.57	0.22	0.51	1.00	2.28	-44.7	19.2
	Y	0.1279	0.1953	0.2727	0.3763	0.1383	0.2153	0.2843	0.399					16.58	25.57	34.69	48.29	31.28	0.53	0.82	1.11	1.54	32.4	-19.2
	W	0.0514	0.1157	0.2208	0.4468	0.0533	0.1218	0.2293	0.4629					6.92	15.69	29.74	60.11	28.11	0.25	0.56	1.06	2.14	-38.5	12.0
	mean concentration per fraction $\bar{c}_{j,k}$																		0.40	0.71	0.98	1.91		

Supplementary Table 30*: Raw data Extended Data Fig. 3 – AA in water, 170 µm, 18 h, pH 5.1 – enrichments in top fraction*

**repeat**		**species**	Ratio species against species (shown in heat maps), Eq. 2 $[A]_{j,k}/[B]_{j,k} - 1$ (%)																			
1	**A**	H	0.0	-43.0	-40.9	-40.9	30.3	-57.1	49.0	78.2	10.7	-7.5	119.8	27.2	0.6	56.4	123.5	288.7	237.7	159.3	-26.9	222.6
		N	75.4	0.0	3.7	3.7	128.5	-24.7	161.3	212.5	94.1	62.3	285.5	123.1	76.4	174.4	292.0	581.7	492.2	354.8	28.2	465.7
		S	69.2	-3.5	0.0	0.0	120.5	-27.4	152.1	201.5	87.2	56.6	271.9	115.3	70.2	164.7	278.2	557.6	471.3	338.8	23.7	445.8
		Q	69.2	-3.5	0.0	0.0	120.5	-27.4	152.1	201.5	87.2	56.6	271.9	115.3	70.2	164.7	278.2	557.6	471.4	338.8	23.7	445.8
		R	-23.3	-56.2	-54.6	-54.6	0.0	-67.1	14.3	36.8	-15.1	-29.0	68.7	-2.4	-22.8	20.1	71.5	198.3	159.1	99.0	-43.9	147.6
		G	133.0	32.8	37.7	37.7	203.6	0.0	247.1	315.1	157.8	115.6	412.1	196.4	134.3	264.4	420.8	805.5	686.7	504.1	70.3	651.5
		D	-32.9	-61.7	-60.3	-60.3	-12.5	-71.2	0.0	19.6	-25.7	-37.9	47.5	-14.6	-32.5	5.0	50.0	160.9	126.6	74.1	-50.9	116.5
		E	-43.9	-68.0	-66.8	-66.8	-26.9	-75.9	-16.4	0.0	-37.9	-48.1	23.4	-28.6	-43.6	-12.2	25.4	118.1	89.5	45.5	-59.0	81.0
		T	-9.6	-48.5	-46.6	-46.6	17.8	-61.2	34.6	61.0	0.0	-16.4	98.6	15.0	-9.1	41.4	102.0	251.2	205.2	134.4	-34.0	191.5
		A	8.1	-38.4	-36.1	-36.1	40.8	-53.6	61.0	92.6	19.6	0.0	137.6	37.5	8.7	69.1	141.6	320.1	265.0	180.3	-21.0	248.6
		P	-54.5	-74.1	-73.1	-73.1	-40.7	-80.5	-32.2	-18.9	-49.7	-57.9	0.0	-42.1	-54.2	-28.8	1.7	76.8	53.6	18.0	-66.7	46.8
		C	-21.4	-55.2	-53.5	-53.5	2.4	-66.3	17.1	40.1	-13.0	-27.3	72.8	0.0	-20.9	23.0	75.7	205.5	165.4	103.8	-42.6	153.6
		K	-0.6	-43.3	-41.2	-41.2	29.6	-57.3	48.1	77.2	10.0	-8.0	118.5	26.5	0.0	55.5	122.2	286.4	235.7	157.8	-27.3	220.7
		M	-36.1	-63.6	-62.2	-62.2	-16.7	-72.6	-4.8	13.9	-29.3	-40.9	40.5	-18.7	-35.7	0.0	42.9	148.5	115.9	65.8	-53.3	106.2
		V	-55.3	-74.5	-73.6	-73.6	-41.7	-80.8	-33.3	-20.3	-50.5	-58.6	-1.7	-43.1	-55.0	-30.0	0.0	73.9	51.1	16.0	-67.3	44.3
		I	-74.3	-85.3	-84.8	-84.8	-66.5	-89.0	-61.7	-54.2	-71.5	-76.2	-43.4	-67.3	-74.1	-59.8	-42.5	0.0	-13.1	-33.3	-81.2	-17.0
		L	-70.4	-83.1	-82.5	-82.5	-61.4	-87.3	-55.9	-47.2	-67.2	-72.6	-34.9	-62.3	-70.2	-53.7	-33.8	15.1	0.0	-23.2	-78.4	-4.5
		F	-61.4	-78.0	-77.2	-77.2	-49.8	-83.4	-42.5	-31.3	-57.3	-64.3	-15.2	-50.9	-61.2	-39.7	-13.8	49.9	30.2	0.0	-71.8	24.4
		Y	36.8	-22.0	-19.1	-19.1	78.3	-41.3	103.9	143.8	51.4	26.6	200.8	74.1	37.6	114.0	205.8	431.8	362.0	254.8	0.0	341.4
		W	-69.0	-82.3	-81.7	-81.7	-59.6	-86.7	-53.8	-44.8	-65.7	-71.3	-31.9	-60.6	-68.8	-51.5	-30.7	20.5	4.7	-19.6	-77.3	0.0
			H	N	S	Q	R	G	D	E	T	A	P	C	K	M	V	I	L	F	Y	W
			**B**																			
2	**A**	H	0.0	-38.3	-39.0	-37.1	22.0	-52.7	15.6	43.6	6.3	-11.9	84.8	37.6	-4.5	37.7	151.9	263.8	214.0	142.0	-21.3	160.5
		N	62.1	0.0	-1.1	2.0	97.8	-23.4	87.3	132.7	72.2	42.9	199.5	123.0	54.8	123.2	308.2	489.6	409.0	292.2	27.6	322.2
		S	63.8	1.1	0.0	3.1	99.9	-22.5	89.4	135.3	74.1	44.4	202.7	125.5	56.4	125.6	312.7	496.0	414.5	296.5	29.0	326.8
		Q	58.9	-2.0	-3.0	0.0	93.8	-24.9	83.6	128.1	68.8	40.0	193.6	118.6	51.7	118.8	300.1	477.9	398.9	284.4	25.1	313.8
		R	-18.0	-49.4	-50.0	-48.4	0.0	-61.2	-5.3	17.7	-12.9	-27.8	51.4	12.8	-21.7	12.9	106.4	198.2	157.4	98.3	-35.5	113.5
		G	111.5	30.5	29.1	33.1	158.0	0.0	144.4	203.7	124.7	86.4	290.8	191.0	101.9	191.2	432.6	669.3	564.1	411.8	66.5	450.8
		D	-13.5	-46.6	-47.2	-45.5	5.6	-59.1	0.0	24.2	-8.1	-23.7	59.9	19.0	-17.4	19.1	117.9	214.7	171.7	109.4	-31.9	125.3
		E	-30.4	-57.0	-57.5	-56.2	-15.0	-67.1	-19.5	0.0	-26.0	-38.6	28.7	-4.2	-33.5	-4.1	75.4	153.3	118.7	68.5	-45.2	81.4
		T	-5.9	-41.9	-42.6	-40.8	14.8	-55.5	8.8	35.1	0.0	-17.0	73.9	29.5	-10.1	29.6	137.0	242.3	195.5	127.7	-25.9	145.1
		A	13.4	-30.0	-30.8	-28.6	38.4	-46.4	31.1	62.9	20.5	0.0	109.6	56.1	8.3	56.2	185.7	312.7	256.2	174.5	-10.7	195.5
		P	-45.9	-66.6	-67.0	-65.9	-34.0	-74.4	-37.4	-22.3	-42.5	-52.3	0.0	-25.5	-48.3	-25.5	36.3	96.9	69.9	31.0	-57.4	41.0
		C	-27.3	-55.2	-55.6	-54.3	-11.3	-65.6	-16.0	4.3	-22.8	-35.9	34.3	0.0	-30.6	0.1	83.0	164.4	128.2	75.9	-42.8	89.3
		K	4.7	-35.4	-36.1	-34.1	27.8	-50.5	21.1	50.4	11.3	-7.7	93.5	44.1	0.0	44.2	163.8	281.0	228.9	153.5	-17.6	172.8
		M	-27.4	-55.2	-55.7	-54.3	-11.4	-65.7	-16.1	4.3	-22.8	-36.0	34.2	-0.1	-30.7	0.0	82.9	164.2	128.0	75.7	-42.8	89.2
		V	-60.3	-75.5	-75.8	-75.0	-51.6	-81.2	-54.1	-43.0	-57.8	-65.0	-26.6	-45.4	-62.1	-45.3	0.0	44.4	24.7	-3.9	-68.7	3.4
		I	-72.5	-83.0	-83.2	-82.7	-66.5	-87.0	-68.2	-60.5	-70.8	-75.8	-49.2	-62.2	-73.8	-62.1	-30.8	0.0	-13.7	-33.5	-78.4	-28.4

		L	-68.2	-80.4	-80.6	-80.0	-61.1	-84.9	-63.2	-54.3	-66.2	-71.9	-41.2	-56.2	-69.6	-56.1	-19.8	15.8	0.0	-22.9	-74.9	-17.1
		F	-58.7	-74.5	-74.8	-74.0	-49.6	-80.5	-52.2	-40.7	-56.1	-63.6	-23.6	-43.1	-60.5	-43.1	4.1	50.3	29.8	0.0	-67.5	7.6
		Y	27.0	-21.6	-22.5	-20.0	55.0	-39.9	46.8	82.4	35.0	12.0	134.7	74.8	21.3	74.9	219.9	362.1	298.9	207.4	0.0	230.9
		W	-61.6	-76.3	-76.6	-75.8	-53.2	-81.8	-55.6	-44.9	-59.2	-66.2	-29.1	-47.2	-63.3	-47.1	-3.3	39.7	20.6	-7.1	-69.8	0.0
			H	N	S	Q	R	G	D	E	T	A	P	C	K	M	V	I	L	F	Y	W
			**B**																			
3	**A**	H	0.0	-32.6	-31.7	-31.2	19.8	-45.1	6.0	22.0	8.7	-8.6	79.2	66.0	-9.2	34.5	53.7	176.6	145.1	94.5	-18.7	75.2
		N	48.4	0.0	1.4	2.2	77.8	-18.6	57.3	81.1	61.3	35.7	166.1	146.4	34.7	99.7	128.2	310.6	263.9	188.7	20.7	160.0
		S	46.4	-1.3	0.0	0.8	75.4	-19.7	55.2	78.7	59.1	33.9	162.5	143.1	32.9	97.0	125.1	305.1	259.0	184.9	19.1	156.5
		Q	45.3	-2.1	-0.8	0.0	74.0	-20.3	53.9	77.3	57.9	32.8	160.4	141.1	31.9	95.4	123.3	301.9	256.1	182.6	18.1	154.5
		R	-16.5	-43.8	-43.0	-42.5	0.0	-54.2	-11.5	1.9	-9.3	-23.7	49.7	38.6	-24.2	12.3	28.3	131.0	104.7	62.4	-32.1	46.2
		G	82.3	22.8	24.5	25.5	118.3	0.0	93.2	122.4	98.1	66.6	226.7	202.6	65.5	145.2	180.2	404.2	346.8	254.6	48.2	219.3
		D	-5.6	-36.4	-35.6	-35.0	13.0	-48.2	0.0	15.2	2.5	-13.7	69.2	56.6	-14.3	26.9	45.0	161.1	131.3	83.6	-23.3	65.3
		E	-18.0	-44.8	-44.0	-43.6	-1.8	-55.0	-13.2	0.0	-10.9	-25.1	46.9	36.0	-25.6	10.2	26.0	126.7	100.9	59.4	-33.4	43.5
		T	-8.0	-38.0	-37.2	-36.7	10.2	-49.5	-2.5	12.3	0.0	-15.9	65.0	52.8	-16.5	23.8	41.4	154.6	125.6	79.0	-25.2	61.2
		A	9.4	-26.3	-25.3	-24.7	31.0	-40.0	15.9	33.5	18.9	0.0	96.1	81.6	-0.7	47.2	68.1	202.6	168.2	112.8	-11.0	91.6
		P	-44.2	-62.4	-61.9	-61.6	-33.2	-69.4	-40.9	-31.9	-39.4	-49.0	0.0	-7.4	-49.4	-25.0	-14.3	54.3	36.8	8.5	-54.6	-2.3
		C	-39.8	-59.4	-58.9	-58.5	-27.8	-66.9	-36.2	-26.5	-34.5	-44.9	8.0	0.0	-45.3	-19.0	-7.4	66.7	47.7	17.2	-51.0	5.5
		K	10.2	-25.8	-24.8	-24.2	31.9	-39.6	16.7	34.4	19.7	0.7	97.5	82.9	0.0	48.2	69.3	204.7	170.0	114.3	-10.4	93.0
		M	-25.7	-49.9	-49.2	-48.8	-11.0	-59.2	-21.2	-9.3	-19.2	-32.0	33.3	23.4	-32.5	0.0	14.3	105.6	82.2	44.6	-39.5	30.2
		V	-34.9	-56.2	-55.6	-55.2	-22.1	-64.3	-31.1	-20.6	-29.3	-40.5	16.6	8.0	-40.9	-12.5	0.0	80.0	59.5	26.6	-47.1	14.0
		I	-63.8	-75.6	-75.3	-75.1	-56.7	-80.2	-61.7	-55.9	-60.7	-67.0	-35.2	-40.0	-67.2	-51.4	-44.4	0.0	-11.4	-29.7	-70.6	-36.7
		L	-59.2	-72.5	-72.1	-71.9	-51.1	-77.6	-56.8	-50.2	-55.7	-62.7	-26.9	-32.3	-63.0	-45.1	-37.3	12.8	0.0	-20.6	-66.8	-28.5
		F	-48.6	-65.4	-64.9	-64.6	-38.4	-71.8	-45.5	-37.3	-44.1	-53.0	-7.8	-14.7	-53.3	-30.8	-21.0	42.2	26.0	0.0	-58.2	-10.0
		Y	23.0	-17.2	-16.0	-15.4	47.3	-32.5	30.3	50.0	33.6	12.4	120.4	104.1	11.6	65.4	89.0	240.2	201.4	139.2	0.0	115.4
		W	-42.9	-61.5	-61.0	-60.7	-31.6	-68.7	-39.5	-30.3	-38.0	-47.8	2.3	-5.2	-48.2	-23.2	-12.3	57.9	40.0	11.1	-53.6	0.0
			H	N	S	Q	R	G	D	E	T	A	P	C	K	M	V	I	L	F	Y	W
			**B**																			

***Supplementary Table 31**: Raw data Extended Data Fig. 3 – AA in water, 170 µm, 18 h, pH 5.1 – enrichments in bottom fraction*

repeat		species	Ratio species against species (shown in heat maps), Eq. 2 $[A]_{j,k}/[B]_{j,k} - 1$ (%)																			
1	A	H	0.0	28.4	23.7	25.5	-6.6	46.4	-13.3	-16.8	-2.8	-0.8	-20.9	-12.7	-1.0	-12.4	-14.4	-29.7	-27.8	-23.1	12.0	-21.0
		N	-22.1	0.0	-3.6	-2.2	-27.3	14.1	-32.4	-35.2	-24.3	-22.7	-38.4	-32.0	-22.9	-31.8	-33.3	-45.2	-43.8	-40.1	-12.7	-38.4
		S	-19.1	3.8	0.0	1.5	-24.5	18.4	-29.9	-32.8	-21.4	-19.8	-36.0	-29.4	-20.0	-29.2	-30.8	-43.2	-41.7	-37.8	-9.4	-36.1
		Q	-20.3	2.3	-1.5	0.0	-25.6	16.7	-30.9	-33.7	-22.5	-20.9	-37.0	-30.4	-21.2	-30.2	-31.8	-44.0	-42.5	-38.7	-10.7	-37.1
		R	7.1	37.5	32.5	34.4	0.0	56.9	-7.1	-10.9	4.1	6.3	-15.3	-6.5	6.0	-6.2	-8.3	-24.7	-22.7	-17.6	20.0	-15.4
		G	-31.7	-12.3	-15.5	-14.3	-36.2	0.0	-40.8	-43.2	-33.6	-32.2	-46.0	-40.4	-32.4	-40.2	-41.5	-52.0	-50.7	-47.5	-23.5	-46.0
		D	15.3	48.0	42.6	44.7	7.6	68.8	0.0	-4.1	12.1	14.4	-8.8	0.7	14.1	1.0	-1.3	-19.0	-16.8	-11.3	29.2	-8.9
		E	20.2	54.3	48.7	50.9	12.2	76.1	4.3	0.0	16.9	19.3	-4.9	5.0	19.0	5.3	2.9	-15.5	-13.2	-7.5	34.7	-5.0
		T	2.9	32.0	27.2	29.1	-4.0	50.6	-10.8	-14.4	0.0	2.1	-18.6	-10.2	1.8	-9.9	-11.9	-27.7	-25.8	-20.9	15.2	-18.7
		A	0.8	29.4	24.6	26.5	-5.9	47.6	-12.6	-16.2	-2.0	0.0	-20.3	-12.0	-0.3	-11.7	-13.7	-29.2	-27.3	-22.5	12.9	-20.4
		P	26.4	62.3	56.3	58.6	18.0	85.1	9.6	5.1	22.9	25.4	0.0	10.4	25.1	10.7	8.2	-11.2	-8.8	-2.7	41.6	-0.1
		C	14.5	47.0	41.6	43.7	6.9	67.7	-0.7	-4.8	11.3	13.6	-9.4	0.0	13.3	0.3	-2.0	-19.5	-17.4	-11.9	28.3	-9.5
		K	1.1	29.7	25.0	26.8	-5.7	48.0	-12.3	-16.0	-1.8	0.3	-20.1	-11.7	0.0	-11.5	-13.5	-29.0	-27.1	-22.2	13.2	-20.2
		M	14.2	46.5	41.2	43.3	6.6	67.2	-1.0	-5.0	11.0	13.3	-9.7	-0.3	13.0	0.0	-2.3	-19.8	-17.6	-12.2	27.9	-9.8
		V	16.8	49.9	44.5	46.6	9.0	71.0	1.3	-2.8	13.5	15.9	-7.6	2.0	15.6	2.3	0.0	-17.9	-15.7	-10.1	30.9	-7.7
		I	42.3	82.6	76.0	78.6	32.8	108.3	23.4	18.3	38.3	41.2	12.6	24.3	40.8	24.6	21.8	0.0	2.7	9.5	59.4	12.4
		L	38.6	77.9	71.4	73.9	29.4	102.9	20.2	15.3	34.7	37.5	9.6	21.0	37.1	21.4	18.6	-2.6	0.0	6.6	55.2	9.5
		F	30.0	66.8	60.7	63.1	21.3	90.3	12.7	8.1	26.3	29.0	2.8	13.5	28.6	13.8	11.3	-8.7	-6.2	0.0	45.6	2.7
		Y	-10.7	14.6	10.4	12.0	-16.7	30.7	-22.6	-25.8	-13.2	-11.4	-29.4	-22.0	-11.7	-21.8	-23.6	-37.3	-35.6	-31.3	0.0	-29.5
		W	26.6	62.5	56.5	58.9	18.2	85.4	9.8	5.3	23.0	25.6	0.1	10.5	25.3	10.9	8.4	-11.0	-8.7	-2.6	41.8	0.0
			H	N	S	Q	R	G	D	E	T	A	P	C	K	M	V	I	L	F	Y	W
			**B**																			
2	A	H	0.0	29.1	23.2	24.3	-7.7	47.3	-14.4	-16.9	-2.8	1.8	-19.8	-14.5	1.0	-10.7	-17.3	-31.3	-29.2	-24.9	7.8	-22.4
		N	-22.5	0.0	-4.6	-3.7	-28.5	14.1	-33.7	-35.7	-24.7	-21.1	-37.9	-33.8	-21.8	-30.8	-36.0	-46.8	-45.1	-41.9	-16.5	-39.9
		S	-18.9	4.8	0.0	0.9	-25.1	19.5	-30.5	-32.6	-21.1	-17.4	-34.9	-30.6	-18.1	-27.5	-32.9	-44.3	-42.5	-39.1	-12.5	-37.1
		Q	-19.6	3.9	-0.9	0.0	-25.8	18.5	-31.1	-33.2	-21.8	-18.1	-35.5	-31.2	-18.8	-28.2	-33.5	-44.8	-43.0	-39.6	-13.3	-37.6
		R	8.4	40.0	33.6	34.8	0.0	59.7	-7.2	-10.0	5.4	10.4	-13.1	-7.3	9.5	-3.2	-10.4	-25.6	-23.2	-18.6	16.8	-15.9
		G	-32.1	-12.4	-16.3	-15.6	-37.4	0.0	-41.9	-43.6	-34.0	-30.9	-45.6	-42.0	-31.5	-39.4	-43.9	-53.4	-51.9	-49.0	-26.8	-47.3
		D	16.8	50.8	43.9	45.2	7.7	72.0	0.0	-3.0	13.5	18.9	-6.3	-0.1	17.9	4.3	-3.4	-19.8	-17.3	-12.3	25.9	-9.4
		E	20.4	55.4	48.4	49.7	11.1	77.4	3.1	0.0	17.0	22.6	-3.4	3.0	21.6	7.5	-0.4	-17.3	-14.7	-9.6	29.8	-6.6
		T	2.9	32.8	26.8	27.9	-5.1	51.6	-11.9	-14.5	0.0	4.8	-17.5	-12.0	3.9	-8.1	-14.9	-29.4	-27.1	-22.8	10.9	-20.2
		A	-1.8	26.8	21.0	22.1	-9.4	44.7	-15.9	-18.4	-4.5	0.0	-21.2	-16.0	-0.8	-12.3	-18.8	-32.6	-30.4	-26.3	5.9	-23.8
		P	24.7	61.0	53.7	55.0	15.0	83.7	6.8	3.6	21.2	26.9	0.0	6.6	25.9	11.4	3.1	-14.4	-11.7	-6.4	34.4	-3.3
		C	16.9	51.0	44.1	45.4	7.9	72.3	0.1	-2.9	13.7	19.1	-6.2	0.0	18.1	4.4	-3.3	-19.7	-17.2	-12.2	26.0	-9.3
		K	-1.0	27.9	22.0	23.1	-8.6	45.9	-15.2	-17.7	-3.8	0.8	-20.6	-15.3	0.0	-11.6	-18.1	-32.0	-29.9	-25.7	6.7	-23.2
		M	12.0	44.6	38.0	39.2	3.3	65.0	-4.1	-7.0	8.8	14.0	-10.2	-4.2	13.1	0.0	-7.4	-23.1	-20.7	-15.9	20.7	-13.1
		V	20.9	56.1	49.0	50.3	11.6	78.1	3.6	0.4	17.5	23.1	-3.0	3.4	22.1	8.0	0.0	-17.0	-14.3	-9.2	30.4	-6.2
		I	45.6	88.0	79.5	81.1	34.4	114.5	24.7	21.0	41.5	48.3	16.8	24.5	47.1	30.1	20.4	0.0	3.2	9.3	57.0	13.0

		L	41.2	82.3	74.0	75.5	30.2	108.0	20.9	17.3	37.2	43.7	13.2	20.7	42.6	26.1	16.7	-3.1	0.0	6.0	52.2	9.5
		F	33.2	72.0	64.2	65.6	22.9	96.2	14.1	10.7	29.5	35.6	6.8	13.9	34.5	19.0	10.2	-8.5	-5.6	0.0	43.6	3.3
		Y	-7.2	19.8	14.3	15.3	-14.4	36.7	-20.6	-22.9	-9.8	-5.5	-25.6	-20.7	-6.3	-17.1	-23.3	-36.3	-34.3	-30.4	0.0	-28.0
		W	28.9	66.4	58.9	60.3	18.9	89.9	10.4	7.1	25.3	31.2	3.4	10.2	30.2	15.1	6.6	-11.5	-8.7	-3.2	39.0	0.0
			H	N	S	Q	R	G	D	E	T	A	P	C	K	M	V	I	L	F	Y	W
			**B**																			
		H	0.0	22.5	21.6	21.3	-3.2	43.6	-8.8	-11.5	-6.0	-1.7	-23.5	-14.2	-2.2	-13.2	-24.8	-33.1	-30.8	-23.1	13.4	-18.2
		N	-18.4	0.0	-0.7	-1.0	-21.0	17.2	-25.5	-27.8	-23.3	-19.8	-37.5	-30.0	-20.2	-29.1	-38.6	-45.4	-43.5	-37.2	-7.5	-33.2
		S	-17.8	0.7	0.0	-0.3	-20.4	18.1	-25.0	-27.2	-22.7	-19.2	-37.1	-29.5	-19.6	-28.6	-38.1	-45.0	-43.1	-36.8	-6.8	-32.7
		Q	-17.6	1.0	0.3	0.0	-20.2	18.4	-24.8	-27.0	-22.5	-19.0	-36.9	-29.3	-19.4	-28.4	-38.0	-44.9	-42.9	-36.6	-6.5	-32.5
		R	3.3	26.6	25.7	25.3	0.0	48.4	-5.7	-8.5	-2.9	1.5	-20.9	-11.4	1.0	-10.3	-22.3	-30.9	-28.5	-20.5	17.1	-15.4
		G	-30.4	-14.7	-15.3	-15.5	-32.6	0.0	-36.5	-38.4	-34.6	-31.6	-46.7	-40.3	-31.9	-39.5	-47.6	-53.4	-51.8	-46.4	-21.0	-43.0
		D	9.6	34.3	33.3	33.0	6.1	57.4	0.0	-3.0	3.0	7.7	-16.1	-6.0	7.2	-4.8	-17.5	-26.7	-24.1	-15.7	24.3	-10.3
		E	13.0	38.4	37.4	37.0	9.3	62.2	3.1	0.0	6.2	11.0	-13.5	-3.1	10.5	-1.9	-15.0	-24.4	-21.8	-13.1	28.1	-7.5
		T	6.4	30.4	29.4	29.1	3.0	52.8	-2.9	-5.8	0.0	4.6	-18.6	-8.7	4.1	-7.6	-19.9	-28.8	-26.3	-18.1	20.7	-12.9
	**A**	A	1.8	24.7	23.8	23.4	-1.5	46.1	-7.2	-9.9	-4.4	0.0	-22.1	-12.7	-0.5	-11.7	-23.4	-31.9	-29.5	-21.7	15.4	-16.7
3		P	30.7	60.1	58.9	58.5	26.5	87.6	19.2	15.7	22.8	28.4	0.0	12.1	27.8	13.4	-1.7	-12.6	-9.5	0.5	48.1	7.0
		C	16.6	42.8	41.8	41.4	12.8	67.4	6.3	3.2	9.5	14.6	-10.8	0.0	14.0	1.2	-12.3	-22.0	-19.3	-10.3	32.2	-4.6
		K	2.3	25.3	24.4	24.0	-1.0	46.8	-6.7	-9.5	-3.9	0.5	-21.7	-12.3	0.0	-11.2	-23.1	-31.6	-29.2	-21.3	15.9	-16.3
		M	15.2	41.1	40.1	39.7	11.5	65.4	5.1	2.0	8.2	13.2	-11.8	-1.2	12.6	0.0	-13.3	-23.0	-20.2	-11.4	30.6	-5.7
		V	32.9	62.8	61.6	61.2	28.6	90.8	21.2	17.6	24.9	30.6	1.7	14.0	30.0	15.4	0.0	-11.1	-8.0	2.2	50.7	8.8
		I	49.5	83.2	81.9	81.4	44.7	114.7	36.4	32.3	40.5	46.9	14.4	28.3	46.2	29.8	12.5	0.0	3.5	15.0	69.5	22.4
		L	44.4	76.9	75.7	75.2	39.8	107.4	31.8	27.8	35.7	41.9	10.5	23.9	41.2	25.4	8.7	-3.4	0.0	11.1	63.8	18.2
		F	30.0	59.3	58.1	57.7	25.8	86.7	18.6	15.1	22.2	27.8	-0.5	11.5	27.1	12.9	-2.2	-13.1	-10.0	0.0	47.4	6.4
		Y	-11.8	8.1	7.3	7.0	-14.6	26.6	-19.5	-21.9	-17.1	-13.3	-32.5	-24.3	-13.7	-23.4	-33.6	-41.0	-38.9	-32.2	0.0	-27.8
		W	22.2	49.7	48.6	48.2	18.2	75.4	11.5	8.1	14.8	20.1	-6.5	4.8	19.5	6.1	-8.1	-18.3	-15.4	-6.0	38.5	0.0
			H	N	S	Q	R	G	D	E	T	A	P	C	K	M	V	I	L	F	Y	W
			**B**																			

Supplementary Table 32*: Raw data Extended Data Fig. 3 – AA in water, 170 µm, 18 h, pH 9.9*

repeat k	species	measured values (counts*min) for W and Y: mAU*min				measured values (counts*min) for W and Y: mAU*min								concentration (µM) $[A]_{j,k,HPLC}$ calibration acc. to Supplementary Table 4				c_0 (µM)	Normalization (Eq. 1) concentration (x c_0)				Ratio species vs mean (Eq. 4) $[A]_{j,k}/\bar{c}_{j,k}-1$ (%)	
		top	II	III	bot	top	II	III	bot					top	II	III	bot		top	II	III	bot	top	bot
	H	37492	59852	73093	142311	38272	63576	76448	145926					11.48	18.70	22.66	43.68	24.13	0.48	0.78	0.94	1.81	12.3	-8.2
	N	65623	95851	118575	211834	67480	100571	124997	217910					13.11	19.35	23.99	42.33	24.70	0.53	0.78	0.97	1.71	25.3	-13.0
	S	69445	97850	126756	209982	72308	102887	133228	216011					14.39	20.37	26.39	43.24	26.10	0.55	0.78	1.01	1.66	30.1	-16.0
	Q	61819	89919	111376	179279	64020	94601	115498	185135					13.31	19.52	24.00	38.55	23.84	0.56	0.82	1.01	1.62	31.8	-18.0
	R	33445	59857	70928	130058	34692	62995	73413	135572					10.87	19.60	23.03	42.37	23.97	0.45	0.82	0.96	1.77	7.0	-10.3
	G	90651	116019	148208	202637	93530	122986	154348	208261					16.58	21.51	27.23	36.98	25.58	0.65	0.84	1.06	1.45	53.0	-26.6
	D	41931	63302	87320	191248	43126	67317	91591	197930					9.19	14.12	19.34	42.07	21.18	0.43	0.67	0.91	1.99	2.5	0.8
	E	37112	60696	82022	197224	37232	63745	84386	203918					8.58	14.35	19.19	46.27	22.10	0.39	0.65	0.87	2.09	-8.4	6.2
	T	51817	88437	113656	254438	53627	94363	117726	261581					9.63	16.69	21.13	47.12	23.64	0.41	0.71	0.89	1.99	-3.9	1.1
	A	54282	82287	112880	211861	56579	87290	117820	220064					11.66	17.84	24.27	45.44	24.80	0.47	0.72	0.98	1.83	11.0	-7.1
1	P	10506	20339	27124	67706	10575	21267	28185	70090					7.26	14.32	19.04	47.44	22.01	0.33	0.65	0.86	2.15	-22.2	9.3
	C	2805	4977	6206	17912	2695	5314	6297	18626					6.63	12.41	15.07	44.05	19.54	0.34	0.63	0.77	2.25	-19.9	14.4
	K	43835	69207	90837	157984	45085	72373	93525	162801					12.24	19.49	25.38	44.16	25.32	0.48	0.77	1.00	1.74	14.1	-11.5
	M	10219	17925	22336	56654	9793	19354	24198	58256					9.05	16.86	21.05	51.97	24.73	0.37	0.68	0.85	2.10	-13.6	6.6
	V	47338	140994	119511	314073	42699	83666	104218	312413					6.90	17.21	17.13	47.98	22.30	0.31	0.77	0.77	2.15	-27.0	9.1
	I	36633	73754	96338	330909	37519	78440	101229	344907					5.36	11.01	14.29	48.89	19.89	0.27	0.55	0.72	2.46	-36.3	24.7
	L	34019	67312	88431	284531	35763	71992	92438	296696					5.89	11.75	15.26	49.02	20.48	0.29	0.57	0.74	2.39	-32.2	21.4
	F	36224	70964	87733	256828	38412	75078	91724	266717					6.87	13.44	16.52	48.19	21.26	0.32	0.63	0.78	2.27	-23.7	15.0
	Y	0.0906	0.1394	0.1702	0.3201	0.1006	0.1477	0.1739	0.3347					11.90	17.88	21.43	40.78	23.00	0.52	0.78	0.93	1.77	22.2	-10.0
	W	0.0536	0.105	0.1167	0.3352	0.0476	0.1088	0.1162	0.3419					6.69	14.12	15.38	44.74	20.23	0.33	0.70	0.76	2.21	-22.0	12.2
	mean concentration per fraction $\bar{c}_{j,k}$																		0.42	0.72	0.89	1.97		
2	H	26452	43548	79378	219504	27531	46968	83139	232566					8.18	13.72	24.63	68.51	28.76	0.28	0.48	0.86	2.38	23.6	-8.5

	N	53751	76085	123718	307981	56404	82111	128917	328618					10.85	15.58	24.89	62.71	28.51	0.38	0.55	0.87	2.20	65.3	-15.5
	S	55133	77863	130425	302309	57773	83916	135485	320108					11.46	16.42	26.99	63.17	29.51	0.39	0.56	0.91	2.14	68.7	-17.8
	Q	51652	73095	117895	258981	53856	78751	122066	275034					11.16	16.06	25.38	56.49	27.27	0.41	0.59	0.93	2.07	77.8	-20.5
	R	22800	40731	78742	201740	23499	43574	79512	213402					7.39	13.45	25.24	66.22	28.08	0.26	0.48	0.90	2.36	14.3	-9.4
	G	88125	102971	144431	268347	91959	111298	153272	283416					16.21	19.29	26.80	49.66	27.99	0.58	0.69	0.96	1.77	151.6	-31.9
	D	22103	50792	99965	353997	23249	54359	103389	376757					4.90	11.37	21.98	78.99	29.31	0.17	0.39	0.75	2.69	-27.3	3.5
	E	18390	42852	92600	363456	18214	45735	95980	385401					4.22	10.22	21.75	86.38	30.64	0.14	0.33	0.71	2.82	-40.1	8.3
	T	32632	60019	120071	409298	34964	64875	124680	433185					6.17	11.40	22.35	76.92	29.21	0.21	0.39	0.77	2.63	-8.2	1.1
	A	36870	64968	120582	342103	37359	68687	125358	362035					7.81	14.06	25.87	74.08	30.45	0.26	0.46	0.85	2.43	11.4	-6.6
	P	5412	12433	28539	118634	5650	13772	30113	125372					3.81	9.02	20.19	84.00	29.25	0.13	0.31	0.69	2.87	-43.5	10.3
	C	2026	4012	7053	33856	2166	3791	8153	35521					5.05	9.41	18.33	83.63	29.11	0.17	0.32	0.63	2.87	-24.6	10.3
	K	26956	53268	97890	255332	28783	57550	102048	268253					7.67	15.25	27.52	72.07	30.63	0.25	0.50	0.90	2.35	8.8	-9.6
	M	5512	10736	25209	93352	5393	11911	26216	98650					4.93	10.24	23.26	86.84	31.32	0.16	0.33	0.74	2.77	-31.6	6.5
	V	24662	55808	114162	567852	18642	45926	112362	580462					3.32	7.79	17.35	87.94	29.10	0.11	0.27	0.60	3.02	-50.5	16.1
	I	14337	37217	99120	632625	14849	40128	103934	669141					2.11	5.60	14.69	94.18	29.14	0.07	0.19	0.50	3.23	-68.5	24.1
	L	13842	35942	92308	531197	14941	38626	96472	561922					2.43	6.29	15.92	92.20	29.21	0.08	0.22	0.55	3.16	-63.9	21.2
	F	16961	39889	93293	449330	17328	41961	97220	476673					3.16	7.53	17.54	85.24	28.37	0.11	0.27	0.62	3.00	-51.7	15.4
	Y	0.0742	0.1091	0.1758	0.5142	0.0763	0.1241	0.1861	0.554					9.37	14.52	22.54	66.52	28.24	0.33	0.51	0.80	2.36	44.2	-9.5
	W	0.0224	0.0554	0.135	0.5669	0.0182	0.0548	0.1387	0.5966					2.68	7.28	18.09	76.87	26.23	0.10	0.28	0.69	2.93	-55.6	12.5
	mean concentration per fraction $\bar{c}_{j,k}$																		0.23	0.40	0.76	2.60		
	H	34494	39844	68474	235800	36210	45041	71682	246713					10.71	12.86	21.24	73.12	29.48	0.36	0.44	0.72	2.48	13.2	-7.6
	N	64529	72855	114305	333672	67456	81087	118500	349564					13.00	15.16	22.93	67.30	29.60	0.44	0.51	0.77	2.27	36.8	-15.3
	S	71312	90294	125700	329255	74530	100022	130708	345425					14.80	19.32	26.02	68.48	32.15	0.46	0.60	0.81	2.13	43.4	-20.7
3	Q	62672	70492	108322	279301	65049	78851	112546	291611					13.51	15.80	23.36	60.39	28.26	0.48	0.56	0.83	2.14	48.9	-20.4
	R	31520	37752	64539	216413	31884	41306	68307	226565					10.11	12.61	21.19	70.66	28.64	0.35	0.44	0.74	2.47	10.0	-8.1
	G	102346	111488	151898	281807	108957	125469	158624	295190					19.02	21.33	27.95	51.93	30.06	0.63	0.71	0.93	1.73	97.1	-35.6
	D	39932	46118	86351	399920	42548	51152	91199	418894					8.92	10.51	19.19	88.51	31.78	0.28	0.33	0.60	2.78	-12.6	3.7

	E	32791	38280	71754	401268	33849	43525	75497	415750					7.69	9.44	16.98	94.24	32.09	0.24	0.29	0.53	2.94	-25.4	9.4
	T	46965	56675	98646	430927	49671	63279	104178	453135					8.82	10.95	18.52	80.72	29.75	0.30	0.37	0.62	2.71	-7.6	1.1
	A	55221	63125	108144	357795	57523	70760	112719	376558					11.86	14.08	23.24	77.25	31.61	0.38	0.45	0.74	2.44	16.9	-8.9
	P	10365	11916	23236	120460	10852	14185	24571	125683					7.30	8.99	16.46	84.73	29.37	0.25	0.31	0.56	2.89	-22.5	7.5
	C	2705	3393	5601	37352	2764	3780	6001	38803					6.59	8.65	13.99	91.81	30.26	0.22	0.29	0.46	3.03	-32.1	13.0
	K	41737	48859	86531	282540	43613	53864	89270	296740					11.75	14.14	24.20	79.74	32.46	0.36	0.44	0.75	2.46	12.8	-8.5
	M	7918	10078	18067	98478	8681	11643	20401	102919					7.51	9.82	17.40	91.09	31.45	0.24	0.31	0.55	2.90	-25.6	7.9
	V	75755	46090	83329	556949	69501	49716	83031	582547					11.12	7.34	12.74	87.27	29.62	0.38	0.25	0.43	2.95	17.0	9.8
	I	30683	37717	69291	640996	31639	41747	72328	667345					4.51	5.75	10.25	94.66	28.79	0.16	0.20	0.36	3.29	-51.2	22.5
	L	28782	35701	64836	543813	30501	39286	67828	565376					5.00	6.32	11.19	93.56	29.02	0.17	0.22	0.39	3.22	-46.3	20.1
	F	30483	36310	65805	469934	32236	40969	68734	489885					5.77	7.11	12.38	88.35	28.41	0.20	0.25	0.44	3.11	-36.7	15.9
	Y	0.0962	0.1155	0.1653	0.6948	0.1017	0.1234	0.1787	0.7349					12.33	14.88	21.42	89.03	34.41	0.36	0.43	0.62	2.59	11.6	-3.6
	W	0.0331	0.0378	0.0898	0.605	0.033	0.044	0.0903	0.6387					4.37	5.40	11.90	82.18	25.96	0.17	0.21	0.46	3.17	-47.6	17.9
	mean concentration per fraction $\bar{c}_{j,k}$																		0.32	0.38	0.62	2.68		

Supplementary Table 33*: Raw data Extended Data Fig. 3 – AA in water, 170 µm, 18 h, pH 9.9 – enrichments in top fraction*

**repeat**		**species**	Ratio species against species (shown in heat maps), Eq. 2 $[A]_{j,k}/[B]_{j,k} - 1$ (%)																			
1	**A**	H	0.0	-10.4	-13.7	-14.8	4.9	-26.6	9.6	22.6	16.8	1.2	44.3	40.2	-1.6	30.0	53.9	76.4	65.5	47.2	-8.1	43.9
		N	11.6	0.0	-3.7	-4.9	17.1	-18.1	22.3	36.8	30.4	12.9	61.0	56.5	9.8	45.1	71.7	96.8	84.7	64.3	2.6	60.6
		S	15.9	3.8	0.0	-1.2	21.6	-14.9	27.0	42.1	35.4	17.2	67.2	62.5	14.0	50.6	78.3	104.4	91.8	70.6	6.5	66.8
		Q	17.3	5.2	1.3	0.0	23.1	-13.9	28.6	43.9	37.1	18.7	69.3	64.5	15.5	52.5	80.6	107.0	94.2	72.7	7.9	68.9
		R	-4.7	-14.6	-17.7	-18.8	0.0	-30.0	4.5	16.9	11.4	-3.6	37.6	33.7	-6.2	23.9	46.7	68.2	57.8	40.3	-12.4	37.2
		G	36.2	22.1	17.6	16.1	42.9	0.0	49.3	67.0	59.2	37.8	96.6	91.0	34.1	77.1	109.6	140.3	125.5	100.5	25.2	96.1
		D	-8.8	-18.2	-21.3	-22.2	-4.3	-33.0	0.0	11.9	6.6	-7.7	31.7	27.9	-10.2	18.6	40.4	60.9	51.0	34.3	-16.1	31.3
		E	-18.4	-26.9	-29.6	-30.5	-14.4	-40.1	-10.6	0.0	-4.7	-17.5	17.7	14.4	-19.7	6.0	25.5	43.9	35.0	20.1	-25.0	17.4
		T	-14.4	-23.3	-26.1	-27.0	-10.2	-37.2	-6.2	4.9	0.0	-13.4	23.5	20.0	-15.8	11.3	31.7	51.0	41.7	26.0	-21.3	23.2
		A	-1.2	-11.4	-14.7	-15.8	3.7	-27.5	8.3	21.2	15.5	0.0	42.6	38.6	-2.7	28.5	52.1	74.3	63.6	45.5	-9.2	42.2
		P	-30.7	-37.9	-40.2	-40.9	-27.3	-49.1	-24.1	-15.0	-19.1	-29.9	0.0	-2.9	-31.8	-9.9	6.6	22.2	14.7	2.0	-36.3	-0.3
		C	-28.7	-36.1	-38.5	-39.2	-25.2	-47.6	-21.8	-12.6	-16.7	-27.8	2.9	0.0	-29.8	-7.3	9.8	25.8	18.1	5.0	-34.4	2.6
		K	1.6	-8.9	-12.3	-13.4	6.6	-25.4	11.4	24.6	18.7	2.8	46.7	42.5	0.0	32.1	56.4	79.3	68.2	49.6	-6.6	46.3
		M	-23.1	-31.1	-33.6	-34.4	-19.3	-43.5	-15.7	-5.7	-10.1	-22.2	11.0	7.8	-24.3	0.0	18.4	35.7	27.3	13.2	-29.3	10.7
		V	-35.0	-41.8	-43.9	-44.6	-31.8	-52.3	-28.8	-20.3	-24.1	-34.2	-6.2	-8.9	-36.1	-15.5	0.0	14.6	7.6	-4.3	-40.3	-6.5
		I	-43.3	-49.2	-51.1	-51.7	-40.5	-58.4	-37.9	-30.5	-33.8	-42.6	-18.2	-20.5	-44.2	-26.3	-12.8	0.0	-6.2	-16.6	-47.9	-18.4
		L	-39.6	-45.9	-47.9	-48.5	-36.6	-55.7	-33.8	-25.9	-29.4	-38.9	-12.8	-15.3	-40.6	-21.5	-7.0	6.6	0.0	-11.1	-44.5	-13.1
		F	-32.1	-39.1	-41.4	-42.1	-28.7	-50.1	-25.5	-16.7	-20.6	-31.3	-2.0	-4.8	-33.2	-11.7	4.5	19.8	12.5	0.0	-37.6	-2.2
		Y	8.8	-2.5	-6.1	-7.3	14.1	-20.1	19.2	33.4	27.1	10.1	57.0	52.5	7.1	41.4	67.4	91.9	80.1	60.1	0.0	56.6
		W	-30.5	-37.7	-40.0	-40.8	-27.1	-49.0	-23.8	-14.8	-18.8	-29.7	0.3	-2.6	-31.6	-9.7	6.9	22.6	15.0	2.3	-36.1	0.0
			H	N	S	Q	R	G	D	E	T	A	P	C	K	M	V	I	L	F	Y	W
			**B**																			
2	**A**	H	0.0	-25.3	-26.7	-30.5	8.1	-50.9	70.1	106.5	34.6	10.9	118.5	63.9	13.6	80.6	149.6	292.6	242.3	155.6	-14.3	178.2
		N	33.8	0.0	-2.0	-7.0	44.7	-34.3	127.6	176.3	80.2	48.4	192.4	119.2	52.0	141.7	234.0	425.4	358.0	242.1	14.7	272.2
		S	36.5	2.0	0.0	-5.1	47.6	-32.9	132.2	181.8	83.8	51.4	198.3	123.7	55.0	146.6	240.7	436.0	367.2	249.0	17.0	279.8
		Q	43.9	7.5	5.4	0.0	55.6	-29.3	144.7	197.0	93.7	59.6	214.4	135.7	63.4	159.8	259.1	464.8	392.4	267.8	23.3	300.2
		R	-7.5	-30.9	-32.3	-35.7	0.0	-54.6	57.3	90.9	24.5	2.6	102.1	51.5	5.0	67.0	130.8	263.1	216.5	136.4	-20.7	157.3
		G	103.6	52.1	49.1	41.5	120.2	0.0	246.3	320.3	174.1	125.9	344.9	233.6	131.2	267.7	408.2	699.3	596.8	420.5	74.5	466.4
		D	-41.2	-56.1	-56.9	-59.1	-36.4	-71.1	0.0	21.4	-20.8	-34.8	28.5	-3.7	-33.2	6.2	46.8	130.8	101.2	50.3	-49.6	63.6
		E	-51.6	-63.8	-64.5	-66.3	-47.6	-76.2	-17.6	0.0	-34.8	-46.3	5.8	-20.6	-45.0	-12.5	20.9	90.2	65.8	23.8	-58.5	34.7
		T	-25.7	-44.5	-45.6	-48.4	-19.7	-63.5	26.3	53.3	0.0	-17.6	62.3	21.7	-15.7	34.2	85.4	191.6	154.2	89.9	-36.3	106.6
		A	-9.9	-32.6	-34.0	-37.3	-2.5	-55.7	53.3	86.1	21.4	0.0	97.0	47.7	2.4	62.8	125.0	253.9	208.5	130.4	-22.7	150.8
		P	-54.2	-65.8	-66.5	-68.2	-50.5	-77.5	-22.2	-5.5	-38.4	-49.2	0.0	-25.0	-48.0	-17.3	14.2	79.7	56.6	17.0	-60.8	27.3
		C	-39.0	-54.4	-55.3	-57.6	-34.0	-70.0	3.8	26.0	-17.8	-32.3	33.4	0.0	-30.7	10.2	52.3	139.6	108.9	56.0	-47.7	69.8
		K	-11.9	-34.2	-35.5	-38.8	-4.8	-56.7	49.8	81.8	18.6	-2.3	92.4	44.3	0.0	59.1	119.8	245.7	201.4	125.1	-24.5	145.0
		M	-44.6	-58.6	-59.4	-61.5	-40.1	-72.8	-5.8	14.3	-25.5	-38.6	21.0	-9.3	-37.1	0.0	38.2	117.4	89.5	41.5	-52.5	54.0
		V	-59.9	-70.1	-70.7	-72.1	-56.7	-80.3	-31.9	-17.3	-46.1	-55.6	-12.4	-34.4	-54.5	-27.6	0.0	57.3	37.1	2.4	-65.7	11.5
		I	-74.5	-81.0	-81.3	-82.3	-72.5	-87.5	-56.7	-47.4	-65.7	-71.7	-44.3	-58.3	-71.1	-54.0	-36.4	0.0	-12.8	-34.9	-78.2	-29.1

		L	-70.8	-78.2	-78.6	-79.7	-68.4	-85.6	-50.3	-39.7	-60.7	-67.6	-36.1	-52.1	-66.8	-47.2	-27.1	14.7	0.0	-25.3	-75.0	-18.7
		F	-60.9	-70.8	-71.3	-72.8	-57.7	-80.8	-33.5	-19.2	-47.3	-56.6	-14.5	-35.9	-55.6	-29.3	-2.4	53.6	33.9	0.0	-66.5	8.8
		Y	16.7	-12.8	-14.5	-18.9	26.2	-42.7	98.4	140.9	57.1	29.4	155.0	91.2	32.5	110.7	191.2	358.0	299.3	198.3	0.0	224.6
		W	-64.1	-73.1	-73.7	-75.0	-61.1	-82.3	-38.9	-25.8	-51.6	-60.1	-21.4	-41.1	-59.2	-35.1	-10.3	41.1	23.0	-8.1	-69.2	0.0
			H	N	S	Q	R	G	D	E	T	A	P	C	K	M	V	I	L	F	Y	W
			**B**																			
		H	0.0	-17.3	-21.1	-24.0	2.9	-42.6	29.5	51.7	22.5	-3.2	46.1	66.8	0.4	52.3	-3.2	132.0	110.9	78.8	1.4	116.1
		N	20.9	0.0	-4.6	-8.1	24.4	-30.6	56.6	83.4	48.1	17.1	76.6	101.6	21.3	84.0	16.9	180.5	154.9	116.1	22.6	161.2
		S	26.7	4.8	0.0	-3.7	30.4	-27.2	64.1	92.2	55.2	22.7	85.1	111.3	27.2	92.9	22.6	193.9	167.1	126.5	28.5	173.7
		Q	31.5	8.8	3.8	0.0	35.4	-24.5	70.4	99.5	61.2	27.4	92.2	119.4	32.0	100.3	27.3	205.2	177.4	135.2	33.4	184.2
		R	-2.8	-19.6	-23.3	-26.1	0.0	-44.2	25.9	47.4	19.1	-5.9	42.0	62.0	-2.5	47.9	-6.0	125.4	104.9	73.7	-1.4	109.9
		G	74.1	44.1	37.5	32.4	79.2	0.0	125.6	164.1	113.4	68.6	154.4	190.4	74.8	165.1	68.5	304.0	267.2	211.3	76.6	276.2
		D	-22.8	-36.1	-39.1	-41.3	-20.6	-55.7	0.0	17.1	-5.4	-25.2	12.8	28.7	-22.5	17.5	-25.3	79.1	62.8	38.0	-21.7	66.8
		E	-34.1	-45.5	-48.0	-49.9	-32.2	-62.1	-14.6	0.0	-19.2	-36.2	-3.7	9.9	-33.8	0.4	-36.2	53.0	39.0	17.9	-33.1	42.4
		T	-18.4	-32.5	-35.6	-38.0	-16.0	-53.1	5.7	23.8	0.0	-21.0	19.2	36.1	-18.1	24.3	-21.0	89.3	72.1	45.9	-17.2	76.3
	**A**	A	3.3	-14.6	-18.5	-21.5	6.3	-40.7	33.8	56.6	26.5	0.0	50.9	72.2	3.7	57.2	-0.1	139.6	117.8	84.6	4.8	123.1
3		P	-31.6	-43.4	-46.0	-48.0	-29.6	-60.7	-11.3	3.8	-16.1	-33.7	0.0	14.1	-31.3	4.2	-33.8	58.8	44.3	22.4	-30.6	47.9
		C	-40.0	-50.4	-52.7	-54.4	-38.3	-65.6	-22.3	-9.0	-26.5	-41.9	-12.4	0.0	-39.8	-8.7	-42.0	39.1	26.5	7.2	-39.2	29.6
		K	-0.4	-17.6	-21.4	-24.3	2.5	-42.8	29.0	51.1	22.1	-3.5	45.6	66.1	0.0	51.7	-3.6	131.1	110.1	78.1	1.0	115.2
		M	-34.3	-45.7	-48.2	-50.1	-32.4	-62.3	-14.9	-0.4	-19.5	-36.4	-4.0	9.5	-34.1	0.0	-36.5	52.4	38.5	17.4	-33.4	41.9
		V	3.4	-14.5	-18.4	-21.4	6.4	-40.6	33.9	56.8	26.7	0.1	51.0	72.4	3.8	57.4	0.0	139.8	118.0	84.8	4.9	123.3
		I	-56.9	-64.3	-66.0	-67.2	-55.6	-75.2	-44.2	-34.6	-47.2	-58.3	-37.0	-28.1	-56.7	-34.4	-58.3	0.0	-9.1	-22.9	-56.3	-6.9
		L	-52.6	-60.8	-62.6	-63.9	-51.2	-72.8	-38.6	-28.1	-41.9	-54.1	-30.7	-20.9	-52.4	-27.8	-54.1	10.0	0.0	-15.2	-51.9	2.5
		F	-44.1	-53.7	-55.8	-57.5	-42.4	-67.9	-27.5	-15.2	-31.5	-45.8	-18.3	-6.7	-43.9	-14.8	-45.9	29.8	17.9	0.0	-43.3	20.9
		Y	-1.4	-18.4	-22.2	-25.1	1.5	-43.4	27.7	49.5	20.8	-4.5	44.0	64.4	-1.0	50.1	-4.6	128.7	107.9	76.2	0.0	113.0
		W	-53.7	-61.7	-63.5	-64.8	-52.4	-73.4	-40.0	-29.8	-43.3	-55.2	-32.4	-22.8	-53.5	-29.5	-55.2	7.4	-2.4	-17.3	-53.1	0.0
			H	N	S	Q	R	G	D	E	T	A	P	C	K	M	V	I	L	F	Y	W
			**B**																			

***Supplementary Table 34**: Raw data Extended Data Fig. 3 – AA in water, 170 µm, 18 h, pH 9.9 – enrichments in bottom fraction*

repeat		species	Ratio species against species (shown in heat maps), Eq. 2 $[A]_{j,k}/[B]_{j,k} - 1$ (%)																			
1	A	H	0.0	5.6	9.2	12.0	2.4	25.2	-8.9	-13.6	-9.2	-1.2	-16.0	-19.7	3.8	-13.9	-15.9	-26.4	-24.4	-20.2	2.1	-18.1
		N	-5.3	0.0	3.5	6.0	-3.1	18.5	-13.7	-18.1	-14.0	-6.4	-20.5	-24.0	-1.7	-18.4	-20.3	-30.3	-28.4	-24.4	-3.3	-22.5
		S	-8.5	-3.3	0.0	2.5	-6.3	14.6	-16.6	-20.9	-16.9	-9.6	-23.1	-26.5	-5.0	-21.2	-23.0	-32.6	-30.8	-26.9	-6.6	-25.1
		Q	-10.7	-5.7	-2.4	0.0	-8.6	11.8	-18.6	-22.8	-18.9	-11.8	-25.0	-28.3	-7.3	-23.1	-24.8	-34.2	-32.5	-28.7	-8.8	-26.9
		R	-2.3	3.1	6.7	9.4	0.0	22.3	-11.0	-15.6	-11.3	-3.5	-17.9	-21.6	1.4	-15.9	-17.8	-28.1	-26.1	-22.0	-0.3	-20.0
		G	-20.1	-15.6	-12.7	-10.6	-18.2	0.0	-27.2	-30.9	-27.4	-21.1	-32.9	-35.9	-17.1	-31.2	-32.8	-41.2	-39.6	-36.2	-18.5	-34.6
		D	9.7	15.9	19.9	22.9	12.3	37.4	0.0	-5.1	-0.3	8.4	-7.8	-11.9	13.9	-5.5	-7.7	-19.2	-17.0	-12.4	12.0	-10.2
		E	15.7	22.2	26.4	29.5	18.4	44.8	5.4	0.0	5.1	14.3	-2.8	-7.1	20.0	-0.4	-2.7	-14.8	-12.5	-7.6	18.1	-5.3
		T	10.1	16.3	20.3	23.3	12.7	37.8	0.3	-4.8	0.0	8.8	-7.5	-11.6	14.3	-5.2	-7.4	-18.9	-16.7	-12.1	12.4	-9.9
		A	1.2	6.9	10.6	13.3	3.6	26.7	-7.8	-12.5	-8.1	0.0	-15.0	-18.7	5.0	-12.8	-14.8	-25.5	-23.5	-19.2	3.3	-17.1
		P	19.0	25.7	30.1	33.3	21.9	49.0	8.5	2.9	8.1	17.6	0.0	-4.4	23.5	2.5	0.2	-12.3	-10.0	-5.0	21.5	-2.5
		C	24.5	31.5	36.1	39.4	27.5	55.9	13.5	7.7	13.1	23.0	4.6	0.0	29.2	7.3	4.8	-8.3	-5.8	-0.6	27.1	2.0
		K	-3.6	1.8	5.3	7.9	-1.3	20.6	-12.2	-16.7	-12.5	-4.8	-19.1	-22.6	0.0	-17.0	-18.9	-29.0	-27.1	-23.1	-1.6	-21.1
		M	16.1	22.6	26.8	30.0	18.9	45.3	5.8	0.4	5.4	14.7	-2.5	-6.8	20.5	0.0	-2.3	-14.5	-12.2	-7.3	18.5	-5.0
		V	18.8	25.5	29.8	33.1	21.7	48.8	8.3	2.7	7.9	17.4	-0.2	-4.6	23.3	2.4	0.0	-12.5	-10.1	-5.1	21.3	-2.7
		I	35.8	43.4	48.4	52.0	39.0	70.0	23.8	17.4	23.3	34.2	14.1	9.0	40.9	17.0	14.3	0.0	2.7	8.4	38.6	11.2
		L	32.3	39.7	44.5	48.1	35.4	65.6	20.5	14.3	20.1	30.7	11.1	6.2	37.2	13.9	11.3	-2.6	0.0	5.6	35.0	8.3
		F	25.3	32.3	36.8	40.2	28.2	56.8	14.1	8.3	13.8	23.8	5.2	0.6	30.0	7.9	5.4	-7.8	-5.3	0.0	27.9	2.5
		Y	-2.0	3.4	7.0	9.7	0.3	22.6	-10.7	-15.3	-11.0	-3.2	-17.7	-21.3	1.7	-15.6	-17.6	-27.9	-25.9	-21.8	0.0	-19.8
		W	22.2	29.0	33.5	36.8	25.1	52.9	11.3	5.6	10.9	20.7	2.6	-1.9	26.8	5.2	2.8	-10.1	-7.6	-2.5	24.7	0.0
			H	N	S	Q	R	G	D	E	T	A	P	C	K	M	V	I	L	F	Y	W
			B																			
2	A	H	0.0	8.3	11.3	15.0	1.0	34.3	-11.6	-15.5	-9.5	-2.1	-17.0	-17.1	1.2	-14.1	-21.2	-26.3	-24.5	-20.7	1.1	-18.7
		N	-7.7	0.0	2.8	6.2	-6.7	24.0	-18.4	-22.0	-16.5	-9.6	-23.4	-23.4	-6.5	-20.7	-27.2	-31.9	-30.3	-26.8	-6.6	-24.9
		S	-10.1	-2.7	0.0	3.4	-9.2	20.6	-20.6	-24.1	-18.7	-12.0	-25.4	-25.5	-9.0	-22.8	-29.2	-33.8	-32.2	-28.8	-9.1	-27.0
		Q	-13.1	-5.8	-3.2	0.0	-12.2	16.7	-23.1	-26.5	-21.3	-14.8	-27.9	-27.9	-12.0	-25.3	-31.5	-35.9	-34.4	-31.1	-12.1	-29.3
		R	-1.0	7.2	10.2	13.9	0.0	32.9	-12.5	-16.3	-10.4	-3.0	-17.9	-17.9	0.2	-14.9	-22.0	-27.0	-25.3	-21.5	0.1	-19.5
		G	-25.5	-19.3	-17.1	-14.3	-24.8	0.0	-34.2	-37.1	-32.6	-27.0	-38.2	-38.2	-24.6	-36.0	-41.3	-45.1	-43.8	-40.9	-24.7	-39.5
		D	13.1	22.5	25.9	30.1	14.3	51.9	0.0	-4.4	2.3	10.8	-6.1	-6.2	14.5	-2.8	-10.8	-16.6	-14.6	-10.3	14.4	-8.0
		E	18.3	28.1	31.7	36.1	19.5	58.9	4.6	0.0	7.0	15.9	-1.8	-1.9	19.8	1.7	-6.7	-12.8	-10.7	-6.2	19.7	-3.8
		T	10.5	19.7	23.0	27.1	11.6	48.4	-2.3	-6.6	0.0	8.3	-8.3	-8.4	11.9	-5.0	-12.9	-18.5	-16.6	-12.4	11.8	-10.1
		A	2.1	10.6	13.6	17.4	3.1	37.1	-9.7	-13.7	-7.6	0.0	-15.3	-15.3	3.4	-12.3	-19.5	-24.7	-22.9	-19.1	3.3	-17.0
		P	20.5	30.5	34.1	38.6	21.7	61.8	6.5	1.9	9.0	18.0	0.0	-0.1	22.0	3.6	-5.0	-11.1	-9.0	-4.4	21.9	-2.0
		C	20.6	30.6	34.2	38.7	21.8	61.9	6.6	1.9	9.1	18.1	0.1	0.0	22.1	3.6	-4.9	-11.1	-9.0	-4.4	22.0	-2.0
		K	-1.2	7.0	9.9	13.6	-0.2	32.6	-12.7	-16.5	-10.6	-3.3	-18.1	-18.1	0.0	-15.1	-22.1	-27.2	-25.5	-21.7	-0.1	-19.7
		M	16.4	26.1	29.5	33.9	17.6	56.3	2.9	-1.6	5.3	14.0	-3.4	-3.5	17.8	0.0	-8.3	-14.2	-12.2	-7.7	17.7	-5.4
		V	26.9	37.4	41.2	45.9	28.1	70.3	12.1	7.2	14.8	24.2	5.3	5.2	28.4	9.0	0.0	-6.5	-4.3	0.6	28.3	3.1
		I	35.7	46.9	51.0	56.0	37.0	82.1	19.9	14.6	22.7	32.9	12.5	12.5	37.3	16.5	6.9	0.0	2.4	7.5	37.2	10.3

		L	32.5	43.5	47.4	52.4	33.8	77.9	17.1	12.0	19.9	29.8	9.9	9.9	34.1	13.8	4.4	-2.3	0.0	5.0	34.0	7.7
		F	26.1	36.6	40.4	45.1	27.4	69.3	11.5	6.6	14.1	23.5	4.7	4.6	27.7	8.4	-0.6	-7.0	-4.8	0.0	27.6	2.5
		Y	-1.1	7.1	10.0	13.7	-0.1	32.8	-12.6	-16.4	-10.5	-3.2	-18.0	-18.0	0.1	-15.0	-22.1	-27.1	-25.4	-21.6	0.0	-19.6
		W	23.0	33.2	36.9	41.5	24.2	65.2	8.7	4.0	11.3	20.5	2.1	2.0	24.5	5.7	-3.0	-9.3	-7.2	-2.5	24.4	0.0
			H	N	S	Q	R	G	D	E	T	A	P	C	K	M	V	I	L	F	Y	W
			**B**																			
3	**A**	H	0.0	9.1	16.5	16.1	0.5	43.5	-10.9	-15.6	-8.6	1.5	-14.0	-18.3	0.9	-14.4	-15.8	-24.6	-23.1	-20.3	-4.1	-21.7
		N	-8.3	0.0	6.8	6.4	-7.8	31.6	-18.4	-22.6	-16.2	-7.0	-21.2	-25.1	-7.5	-21.5	-22.8	-30.8	-29.5	-26.9	-12.1	-28.2
		S	-14.1	-6.3	0.0	-0.3	-13.7	23.3	-23.5	-27.5	-21.5	-12.9	-26.2	-29.8	-13.3	-26.5	-27.7	-35.2	-33.9	-31.5	-17.7	-32.7
		Q	-13.8	-6.0	0.3	0.0	-13.4	23.7	-23.3	-27.3	-21.2	-12.6	-25.9	-29.6	-13.0	-26.2	-27.5	-35.0	-33.7	-31.3	-17.4	-32.5
		R	-0.5	8.5	15.8	15.5	0.0	42.8	-11.4	-16.0	-9.1	0.9	-14.5	-18.7	0.4	-14.8	-16.3	-25.0	-23.5	-20.7	-4.6	-22.1
		G	-30.3	-24.0	-18.9	-19.1	-30.0	0.0	-38.0	-41.2	-36.3	-29.3	-40.1	-43.1	-29.7	-40.3	-41.4	-47.4	-46.4	-44.4	-33.2	-45.4
		D	12.3	22.5	30.8	30.3	12.9	61.2	0.0	-5.2	2.6	13.9	-3.5	-8.2	13.4	-3.8	-5.5	-15.3	-13.6	-10.5	7.6	-12.0
		E	18.4	29.2	37.9	37.5	19.1	70.0	5.5	0.0	8.3	20.2	1.8	-3.2	19.5	1.4	-0.3	-10.7	-8.9	-5.6	13.5	-7.2
		T	9.4	19.3	27.4	27.0	10.0	57.0	-2.6	-7.6	0.0	11.0	-6.0	-10.6	10.4	-6.3	-7.9	-17.5	-15.9	-12.8	4.9	-14.3
		A	-1.4	7.5	14.8	14.4	-0.9	41.5	-12.2	-16.8	-9.9	0.0	-15.3	-19.4	-0.5	-15.6	-17.1	-25.7	-24.2	-21.4	-5.5	-22.8
		P	16.3	26.9	35.5	35.0	17.0	67.0	3.6	-1.8	6.3	18.0	0.0	-4.9	17.4	-0.4	-2.1	-12.3	-10.5	-7.2	11.5	-8.9
		C	22.3	33.4	42.5	42.0	23.0	75.6	9.0	3.3	11.8	24.1	5.2	0.0	23.5	4.8	3.0	-7.7	-5.9	-2.5	17.3	-4.1
		K	-0.9	8.1	15.4	15.0	-0.4	42.2	-11.8	-16.4	-9.4	0.5	-14.8	-19.0	0.0	-15.2	-16.6	-25.3	-23.8	-21.0	-5.0	-22.4
		M	16.8	27.4	36.0	35.5	17.4	67.6	4.0	-1.4	6.7	18.5	0.4	-4.6	17.9	0.0	-1.7	-11.9	-10.2	-6.9	11.9	-8.5
		V	18.8	29.6	38.4	37.9	19.4	70.5	5.8	0.3	8.6	20.6	2.1	-2.9	19.9	1.7	0.0	-10.4	-8.6	-5.3	13.9	-6.9
		I	32.6	44.6	54.4	53.9	33.3	90.3	18.1	11.9	21.2	34.5	14.0	8.4	33.8	13.5	11.6	0.0	2.0	5.7	27.1	3.9
		L	30.0	41.8	51.4	50.9	30.7	86.6	15.8	9.8	18.8	31.9	11.8	6.3	31.2	11.3	9.4	-1.9	0.0	3.7	24.6	1.9
		F	25.4	36.8	46.1	45.6	26.1	80.0	11.7	5.9	14.6	27.3	7.8	2.5	26.6	7.4	5.6	-5.4	-3.5	0.0	20.2	-1.7
		Y	4.3	13.8	21.5	21.1	4.9	49.7	-7.1	-11.9	-4.6	5.8	-10.3	-14.7	5.3	-10.7	-12.2	-21.3	-19.8	-16.8	0.0	-18.3
		W	27.6	39.2	48.6	48.2	28.3	83.2	13.7	7.8	16.7	29.5	9.7	4.3	28.8	9.3	7.4	-3.7	-1.8	1.8	22.4	0.0
			H	N	S	Q	R	G	D	E	T	A	P	C	K	M	V	I	L	F	Y	W
			**B**																			

***Supplementary Table 35**: Raw data Extended Data Fig. 4 – AA in water, 170 µm, 18 h, 1 mM NaCl, pH 7.4*

repeat k	species	measured values (counts*min)				concentration (µM) $[A]_{j,k,HPLC}$ calibration acc. to Supplementary Table 4				c_0 (µM)	Normalization (Eq. 1) concentration (x c_0)				Ratio species vs mean (Eq. 4) $[A]_{j,k}/\bar{c}_{j,k}-1$ (%)	
		top	II	III	bot	top	II	III	bot		top	II	III	bot	top	bot
1	H	240129	228509	248856	270086	26.75	25.46	27.72	30.09	27.50	0.97	0.93	1.01	1.09	2.8	-2.0
	N	445395	398460	431669	460378	40.30	36.06	39.06	41.66	39.27	1.03	0.92	0.99	1.06	8.5	-5.0
	S	396440	365236	391027	432022	28.25	26.03	27.87	30.79	28.23	1.00	0.92	0.99	1.09	5.8	-2.4
	Q	372695	345029	368662	399270	32.37	29.97	32.02	34.68	32.26	1.00	0.93	0.99	1.08	6.1	-3.7
	D	368979	362075	398502	438519	33.24	32.62	35.90	39.51	35.32	0.94	0.92	1.02	1.12	-0.5	0.2
	E	345228	345953	383664	421874	31.94	32.01	35.50	39.04	34.62	0.92	0.92	1.03	1.13	-2.5	1.0
	T	410640	398772	437069	479041	26.49	25.72	28.19	30.90	27.83	0.95	0.92	1.01	1.11	0.6	-0.6
	A	383676	368233	408779	446861	26.67	25.60	28.41	31.06	27.94	0.95	0.92	1.02	1.11	0.9	-0.4
	P	102069	103787	117429	129617	24.36	24.77	28.03	30.94	27.02	0.90	0.92	1.04	1.14	-4.7	2.5
	C	27079	27023	29840	34156	19.55	19.51	21.54	24.66	21.32	0.92	0.92	1.01	1.16	-3.1	3.6
	K	330308	317788	345357	375746	31.25	30.07	32.68	35.55	32.39	0.96	0.93	1.01	1.10	2.0	-1.7
	V	533798	505323	581994	630861	27.78	26.29	30.28	32.83	29.29	0.95	0.90	1.03	1.12	0.2	0.3
	I	470144	492384	558804	622989	22.51	23.57	26.75	29.83	25.66	0.88	0.92	1.04	1.16	-7.3	4.1
	L	438339	445203	507954	558879	23.99	24.37	27.80	30.59	26.69	0.90	0.91	1.04	1.15	-5.0	2.6
	F	375372	380924	425519	467691	24.67	25.03	27.96	30.73	27.10	0.91	0.92	1.03	1.13	-3.8	1.6
	mean concentration per fraction $\bar{c}_{j,k}$										0.95	0.92	1.02	1.12		
2	H	189131	263141	216165	309541	21.07	29.31	24.08	34.48	27.24	0.77	1.08	0.88	1.27	1.4	-2.4
	N	351665	460045	377286	515134	31.82	41.63	34.14	46.62	38.55	0.83	1.08	0.89	1.21	8.2	-6.8
	S	314792	419321	337242	468966	22.43	29.88	24.03	33.42	27.44	0.82	1.09	0.88	1.22	7.2	-6.1
	Q	291966	392190	317134	435468	25.36	34.07	27.55	37.83	31.20	0.81	1.09	0.88	1.21	6.6	-6.6
	D	297663	410603	346339	501995	26.82	36.99	31.20	45.23	35.06	0.76	1.06	0.89	1.29	0.3	-0.6
	E	279535	393242	332058	492054	25.86	36.39	30.72	45.53	34.63	0.75	1.05	0.89	1.31	-2.1	1.3

	T	328840	456837	378122	545952	21.21	29.47	24.39	35.22	27.57	0.77	1.07	0.88	1.28	0.8	-1.6
	A	311232	426409	349443	505379	21.63	29.64	24.29	35.13	27.67	0.78	1.07	0.88	1.27	2.5	-2.2
	P	83665	121410	100875	155100	19.97	28.98	24.08	37.02	27.51	0.73	1.05	0.88	1.35	-4.9	3.7
	C	22142	31860	25885	39520	15.99	23.00	18.69	28.53	21.55	0.74	1.07	0.87	1.32	-2.8	2.0
	K	262383	360143	300681	428955	24.83	34.08	28.45	40.59	31.98	0.78	1.07	0.89	1.27	1.7	-2.2
	V	428096	567661	517430	773232	22.28	29.54	26.92	40.23	29.74	0.75	0.99	0.91	1.35	-1.8	4.3
	I	383630	563370	486184	767202	18.37	26.97	23.28	36.73	26.34	0.70	1.02	0.88	1.39	-8.6	7.5
	L	363428	493363	446472	677633	19.89	27.00	24.44	37.09	27.10	0.73	1.00	0.90	1.37	-3.8	5.5
	F	303814	434544	369083	564551	19.96	28.55	24.25	37.10	27.47	0.73	1.04	0.88	1.35	-4.7	4.1
	mean concentration per fraction $\bar{c}_{j,k}$										0.76	1.05	0.88	1.30		
3	H	196502	243344	280460	326644	21.89	27.11	31.24	36.39	29.16	0.75	0.93	1.07	1.25	10.8	-5.6
	N	366517	429117	486459	521288	33.17	38.83	44.02	47.17	40.80	0.81	0.95	1.08	1.16	20.0	-12.5
	S	333932	392052	441604	476646	23.80	27.94	31.47	33.97	29.29	0.81	0.95	1.07	1.16	19.9	-12.3
	Q	300096	364856	409761	440921	26.07	31.69	35.59	38.30	32.91	0.79	0.96	1.08	1.16	16.9	-11.9
	D	275173	387515	448942	547803	24.79	34.91	40.45	49.36	37.38	0.66	0.93	1.08	1.32	-2.1	-0.1
	E	254073	371302	428383	541354	23.51	34.36	39.64	50.09	36.90	0.64	0.93	1.07	1.36	-5.9	2.7
	T	317414	427170	493242	586441	20.48	27.56	31.82	37.83	29.42	0.70	0.94	1.08	1.29	2.8	-2.7
	A	303451	399516	458088	539780	21.09	27.77	31.84	37.52	29.56	0.71	0.94	1.08	1.27	5.4	-3.9
	P	74260	108808	133685	174434	17.72	25.97	31.91	41.63	29.31	0.60	0.89	1.09	1.42	-10.7	7.5
	C	20475	28197	33598	43901	14.78	20.36	24.26	31.70	22.77	0.65	0.89	1.07	1.39	-4.2	5.3
	K	256916	344999	391574	453888	24.31	32.64	37.05	42.95	34.24	0.71	0.95	1.08	1.25	4.8	-5.1
	V	370458	577338	648297	857633	19.28	30.04	33.73	44.63	31.92	0.60	0.94	1.06	1.40	-10.8	5.8
	I	316378	524358	625080	898488	15.15	25.10	29.93	43.01	28.30	0.54	0.89	1.06	1.52	-21.0	15.0
	L	307635	480636	564607	779463	16.84	26.31	30.90	42.66	29.18	0.58	0.90	1.06	1.46	-14.8	10.6
	F	268158	407992	475933	630506	17.62	26.81	31.27	41.43	29.28	0.60	0.92	1.07	1.41	-11.2	7.1
	mean concentration per fraction $\bar{c}_{j,k}$										0.68	0.93	1.07	1.32		

Supplementary Table 36*: Raw data Extended Data Fig. 4 – AA in water, 170 µm, 18 h, 1 mM NaCl, pH 7.4 – enrichments in top fraction*

**repeat**		**species**	Ratio species against species (shown in heat maps), Eq. 2 $[A]_{j,k}/[B]_{j,k} - 1$ (%)														
		H	0.0	-5.2	-2.8	-3.1	3.3	5.4	2.2	1.9	7.9	6.0	0.8	2.6	10.9	8.2	6.8
		N	5.5	0.0	2.6	2.3	9.0	11.2	7.8	7.5	13.8	11.9	6.4	8.2	17.0	14.2	12.7
		S	2.9	-2.5	0.0	-0.3	6.3	8.5	5.1	4.8	11.0	9.1	3.7	5.5	14.1	11.3	9.9
		Q	3.2	-2.2	0.3	0.0	6.6	8.8	5.4	5.1	11.3	9.4	4.0	5.8	14.4	11.6	10.2
		D	-3.2	-8.3	-5.9	-6.2	0.0	2.0	-1.1	-1.4	4.4	2.6	-2.5	-0.7	7.3	4.7	3.4
		E	-5.1	-10.1	-7.8	-8.1	-2.0	0.0	-3.1	-3.4	2.3	0.6	-4.4	-2.7	5.2	2.6	1.4
		T	-2.1	-7.2	-4.9	-5.1	1.1	3.2	0.0	-0.3	5.6	3.8	-1.4	0.4	8.5	5.9	4.6
	**A**	A	-1.8	-7.0	-4.6	-4.9	1.4	3.5	0.3	0.0	5.9	4.1	-1.1	0.7	8.9	6.2	4.9
1		P	-7.3	-12.2	-9.9	-10.2	-4.2	-2.3	-5.3	-5.6	0.0	-1.7	-6.6	-4.9	2.8	0.3	-1.0
		C	-5.7	-10.6	-8.3	-8.6	-2.6	-0.6	-3.7	-3.9	1.7	0.0	-5.0	-3.3	4.6	2.0	0.8
		K	-0.8	-6.0	-3.6	-3.8	2.5	4.6	1.4	1.1	7.0	5.2	0.0	1.8	10.0	7.3	6.0
		V	-2.5	-7.6	-5.2	-5.5	0.7	2.8	-0.4	-0.7	5.2	3.4	-1.7	0.0	8.1	5.5	4.2
		I	-9.8	-14.5	-12.4	-12.6	-6.8	-4.9	-7.9	-8.1	-2.7	-4.4	-9.1	-7.5	0.0	-2.4	-3.7
		L	-7.6	-12.4	-10.2	-10.4	-4.5	-2.6	-5.6	-5.8	-0.3	-2.0	-6.8	-5.2	2.5	0.0	-1.2
		F	-6.4	-11.3	-9.0	-9.3	-3.3	-1.3	-4.4	-4.7	1.0	-0.8	-5.7	-4.0	3.8	1.3	0.0
			H	N	S	Q	D	E	T	A	P	C	K	V	I	L	F
			**B**														
		H	0.0	-6.3	-5.4	-4.8	1.1	3.6	0.5	-1.0	6.6	4.3	-0.3	3.3	10.9	5.4	6.4
		N	6.7	0.0	1.0	1.5	7.9	10.5	7.3	5.6	13.7	11.3	6.3	10.2	18.4	12.5	13.6
		S	5.7	-1.0	0.0	0.6	6.9	9.4	6.3	4.6	12.6	10.2	5.3	9.2	17.2	11.4	12.5
		Q	5.1	-1.5	-0.6	0.0	6.3	8.8	5.7	4.0	12.0	9.6	4.7	8.5	16.6	10.8	11.8
		D	-1.1	-7.3	-6.4	-5.9	0.0	2.4	-0.6	-2.2	5.4	3.1	-1.5	2.1	9.7	4.2	5.2
		E	-3.4	-9.5	-8.6	-8.1	-2.3	0.0	-2.9	-4.4	2.9	0.7	-3.8	-0.3	7.1	1.8	2.8
		T	-0.5	-6.8	-5.9	-5.4	0.6	3.0	0.0	-1.6	6.0	3.7	-0.9	2.7	10.3	4.8	5.8
	**A**	A	1.1	-5.3	-4.4	-3.8	2.2	4.7	1.6	0.0	7.7	5.4	0.7	4.4	12.1	6.5	7.6
2		P	-6.2	-12.1	-11.2	-10.7	-5.1	-2.8	-5.6	-7.2	0.0	-2.1	-6.5	-3.1	4.1	-1.1	-0.1
		C	-4.1	-10.1	-9.3	-8.7	-3.0	-0.7	-3.6	-5.1	2.2	0.0	-4.4	-1.0	6.4	1.1	2.1
		K	0.3	-6.0	-5.0	-4.5	1.5	3.9	0.9	-0.7	6.9	4.6	0.0	3.6	11.3	5.8	6.8
		V	-3.2	-9.3	-8.4	-7.9	-2.1	0.3	-2.7	-4.2	3.2	1.0	-3.5	0.0	7.4	2.1	3.0
		I	-9.8	-15.5	-14.7	-14.2	-8.8	-6.6	-9.4	-10.8	-3.9	-6.0	-10.2	-6.9	0.0	-5.0	-4.1
		L	-5.1	-11.1	-10.2	-9.7	-4.1	-1.8	-4.6	-6.1	1.1	-1.1	-5.5	-2.0	5.2	0.0	1.0
		F	-6.0	-11.9	-11.1	-10.6	-5.0	-2.7	-5.5	-7.0	0.1	-2.0	-6.4	-3.0	4.2	-1.0	0.0
			H	N	S	Q	D	E	T	A	P	C	K	V	I	L	F
			**B**														
		H	0.0	-7.7	-7.6	-5.2	13.2	17.8	7.9	5.2	24.1	15.7	5.7	24.3	40.3	30.1	24.8
3	**A**	N	8.3	0.0	0.1	2.6	22.6	27.6	16.8	13.9	34.4	25.2	14.5	34.6	51.9	40.9	35.1
		S	8.2	-0.1	0.0	2.6	22.5	27.5	16.7	13.8	34.3	25.2	14.4	34.5	51.8	40.8	35.0
		Q	5.5	-2.6	-2.5	0.0	19.4	24.3	13.8	11.0	31.0	22.0	11.5	31.1	48.0	37.2	31.6

		D	-11.7	-18.4	-18.4	-16.3	0.0	4.1	-4.7	-7.1	9.7	2.2	-6.6	9.8	23.9	14.9	10.2
		E	-15.1	-21.6	-21.6	-19.6	-3.9	0.0	-8.5	-10.7	5.4	-1.8	-10.3	5.5	19.0	10.4	5.9
		T	-7.3	-14.4	-14.3	-12.1	4.9	9.2	0.0	-2.5	15.1	7.2	-2.0	15.2	30.0	20.6	15.7
		A	-4.9	-12.2	-12.2	-9.9	7.6	12.0	2.5	0.0	18.0	9.9	0.5	18.2	33.3	23.7	18.6
		P	-19.4	-25.6	-25.6	-23.6	-8.8	-5.1	-13.1	-15.3	0.0	-6.8	-14.8	0.1	13.0	4.8	0.5
		C	-13.5	-20.2	-20.1	-18.0	-2.1	1.9	-6.7	-9.0	7.3	0.0	-8.6	7.5	21.3	12.5	7.9
		K	-5.4	-12.7	-12.6	-10.4	7.0	11.4	2.0	-0.5	17.4	9.4	0.0	17.6	32.7	23.0	18.0
		V	-19.6	-25.7	-25.7	-23.7	-9.0	-5.2	-13.2	-15.4	-0.1	-7.0	-14.9	0.0	12.8	4.6	0.4
		I	-28.7	-34.2	-34.1	-32.4	-19.3	-16.0	-23.1	-25.0	-11.5	-17.5	-24.6	-11.4	0.0	-7.2	-11.0
		L	-23.1	-29.0	-29.0	-27.1	-13.0	-9.4	-17.1	-19.1	-4.6	-11.1	-18.7	-4.4	7.8	0.0	-4.1
		F	-19.9	-26.0	-25.9	-24.0	-9.3	-5.6	-13.5	-15.7	-0.5	-7.3	-15.3	-0.4	12.4	4.3	0.0
			H	N	S	Q	D	E	T	A	P	C	K	V	I	L	F
			**B**														

***Supplementary Table 37**: Raw data Extended Data Fig. 4 – AA in water, 170 µm, 18 h, 1 mM NaCl, pH 7.4 – enrichments in bottom fraction*

repeat		species	Ratio species against species (shown in heat maps), Eq. 2 $[A]_{j,k}/[B]_{j,k} - 1$ (%)														
1	**A**	H	0.0	3.1	0.3	1.8	-2.2	-3.0	-1.5	-1.6	-4.4	-5.4	-0.3	-2.4	-5.9	-4.6	-3.5
		N	-3.0	0.0	-2.7	-1.3	-5.2	-5.9	-4.5	-4.6	-7.3	-8.3	-3.4	-5.3	-8.7	-7.4	-6.5
		S	-0.3	2.8	0.0	1.4	-2.5	-3.3	-1.8	-1.9	-4.7	-5.7	-0.7	-2.7	-6.2	-4.9	-3.9
		Q	-1.7	1.3	-1.4	0.0	-3.9	-4.7	-3.2	-3.3	-6.1	-7.1	-2.1	-4.1	-7.5	-6.2	-5.2
		D	2.3	5.4	2.6	4.1	0.0	-0.8	0.7	0.6	-2.3	-3.3	1.9	-0.2	-3.7	-2.4	-1.4
		E	3.1	6.3	3.4	4.9	0.8	0.0	1.5	1.4	-1.5	-2.5	2.7	0.6	-3.0	-1.6	-0.6
		T	1.5	4.7	1.8	3.3	-0.7	-1.5	0.0	-0.1	-3.0	-4.0	1.2	-0.9	-4.4	-3.1	-2.1
		A	1.6	4.8	2.0	3.4	-0.6	-1.4	0.1	0.0	-2.9	-3.9	1.3	-0.8	-4.3	-3.0	-2.0
		P	4.6	7.9	5.0	6.5	2.3	1.5	3.1	3.0	0.0	-1.0	4.3	2.2	-1.5	-0.1	0.9
		C	5.8	9.1	6.1	7.6	3.4	2.6	4.2	4.0	1.1	0.0	5.4	3.2	-0.5	0.9	2.0
		K	0.3	3.5	0.7	2.1	-1.9	-2.6	-1.2	-1.3	-4.1	-5.1	0.0	-2.0	-5.5	-4.2	-3.2
		V	2.4	5.6	2.8	4.2	0.2	-0.6	0.9	0.8	-2.1	-3.1	2.1	0.0	-3.6	-2.2	-1.2
		I	6.2	9.5	6.6	8.1	3.9	3.1	4.6	4.5	1.5	0.5	5.9	3.7	0.0	1.4	2.5
		L	4.8	8.0	5.1	6.6	2.5	1.7	3.2	3.1	0.1	-0.9	4.4	2.3	-1.4	0.0	1.1
		F	3.7	6.9	4.0	5.5	1.4	0.6	2.1	2.0	-0.9	-2.0	3.3	1.2	-2.4	-1.1	0.0
			H	N	S	Q	D	E	T	A	P	C	K	V	I	L	F
			**B**														
2	**A**	H	0.0	4.7	4.0	4.4	-1.9	-3.7	-0.9	-0.3	-5.9	-4.4	-0.2	-6.4	-9.2	-7.5	-6.3
		N	-4.5	0.0	-0.7	-0.3	-6.3	-8.0	-5.3	-4.7	-10.1	-8.7	-4.7	-10.6	-13.3	-11.6	-10.5
		S	-3.8	0.7	0.0	0.5	-5.6	-7.4	-4.7	-4.1	-9.5	-8.0	-4.0	-10.0	-12.7	-11.0	-9.8
		Q	-4.2	0.3	-0.4	0.0	-6.0	-7.8	-5.1	-4.5	-9.9	-8.4	-4.5	-10.4	-13.1	-11.4	-10.2
		D	1.9	6.7	5.9	6.4	0.0	-1.9	1.0	1.6	-4.1	-2.6	1.7	-4.6	-7.5	-5.7	-4.5
		E	3.9	8.7	8.0	8.5	1.9	0.0	2.9	3.6	-2.3	-0.7	3.6	-2.8	-5.7	-3.9	-2.6
		T	0.9	5.6	4.9	5.4	-1.0	-2.9	0.0	0.6	-5.1	-3.5	0.7	-5.6	-8.4	-6.7	-5.4
		A	0.3	5.0	4.2	4.7	-1.6	-3.5	-0.6	0.0	-5.7	-4.1	0.0	-6.2	-9.0	-7.2	-6.0
		P	6.3	11.3	10.5	11.0	4.3	2.3	5.4	6.0	0.0	1.6	6.0	-0.5	-3.5	-1.7	-0.4
		C	4.6	9.5	8.7	9.2	2.6	0.7	3.6	4.3	-1.6	0.0	4.3	-2.1	-5.1	-3.2	-2.0
		K	0.2	4.9	4.2	4.7	-1.6	-3.5	-0.7	0.0	-5.7	-4.1	0.0	-6.2	-9.0	-7.3	-6.0
		V	6.8	11.9	11.1	11.6	4.9	2.9	5.9	6.6	0.5	2.2	6.6	0.0	-3.0	-1.1	0.2
		I	10.2	15.3	14.5	15.0	8.1	6.1	9.2	9.9	3.6	5.3	9.9	3.1	0.0	1.9	3.3
		L	8.1	13.2	12.4	12.9	6.1	4.1	7.1	7.8	1.7	3.4	7.8	1.2	-1.9	0.0	1.3
		F	6.7	11.7	10.9	11.4	4.7	2.7	5.7	6.4	0.4	2.0	6.4	-0.2	-3.2	-1.3	0.0
			H	N	S	Q	D	E	T	A	P	C	K	V	I	L	F
			**B**														
3	**A**	H	0.0	7.9	7.6	7.2	-5.5	-8.1	-2.9	-1.7	-12.1	-10.3	-0.5	-10.7	-17.9	-14.6	-11.8
		N	-7.4	0.0	-0.3	-0.6	-12.4	-14.8	-10.1	-8.9	-18.6	-16.9	-7.8	-17.3	-23.9	-20.9	-18.3
		S	-7.1	0.3	0.0	-0.4	-12.2	-14.6	-9.8	-8.7	-18.4	-16.7	-7.6	-17.1	-23.7	-20.7	-18.0
		Q	-6.8	0.6	0.4	0.0	-11.9	-14.3	-9.5	-8.3	-18.1	-16.4	-7.2	-16.8	-23.4	-20.4	-17.8

D	5.8	14.2	13.9	13.5	0.0	-2.7	2.7	4.0	-7.0	-5.1	5.3	-5.6	-13.1	-9.7	-6.7
E	8.8	17.4	17.1	16.7	2.8	0.0	5.6	6.9	-4.4	-2.5	8.2	-2.9	-10.7	-7.2	-4.0
T	3.0	11.2	10.9	10.5	-2.6	-5.3	0.0	1.3	-9.5	-7.6	2.5	-8.0	-15.4	-12.1	-9.1
A	1.7	9.8	9.5	9.1	-3.9	-6.5	-1.3	0.0	-10.6	-8.8	1.2	-9.2	-16.5	-13.2	-10.3
P	13.8	22.9	22.5	22.1	7.6	4.6	10.5	11.9	0.0	2.1	13.2	1.6	-6.6	-2.8	0.4
C	11.5	20.4	20.0	19.6	5.4	2.5	8.2	9.6	-2.0	0.0	11.0	-0.5	-8.4	-4.8	-1.6
K	0.5	8.5	8.2	7.8	-5.0	-7.6	-2.4	-1.2	-11.7	-9.9	0.0	-10.3	-17.5	-14.2	-11.3
V	12.0	20.9	20.6	20.1	5.9	3.0	8.7	10.1	-1.6	0.5	11.5	0.0	-8.0	-4.4	-1.2
I	21.8	31.5	31.1	30.6	15.1	12.0	18.2	19.7	7.0	9.2	21.2	8.7	0.0	4.0	7.4
L	17.2	26.5	26.1	25.7	10.7	7.7	13.7	15.2	2.9	5.1	16.6	4.6	-3.8	0.0	3.3
F	13.4	22.4	22.0	21.6	7.1	4.2	10.0	11.5	-0.4	1.7	12.8	1.2	-6.9	-3.2	0.0
	H	N	S	Q	D	E	T	A	P	C	K	V	I	L	F
	**B**														

***Supplementary Table 38**: Raw data Extended Data Fig. 4 – AA in water, 170 µm, 18 h, 10 mM NaCl, pH 7.4*

repeat k	species	measured values (counts*min)				concentration (µM) $[A]_{j,k,HPLC}$ calibration acc. to Supplementary Table 4				c_0 (µM)	Normalization (Eq. 1) concentration (x c_0)				Ratio species vs mean (Eq. 4) $[A]_{j,k}/\bar{c}_{j,k}-1$ (%)	
		top	II	III	bot	top	II	III	bot		top	II	III	bot	top	bot
1	H	230724	264264	345433	286340	25.70	29.44	38.48	31.90	31.38	0.82	0.94	1.23	1.02	-4.0	-9.9
	N	423090	473516	475250	484207	38.29	42.85	43.01	43.82	41.99	0.91	1.02	1.02	1.04	6.9	-7.5
	S	373347	428518	409123	441441	26.61	30.54	29.16	31.46	29.44	0.90	1.04	0.99	1.07	6.0	-5.2
	Q	350471	400003	378236	406072	30.44	34.74	32.85	35.27	33.33	0.91	1.04	0.99	1.06	7.1	-6.2
	D	350967	417314	411426	465792	31.62	37.60	37.07	41.97	37.06	0.85	1.01	1.00	1.13	0.0	0.4
	E	327726	395480	391173	451693	30.32	36.59	36.19	41.79	36.23	0.84	1.01	1.00	1.15	-1.8	2.3
	T	394040	463855	454294	509794	25.42	29.92	29.31	32.89	29.38	0.87	1.02	1.00	1.12	1.4	-0.8
	A	364563	427050	415996	467535	25.34	29.68	28.92	32.50	29.11	0.87	1.02	0.99	1.12	2.1	-1.0
	P	99060	118743	120362	141169	23.64	28.34	28.73	33.69	28.60	0.83	0.99	1.00	1.18	-3.1	4.5
	C	27580	30612	32191	37485	19.91	22.10	23.24	27.06	23.08	0.86	0.96	1.01	1.17	1.2	4.0
	K	317978	372212	362733	399125	30.09	35.22	34.32	37.76	34.35	0.88	1.03	1.00	1.10	2.7	-2.5
	V	497143	593948	622721	697719	25.87	30.91	32.40	36.30	31.37	0.82	0.99	1.03	1.16	-3.3	2.6
	I	446752	552150	567144	693409	21.39	26.43	27.15	33.20	27.04	0.79	0.98	1.00	1.23	-7.3	8.9
	L	418348	508346	520782	617461	22.90	27.82	28.50	33.80	28.25	0.81	0.98	1.01	1.20	-5.0	6.1
	F	358733	431875	434511	510095	23.57	28.38	28.55	33.52	28.50	0.83	1.00	1.00	1.18	-3.0	4.3
	mean concentration per fraction $\bar{c}_{j,k}$										0.85	1.00	1.02	1.13		
2	H	222749	258462	221123	317327	24.81	28.79	24.63	35.35	28.40	0.87	1.01	0.87	1.24	4.8	-7.5
	N	420747	479383	401584	499454	38.07	43.38	36.34	45.20	40.75	0.93	1.06	0.89	1.11	12.1	-17.6
	S	377677	439196	361282	468316	26.92	31.30	25.75	33.38	29.33	0.92	1.07	0.88	1.14	10.1	-15.5
	Q	353644	401799	332383	425535	30.72	34.90	28.87	36.96	32.86	0.93	1.06	0.88	1.12	12.1	-16.4
	D	330522	391349	342417	544351	29.78	35.26	30.85	49.04	36.23	0.82	0.97	0.85	1.35	-1.4	0.6
	E	311726	363126	322903	538164	28.84	33.60	29.88	49.80	35.53	0.81	0.95	0.84	1.40	-2.6	4.1

	T	380868	432844	381804	568785	24.57	27.92	24.63	36.69	28.45	0.86	0.98	0.87	1.29	3.6	-4.2
	A	352483	412317	352895	525566	24.50	28.66	24.53	36.53	28.56	0.86	1.00	0.86	1.28	2.9	-4.9
	P	88969	106613	95355	171988	21.24	25.45	22.76	41.05	27.62	0.77	0.92	0.82	1.49	-7.8	10.4
	C	26337	30824	26814	43786	19.02	22.26	19.36	31.61	23.06	0.82	0.97	0.84	1.37	-1.1	1.9
	K	315232	366816	310383	437843	29.83	34.71	29.37	41.43	33.83	0.88	1.03	0.87	1.22	5.8	-9.0
	V	454514	537213	487665	839222	23.65	27.95	25.38	43.67	30.16	0.78	0.93	0.84	1.45	-5.9	7.6
	I	388692	460948	429786	905726	18.61	22.07	20.58	43.36	26.15	0.71	0.84	0.79	1.66	-14.6	23.2
	L	374091	438056	409460	776960	20.47	23.98	22.41	42.52	27.35	0.75	0.88	0.82	1.56	-10.2	15.6
	F	324284	380843	347574	634141	21.31	25.02	22.84	41.67	27.71	0.77	0.90	0.82	1.50	-7.8	11.7
	mean concentration per fraction $\bar{c}_{j,k}$										0.83	0.97	0.85	1.35		
3	H	247326	233094	238582	288036	27.55	25.97	26.58	32.09	28.05	0.98	0.93	0.95	1.14	3.8	-3.2
	N	457553	422836	416762	480712	41.41	38.26	37.71	43.50	40.22	1.03	0.95	0.94	1.08	8.8	-8.5
	S	415340	375742	383445	450700	29.60	26.78	27.33	32.12	28.96	1.02	0.92	0.94	1.11	8.0	-6.1
	Q	388020	354085	350453	406586	33.70	30.76	30.44	35.32	32.55	1.04	0.94	0.94	1.08	9.4	-8.2
	D	373104	366124	381154	470714	33.62	32.99	34.34	42.41	35.84	0.94	0.92	0.96	1.18	-0.9	0.1
	E	364838	348709	366626	463637	33.76	32.27	33.92	42.90	35.71	0.95	0.90	0.95	1.20	-0.1	1.7
	T	421808	403655	418180	512779	27.21	26.04	26.98	33.08	28.33	0.96	0.92	0.95	1.17	1.5	-1.2
	A	393267	372602	384337	472311	27.34	25.90	26.72	32.83	28.20	0.97	0.92	0.95	1.16	2.4	-1.5
	P	103183	102238	108470	143334	24.63	24.40	25.89	34.21	27.28	0.90	0.89	0.95	1.25	-4.6	6.1
	C	29880	28869	29498	36824	21.57	20.84	21.30	26.59	22.58	0.96	0.92	0.94	1.18	1.0	-0.3
	K	347794	329846	338150	400990	32.91	31.21	31.99	37.94	33.51	0.98	0.93	0.95	1.13	3.7	-4.2
	V	501129	537651	541296	713218	26.08	27.98	28.17	37.11	29.83	0.87	0.94	0.94	1.24	-7.6	5.3
	I	463474	485993	533488	708325	22.19	23.27	25.54	33.91	26.23	0.85	0.89	0.97	1.29	-10.6	9.4
	L	424052	452061	481678	619331	23.21	24.74	26.36	33.90	27.05	0.86	0.91	0.97	1.25	-9.4	6.0
	F	376074	377785	407097	518372	24.71	24.82	26.75	34.06	27.59	0.90	0.90	0.97	1.23	-5.4	4.5
	mean concentration per fraction $\bar{c}_{j,k}$										0.95	0.92	0.95	1.18		

***Supplementary Table 39**: Raw data Extended Data Fig. 4 – AA in water, 170 µm, 18 h, 10 mM NaCl, pH 7.4 – enrichments in top fraction*

**repeat**		**species**	Ratio species against species (shown in heat maps), Eq. 2 $[A]_{j,k}/[B]_{j,k} - 1$ (%)														
1	**A**	H	0.0	-10.2	-9.4	-10.3	-4.0	-2.1	-5.3	-5.9	-0.9	-5.1	-6.5	-0.7	3.6	1.1	-1.0
		N	11.3	0.0	0.9	-0.2	6.9	8.9	5.4	4.7	10.3	5.7	4.1	10.6	15.3	12.5	10.3
		S	10.3	-0.9	0.0	-1.1	5.9	8.0	4.5	3.8	9.3	4.7	3.2	9.6	14.3	11.5	9.3
		Q	11.5	0.2	1.1	0.0	7.1	9.1	5.6	4.9	10.5	5.9	4.3	10.8	15.5	12.7	10.5
		D	4.2	-6.4	-5.6	-6.6	0.0	1.9	-1.4	-2.0	3.2	-1.1	-2.6	3.5	7.9	5.3	3.2
		E	2.2	-8.2	-7.4	-8.4	-1.9	0.0	-3.2	-3.8	1.3	-3.0	-4.4	1.5	5.8	3.3	1.2
		T	5.6	-5.1	-4.3	-5.3	1.4	3.3	0.0	-0.6	4.6	0.3	-1.2	4.9	9.4	6.7	4.6
		A	6.3	-4.5	-3.7	-4.7	2.0	4.0	0.6	0.0	5.3	0.9	-0.6	5.6	10.1	7.4	5.3
		P	0.9	-9.3	-8.5	-9.5	-3.1	-1.2	-4.4	-5.0	0.0	-4.2	-5.6	0.2	4.5	2.0	0.0
		C	5.3	-5.4	-4.5	-5.5	1.1	3.1	-0.3	-0.9	4.4	0.0	-1.5	4.6	9.1	6.5	4.3
		K	6.9	-3.9	-3.1	-4.1	2.7	4.6	1.3	0.6	6.0	1.5	0.0	6.2	10.8	8.1	5.9
		V	0.7	-9.6	-8.8	-9.7	-3.3	-1.5	-4.7	-5.3	-0.2	-4.4	-5.9	0.0	4.3	1.8	-0.3
		I	-3.4	-13.3	-12.5	-13.4	-7.3	-5.5	-8.6	-9.1	-4.3	-8.3	-9.7	-4.1	0.0	-2.4	-4.4
		L	-1.1	-11.1	-10.3	-11.3	-5.0	-3.2	-6.3	-6.9	-2.0	-6.1	-7.5	-1.7	2.5	0.0	-2.0
		F	1.0	-9.3	-8.5	-9.5	-3.1	-1.2	-4.4	-5.0	0.0	-4.2	-5.6	0.3	4.6	2.0	0.0
			H	N	S	Q	D	E	T	A	P	C	K	V	I	L	F
			**B**														
2	**A**	H	0.0	-6.5	-4.8	-6.5	6.3	7.6	1.2	1.8	13.7	6.0	-0.9	11.4	22.8	16.7	13.6
		N	6.9	0.0	1.8	0.0	13.7	15.1	8.2	8.9	21.5	13.3	6.0	19.2	31.3	24.8	21.5
		S	5.0	-1.8	0.0	-1.8	11.6	13.0	6.3	6.9	19.4	11.3	4.1	17.0	29.0	22.5	19.3
		Q	7.0	0.0	1.9	0.0	13.7	15.1	8.2	8.9	21.6	13.4	6.0	19.2	31.4	24.8	21.6
		D	-5.9	-12.0	-10.4	-12.1	0.0	1.2	-4.8	-4.2	6.9	-0.3	-6.8	4.8	15.5	9.8	6.9
		E	-7.1	-13.1	-11.5	-13.1	-1.2	0.0	-6.0	-5.4	5.6	-1.5	-7.9	3.5	14.1	8.4	5.6
		T	-1.2	-7.6	-5.9	-7.6	5.1	6.4	0.0	0.6	12.3	4.7	-2.1	10.1	21.4	15.3	12.3
		A	-1.8	-8.2	-6.5	-8.2	4.4	5.7	-0.6	0.0	11.6	4.1	-2.7	9.4	20.6	14.6	11.6
		P	-12.0	-17.7	-16.2	-17.8	-6.5	-5.3	-11.0	-10.4	0.0	-6.8	-12.8	-2.0	8.0	2.7	0.0
		C	-5.6	-11.8	-10.1	-11.8	0.3	1.6	-4.5	-3.9	7.3	0.0	-6.5	5.2	15.9	10.1	7.2
		K	0.9	-5.6	-3.9	-5.7	7.3	8.6	2.1	2.7	14.7	6.9	0.0	12.4	23.9	17.7	14.6
		V	-10.3	-16.1	-14.5	-16.1	-4.6	-3.4	-9.2	-8.6	2.0	-4.9	-11.1	0.0	10.2	4.7	2.0
		I	-18.6	-23.9	-22.5	-23.9	-13.4	-12.4	-17.6	-17.1	-7.4	-13.7	-19.3	-9.3	0.0	-5.0	-7.5
		L	-14.3	-19.9	-18.4	-19.9	-8.9	-7.8	-13.3	-12.7	-2.6	-9.2	-15.1	-4.5	5.2	0.0	-2.6
		F	-12.0	-17.7	-16.2	-17.7	-6.4	-5.3	-10.9	-10.4	0.0	-6.7	-12.8	-1.9	8.1	2.7	0.0
			H	N	S	Q	D	E	T	A	P	C	K	V	I	L	F
			**B**														
3	**A**	H	0.0	-4.6	-3.9	-5.1	4.7	3.9	2.3	1.3	8.8	2.8	0.0	12.4	16.1	14.5	9.7
		N	4.8	0.0	0.7	-0.6	9.8	8.9	7.2	6.2	14.0	7.7	4.8	17.8	21.7	20.0	14.9
		S	4.1	-0.7	0.0	-1.3	9.0	8.1	6.4	5.4	13.2	7.0	4.1	16.9	20.8	19.2	14.1
		Q	5.4	0.6	1.3	0.0	10.4	9.5	7.8	6.8	14.7	8.3	5.4	18.4	22.4	20.7	15.6

		D	-4.5	-8.9	-8.2	-9.4	0.0	-0.8	-2.4	-3.3	3.9	-1.8	-4.5	7.3	10.9	9.3	4.7
		E	-3.8	-8.2	-7.5	-8.7	0.8	0.0	-1.6	-2.5	4.7	-1.1	-3.7	8.1	11.7	10.2	5.5
		T	-2.2	-6.7	-6.0	-7.2	2.4	1.6	0.0	-0.9	6.4	0.5	-2.2	9.9	13.5	12.0	7.2
		A	-1.3	-5.8	-5.2	-6.4	3.4	2.6	0.9	0.0	7.4	1.5	-1.3	10.9	14.6	13.0	8.2
		P	-8.1	-12.3	-11.7	-12.8	-3.8	-4.5	-6.0	-6.9	0.0	-5.5	-8.1	3.3	6.7	5.2	0.8
		C	-2.7	-7.2	-6.5	-7.7	1.9	1.1	-0.5	-1.4	5.9	0.0	-2.7	9.3	13.0	11.4	6.7
		K	0.0	-4.6	-3.9	-5.2	4.7	3.9	2.2	1.3	8.8	2.8	0.0	12.3	16.1	14.5	9.6
		V	-11.0	-15.1	-14.5	-15.6	-6.8	-7.5	-9.0	-9.8	-3.2	-8.5	-11.0	0.0	3.3	1.9	-2.4
		I	-13.9	-17.8	-17.2	-18.3	-9.8	-10.5	-11.9	-12.7	-6.3	-11.5	-13.8	-3.2	0.0	-1.4	-5.6
		L	-12.7	-16.7	-16.1	-17.1	-8.5	-9.2	-10.7	-11.5	-5.0	-10.2	-12.6	-1.8	1.4	0.0	-4.2
		F	-8.8	-13.0	-12.4	-13.5	-4.5	-5.2	-6.7	-7.6	-0.8	-6.3	-8.8	2.5	5.9	4.4	0.0
			H	N	S	Q	D	E	T	A	P	C	K	V	I	L	F
			**B**														

***Supplementary Table 40**: Raw data Extended Data Fig. 4 – AA in water, 170 µm, 18 h, 10 mM NaCl, pH 7.4 – enrichments in bottom fraction*

**repeat**		**species**	Ratio species against species (shown in heat maps), Eq. 2 $[A]_{j,k}/[B]_{j,k} - 1$ (%)														
1	**A**	H	0.0	-2.6	-4.9	-4.0	-10.2	-11.9	-9.2	-8.9	-13.7	-13.3	-7.5	-12.2	-17.2	-15.0	-13.6
		N	2.7	0.0	-2.3	-1.4	-7.8	-9.6	-6.8	-6.5	-11.4	-11.0	-5.1	-9.8	-15.0	-12.8	-11.3
		S	5.1	2.4	0.0	1.0	-5.6	-7.4	-4.5	-4.3	-9.3	-8.9	-2.8	-7.7	-13.0	-10.7	-9.1
		Q	4.1	1.4	-1.0	0.0	-6.5	-8.3	-5.4	-5.2	-10.2	-9.7	-3.7	-8.6	-13.8	-11.5	-10.0
		D	11.4	8.5	6.0	7.0	0.0	-1.9	1.2	1.4	-3.9	-3.4	3.0	-2.2	-7.8	-5.3	-3.7
		E	13.5	10.6	8.0	9.0	1.9	0.0	3.1	3.3	-2.1	-1.6	4.9	-0.3	-6.0	-3.5	-1.9
		T	10.1	7.3	4.7	5.8	-1.2	-3.0	0.0	0.3	-5.0	-4.6	1.8	-3.3	-8.8	-6.4	-4.8
		A	9.8	7.0	4.5	5.5	-1.4	-3.2	-0.3	0.0	-5.2	-4.8	1.5	-3.5	-9.1	-6.7	-5.1
		P	15.9	12.9	10.2	11.3	4.0	2.1	5.3	5.5	0.0	0.5	7.1	1.8	-4.0	-1.5	0.2
		C	15.4	12.4	9.7	10.8	3.6	1.6	4.8	5.0	-0.5	0.0	6.7	1.3	-4.5	-2.0	-0.3
		K	8.2	5.4	2.9	3.9	-2.9	-4.7	-1.8	-1.5	-6.7	-6.2	0.0	-5.0	-10.4	-8.1	-6.5
		V	13.9	10.9	8.3	9.4	2.2	0.3	3.4	3.7	-1.8	-1.3	5.3	0.0	-5.7	-3.2	-1.6
		I	20.8	17.6	14.9	16.0	8.4	6.4	9.7	10.0	4.2	4.7	11.6	6.1	0.0	2.6	4.4
		L	17.7	14.6	11.9	13.0	5.6	3.7	6.9	7.1	1.5	2.0	8.8	3.4	-2.6	0.0	1.7
		F	15.7	12.7	10.0	11.1	3.8	1.9	5.1	5.3	-0.2	0.3	6.9	1.6	-4.2	-1.7	0.0
			H	N	S	Q	D	E	T	A	P	C	K	V	I	L	F
			**B**														
2	**A**	H	0.0	12.2	9.4	10.7	-8.0	-11.2	-3.5	-2.7	-16.2	-9.2	1.7	-14.0	-24.9	-19.9	-17.2
		N	-10.9	0.0	-2.5	-1.4	-18.1	-20.9	-14.0	-13.3	-25.4	-19.1	-9.4	-23.4	-33.1	-28.7	-26.2
		S	-8.6	2.6	0.0	1.2	-15.9	-18.8	-11.8	-11.1	-23.4	-17.0	-7.1	-21.4	-31.4	-26.8	-24.3
		Q	-9.6	1.4	-1.1	0.0	-16.9	-19.7	-12.8	-12.1	-24.3	-18.0	-8.1	-22.3	-32.2	-27.7	-25.2
		D	8.7	22.0	19.0	20.3	0.0	-3.4	5.0	5.8	-8.9	-1.3	10.5	-6.5	-18.4	-13.0	-10.0
		E	12.6	26.4	23.2	24.6	3.5	0.0	8.7	9.6	-5.7	2.2	14.5	-3.2	-15.5	-9.9	-6.8
		T	3.6	16.3	13.3	14.7	-4.7	-8.0	0.0	0.8	-13.2	-5.9	5.3	-10.9	-22.2	-17.1	-14.2
		A	2.8	15.3	12.4	13.7	-5.5	-8.7	-0.8	0.0	-13.9	-6.7	4.5	-11.6	-22.8	-17.7	-14.9
		P	19.4	34.0	30.6	32.1	9.8	6.0	15.2	16.2	0.0	8.4	21.4	2.6	-10.4	-4.4	-1.2
		C	10.1	23.6	20.5	21.9	1.3	-2.2	6.3	7.2	-7.8	0.0	12.0	-5.3	-17.3	-11.8	-8.8
		K	-1.6	10.4	7.6	8.9	-9.5	-12.6	-5.0	-4.3	-17.6	-10.7	0.0	-15.4	-26.1	-21.3	-18.6
		V	16.3	30.5	27.3	28.7	7.0	3.3	12.3	13.2	-2.6	5.6	18.2	0.0	-12.7	-6.9	-3.7
		I	33.2	49.5	45.7	47.4	22.5	18.3	28.6	29.6	11.6	20.9	35.4	14.5	0.0	6.6	10.3
		L	24.9	40.2	36.7	38.3	14.9	11.0	20.6	21.6	4.6	13.4	27.0	7.4	-6.2	0.0	3.4
		F	20.8	35.6	32.2	33.7	11.1	7.3	16.6	17.5	1.2	9.7	22.8	3.9	-9.3	-3.3	0.0
			H	N	S	Q	D	E	T	A	P	C	K	V	I	L	F
			**B**														
3	**A**	H	0.0	5.8	3.1	5.5	-3.3	-4.8	-2.0	-1.7	-8.8	-2.9	1.1	-8.0	-11.5	-8.7	-7.3
		N	-5.5	0.0	-2.5	-0.3	-8.6	-10.0	-7.4	-7.1	-13.7	-8.2	-4.5	-13.1	-16.4	-13.7	-12.4
		S	-3.0	2.6	0.0	2.3	-6.3	-7.7	-5.0	-4.7	-11.5	-5.8	-2.0	-10.8	-14.2	-11.5	-10.2
		Q	-5.2	0.3	-2.2	0.0	-8.3	-9.7	-7.1	-6.8	-13.5	-7.9	-4.2	-12.8	-16.1	-13.4	-12.1

		D	3.4	9.4	6.7	9.1	0.0	-1.5	1.3	1.6	-5.6	0.5	4.5	-4.9	-8.5	-5.6	-4.2
		E	5.0	11.1	8.3	10.7	1.5	0.0	2.9	3.2	-4.2	2.0	6.1	-3.4	-7.1	-4.1	-2.7
		T	2.1	8.0	5.3	7.6	-1.3	-2.8	0.0	0.3	-6.9	-0.8	3.2	-6.1	-9.7	-6.8	-5.4
		A	1.8	7.7	5.0	7.3	-1.6	-3.1	-0.3	0.0	-7.1	-1.1	2.9	-6.4	-9.9	-7.1	-5.7
		P	9.6	15.9	13.0	15.6	6.0	4.4	7.4	7.7	0.0	6.5	10.8	0.8	-3.0	0.1	1.6
		C	2.9	8.9	6.2	8.6	-0.5	-2.0	0.8	1.1	-6.1	0.0	4.0	-5.3	-8.9	-6.0	-4.6
		K	-1.0	4.7	2.1	4.4	-4.3	-5.8	-3.1	-2.8	-9.7	-3.9	0.0	-9.0	-12.4	-9.6	-8.3
		V	8.7	15.0	12.1	14.7	5.1	3.6	6.5	6.8	-0.8	5.6	9.9	0.0	-3.8	-0.7	0.8
		I	13.0	19.5	16.6	19.2	9.3	7.6	10.7	11.0	3.1	9.8	14.2	3.9	0.0	3.2	4.7
		L	9.5	15.9	13.0	15.5	5.9	4.3	7.3	7.6	-0.1	6.4	10.7	0.7	-3.1	0.0	1.5
		F	7.9	14.2	11.3	13.8	4.3	2.8	5.7	6.0	-1.5	4.8	9.1	-0.7	-4.5	-1.5	0.0
			H	N	S	Q	D	E	T	A	P	C	K	V	I	L	F
			**B**														

***Supplementary Table 41**: Raw data Extended Data Fig. 4 – AA in water, 170 µm, 18 h, 100 mM NaCl, pH 7.4*

repeat k	species	measured values (counts*min)				concentration (µM) $[A]_{j,k,HPLC}$ calibration acc. to Supplementary Table 4				c_0 (µM)	Normalization (Eq. 1) concentration (x c_0)				Ratio species vs mean (Eq. 4) $[A]_{j,k}/\bar{c}_{j,k}-1$ (%)	
		top	II	III	bot	top	II	III	bot		top	II	III	bot	top	bot
1	H	224736	226101	237934	273864	25.04	25.19	26.51	30.51	26.81	0.93	0.94	0.99	1.14	4.9	-2.7
	N	404928	436605	422385	468544	36.64	39.51	38.22	42.40	39.19	0.93	1.01	0.98	1.08	5.0	-7.5
	S	355640	356018	377900	428608	25.35	25.37	26.93	30.55	27.05	0.94	0.94	1.00	1.13	5.2	-3.4
	Q	332795	333915	346837	401403	28.91	29.00	30.13	34.87	30.73	0.94	0.94	0.98	1.13	5.6	-3.0
	D	339283	355321	375212	442294	30.57	32.01	33.81	39.85	34.06	0.90	0.94	0.99	1.17	0.8	0.1
	E	319897	336739	356551	423171	29.60	31.16	32.99	39.16	33.23	0.89	0.94	0.99	1.18	0.0	0.8
	T	378197	391071	412741	485274	24.40	25.23	26.63	31.30	26.89	0.91	0.94	0.99	1.16	1.9	-0.4
	A	351376	357707	382887	442518	24.42	24.86	26.61	30.76	26.67	0.92	0.93	1.00	1.15	2.8	-1.4
	P	94762	100523	109214	129013	22.62	23.99	26.07	30.79	25.87	0.87	0.93	1.01	1.19	-1.8	1.8
	C	26993	28332	30802	36403	19.49	20.46	22.24	26.28	22.12	0.88	0.92	1.01	1.19	-1.1	1.6
	K	308859	318081	332711	388763	29.22	30.10	31.48	36.78	31.90	0.92	0.94	0.99	1.15	2.9	-1.4
	V	442620	546853	568909	693889	23.03	28.45	29.60	36.11	29.30	0.79	0.97	1.01	1.23	-11.7	5.4
	I	438559	482326	521829	634442	21.00	23.09	24.98	30.37	24.86	0.84	0.93	1.00	1.22	-5.2	4.5
	L	390733	447505	474751	565271	21.39	24.49	25.98	30.94	25.70	0.83	0.95	1.01	1.20	-6.6	2.9
	F	344278	371835	396976	477338	22.62	24.43	26.08	31.37	26.13	0.87	0.94	1.00	1.20	-2.8	2.7
	mean concentration per fraction $\bar{c}_{j,k}$										0.89	0.94	1.00	1.17		
2	H	173643	234142	269606	326096	19.34	26.08	30.03	36.33	27.95	0.69	0.93	1.07	1.30	7.7	-8.3
	N	344979	429659	478056	536715	31.22	38.88	43.26	48.57	40.48	0.77	0.96	1.07	1.20	20.0	-15.4
	S	297362	379885	431805	484546	21.19	27.07	30.77	34.53	28.39	0.75	0.95	1.08	1.22	16.2	-14.2
	Q	276582	354620	395115	456990	24.02	30.80	34.32	39.69	32.21	0.75	0.96	1.07	1.23	16.1	-13.1
	D	253423	346537	415954	557602	22.83	31.22	37.48	50.24	35.44	0.64	0.88	1.06	1.42	0.3	0.0
	E	230767	322996	395515	543256	21.35	29.89	36.60	50.27	34.53	0.62	0.87	1.06	1.46	-3.7	2.7

	T	285932	386391	461744	600370	18.44	24.93	29.79	38.73	27.97	0.66	0.89	1.06	1.38	2.6	-2.3
	A	268712	360876	438305	542960	18.68	25.08	30.47	37.74	27.99	0.67	0.90	1.09	1.35	3.9	-4.9
	P	63488	92714	117230	174168	15.15	22.13	27.98	41.57	26.71	0.57	0.83	1.05	1.56	-11.7	9.8
	C	18765	26449	33121	45501	13.55	19.10	23.91	32.85	22.35	0.61	0.85	1.07	1.47	-5.7	3.7
	K	248476	332383	379532	458116	23.51	31.45	35.91	43.35	33.55	0.70	0.94	1.07	1.29	9.1	-8.9
	V	327178	472185	612570	870233	17.02	24.57	31.87	45.28	29.69	0.57	0.83	1.07	1.53	-10.7	7.6
	I	273433	403674	532452	881870	13.09	19.33	25.49	42.22	25.03	0.52	0.77	1.02	1.69	-18.6	19.0
	L	261361	380338	494177	771590	14.30	20.82	27.05	42.23	26.10	0.55	0.80	1.04	1.62	-14.7	14.1
	F	233426	336543	423390	634890	15.34	22.11	27.82	41.72	26.75	0.57	0.83	1.04	1.56	-10.7	10.0
	mean concentration per fraction $\bar{c}_{j,k}$										0.64	0.88	1.06	1.42		
3	H	155995	225111	245373	293789	17.38	25.08	27.33	32.73	25.63	0.68	0.98	1.07	1.28	11.8	-6.7
	N	324330	410737	435242	495953	29.35	37.17	39.39	44.88	37.70	0.78	0.99	1.04	1.19	28.4	-13.0
	S	283227	364140	405855	449923	20.18	25.95	28.92	32.06	26.78	0.75	0.97	1.08	1.20	24.3	-12.5
	Q	258978	338409	355964	417675	22.50	29.39	30.92	36.28	29.77	0.76	0.99	1.04	1.22	24.6	-11.0
	D	210859	338058	370607	483813	19.00	30.46	33.39	43.59	31.61	0.60	0.96	1.06	1.38	-0.9	0.8
	E	183648	317885	360272	470097	16.99	29.41	33.34	43.50	30.81	0.55	0.95	1.08	1.41	-9.0	3.1
	T	246518	377683	415470	526726	15.90	24.36	26.80	33.98	25.26	0.63	0.96	1.06	1.35	3.8	-1.7
	A	240848	351908	394736	482064	16.74	24.46	27.44	33.51	25.54	0.66	0.96	1.07	1.31	8.1	-4.1
	P	53026	92516	102989	147209	12.66	22.08	24.58	35.14	23.61	0.54	0.94	1.04	1.49	-11.6	8.7
	C	16681	26162	28500	39401	12.04	18.89	20.58	28.45	19.99	0.60	0.94	1.03	1.42	-0.6	4.0
	K	221656	318955	341834	410221	20.97	30.18	32.34	38.81	30.58	0.69	0.99	1.06	1.27	13.1	-7.3
	V	219269	549843	499216	680948	11.41	28.61	25.98	35.43	25.36	0.45	1.13	1.02	1.40	-25.8	2.1
	I	198314	405014	462682	709182	9.49	19.39	22.15	33.95	21.25	0.45	0.91	1.04	1.60	-26.3	16.7
	L	183861	393892	421281	617319	10.06	21.56	23.06	33.79	22.12	0.46	0.97	1.04	1.53	-24.9	11.6
	F	182147	333957	371674	530613	11.97	21.94	24.42	34.87	23.30	0.51	0.94	1.05	1.50	-15.3	9.3
	mean concentration per fraction $\bar{c}_{j,k}$										0.61	0.97	1.05	1.37		

Supplementary Table 42*: Raw data Extended Data Fig. 4 – AA in water, 170 µm, 18 h, 100 mM NaCl, pH 7.4 – enrichments in top fraction*

repeat		species	Ratio species against species (shown in heat maps), Eq. 2 $[A]_{j,k}/[B]_{j,k} - 1$ (%)														
		H	0.0	-0.1	-0.3	-0.7	4.0	4.8	2.9	2.0	6.8	6.0	1.9	18.8	10.6	12.2	7.8
		N	0.1	0.0	-0.2	-0.6	4.2	4.9	3.0	2.1	6.9	6.1	2.0	18.9	10.7	12.4	8.0
		S	0.3	0.2	0.0	-0.4	4.4	5.2	3.3	2.3	7.2	6.3	2.3	19.2	11.0	12.6	8.2
		Q	0.7	0.6	0.4	0.0	4.8	5.6	3.7	2.7	7.6	6.8	2.7	19.7	11.4	13.1	8.7
		D	-3.9	-4.0	-4.2	-4.6	0.0	0.7	-1.1	-2.0	2.6	1.9	-2.0	14.2	6.3	7.9	3.7
		E	-4.6	-4.7	-4.9	-5.3	-0.7	0.0	-1.8	-2.7	1.9	1.1	-2.8	13.3	5.5	7.1	2.9
		T	-2.8	-3.0	-3.2	-3.6	1.1	1.9	0.0	-0.9	3.8	3.0	-1.0	15.4	7.4	9.0	4.8
	**A**	A	-1.9	-2.0	-2.3	-2.6	2.1	2.8	0.9	0.0	4.8	3.9	0.0	16.5	8.5	10.1	5.8
1		P	-6.4	-6.5	-6.7	-7.1	-2.6	-1.9	-3.6	-4.5	0.0	-0.8	-4.6	11.2	3.5	5.1	1.0
		C	-5.6	-5.7	-6.0	-6.3	-1.8	-1.1	-2.9	-3.8	0.8	0.0	-3.8	12.1	4.3	5.9	1.8
		K	-1.9	-2.0	-2.2	-2.6	2.1	2.8	1.0	0.0	4.8	4.0	0.0	16.6	8.5	10.1	5.8
		V	-15.8	-15.9	-16.1	-16.4	-12.4	-11.8	-13.4	-14.2	-10.1	-10.8	-14.2	0.0	-6.9	-5.5	-9.2
		I	-9.6	-9.7	-9.9	-10.2	-5.9	-5.2	-6.9	-7.8	-3.4	-4.2	-7.8	7.4	0.0	1.5	-2.5
		L	-10.9	-11.0	-11.2	-11.6	-7.3	-6.6	-8.3	-9.2	-4.8	-5.6	-9.2	5.9	-1.5	0.0	-3.9
		F	-7.3	-7.4	-7.6	-8.0	-3.5	-2.8	-4.6	-5.5	-1.0	-1.7	-5.5	10.2	2.5	4.1	0.0
			H	N	S	Q	D	E	T	A	P	C	K	V	I	L	F
			**B**														
		H	0.0	-10.2	-7.3	-7.2	7.4	11.9	5.0	3.7	22.0	14.2	-1.2	20.7	32.4	26.3	20.7
		N	11.4	0.0	3.3	3.4	19.7	24.7	16.9	15.6	35.9	27.2	10.1	34.5	47.5	40.7	34.5
		S	7.8	-3.2	0.0	0.1	15.9	20.7	13.2	11.9	31.6	23.1	6.5	30.2	42.7	36.2	30.2
		Q	7.8	-3.3	-0.1	0.0	15.8	20.6	13.1	11.8	31.5	23.1	6.4	30.1	42.6	36.1	30.1
		D	-6.9	-16.5	-13.7	-13.6	0.0	4.2	-2.3	-3.5	13.5	6.3	-8.1	12.3	23.2	17.5	12.3
		E	-10.6	-19.8	-17.1	-17.1	-4.0	0.0	-6.2	-7.3	9.0	2.0	-11.7	7.8	18.3	12.8	7.9
		T	-4.7	-14.5	-11.7	-11.6	2.4	6.6	0.0	-1.2	16.2	8.8	-5.9	15.0	26.1	20.3	15.0
	**A**	A	-3.6	-13.5	-10.6	-10.5	3.6	7.9	1.2	0.0	17.6	10.1	-4.8	16.4	27.6	21.7	16.4
2		P	-18.0	-26.4	-24.0	-23.9	-11.9	-8.3	-14.0	-15.0	0.0	-6.4	-19.0	-1.1	8.5	3.5	-1.1
		C	-12.4	-21.4	-18.8	-18.7	-5.9	-2.0	-8.1	-9.2	6.8	0.0	-13.5	5.7	15.9	10.6	5.7
		K	1.2	-9.1	-6.1	-6.1	8.8	13.3	6.3	5.0	23.5	15.6	0.0	22.2	34.0	27.8	22.2
		V	-17.2	-25.6	-23.2	-23.1	-11.0	-7.3	-13.0	-14.1	1.1	-5.4	-18.2	0.0	9.7	4.6	0.0
		I	-24.4	-32.2	-29.9	-29.9	-18.8	-15.4	-20.7	-21.6	-7.8	-13.7	-25.4	-8.8	0.0	-4.6	-8.8
		L	-20.8	-28.9	-26.6	-26.5	-14.9	-11.4	-16.9	-17.9	-3.4	-9.6	-21.8	-4.4	4.8	0.0	-4.4
		F	-17.2	-25.6	-23.2	-23.1	-11.0	-7.3	-13.0	-14.1	1.1	-5.4	-18.2	0.0	9.7	4.6	0.0
			H	N	S	Q	D	E	T	A	P	C	K	V	I	L	F
			**B**														
		H	0.0	-12.9	-10.0	-10.3	12.8	22.9	7.7	3.4	26.5	12.5	-1.1	50.7	51.7	49.0	32.0
3	**A**	N	14.8	0.0	3.3	3.0	29.5	41.2	23.7	18.8	45.3	29.2	13.5	73.0	74.2	71.1	51.6
		S	11.2	-3.2	0.0	-0.2	25.4	36.7	19.7	15.0	40.6	25.1	9.9	67.5	68.7	65.6	46.7
		Q	11.4	-3.0	0.2	0.0	25.7	37.0	20.0	15.3	41.0	25.4	10.2	67.9	69.1	66.1	47.1

		D	-11.4	-22.8	-20.3	-20.5	0.0	9.0	-4.5	-8.3	12.1	-0.2	-12.4	33.6	34.5	32.1	17.0
		E	-18.7	-29.2	-26.8	-27.0	-8.2	0.0	-12.4	-15.9	2.9	-8.5	-19.6	22.6	23.4	21.2	7.4
		T	-7.2	-19.1	-16.5	-16.7	4.7	14.1	0.0	-4.0	17.5	4.5	-8.2	39.9	40.9	38.4	22.6
		A	-3.3	-15.8	-13.0	-13.2	9.1	18.9	4.1	0.0	22.3	8.8	-4.4	45.7	46.7	44.1	27.6
		P	-21.0	-31.2	-28.9	-29.1	-10.8	-2.8	-14.9	-18.2	0.0	-11.0	-21.9	19.1	19.9	17.8	4.3
		C	-11.1	-22.6	-20.1	-20.3	0.2	9.2	-4.3	-8.1	12.4	0.0	-12.2	33.9	34.8	32.4	17.3
		K	1.2	-11.9	-9.0	-9.2	14.1	24.4	9.0	4.6	28.0	13.8	0.0	52.4	53.5	50.7	33.5
		V	-33.6	-42.2	-40.3	-40.4	-25.1	-18.4	-28.5	-31.4	-16.0	-25.3	-34.4	0.0	0.7	-1.1	-12.4
		I	-34.1	-42.6	-40.7	-40.9	-25.6	-19.0	-29.0	-31.8	-16.6	-25.8	-34.8	-0.7	0.0	-1.8	-13.0
		L	-32.9	-41.6	-39.6	-39.8	-24.3	-17.5	-27.7	-30.6	-15.1	-24.5	-33.7	1.1	1.8	0.0	-11.4
		F	-24.2	-34.0	-31.8	-32.0	-14.5	-6.9	-18.4	-21.6	-4.2	-14.7	-25.1	14.2	15.0	12.9	0.0
			H	N	S	Q	D	E	T	A	P	C	K	V	I	L	F
			**B**														

***Supplementary Table 43**: Raw data Extended Data Fig. 4 – AA in water, 170 µm, 18 h, 100 mM NaCl, pH 7.4 – enrichments in bottom fraction*

repeat		species	Ratio species against species (shown in heat maps), Eq. 2 $[A]_{j,k}/[B]_{j,k} - 1$ (%)														
1	**A**	H	0.0	5.2	0.8	0.3	-2.7	-3.4	-2.3	-1.3	-4.4	-4.2	-1.3	-7.7	-6.9	-5.5	-5.2
		N	-4.9	0.0	-4.2	-4.7	-7.5	-8.2	-7.1	-6.2	-9.1	-9.0	-6.2	-12.2	-11.5	-10.1	-9.9
		S	-0.8	4.4	0.0	-0.5	-3.5	-4.2	-3.0	-2.1	-5.1	-5.0	-2.1	-8.4	-7.6	-6.2	-5.9
		Q	-0.3	4.9	0.5	0.0	-3.0	-3.7	-2.5	-1.6	-4.7	-4.5	-1.6	-7.9	-7.1	-5.7	-5.5
		D	2.8	8.2	3.6	3.1	0.0	-0.7	0.5	1.4	-1.7	-1.5	1.5	-5.1	-4.2	-2.8	-2.5
		E	3.6	8.9	4.4	3.9	0.7	0.0	1.2	2.2	-1.0	-0.8	2.2	-4.4	-3.5	-2.1	-1.8
		T	2.3	7.6	3.1	2.6	-0.5	-1.2	0.0	0.9	-2.2	-2.0	1.0	-5.5	-4.7	-3.3	-3.0
		A	1.4	6.6	2.1	1.7	-1.4	-2.1	-0.9	0.0	-3.1	-2.9	0.0	-6.4	-5.6	-4.2	-3.9
		P	4.6	10.0	5.4	4.9	1.7	1.0	2.2	3.2	0.0	0.2	3.2	-3.4	-2.6	-1.1	-0.8
		C	4.4	9.9	5.2	4.7	1.6	0.8	2.1	3.0	-0.2	0.0	3.0	-3.6	-2.7	-1.3	-1.0
		K	1.3	6.6	2.1	1.6	-1.4	-2.1	-0.9	0.0	-3.1	-3.0	0.0	-6.4	-5.6	-4.2	-3.9
		V	8.3	13.9	9.1	8.6	5.3	4.6	5.9	6.8	3.5	3.7	6.9	0.0	0.9	2.4	2.6
		I	7.4	12.9	8.2	7.7	4.4	3.7	4.9	5.9	2.6	2.8	5.9	-0.9	0.0	1.5	1.8
		L	5.8	11.3	6.6	6.1	2.9	2.2	3.4	4.4	1.1	1.3	4.4	-2.3	-1.5	0.0	0.3
		F	5.5	11.0	6.3	5.8	2.6	1.9	3.1	4.1	0.9	1.0	4.1	-2.6	-1.7	-0.3	0.0
			H	N	S	Q	D	E	T	A	P	C	K	V	I	L	F
			**B**														
2	**A**	H	0.0	8.3	6.9	5.5	-8.3	-10.7	-6.1	-3.6	-16.5	-11.6	0.6	-14.8	-22.9	-19.7	-16.7
		N	-7.7	0.0	-1.4	-2.6	-15.4	-17.6	-13.3	-11.0	-22.9	-18.4	-7.1	-21.3	-28.9	-25.9	-23.1
		S	-6.4	1.4	0.0	-1.3	-14.2	-16.5	-12.2	-9.8	-21.9	-17.2	-5.9	-20.3	-27.9	-24.8	-22.0
		Q	-5.2	2.7	1.3	0.0	-13.1	-15.4	-11.0	-8.6	-20.8	-16.2	-4.6	-19.2	-26.9	-23.8	-21.0
		D	9.0	18.1	16.5	15.0	0.0	-2.6	2.4	5.1	-8.9	-3.6	9.7	-7.1	-16.0	-12.4	-9.1
		E	12.0	21.4	19.7	18.1	2.7	0.0	5.2	8.0	-6.5	-0.9	12.7	-4.5	-13.7	-10.0	-6.7
		T	6.5	15.4	13.8	12.4	-2.3	-4.9	0.0	2.7	-11.0	-5.8	7.2	-9.2	-17.9	-14.4	-11.2
		A	3.7	12.4	10.9	9.4	-4.9	-7.4	-2.6	0.0	-13.4	-8.3	4.4	-11.6	-20.1	-16.7	-13.6
		P	19.7	29.7	28.0	26.3	9.8	6.9	12.4	15.4	0.0	5.9	20.5	2.0	-7.7	-3.8	-0.2
		C	13.1	22.5	20.8	19.3	3.7	0.9	6.1	9.0	-5.6	0.0	13.8	-3.6	-12.9	-9.2	-5.8
		K	-0.6	7.7	6.2	4.8	-8.9	-11.3	-6.7	-4.2	-17.0	-12.1	0.0	-15.3	-23.4	-20.2	-17.2
		V	17.3	27.1	25.4	23.8	7.6	4.8	10.2	13.1	-2.0	3.8	18.1	0.0	-9.6	-5.7	-2.2
		I	29.8	40.6	38.7	36.9	19.0	15.8	21.8	25.1	8.4	14.8	30.6	10.6	0.0	4.2	8.1
		L	24.5	34.9	33.0	31.3	14.2	11.1	16.9	20.0	4.0	10.1	25.3	6.1	-4.1	0.0	3.7
		F	20.0	30.0	28.2	26.6	10.0	7.1	12.6	15.7	0.2	6.1	20.7	2.3	-7.5	-3.6	0.0
			H	N	S	Q	D	E	T	A	P	C	K	V	I	L	F
			**B**														
3	**A**	H	0.0	7.3	6.7	4.8	-7.4	-9.6	-5.1	-2.7	-14.2	-10.3	0.6	-8.6	-20.1	-16.4	-14.7
		N	-6.8	0.0	-0.6	-2.3	-13.7	-15.7	-11.5	-9.3	-20.0	-16.3	-6.2	-14.8	-25.5	-22.1	-20.4
		S	-6.2	0.6	0.0	-1.7	-13.2	-15.2	-11.0	-8.8	-19.5	-15.9	-5.7	-14.3	-25.1	-21.6	-20.0
		Q	-4.6	2.4	1.8	0.0	-11.6	-13.7	-9.4	-7.1	-18.1	-14.4	-4.0	-12.8	-23.7	-20.2	-18.6

		D	8.0	15.8	15.2	13.2	0.0	-2.3	2.5	5.1	-7.3	-3.1	8.6	-1.3	-13.7	-9.7	-7.8
		E	10.6	18.6	17.9	15.9	2.4	0.0	5.0	7.6	-5.1	-0.8	11.2	1.0	-11.7	-7.6	-5.7
		T	5.3	13.0	12.3	10.4	-2.5	-4.7	0.0	2.5	-9.6	-5.5	6.0	-3.7	-15.8	-12.0	-10.1
		A	2.8	10.2	9.6	7.7	-4.9	-7.1	-2.4	0.0	-11.8	-7.8	3.4	-6.1	-17.9	-14.1	-12.3
		P	16.5	25.0	24.3	22.1	7.9	5.4	10.6	13.4	0.0	4.6	17.2	6.5	-6.9	-2.6	-0.6
		C	11.4	19.5	18.9	16.8	3.2	0.8	5.8	8.5	-4.4	0.0	12.1	1.8	-10.9	-6.8	-4.9
		K	-0.6	6.6	6.0	4.2	-8.0	-10.1	-5.6	-3.3	-14.7	-10.8	0.0	-9.2	-20.6	-16.9	-15.2
		V	9.4	17.4	16.7	14.7	1.3	-1.0	3.9	6.5	-6.1	-1.8	10.1	0.0	-12.6	-8.5	-6.6
		I	25.1	34.2	33.5	31.1	15.9	13.2	18.8	21.8	7.4	12.3	25.9	14.4	0.0	4.6	6.8
		L	19.6	28.3	27.6	25.4	10.8	8.2	13.6	16.4	2.7	7.3	20.3	9.3	-4.4	0.0	2.1
		F	17.2	25.7	25.0	22.8	8.5	6.0	11.2	14.0	0.6	5.1	17.9	7.1	-6.4	-2.0	0.0
			H	N	S	Q	D	E	T	A	P	C	K	V	I	L	F
			**B**														

***Supplementary Table 44**: Raw data Extended Data Fig. 5b – nucleobases in 10 % formamide, 170µm, 18h*

repeat k	species	measured values (µAU*min)				concentration (µM) $[A]_{j,k,HPLC}$ calibration acc. to Supplementary Table 3				c_0 (µM)	Normalization (Eq. 1) concentration (x c_0)				Ratio species vs mean (Eq. 4) $[A]_{j,k}/\bar{c}_{j,k} - 1$ (%)		Ratio species against species (shown in heat maps), Eq. 2 $[A]_{j,k}/[B]_{j,k} - 1$ (%)											
		top	II	III	bot	top	II	III	bot		top	II	III	bot	top	bot	top part (blue shade)						bot part (red shade)					
	C	120.45	545.29	504.07	1746.04	2.65	11.99	11.08	38.39	16.03	0.17	0.75	0.69	2.4	-23.42	23.7	C	0	-25.49	-38.97	-27.87	-13.97	C	0	33.41	49.42	39.49	10.34
	A	496.26	2364.1	2072.88	4017.41	4.37	20.81	18.24	35.36	19.69	0.22	1.06	0.93	1.8	2.78	-7.28	A	34.22	0	-18.08	-3.19	15.46	A	-25.04	0	12	4.56	-17.3
1	G	228.53	954.64	840.15	1353.19	10.59	44.23	38.92	62.69	39.11	0.27	1.13	1	1.6	25.47	-17.21	G	63.84	22.07	0	18.18	40.95	G	-33.07	-10.71	0	-6.64	-26.15
	U	299.94	1412.76	1276.39	2248.26	4.15	19.57	17.68	31.14	18.14	0.23	1.08	0.97	1.72	6.16	-11.32	U	38.64	3.29	-15.39	0	19.26	U	-28.31	-4.36	7.11	0	-20.9
	T	242.59	1076.72	990.92	2741.81	3.81	16.91	15.56	43.06	19.84	0.19	0.85	0.78	2.17	-10.99	12.11	T	16.24	-13.39	-29.05	-16.15	0	T	-9.37	20.91	35.42	26.43	0
	mean concentration per fraction $\bar{c}_{j,k}$										0.22	0.97	0.87	1.94				C	A	G	U	T		C	A	G	U	T
	C	128.75	293.36	496.25	1789.68	2.83	6.45	10.91	39.35	14.88	0.19	0.43	0.73	2.64	-36.9	19.3	C	0	-44.61	-51.66	-44.09	-20.73	C	0	26.84	39.03	30.08	9.33
	A	801.53	1542.57	2128.69	4865.74	7.05	13.58	18.73	42.82	20.55	0.34	0.66	0.91	2.08	13.91	-5.94	A	80.53	0	-12.74	0.93	43.1	A	-21.16	0	9.61	2.55	-13.8
2	G	359.24	683.84	873.09	1736.06	16.64	31.68	40.45	80.43	42.3	0.39	0.75	0.96	1.9	30.54	-14.19	G	106.89	14.6	0	15.67	63.99	G	-28.07	-8.77	0	-6.44	-21.36
	U	472.73	945.11	1316.87	2824.35	6.55	13.09	18.24	39.12	19.25	0.34	0.68	0.95	2.03	12.85	-8.29	U	78.86	-0.92	-13.55	0	41.78	U	-23.12	-2.49	6.88	0	-15.95
	T	298.07	649.75	1017.76	3004	4.68	10.2	15.98	47.18	19.51	0.24	0.52	0.82	2.42	-20.4	9.12	T	26.16	-30.12	-39.02	-29.47	0	T	-8.53	16.01	27.17	18.98	0
	mean concentration per fraction $\bar{c}_{j,k}$										0.3	0.61	0.87	2.22				C	A	G	U	T		C	A	G	U	T
	C	138.48	209.22	425.07	2262.85	3.04	4.6	9.35	49.75	16.68	0.18	0.28	0.56	2.98	-28.46	9.32	C	0	-31.85	-43.7	-34.93	-17.24	C	0	11.39	18.4	13.45	5.28
	A	627.19	814.02	1658.42	6270.06	5.52	7.16	14.6	55.18	20.62	0.27	0.35	0.71	2.68	4.98	-1.86	A	46.74	0	-17.39	-4.52	21.44	A	-10.23	0	6.29	1.84	-5.49
3	G	273.61	338.08	639.11	2125.81	12.68	15.66	29.61	98.49	39.11	0.32	0.4	0.76	2.52	27.08	-7.67	G	77.63	21.05	0	15.58	47.01	G	-15.54	-5.92	0	-4.19	-11.08
	U	385.13	495.26	1003.47	3609.56	5.33	6.86	13.9	50	19.02	0.28	0.36	0.73	2.63	9.95	-3.64	U	53.69	4.74	-13.48	0	27.19	U	-11.85	-1.81	4.37	0	-7.2
	T	291.5	451.55	800.99	3744.4	4.58	7.09	12.58	58.81	20.76	0.22	0.34	0.61	2.83	-13.55	3.84	T	20.83	-17.65	-31.98	-21.38	0	T	-5.02	5.81	12.46	7.76	0
	mean concentration per fraction $\bar{c}_{j,k}$										0.26	0.35	0.67	2.73				C	A	G	U	T		C	A	G	U	T

***Supplementary Table 45**: Raw data Extended Data Fig. 5c – nucleobases in 100 mM phosphate buffer, 170µm, 18h*

repeat k	species	measured values (µAU*s)				concentration (µM) $[A]_{j,k,HPLC}$ calibration acc. to Supplementary Table 3				c_0 (µM)	Normalization (Eq. 1) concentration (x c_0)				Ratio species vs mean (Eq. 4) $[A]_{j,k}/\bar{c}_{j,k}-1$ (%)		Ratio species against species (shown in heat maps), Eq. 2 $[A]_{j,k}/[B]_{j,k}-1$ (%)											
		top	II	III	bot	top	II	III	bot		top	II	III	bot	top	bot	top part (blue shade)						bot part (red shade)					
1	C	13614	28278	59087	168060	4.99	10.36	21.65	61.58	24.65	0.2	0.42	0.88	2.5	-46.09	23.89	C	0	-62.42	-55.78	-54.77	-12.4	C	0	49.8	42.67	38.45	5.83
	A	97457	135393	189061	301763	14.3	19.86	27.73	44.26	26.54	0.54	0.75	1.05	1.67	43.46	-17.3	A	166.13	0	17.68	20.37	133.14	A	-33.25	0	-4.76	-7.58	-29.36
	G	24668	33469	63044	94385	19.05	25.84	48.68	72.88	41.61	0.46	0.62	1.17	1.75	21.91	-13.16	G	126.14	-15.03	0	2.28	98.11	G	-29.91	5	0	-2.96	-25.82
	U	49084	75640	116042	197946	11.33	17.46	26.79	45.7	25.32	0.45	0.69	1.06	1.8	19.19	-10.51	U	121.1	-16.92	-2.23	0	93.69	U	-27.77	8.2	3.05	0	-23.56
	T	26945	54564	109621	275339	7.05	14.28	28.69	72.07	30.53	0.23	0.47	0.94	2.36	-38.46	17.07	T	14.15	-57.11	-49.52	-48.37	0	T	-5.51	41.55	34.81	30.82	0
	mean concentration per fraction $\bar{c}_{j,k}$										0.38	0.59	1.02	2.02				C	A	G	U	T		C	A	G	U	T
2	C	29329	44746	68171	181643	10.75	16.4	24.98	66.56	29.67	0.36	0.55	0.84	2.24	-28.63	23.39	C	0	-47.1	-26.32	-41.53	-4.57	C	0	47.98	44.86	37.55	4.26
	A	138311	165949	197499	306220	20.29	24.34	28.97	44.92	29.63	0.68	0.82	0.98	1.52	34.92	-16.62	A	89.04	0	39.29	10.53	80.41	A	-32.42	0	-2.11	-7.05	-29.54
	G	22565	38363	51600	71086	17.42	29.62	39.84	54.89	35.44	0.49	0.84	1.12	1.55	-3.14	-14.82	G	35.72	-28.21	0	-20.65	29.52	G	-30.97	2.15	0	-5.05	-28.03
	U	77666	97502	121836	204462	17.93	22.51	28.13	47.2	28.94	0.62	0.78	0.97	1.63	22.07	-10.29	U	71.04	-9.52	26.03	0	63.23	U	-27.3	7.58	5.32	0	-24.2
	T	53556	82398	124868	303603	14.02	21.57	32.68	79.47	36.94	0.38	0.58	0.88	2.15	-25.22	18.34	T	4.79	-44.57	-22.79	-38.73	0	T	-4.09	41.93	38.94	31.93	0
	mean concentration per fraction $\bar{c}_{j,k}$										0.51	0.71	0.96	1.82				C	A	G	U	T		C	A	G	U	T
3	C	15404	53622	63211	116411	5.64	19.65	23.16	42.66	22.78	0.25	0.86	1.02	1.87	-24.86	18.4	C	0	-39.49	-30.71	-35.2	-1.57	C	0	30.17	49.04	26.38	0.74
	A	69959	187931	179696	245780	10.26	27.57	26.36	36.05	25.06	0.41	1.1	1.05	1.44	24.16	-9.05	A	65.25	0	14.51	7.09	62.66	A	-23.18	0	14.49	-2.91	-22.61
	G	13322	45215	43666	46809	10.29	34.91	33.72	36.14	28.77	0.36	1.21	1.17	1.26	8.43	-20.56	G	44.31	-12.67	0	-6.48	42.05	G	-32.9	-12.66	0	-15.2	-32.41
	U	39694	111196	110506	153819	9.16	25.67	25.51	35.51	23.96	0.38	1.07	1.06	1.48	15.94	-6.32	U	54.31	-6.62	6.93	0	51.9	U	-20.87	3	17.93	0	-20.29
	T	27598	94565	112548	203790	7.22	24.75	29.46	53.34	28.69	0.25	0.86	1.03	1.86	-23.67	17.53	T	1.59	-38.52	-29.6	-34.17	0	T	-0.73	29.22	47.95	25.45	0
	mean concentration per fraction $\bar{c}_{j,k}$										0.33	1.02	1.07	1.58				C	A	G	U	T		C	A	G	U	T

***Supplementary Table 46**: Raw data Extended Data Fig. 5d – nucleobases in 10 % methanol, 170µm, 18h*

repeat k	species	measured values (µAU*s)				concentration (µM) $[A]_{j,k,HPLC}$ calibration acc. to Supplementary Table 3				c_0 (µM)	Normalization (Eq. 1) concentration (x c_0)				Ratio species vs mean (Eq. 4) $[A]_{j,k}/\bar{c}_{j,k}-1$ (%)		Ratio species against species (shown in heat maps), Eq. 2 $[A]_{j,k}/[B]_{j,k}-1$ (%)											
		top	II	III	bot	top	II	III	bot		top	II	III	bot	top	bot	top part (blue shade)						bot part (red shade)					
1	C	78158	85191	52270	84753	28.64	31.22	19.15	31.06	27.52	1.04	1.13	0.7	1.13	-1.72	0.17	C	0	-3.21	-2.58	-2.97	0.27	C	0	0.2	-0.13	1.58	-0.79
	A	158450	165766	99234	165978	23.24	24.32	14.56	24.35	21.62	1.08	1.12	0.67	1.13	1.53	-0.03	A	3.31	0	0.65	0.24	3.59	A	-0.2	0	-0.33	1.38	-0.99
	G	53827	56935	33830	56937	41.56	43.96	26.12	43.96	38.9	1.07	1.13	0.67	1.13	0.88	0.3	G	2.65	-0.64	0	-0.4	2.92	G	0.13	0.33	0	1.71	-0.66
	U	97095	102497	61898	100565	22.42	23.66	14.29	23.22	20.9	1.07	1.13	0.68	1.11	1.29	-1.39	U	3.06	-0.24	0.41	0	3.34	U	-1.56	-1.36	-1.69	0	-2.34
	T	86237	93612	57943	94509	22.57	24.5	15.17	24.74	21.75	1.04	1.13	0.7	1.14	-1.98	0.97	T	-0.26	-3.46	-2.84	-3.23	0	T	0.8	1	0.67	2.39	0
	mean concentration per fraction $\bar{c}_{j,k}$										1.06	1.13	0.68	1.13				C	A	G	U	T		C	A	G	U	T
2	C	77435	79594	81073	90067	28.37	29.16	29.71	33	30.06	0.94	0.97	0.99	1.1	-2.69	3.82	C	0	-4.65	-4.85	-3.23	-0.52	C	0	6.93	6.77	5.06	0.77
	A	157713	159110	156923	163580	23.13	23.34	23.02	23.99	23.37	0.99	1	0.98	1.03	2.05	-2.91	A	4.87	0	-0.22	1.48	4.33	A	-6.48	0	-0.15	-1.75	-5.76
	G	50435	50729	49930	52275	38.94	39.17	38.55	40.36	39.26	0.99	1	0.98	1.03	2.27	-2.76	G	5.1	0.22	0	1.7	4.56	G	-6.34	0.15	0	-1.6	-5.62
	U	95589	97173	96838	102402	22.07	22.43	22.36	23.64	22.62	0.98	0.99	0.99	1.04	0.56	-1.18	U	3.34	-1.46	-1.67	0	2.81	U	-4.82	1.78	1.63	0	-4.08
	T	86426	88920	89788	99238	22.62	23.28	23.5	25.98	23.84	0.95	0.98	0.99	1.09	-2.19	3.03	T	0.52	-4.15	-4.36	-2.73	0	T	-0.77	6.11	5.96	4.26	0
	mean concentration per fraction $\bar{c}_{j,k}$										0.97	0.99	0.99	1.06				C	A	G	U	T		C	A	G	U	T
3	C	69906	72633	74690	85031	25.61	26.61	27.37	31.16	27.69	0.93	0.96	0.99	1.13	-2.03	2.47	C	0	-3.91	-2.67	-2.87	-0.59	C	0	4.54	4.21	3.32	0.45
	A	143021	144255	147053	159901	20.98	21.16	21.57	23.46	21.79	0.96	0.97	0.99	1.08	1.95	-1.98	A	4.07	0	1.29	1.08	3.45	A	-4.35	0	-0.32	-1.17	-3.91
	G	47778	48833	50180	54277	36.89	37.71	38.75	41.91	38.81	0.95	0.97	1	1.08	0.66	-1.67	G	2.74	-1.27	0	-0.2	2.13	G	-4.04	0.32	0	-0.86	-3.61
	U	87054	88633	90374	99551	20.1	20.46	20.86	22.98	21.1	0.95	0.97	0.99	1.09	0.86	-0.82	U	2.95	-1.07	0.2	0	2.34	U	-3.21	1.19	0.87	0	-2.77
	T	78244	80635	83241	94181	20.48	21.11	21.79	24.65	22.01	0.93	0.96	0.99	1.12	-1.44	2.01	T	0.6	-3.33	-2.09	-2.29	0	T	-0.45	4.07	3.74	2.85	0
	mean concentration per fraction $\bar{c}_{j,k}$										0.94	0.97	0.99	1.1				C	A	G	U	T		C	A	G	U	T
4	C	69193	78890	73921	84430	25.35	28.91	27.09	30.94	28.07	0.9	1.03	0.96	1.1	-1.59	1.06	C	0	-1.85	-4.84	-0.58	-0.53	C	0	0.83	4.23	0.33	0.05
	A	137681	155601	141664	163529	20.2	22.82	20.78	23.99	21.95	0.92	1.04	0.95	1.09	0.26	0.23	A	1.88	0	-3.05	1.29	1.34	A	-0.83	0	3.36	-0.5	-0.78
	G	44412	49469	43806	49477	34.29	38.2	33.83	38.2	36.13	0.95	1.06	0.94	1.06	3.41	-3.04	G	5.09	3.15	0	4.48	4.53	G	-4.06	-3.25	0	-3.74	-4.01
	U	83668	95093	88454	101166	19.32	21.95	20.42	23.36	21.26	0.91	1.03	0.96	1.1	-1.02	0.73	U	0.59	-1.27	-4.28	0	0.05	U	-0.33	0.51	3.89	0	-0.28

	T	77238	87689	81616	93704	20.22	22.95	21.36	24.53	22.27	0.91	1.03	0.96	1.1	-1.07	1.02	T	0.53	-1.32	-4.33	-0.05	0	T	-0.05	0.79	4.18	0.28	0
	mean concentration per fraction $\bar{c}_{j,k}$										0.92	1.04	0.95	1.09				C	A	G	U	T		C	A	G	U	T
5	C	73499	77980	79620	101506	26.93	28.57	29.17	37.19	30.47	0.88	0.94	0.96	1.22	-3.1	4.42	C	0	-4.92	-5.93	-3.19	-1.22	C	0	7.61	9.74	3.98	1.41
	A	150313	157392	155633	183417	22.05	23.09	22.83	26.9	23.72	0.93	0.97	0.96	1.13	1.91	-2.97	A	5.17	0	-1.06	1.82	3.89	A	-7.07	0	1.97	-3.37	-5.76
	G	60032	62602	61853	71072	46.35	48.34	47.76	54.88	49.33	0.94	0.98	0.97	1.11	3	-4.85	G	6.3	1.07	0	2.91	5	G	-8.87	-1.94	0	-5.25	-7.59
	U	91144	95514	95440	117192	21.04	22.05	22.03	27.06	23.05	0.91	0.96	0.96	1.17	0.09	0.42	U	3.3	-1.78	-2.83	0	2.04	U	-3.83	3.49	5.54	0	-2.47
	T	81971	86748	87427	110267	21.46	22.71	22.88	28.86	23.98	0.89	0.95	0.95	1.2	-1.9	2.97	T	1.24	-3.74	-4.76	-1.99	0	T	-1.39	6.11	8.21	2.53	0
	mean concentration per fraction $\bar{c}_{j,k}$										0.91	0.96	0.96	1.17				C	A	G	U	T		C	A	G	U	T
6	C	61043	69797	80709	79576	22.37	25.57	29.57	29.16	26.67	0.84	0.96	1.11	1.09	-1.05	2.6	C	0	-1.73	-2.05	-0.63	-0.82	C	0	4.71	5.2	2.23	1.06
	A	122004	139951	160588	149274	17.9	20.53	23.56	21.9	20.97	0.85	0.98	1.12	1.04	0.69	-2.01	A	1.76	0	-0.33	1.11	0.92	A	-4.5	0	0.47	-2.37	-3.49
	G	38231	44079	49886	46405	29.52	34.04	38.52	35.83	34.48	0.86	0.99	1.12	1.04	1.01	-2.47	G	2.09	0.33	0	1.44	1.25	G	-4.94	-0.47	0	-2.83	-3.94
	U	74254	85319	98221	94090	17.14	19.7	22.68	21.72	20.31	0.84	0.97	1.12	1.07	-0.42	0.36	U	0.64	-1.1	-1.42	0	-0.19	U	-2.18	2.43	2.91	0	-1.14
	T	68337	77934	89529	87427	17.89	20.4	23.43	22.88	21.15	0.85	0.96	1.11	1.08	-0.23	1.53	T	0.83	-0.91	-1.23	0.19	0	T	-1.05	3.61	4.1	1.16	0
	mean concentration per fraction $\bar{c}_{j,k}$										0.85	0.97	1.11	1.07				C	A	G	U	T		C	A	G	U	T

Supplementary Table 47*: Raw data Extended Data Fig. 5e – nucleobases in water, 127 µm, 18h*

rep eat k	spe cies	measured values (µAU*min)				concentration (µM) $[A]_{j,k,HPLC}$ calibration acc. to Supplementary Table 3				c_0 (µM)	Normalization (Eq. 1) concentration (x c_0)				Ratio species vs mean (Eq. 4) $[A]_{j,k}/\bar{c}_{j,k}-1$ (%)		Ratio species against species (shown in heat maps), Eq. 2 $[A]_{j,k}/[B]_{j,k}-1$ (%)											
		top	II	III	bot	top	II	III	bot		top	II	III	bot	top	bot	top part (blue shade)						bot part (red shade)					
	C	1212.92	1308.97	1890.54	2132.85	26.67	28.78	41.56	46.89	35.97	0.74	0.8	1.16	1.3	-11.76	8.89	C	0	-18.55	-21.06	-14.65	-0.04	C	0	16.33	17.78	12.84	0.33
	A	5811.7	5625	6953.27	7155.56	51.15	49.51	61.2	62.98	56.21	0.91	0.88	1.09	1.12	8.33	-6.39	A	22.77	0	-3.09	4.78	22.71	A	-14.04	0	1.25	-3	-13.76
1	G	1003.53	961.55	1127.11	1182.7	46.49	44.55	52.22	54.79	49.51	0.94	0.9	1.05	1.11	11.78	-7.55	G	26.68	3.19	0	8.11	26.62	G	-15.1	-1.23	0	-4.2	-14.82
	U	1742.74	1731.05	2234.5	2317.84	24.14	23.98	30.95	32.11	27.79	0.87	0.86	1.11	1.16	3.39	-3.49	U	17.17	-4.56	-7.5	0	17.12	U	-11.38	3.1	4.38	0	-11.09
	T	1445.54	1571.36	2247.75	2532.51	22.7	24.68	35.3	39.77	30.61	0.74	0.81	1.15	1.3	-11.73	8.54	T	0.04	-18.51	-21.03	-14.62	0	T	-0.33	15.95	17.4	12.47	0
	mean concentration per fraction $\bar{c}_{j,k}$										0.84	0.85	1.11	1.2				C	A	G	U	T		C	A	G	U	T
	C	1159.97	1488.63	1774.75	2341.46	25.5	32.73	39.02	51.48	37.18	0.69	0.88	1.05	1.38	-13.48	10.88	C	0	-19.74	-24.86	-15.85	-1.38	C	0	19.69	24.26	14.16	0.7
	A	5558.72	6240.86	6695.57	7524.83	48.92	54.93	58.93	66.23	57.25	0.85	0.96	1.03	1.16	7.79	-7.36	A	24.59	0	-6.38	4.84	22.87	A	-16.45	0	3.82	-4.62	-15.86
2	G	903.78	943.6	1009.92	1103.23	41.87	43.72	46.79	51.11	45.87	0.91	0.95	1.02	1.11	15.14	-10.76	G	33.08	6.82	0	11.99	31.25	G	-19.52	-3.68	0	-8.13	-18.96
	U	1685.02	1942.51	2134.34	2507.16	23.34	26.91	29.56	34.73	28.63	0.82	0.94	1.03	1.21	2.82	-2.87	U	18.84	-4.61	-10.7	0	17.2	U	-12.4	4.84	8.85	0	-11.79
	T	1402.12	1775.26	2115.04	2771.75	22.02	27.88	33.22	43.53	31.66	0.7	0.88	1.05	1.37	-12.27	10.11	T	1.4	-18.61	-23.81	-14.68	0	T	-0.7	18.85	23.39	13.36	0
	mean concentration per fraction $\bar{c}_{j,k}$										0.79	0.92	1.04	1.25				C	A	G	U	T		C	A	G	U	T
	C	1081.12	1433.7	1719.97	2077.44	23.77	31.52	37.81	45.67	34.69	0.69	0.91	1.09	1.32	-12.33	7.91	C	0	-21.64	-15.89	-16.99	-3.23	C	0	16.89	10.64	11.86	2.02
	A	5441.03	5936.8	6506.52	7008.68	47.89	52.25	57.26	61.68	54.77	0.87	0.95	1.05	1.13	11.88	-7.68	A	27.62	0	7.34	5.94	23.5	A	-14.45	0	-5.35	-4.3	-12.73
3	G	1293.6	1501	1668.19	1889.65	59.93	69.54	77.29	87.55	73.58	0.81	0.95	1.05	1.19	4.24	-2.47	G	18.9	-6.83	0	-1.3	15.06	G	-9.62	5.65	0	1.11	-7.79
	U	1635.63	1856.95	2102.43	2332.26	22.66	25.72	29.12	32.31	27.45	0.83	0.94	1.06	1.18	5.61	-3.54	U	20.47	-5.6	1.32	0	16.58	U	-10.61	4.49	-1.1	0	-8.8
	T	1349.53	1747.03	2068.44	2459.84	21.19	27.44	32.49	38.63	29.94	0.71	0.92	1.09	1.29	-9.4	5.78	T	3.34	-19.03	-13.09	-14.22	0	T	-1.98	14.58	8.45	9.65	0
	mean concentration per fraction $\bar{c}_{j,k}$										0.78	0.93	1.07	1.22				C	A	G	U	T		C	A	G	U	T

***Supplementary Table 48**: Raw data Extended Data Fig. 5f – nucleobases in water, 150 µm, 18h*

repeat k	species	measured values (µAU*min)				concentration (µM) $[A]_{j,k,HPLC}$ calibration acc. to Supplementary Table 3				c_0 (µM)	Normalization (Eq. 1) concentration (x c_0)				Ratio species vs mean (Eq. 4) $[A]_{j,k}/\bar{c}_{j,k} - 1$ (%)		Ratio species against species (shown in heat maps), Eq. 2 $[A]_{j,k}/[B]_{j,k} - 1$ (%)											
		top	II	III	bot	top	II	III	bot		top	II	III	bot	top	bot	top part (blue shade)						bot part (red shade)					
1	C	859.86	1159.13	1832.82	2756.22	18.9	25.48	40.29	60.6	36.32	0.52	0.7	1.11	1.67	-19.85	14.63	C	0	-29.6	-32.66	-23.21	-2.95	C	0	30.1	28	21.96	0.79
	A	4489.45	5223.05	6789.36	7787.47	39.51	45.97	59.75	68.54	53.44	0.74	0.86	1.12	1.28	13.85	-11.89	A	42.04	0	-4.35	9.08	37.85	A	-23.13	0	-1.61	-6.25	-22.52
	G	760.04	793.91	1097.56	1281.62	35.21	36.78	50.85	59.38	45.55	0.77	0.81	1.12	1.3	19.03	-10.45	G	48.51	4.55	0	14.04	44.12	G	-21.88	1.63	0	-4.72	-21.26
	U	1315.88	1605.43	2188.41	2655.78	18.23	22.24	30.31	36.79	26.89	0.68	0.83	1.13	1.37	4.38	-6.01	U	30.22	-8.32	-12.31	0	26.38	U	-18.01	6.67	4.95	0	-17.36
	T	1045.6	1371.79	2153.59	3227.01	16.42	21.54	33.82	50.68	30.62	0.54	0.7	1.1	1.66	-17.41	13.73	T	3.04	-27.46	-30.61	-20.87	0	T	-0.79	29.07	27	21	0
	mean concentration per fraction $\bar{c}_{j,k}$										0.65	0.78	1.12	1.46				C	A	G	U	T		C	A	G	U	T
2	C	876.27	1631.06	1701.66	2485.39	19.26	35.86	37.41	54.64	36.79	0.52	0.97	1.02	1.49	-21.95	10.88	C	0	-33.68	-34.13	-26.92	-1.21	C	0	21	20.37	15.06	1.72
	A	4971.9	6558.56	5932.42	7729.57	43.76	57.72	52.21	68.03	55.43	0.79	1.04	0.94	1.23	17.67	-8.37	A	50.77	0	-0.68	10.18	48.94	A	-17.36	0	-0.53	-4.91	-15.94
	G	798.78	1044.11	937.02	1239.9	37.01	48.37	43.41	57.44	46.56	0.79	1.04	0.93	1.23	18.48	-7.88	G	51.81	0.69	0	10.93	49.96	G	-16.92	0.53	0	-4.41	-15.5
	U	1427.28	2041.44	1928.25	2571.13	19.77	28.28	26.71	35.61	27.59	0.72	1.02	0.97	1.29	6.8	-3.63	U	36.84	-9.24	-9.86	0	35.18	U	-13.09	5.17	4.61	0	-11.6
	T	1044.13	1941.59	2017.97	2876.19	16.4	30.49	31.69	45.17	30.94	0.53	0.99	1.02	1.46	-21	9.01	T	1.23	-32.86	-33.32	-26.03	0	T	-1.69	18.96	18.34	13.12	0
	mean concentration per fraction $\bar{c}_{j,k}$										0.67	1.01	0.98	1.34				C	A	G	U	T		C	A	G	U	T
3	C	829.95	1023.2	1581.15	3367.89	18.25	22.5	34.76	74.04	37.39	0.49	0.6	0.93	1.98	-24.26	17.36	C	0	-36.97	-36.6	-30.5	0.08	C	0	38.8	32.97	27.56	-0.38
	A	4895.84	4862.88	6512.05	9022.54	43.09	42.8	57.31	79.41	55.65	0.77	0.77	1.03	1.43	20.15	-15.44	A	58.64	0	0.57	10.25	58.77	A	-27.95	0	-4.2	-8.1	-28.23
	G	781.25	746.22	1020.3	1511.52	36.19	34.57	47.27	70.03	47.02	0.77	0.74	1.01	1.49	19.46	-11.73	G	57.74	-0.57	0	9.62	57.87	G	-24.79	4.39	0	-4.07	-25.08
	U	1417.25	1471.14	2050.95	3133.35	19.63	20.38	28.41	43.4	27.96	0.7	0.73	1.02	1.55	8.98	-7.99	U	43.89	-9.3	-8.78	0	44.01	U	-21.61	8.81	4.24	0	-21.9
	T	985.2	1213.99	1865.71	4016.37	15.47	19.07	29.3	63.08	31.73	0.49	0.6	0.92	1.99	-24.33	17.81	T	-0.08	-37.02	-36.66	-30.56	0	T	0.38	39.33	33.47	28.05	0
	mean concentration per fraction $\bar{c}_{j,k}$										0.64	0.69	0.98	1.69				C	A	G	U	T		C	A	G	U	T

***Supplementary Table 49**: Raw data Extended Data Fig. 5g – nucleobases in water, 200 µm, 18h*

repeat k	species	measured values (µAU*s) top	II	III	bot	concentration (µM) $[A]_{j,k,HPLC}$ calibration acc. to Supplementary Table 3 top	II	III	bot	c_0 (µM)	Normalization (Eq. 1) concentration (x c_0) top	II	III	bot	Ratio species vs mean (Eq. 4) $[A]_{j,k}/\bar{c}_{j,k}-1$ (%) top	bot
1	C	29851	75725	140710	169921	10.94	27.75	51.56	62.26	38.13	0.29	0.73	1.35	1.63	-41.11	15.4
	A	267376	401379	465079	514578	39.22	58.88	68.22	75.48	60.45	0.65	0.97	1.13	1.25	33.18	-11.76
	G	52943	78178	85801	93774	40.88	60.37	66.25	72.41	59.98	0.68	1.01	1.1	1.21	39.91	-14.69
	U	67876	118021	152663	174324	15.67	27.25	35.24	40.24	29.6	0.53	0.92	1.19	1.36	8.66	-3.93
	T	35609	90747	165867	200390	9.32	23.75	43.42	52.45	32.24	0.29	0.74	1.35	1.63	-40.65	14.98
	mean concentration per fraction $\bar{c}_{j,k}$										0.49	0.87	1.22	1.42		
2	C	49227	104983	131775	151478	18.04	38.47	48.28	55.5	40.07	0.45	0.96	1.2	1.39	-20.81	8.29
	A	270630	433283	472666	505007	39.7	63.56	69.33	74.08	61.67	0.64	1.03	1.12	1.2	13.26	-6.08
	G	58792	85466	89999	98099	45.4	65.99	69.49	75.75	64.16	0.71	1.03	1.08	1.18	24.49	-7.69
	U	75938	132344	152537	166781	17.53	30.55	35.21	38.5	30.45	0.58	1	1.16	1.26	1.29	-1.14
	T	60442	127301	155157	177320	15.82	33.32	40.61	46.41	34.04	0.46	0.98	1.19	1.36	-18.23	6.6
	mean concentration per fraction $\bar{c}_{j,k}$										0.57	1	1.15	1.28		
3	C	34563	61232	112870	181906	12.66	22.44	41.36	66.65	35.78	0.35	0.63	1.16	1.86	-36.78	27.57
	A	308651	398003	475136	455906	45.27	58.38	69.7	66.87	60.06	0.75	0.97	1.16	1.11	34.64	-23.75
	G	52211	69672	84736	85988	40.32	53.8	65.43	66.4	56.48	0.71	0.95	1.16	1.18	27.47	-19.51
	U	78052	109834	150754	166861	18.02	25.36	34.8	38.52	29.18	0.62	0.87	1.19	1.32	10.3	-9.59
	T	42282	76418	135915	214615	11.07	20	35.58	56.18	30.71	0.36	0.65	1.16	1.83	-35.63	25.28
	mean concentration per fraction $\bar{c}_{j,k}$										0.56	0.81	1.17	1.46		

Ratio species against species (shown in heat maps), Eq. 2: $[A]_{j,k}/[B]_{j,k}-1$ (%)

repeat k	species	top part (blue shade)						bot part (red shade)					
1	C	C	0	-55.78	-57.91	-45.81	-0.78	C	0	30.78	35.27	20.12	0.36
	A	A	126.16	0	-4.81	22.56	124.39	A	-23.54	0	3.43	-8.16	-23.26
	G	G	137.59	5.05	0	28.76	135.73	G	-26.07	-3.31	0	-11.2	-25.8
	U	U	84.52	-18.41	-22.34	0	83.08	U	-16.75	8.88	12.61	0	-16.45
	T	T	0.79	-55.43	-57.58	-45.38	0	T	-0.36	30.31	34.78	19.68	0
			C	A	G	U	T		C	A	G	U	T
2	C	C	0	-30.08	-36.39	-21.82	-3.15	C	0	15.3	17.31	9.54	1.59
	A	A	43.02	0	-9.02	11.82	38.52	A	-13.27	0	1.75	-5	-11.89
	G	G	57.2	9.92	0	22.9	52.25	G	-14.76	-1.72	0	-6.63	-13.41
	U	U	27.91	-10.57	-18.63	0	23.88	U	-8.71	5.26	7.1	0	-7.26
	T	T	3.25	-27.81	-34.32	-19.28	0	T	-1.56	13.5	15.48	7.83	0
			C	A	G	U	T		C	A	G	U	T
3	C	C	0	-53.05	-50.41	-42.69	-1.79	C	0	67.3	58.49	41.1	1.83
	A	A	112.97	0	5.62	22.06	109.15	A	-40.23	0	-5.27	-15.66	-39.13
	G	G	101.63	-5.32	0	15.56	98.02	G	-36.9	5.56	0	-10.97	-35.75
	U	U	74.48	-18.07	-13.47	0	71.35	U	-29.13	18.57	12.33	0	-27.83
	T	T	1.83	-52.19	-49.5	-41.64	0	T	-1.8	64.3	55.64	38.56	0
			C	A	G	U	T		C	A	G	U	T

***Supplementary Table 50**: Raw data Extended Data Fig. 5h – nucleobases in water, 170 µm, 18h, pH 3*
Due to technical problems, for the third replicate the temperature gradient was not applied for the entire duration of the experiment, which is why we do not take it into account for the calculation of the heat flow-driven enrichment.

repeat k	species	measured values (µAU*min)				concentration (µM) $[A]_{j,k,HPLC}$ calibration acc. to Supplementary Table 3				c_0 (µM)	Normalization (Eq. 1) concentration (x c_0)				Ratio species vs mean (Eq. 4) $[A]_{j,k}/\bar{c}_{j,k}-1$ (%)		Ratio species against species (shown in heat maps), Eq. 2 $[A]_{j,k}/[B]_{j,k}-1$ (%)											
		top	II	III	bot	top	II	III	bot		top	II	III	bot	top	bot	top part (blue shade)						bot part (red shade)					
1	C	846.79	1460.52	1607.47	1397.6	18.62	32.11	35.34	30.73	29.2	0.64	1.1	1.21	1.05	2.23	-15.42	C	0	-6.52	-14.19	2.29	47.43	C	0	-2.5	1.08	-23.25	-37.26
	A	1951.56	3032.08	3373.53	3088.2	17.18	26.69	29.69	27.18	25.18	0.68	1.06	1.18	1.08	9.36	-13.26	A	6.97	0	-8.21	9.42	57.71	A	2.56	0	3.67	-21.29	-35.65
	G	581.85	823.23	912.01	815.28	26.96	38.14	42.25	37.77	36.28	0.74	1.05	1.16	1.04	19.13	-16.33	G	16.53	8.94	0	19.2	71.81	G	-1.07	-3.54	0	-24.07	-37.93
	U	1317.27	1958.65	2279.63	2897.69	18.25	27.13	31.58	40.14	29.27	0.62	0.93	1.08	1.37	-0.06	10.2	U	-2.24	-8.61	-16.11	0	44.13	U	30.3	27.04	31.7	0	-18.25
	T	814.79	1594.84	1966.43	3160.18	12.8	25.05	30.88	49.63	29.59	0.43	0.85	1.04	1.68	-30.66	34.81	T	-32.17	-36.59	-41.8	-30.62	0	T	59.39	55.41	61.11	22.33	0
	mean concentration per fraction $\bar{c}_{j,k}$										0.62	1	1.14	1.24				C	A	G	U	T		C	A	G	U	T
2	C	850.39	1203.32	1433.84	1605.78	18.7	26.46	31.52	35.3	27.99	0.67	0.95	1.13	1.26	-1.37	-12.35	C	0	-8.86	-19.17	-3.74	43.62	C	0	-4.1	6.02	-15.08	-35.11
	A	2200.16	2711.59	3149.67	3948.02	19.36	23.87	27.72	34.75	26.42	0.73	0.9	1.05	1.31	8.22	-8.61	A	9.73	0	-11.3	5.62	57.59	A	4.27	0	10.55	-11.45	-32.34
	G	794.03	890.45	1016.65	1143.17	36.79	41.25	47.1	52.96	44.53	0.83	0.93	1.06	1.19	22.01	-17.33	G	23.71	12.74	0	19.08	77.67	G	-5.68	-9.54	0	-19.9	-38.8
	U	1354.97	1662.51	1894.14	2900.26	18.77	23.03	26.24	40.17	27.05	0.69	0.85	0.97	1.49	2.46	3.21	U	3.89	-5.32	-16.02	0	49.2	U	17.76	12.93	24.85	0	-23.59
	T	771.97	1117.49	1524.61	3226.26	12.12	17.55	23.94	50.67	26.07	0.47	0.67	0.92	1.94	-31.33	35.07	T	-30.37	-36.54	-43.72	-32.97	0	T	54.11	47.79	63.39	30.87	0
	mean concentration per fraction $\bar{c}_{j,k}$										0.68	0.86	1.02	1.44				C	A	G	U	T		C	A	G	U	T

***Supplementary Table 51**: Raw data Extended Data Fig. 5i – nucleobases in water, 170 µm, 18h, pH 4*
Due to technical problems, for the third replicate the temperature gradient was not applied for the entire duration of the experiment, which is why we do not take it into account for the calculation of the heat flow-driven enrichment.

repeat k	species	measured values (µAU*s)				concentration (µM) $[A]_{j,k,HPLC}$ calibration acc. to Supplementary Table 3				c_0 (µM)	Normalization (Eq. 1) concentration (x c_0)				Ratio species vs mean (Eq. 4) $[A]_{j,k}/\bar{c}_{j,k} - 1$ (%)		Ratio species against species (shown in heat maps), Eq. 2 $[A]_{j,k}/[B]_{j,k} - 1$ (%)											
		top	II	III	bot	top	II	III	bot		top	II	III	bot	top	bot	top part (blue shade)						bot part (red shade)					
1	C	55180	43628	68083	112994	20.22	15.99	24.95	41.4	25.64	0.79	0.62	0.97	1.61	5.97	11.42	C	0	1.58	-16.31	12.22	54.36	C	0	16.54	50.66	14.99	-8.76
	A	120551	128740	156657	215154	17.68	18.88	22.98	31.56	22.78	0.78	0.83	1.01	1.39	4.33	-4.39	A	-1.55	0	-17.61	10.48	51.96	A	-14.2	0	29.27	-1.33	-21.71
	G	48984	50612	52616	55720	37.82	39.08	40.63	43.02	40.14	0.94	0.97	1.01	1.07	26.62	-26.04	G	19.49	21.37	0	34.09	84.44	G	-33.62	-22.64	0	-23.67	-39.44
	U	68861	81036	104456	137613	15.9	18.71	24.11	31.77	22.62	0.7	0.83	1.07	1.4	-5.57	-3.1	U	-10.89	-9.49	-25.43	0	37.55	U	-13.04	1.35	31.02	0	-20.65
	T	46050	62152	92815	159526	12.05	16.27	24.29	41.76	23.59	0.51	0.69	1.03	1.77	-31.35	22.12	T	-35.22	-34.19	-45.78	-27.3	0	T	9.6	27.73	65.11	26.03	0
	mean concentration per fraction $\bar{c}_{j,k}$										0.74	0.79	1.02	1.45				C	A	G	U	T		C	A	G	U	T
2	C	58200	68579	75452	81354	21.33	25.13	27.65	29.81	25.98	0.82	0.97	1.06	1.15	-4.81	4.51	C	0	-7.8	-11.26	-6.66	3.1	C	0	6.78	11.24	5.65	-0.2
	A	147918	164129	173943	178542	21.7	24.08	25.51	26.19	24.37	0.89	0.99	1.05	1.07	3.24	-2.12	A	8.46	0	-3.75	1.23	11.82	A	-6.35	0	4.18	-1.06	-6.53
	G	38445	41587	43334	42869	29.69	32.11	33.46	33.1	32.09	0.93	1	1.04	1.03	7.26	-6.05	G	12.69	3.9	0	5.18	16.18	G	-10.11	-4.02	0	-5.03	-10.29
	U	94095	105021	112612	116204	21.72	24.25	26	26.83	24.7	0.88	0.98	1.05	1.09	1.98	-1.07	U	7.14	-1.22	-4.92	0	10.46	U	-5.34	1.07	5.3	0	-5.53
	T	79154	97074	107101	114298	20.72	25.41	28.03	29.92	26.02	0.8	0.98	1.08	1.15	-7.67	4.72	T	-3	-10.57	-13.92	-9.47	0	T	0.2	6.99	11.47	5.86	0
	mean concentration per fraction $\bar{c}_{j,k}$										0.86	0.98	1.06	1.1				C	A	G	U	T		C	A	G	U	T

***Supplementary Table 52**: Raw data Extended Data Fig. 5j – nucleobases in water, 170 µm, 18h, pH 5*

repeat k	species	measured values (µAU*min)				concentration (µM) $[A]_{j,k,HPLC}$ calibration acc. to Supplementary Table 3				c_0 (µM)	Normalization (Eq. 1) concentration (x c_0)				Ratio species vs mean (Eq. 4) $[A]_{j,k}/\bar{c}_{j,k} - 1$ (%)		Ratio species against species (shown in heat maps), Eq. 2 $[A]_{j,k}/[B]_{j,k} - 1$ (%)											
		top	II	III	bot	top	II	III	bot		top	II	III	bot	top	bot	top part (blue shade)						bot part (red shade)					
1	C	587.34	912.57	1270.36	3093.81	12.91	20.06	27.93	68.02	32.23	0.4	0.62	0.87	2.11	-29.27	21.19	C	0	-43.43	-42.7	-34.89	-1.98	C	0	47.78	43.5	31.74	0.69
	A	2402.21	3027.1	3295.29	4844.31	21.14	26.64	29	42.64	29.86	0.71	0.89	0.97	1.43	25.02	-17.99	A	76.76	0	1.28	15.08	73.26	A	-32.33	0	-2.89	-10.85	-31.86
	G	380.08	475.55	519.27	799.37	17.61	22.03	24.06	37.03	25.18	0.7	0.87	0.96	1.47	23.45	-15.55	G	74.53	-1.26	0	13.64	71.08	G	-30.32	2.98	0	-8.2	-29.83
	U	1401.87	1875.82	2185.73	3649.52	19.42	25.98	30.28	50.55	31.56	0.62	0.82	0.96	1.6	8.64	-8.01	U	53.59	-13.11	-12	0	50.55	U	-24.09	12.17	8.93	0	-23.57
	T	849.17	1308.32	1798.81	4354.33	13.34	20.55	28.25	68.39	32.63	0.41	0.63	0.87	2.1	-27.84	20.35	T	2.02	-42.28	-41.55	-33.58	0	T	-0.69	46.76	42.51	30.83	0
	mean concentration per fraction $\bar{c}_{j,k}$										0.57	0.77	0.92	1.74				C	A	G	U	T		C	A	G	U	T
2	C	366.76	915.19	1375.27	2338.75	8.06	20.12	30.24	51.42	27.46	0.29	0.73	1.1	1.87	-23.08	16.22	C	0	-35.16	-36.53	-28.67	1.95	C	0	33.93	34.75	23.88	-0.61
	A	1427.36	3064.26	3709.26	4406.89	12.56	26.97	32.65	38.79	27.74	0.45	0.97	1.18	1.4	18.62	-13.22	A	54.22	0	-2.12	10	57.22	A	-25.33	0	0.62	-7.5	-25.79
	G	408.3	864.84	1030.59	1226.34	18.92	40.07	47.75	56.82	40.89	0.46	0.98	1.17	1.39	21.19	-13.75	G	57.56	2.17	0	12.39	60.63	G	-25.79	-0.61	0	-8.07	-26.24
	U	858.37	1893.45	2436.83	3151.73	11.89	26.23	33.75	43.66	28.88	0.41	0.91	1.17	1.51	7.83	-6.18	U	40.19	-9.09	-11.02	0	42.93	U	-19.28	8.11	8.78	0	-19.77
	T	508.67	1294.28	1933.9	3327.34	7.99	20.33	30.37	52.26	27.74	0.29	0.73	1.1	1.88	-24.55	16.94	T	-1.91	-36.4	-37.75	-30.03	0	T	0.62	34.75	35.58	24.64	0
	mean concentration per fraction $\bar{c}_{j,k}$										0.38	0.87	1.14	1.61				C	A	G	U	T		C	A	G	U	T
3	C	362.27	1068.77	1630.54	2183.51	7.96	23.5	35.85	48	28.83	0.28	0.82	1.24	1.67	-39.74	16.33	C	0	-55.7	-53.82	-46.09	-1.98	C	0	35.46	31.38	23.17	1.34
	A	2052.56	3219.03	3847.39	4045.81	18.06	28.33	33.86	35.61	28.97	0.62	0.98	1.17	1.23	36.02	-14.12	A	125.73	0	4.25	21.69	121.26	A	-26.18	0	-3.01	-9.07	-25.19
	G	310.91	511.87	597.37	658.71	14.4	23.71	27.68	30.52	24.08	0.6	0.98	1.15	1.27	30.47	-11.45	G	116.53	-4.08	0	16.73	112.24	G	-23.88	3.11	0	-6.25	-22.87
	U	1109.56	2028.86	2594.78	2927.05	15.37	28.1	35.94	40.54	29.99	0.51	0.94	1.2	1.35	11.77	-5.55	U	85.5	-17.83	-14.33	0	81.82	U	-18.81	9.98	6.67	0	-17.73
	T	525.62	1536.89	2332.33	3064.29	8.25	24.14	36.63	48.13	29.29	0.28	0.82	1.25	1.64	-38.52	14.8	T	2.02	-54.8	-52.88	-45	0	T	-1.32	33.67	29.65	21.55	0
	mean concentration per fraction $\bar{c}_{j,k}$										0.46	0.91	1.2	1.43				C	A	G	U	T		C	A	G	U	T

***Supplementary Table 53**: Raw data Extended Data Fig. 5k – nucleobases in water, 170 µm, 18h, pH 7*
Due to technical problems, for the third replicate the temperature gradient was not applied for the entire duration of the experiment, which is why we do not take it into account for the calculation of the heat flow-driven enrichment.

repeat k	species	measured values (µAU*s)				concentration (µM) $[A]_{j,k,HPLC}$ calibration acc. to Supplementary Table 3				c_0 (µM)	Normalization (Eq. 1) concentration (x c_0)				Ratio species vs mean (Eq. 4) $[A]_{j,k}/\bar{c}_{j,k} - 1$ (%)		Ratio species against species (shown in heat maps), Eq. 2 $[A]_{j,k}/[B]_{j,k} - 1$ (%)											
		top	II	III	bot	top	II	III	bot		top	II	III	bot	top	bot	top part (blue shade)						bot part (red shade)					
1	C	38356	61527	91932	102887	14.05	22.54	33.69	37.7	27	0.52	0.84	1.25	1.4	-23.71	8.61	C	0	-32.25	-42.11	-24.03	-3.31	C	0	14.59	19.94	9.81	1.39
	A	128238	162307	173584	203371	18.81	23.81	25.46	29.83	24.48	0.77	0.97	1.04	1.22	12.61	-5.21	A	47.61	0	-14.55	12.14	42.72	A	-12.73	0	4.67	-4.17	-11.52
	G	35672	49091	27718	46187	27.54	37.91	21.4	35.66	30.63	0.9	1.24	0.7	1.16	31.78	-9.44	G	72.74	17.02	0	31.23	67.02	G	-16.62	-4.46	0	-8.45	-15.47
	U	75869	100297	125881	140802	17.52	23.15	29.06	32.51	25.56	0.69	0.91	1.14	1.27	0.42	-1.09	U	31.63	-10.82	-23.8	0	27.27	U	-8.93	4.36	9.23	0	-7.67
	T	56704	89706	129779	145055	14.84	23.48	33.97	37.97	27.57	0.54	0.85	1.23	1.38	-21.1	7.13	T	3.43	-29.93	-40.13	-21.43	0	T	-1.37	13.02	18.3	8.3	0
	mean concentration per fraction $\bar{c}_{j,k}$										0.68	0.96	1.07	1.29				C	A	G	U	T		C	A	G	U	T
2	C	39219	60681	79652	100721	14.37	22.23	29.19	36.91	25.67	0.56	0.87	1.14	1.44	-16.85	10.95	C	0	-30.71	-19.02	-22.75	-3.91	C	0	25.36	19.8	14.65	-0.18
	A	130685	159701	171198	185488	19.17	23.43	25.11	27.21	23.73	0.81	0.99	1.06	1.15	20.01	-11.5	A	44.33	0	16.88	11.49	38.68	A	-20.23	0	-4.44	-8.54	-20.38
	G	32468	45106	53962	56366	25.07	34.83	41.67	43.52	36.27	0.69	0.96	1.15	1.2	2.67	-7.38	G	23.48	-14.44	0	-4.61	18.65	G	-16.53	4.65	0	-4.29	-16.68
	U	76278	99060	113760	131982	17.61	22.87	26.26	30.47	24.3	0.72	0.94	1.08	1.25	7.64	-3.23	U	29.46	-10.31	4.84	0	24.39	U	-12.78	9.34	4.49	0	-12.94
	T	57560	85949	109445	142298	15.07	22.5	28.65	37.25	25.86	0.58	0.87	1.11	1.44	-13.47	11.15	T	4.07	-27.89	-15.72	-19.61	0	T	0.18	25.59	20.02	14.86	0
	mean concentration per fraction $\bar{c}_{j,k}$										0.67	0.92	1.11	1.3				C	A	G	U	T		C	A	G	U	T

Supplementary Table 54*: Raw data Extended Data Fig. 5l – nucleobases in water, 170 µm, 18h, pH 10*

repeat k	species	measured values (µAU*s)				concentration (µM) $[A]_{j,k,HPLC}$ calibration acc. to Supplementary Table 3				c_0 (µM)	Normalization (Eq. 1) concentration (x c_0)				Ratio species vs mean (Eq. 4) $[A]_{j,k}/\bar{c}_{j,k} - 1$ (%)		Ratio species against species (shown in heat maps), Eq. 2 $[A]_{j,k}/[B]_{j,k} - 1$ (%)											
		top	II	III	bot	top	II	III	bot		top	II	III	bot	top	bot	top part (blue shade)						bot part (red shade)					
1	C	29587	50455	70242	118956	10.84	18.49	25.74	43.59	24.66	0.44	0.75	1.04	1.77	-10.37	-5.14	C	0	-32.94	-19.98	-3.69	25.15	C	0	18.11	-0.31	-13.28	-21.12
	A	104484	137461	157117	238505	15.33	20.16	23.05	34.99	23.38	0.66	0.86	0.99	1.5	33.67	-19.68	A	49.13	0	19.33	43.63	86.64	A	-15.33	0	-15.6	-26.57	-33.21
	G	34489	47055	58281	111305	26.63	36.33	45	85.95	48.48	0.55	0.75	0.93	1.77	12.02	-4.84	G	24.97	-16.2	0	20.36	56.41	G	0.32	18.48	0	-13	-20.87
	U	47694	69292	88061	212958	11.01	16	20.33	49.16	24.13	0.46	0.66	0.84	2.04	-6.94	9.39	U	3.83	-30.38	-16.92	0	29.94	U	15.31	36.19	14.95	0	-9.04
	T	33568	56593	78001	214131	8.79	14.81	20.42	56.05	25.02	0.35	0.59	0.82	2.24	-28.38	20.26	T	-20.1	-46.42	-36.06	-23.04	0	T	26.78	49.73	26.38	9.94	0
	mean concentration per fraction $\bar{c}_{j,k}$										0.49	0.72	0.92	1.86				C	A	G	U	T		C	A	G	U	T
2	C	28394	42891	64863	145029	10.4	15.72	23.77	53.14	25.76	0.4	0.61	0.92	2.06	-8.59	-6.98	C	0	-33.94	-14.29	-1.61	29.35	C	0	18.32	-6.52	-15.28	-21.86
	A	100903	123827	147618	287744	14.8	18.16	21.65	42.21	24.21	0.61	0.75	0.89	1.74	38.37	-21.38	A	51.37	0	29.75	48.93	95.81	A	-15.49	0	-21	-28.4	-33.96
	G	31542	39595	48863	147727	24.36	30.57	37.73	114.07	51.68	0.47	0.59	0.73	2.21	6.65	-0.48	G	16.67	-22.93	0	14.79	50.91	G	6.98	26.58	0	-9.36	-16.41
	U	44987	57351	69134	266836	10.39	13.24	15.96	61.6	25.3	0.41	0.52	0.63	2.44	-7.09	9.8	U	1.64	-32.86	-12.88	0	31.47	U	18.03	39.66	10.33	0	-7.77
	T	31334	45612	59488	264935	8.2	11.94	15.57	69.35	26.27	0.31	0.45	0.59	2.64	-29.33	19.05	T	-22.69	-48.93	-33.74	-23.94	0	T	27.97	51.42	19.63	8.43	0
	mean concentration per fraction $\bar{c}_{j,k}$										0.44	0.59	0.75	2.22				C	A	G	U	T		C	A	G	U	T
3	C	28808	45790	62642	113481	10.56	16.78	22.95	41.58	22.97	0.46	0.73	1	1.81	-10.9	-1.78	C	0	-29.99	-21.44	-5.05	16.68	C	0	21.3	5.33	-9.1	-17.81
	A	100158	129649	152746	227717	14.69	19.02	22.41	33.4	22.38	0.66	0.85	1	1.49	27.27	-19.03	A	42.84	0	12.21	35.62	66.67	A	-17.56	0	-13.16	-25.06	-32.24
	G	34649	45533	54923	101799	26.75	35.16	42.41	78.6	45.73	0.59	0.77	0.93	1.72	13.42	-6.75	G	27.29	-10.88	0	20.86	48.53	G	-5.06	15.16	0	-13.7	-21.97
	U	46407	64984	81145	190956	10.71	15	18.73	44.08	22.13	0.48	0.68	0.85	1.99	-6.16	8.05	U	5.32	-26.27	-17.26	0	22.89	U	10.01	33.45	15.88	0	-9.58
	T	33459	51081	68119	187121	8.76	13.37	17.83	48.98	22.23	0.39	0.6	0.8	2.2	-23.64	19.51	T	-14.3	-40	-32.67	-18.63	0	T	21.67	47.59	28.16	10.6	0
	mean concentration per fraction $\bar{c}_{j,k}$										0.52	0.73	0.92	1.84				C	A	G	U	T		C	A	G	U	T

Supplementary Table 55*: Raw data Extended Data Fig. 5m – nucleobases in water, 170 µm, 18h, pH 11*

repeat k	species	measured values (µAU*min)				concentration (µM) $[A]_{j,k,HPLC}$ calibration acc. to Supplementary Table 3				c_0 (µM)	Normalization (Eq. 1) concentration (x c_0)				Ratio species vs mean (Eq. 4) $[A]_{j,k}/\bar{c}_{j,k} - 1$ (%)		Ratio species against species (shown in heat maps), Eq. 2 $[A]_{j,k}/[B]_{j,k} - 1$ (%)											
		top	II	III	bot	top	II	III	bot		top	II	III	bot	top	bot	top part (blue shade)						bot part (red shade)					
1	C	847.2	1044.95	1175.07	1924.17	18.63	22.97	25.83	42.3	27.43	0.68	0.84	0.94	1.54	-7.73	6.73	C	0	-21.23	-15.31	-4.46	8.5	C	0	33.77	18.07	1.88	-9.81
	A	2852.06	3217.89	3351.72	3814.29	25.1	28.32	29.5	33.57	29.12	0.86	0.97	1.01	1.15	17.14	-20.22	A	26.95	0	7.52	21.29	37.74	A	-25.25	0	-11.74	-23.84	-32.58
	G	767.81	873.76	938.77	1250.91	35.57	40.48	43.49	57.95	44.37	0.8	0.91	0.98	1.31	8.95	-9.6	G	18.07	-6.99	0	12.8	28.11	G	-15.3	13.3	0	-13.71	-23.61
	U	1471.72	1732.71	1944.93	3134.5	20.39	24	26.94	43.42	28.69	0.71	0.84	0.94	1.51	-3.42	4.76	U	4.67	-17.55	-11.35	0	13.57	U	-1.84	31.3	15.89	0	-11.47
	T	1078.88	1333.57	1536.5	2947.67	16.94	20.94	24.13	46.29	27.08	0.63	0.77	0.89	1.71	-14.95	18.33	T	-7.83	-27.4	-21.94	-11.95	0	T	10.87	48.31	30.91	12.96	0
	mean concentration per fraction $\bar{c}_{j,k}$										0.74	0.87	0.95	1.44				C	A	G	U	T		C	A	G	U	T
2	C	1074.82	1323.56	1516.1	2060.58	23.63	29.1	33.33	45.3	32.84	0.72	0.89	1.01	1.38	-15.14	26.82	C	0	-29.84	-25.12	-12.8	1.62	C	0	72.97	48.56	29.72	8.65
	A	3416.61	3549.74	3702.11	2656.81	30.07	31.24	32.58	23.38	29.32	1.03	1.07	1.11	0.8	20.96	-26.68	A	42.54	0	6.73	24.29	44.84	A	-42.19	0	-14.11	-25	-37.19
	G	900.08	996.78	980.14	869.76	41.7	46.18	45.41	40.3	43.4	0.96	1.06	1.05	0.93	13.33	-14.63	G	33.55	-6.31	0	16.45	35.7	G	-32.69	16.43	0	-12.68	-26.87
	U	1922.98	2297.05	2623.34	2478.06	26.64	31.82	36.34	34.33	32.28	0.83	0.99	1.13	1.06	-2.68	-2.24	U	14.68	-19.54	-14.13	0	16.54	U	-22.91	33.34	14.52	0	-16.25
	T	1492.98	1960.11	2303.71	2676.99	23.45	30.78	36.18	42.04	33.11	0.71	0.93	1.09	1.27	-16.49	16.73	T	-1.59	-30.96	-26.31	-14.19	0	T	-7.96	59.2	36.73	19.4	0
	mean concentration per fraction $\bar{c}_{j,k}$										0.85	0.99	1.08	1.09				C	A	G	U	T		C	A	G	U	T
3	C	915.17	1156.35	1271.71	1702.06	20.12	25.42	27.96	37.42	27.73	0.73	0.92	1.01	1.35	-7.29	10.49	C	0	-19.37	-13.99	-3.22	4.47	C	0	32.68	22.31	8.03	-2.76
	A	2829.36	3306.38	3242.91	3197.66	24.9	29.1	28.54	28.14	27.67	0.9	1.05	1.03	1.02	14.98	-16.72	A	24.03	0	6.68	20.03	29.58	A	-24.63	0	-7.81	-18.58	-26.71
	G	744.93	894.14	919	974.27	34.51	41.42	42.58	45.14	40.91	0.84	1.01	1.04	1.1	7.78	-9.67	G	16.26	-6.26	0	12.51	21.46	G	-18.24	8.48	0	-11.67	-20.5
	U	1547.06	1955.65	2173.75	2577.41	21.43	27.09	30.11	35.7	28.58	0.75	0.95	1.05	1.25	-4.21	2.27	U	3.33	-16.69	-11.12	0	7.96	U	-7.44	22.81	13.22	0	-9.99
	T	1256.39	1607.21	1862.28	2510.52	19.73	25.24	29.25	39.43	28.41	0.69	0.89	1.03	1.39	-11.26	13.63	T	-4.28	-22.83	-17.67	-7.37	0	T	2.84	36.45	25.78	11.1	0
	mean concentration per fraction $\bar{c}_{j,k}$										0.78	0.96	1.03	1.22				C	A	G	U	T		C	A	G	U	T

Supplementary Table 56*: Raw data Extended Data Fig. 5n – 2’,3’-Ribonucleotides in water, 170µm, 18h*

repeat k	species	measured values (mAU*min)				concentration (µM) $[A]_{j,k,HPLC}$ calibration acc. to Supplementary Table 3				c_0 (µM)	Normalization (Eq. 1) concentration (x c_0)				Ratio species vs mean (Eq. 4) $[A]_{j,k}/\bar{c}_{j,k}-1$ (%)		Ratio species against species (shown in heat maps), Eq. 2 $[A]_{j,k}/[B]_{j,k}-1$ (%)									
		top	II	III	bot	top	II	III	bot		top	II	III	bot	top	bot	top part (blue shade)					bot part (red shade)				
1	C	0.461	0.854	1.775	4.786	6.08	11.26	23.39	63.08	25.95	0.23	0.43	0.9	2.43	-21.54	8.83	C	0	-21.52	-27.71	-30.59	C	0	8.78	12.96	14.84
	U	0.677	1.138	2.190	5.069	7.71	12.95	24.92	57.68	25.82	0.3	0.5	0.97	2.23	-0.02	0.05	U	27.42	0	-7.88	-11.55	U	-8.07	0	3.84	5.56
	A	0.858	1.419	2.615	5.695	8.19	13.55	24.96	54.36	25.27	0.32	0.54	0.99	2.15	8.53	-3.65	A	38.32	8.56	0	-3.98	A	-11.47	-3.7	0	1.66
	G	1.139	1.858	3.360	7.145	8.61	14.05	25.4	54.01	25.52	0.34	0.55	1	2.12	13.03	-5.23	G	44.06	13.06	4.15	0	G	-12.92	-5.27	-1.63	0
	mean concentration per fraction $\bar{c}_{j,k}$										0.3	0.51	0.96	2.23				C	U	A	G		C	U	A	G
2	C	0.434	0.556	1.101	4.556	5.72	7.33	14.52	60.05	21.91	0.26	0.33	0.66	2.74	-23.15	9.54	C	0	-23.49	-29.97	-31.96	C	0	9.73	13.8	16.08
	U	0.674	0.806	1.487	4.935	7.67	9.17	16.92	56.16	22.48	0.34	0.41	0.75	2.5	0.45	-0.17	U	30.71	0	-8.47	-11.07	U	-8.87	0	3.7	5.78
	A	0.867	1.016	1.817	5.603	8.28	9.7	17.34	53.48	22.2	0.37	0.44	0.78	2.41	9.75	-3.74	A	42.81	9.25	0	-2.84	A	-12.12	-3.57	0	2
	G	1.145	1.339	2.403	7.044	8.65	10.12	18.16	53.25	22.55	0.38	0.45	0.81	2.36	12.95	-5.63	G	46.98	12.45	2.92	0	G	-13.85	-5.47	-1.97	0
	mean concentration per fraction $\bar{c}_{j,k}$										0.34	0.41	0.75	2.5				C	U	A	G		C	U	A	G
3	C	0.607	0.098	2.198	4.354	8.01	1.29	28.98	57.4	23.92	0.33	0.05	1.21	2.4	-22.48	7.19	C	0	-23.37	-28.46	-31.39	C	0	7.47	10.43	11.67
	U	0.911	0.136	2.638	4.657	10.37	1.55	30.02	53	23.73	0.44	0.07	1.26	2.23	1.15	-0.26	U	30.49	0	-6.65	-10.47	U	-6.95	0	2.75	3.9
	A	1.130	0.173	3.110	5.250	10.79	1.65	29.69	50.12	23.06	0.47	0.07	1.29	2.17	8.35	-2.93	A	39.78	7.12	0	-4.1	A	-9.45	-2.68	0	1.12
	G	1.508	0.224	3.988	6.642	11.4	1.69	30.15	50.21	23.36	0.49	0.07	1.29	2.15	12.98	-4.01	G	45.75	11.69	4.27	0	G	-10.45	-3.76	-1.11	0
	mean concentration per fraction $\bar{c}_{j,k}$										0.43	0.07	1.26	2.24				C	U	A	G		C	U	A	G

Supplementary Table 57*: Raw data Extended Data Fig. 5o – 3',5'-Ribonucleotides in water, 170µm, 18h*

repeat k	species	measured values (mAU*min)				concentration (µM) $[A]_{j,k,HPLC}$ calibration acc. to Supplementary Table 3				c_0 (µM)	Normalization (Eq. 1) concentration (x c_0)				Ratio species vs mean (Eq. 4) $[A]_{j,k}/\bar{c}_{j,k}-1$ (%)		Ratio species against species (shown in heat maps), Eq. 2 $[A]_{j,k}/[B]_{j,k}-1$ (%)									
		top	II	III	bot	top	II	III	bot		top	II	III	bot	top	bot	top part (blue shade)					bot part (red shade)				
1	C	0.572	0.853	1.492	3.042	10.01	14.92	26.12	53.24	26.07	0.38	0.57	1	2.04	-4.64	-0.84	C	0	-11.56	-7.83	2.15	C	0	2.86	-1.04	-4.87
	A	1.568	2.129	3.577	7.166	11.39	15.47	25.99	52.07	26.23	0.43	0.59	0.99	1.99	7.83	-3.6	A	13.07	0	4.22	15.51	A	-2.78	0	-3.8	-7.51
	G	1.260	1.705	2.890	6.239	12.79	17.31	29.33	63.33	30.69	0.42	0.56	0.96	2.06	3.46	0.21	G	8.49	-4.05	0	10.82	G	1.06	3.95	0	-3.86
	U	0.858	1.203	2.170	4.900	8.29	11.62	20.96	47.33	22.05	0.38	0.53	0.95	2.15	-6.65	4.23	U	-2.11	-13.43	-9.77	0	U	5.12	8.13	4.02	0
	mean concentration per fraction $\bar{c}_{j,k}$										0.4	0.56	0.97	2.06				C	A	G	U		C	A	G	U
2	C	0.740	0.821	1.075	3.399	12.94	14.36	18.82	59.49	26.4	0.49	0.54	0.71	2.25	-4.73	3.16	C	0	-11.41	-3.33	-3.43	C	0	6.42	5.58	0.96
	A	2.005	2.135	2.683	7.672	14.57	15.51	19.5	55.74	26.33	0.55	0.59	0.74	2.12	7.53	-3.06	A	12.87	0	9.12	9.01	A	-6.03	0	-0.79	-5.13
	G	1.530	1.801	2.300	6.440	15.53	18.28	23.35	65.38	30.64	0.51	0.6	0.76	2.13	-1.45	-2.29	G	3.44	-8.36	0	-0.1	G	-5.28	0.8	0	-4.37
	U	1.152	1.248	1.613	5.063	11.12	12.05	15.58	48.9	21.92	0.51	0.55	0.71	2.23	-1.35	2.18	U	3.55	-8.26	0.11	0	U	-0.95	5.4	4.57	0
	mean concentration per fraction $\bar{c}_{j,k}$										0.51	0.57	0.73	2.18				C	A	G	U		C	A	G	U
3	C	0.363	0.469	0.865	3.282	6.36	8.21	15.13	57.45	21.79	0.29	0.38	0.69	2.64	-6.4	2.69	C	0	-11.04	-9.73	-3.99	C	0	5.75	4.76	0.51
	A	0.992	1.339	2.223	7.537	7.21	9.73	16.15	54.76	21.96	0.33	0.44	0.74	2.49	5.22	-2.89	A	12.41	0	1.48	7.93	A	-5.44	0	-0.93	-4.95
	G	0.806	1.107	1.782	6.270	8.18	11.24	18.09	63.65	25.29	0.32	0.44	0.72	2.52	3.69	-1.98	G	10.78	-1.46	0	6.36	G	-4.55	0.94	0	-4.06
	U	0.568	0.743	1.260	4.898	5.49	7.17	12.17	47.31	18.03	0.3	0.4	0.67	2.62	-2.51	2.17	U	4.16	-7.35	-5.98	0	U	-0.51	5.21	4.23	0
	mean concentration per fraction $\bar{c}_{j,k}$										0.31	0.42	0.7	2.57				C	A	G	U		C	A	G	U

Supplementary Table 58*: Raw data Extended Data Fig. 5p – 3',5'-Ribonucleotides in 10 % formamide, 170µm, 18h*

repeat k	species	measured values (µAU*min)				concentration (µM) $[A]_{j,k,HPLC}$ calibration acc. to Supplementary Table 3				c_0 (µM)	Normalization (Eq. 1) concentration (x c_0)				Ratio species vs mean (Eq. 4) $[A]_{j,k}/\bar{c}_{j,k}-1$ (%)		Ratio species against species (shown in heat maps), Eq. 2 $[A]_{j,k}/[B]_{j,k}-1$ (%)									
		top	II	III	bot	top	II	III	bot		top	II	III	bot	top	bot	top part (blue shade)					bot part (red shade)				
1	C	210.9	226.38	898.32	5455.3	3.69	3.96	15.72	95.48	29.71	0.12	0.13	0.53	3.21	-20.26	3.24	C	0	-21.9	-29.29	-24.33	C	0	3.62	5.34	4.15
	A	530.08	549.65	1916.11	10335.3	3.85	3.99	13.92	75.1	24.22	0.16	0.16	0.57	3.1	2.1	-0.37	A	28.04	0	-9.46	-3.11	A	-3.49	0	1.66	0.52
	G	474.41	487.03	1603.12	8237.32	4.82	4.94	16.27	83.62	27.41	0.18	0.18	0.59	3.05	12.78	-2	G	41.42	10.45	0	7.02	G	-5.07	-1.64	0	-1.13
	U	334.7	344.35	1186.14	6290.47	3.23	3.33	11.46	60.76	19.69	0.16	0.17	0.58	3.09	5.38	-0.88	U	32.15	3.21	-6.56	0	U	-3.99	-0.51	1.14	0
	mean concentration per fraction $\bar{c}_{j,k}$										0.16	0.16	0.57	3.11				C	A	G	U		C	A	G	U
2	C	209.7	242.48	473.76	4047.95	3.67	4.24	8.29	70.85	21.76	0.17	0.2	0.38	3.26	-21	4.65	C	0	-23.51	-29.53	-25.19	C	0	5.26	7.6	6.05
	A	551.76	614.38	1104.48	7740.06	4.01	4.46	8.03	56.24	18.18	0.22	0.25	0.44	3.09	3.28	-0.58	A	30.73	0	-7.87	-2.21	A	-5	0	2.22	0.75
	G	489.96	549.66	955.58	6194.96	4.97	5.58	9.7	62.89	20.78	0.24	0.27	0.47	3.03	12.1	-2.74	G	41.9	8.54	0	6.15	G	-7.06	-2.17	0	-1.44
	U	343.21	386.68	686.53	4673.27	3.32	3.74	6.63	45.14	14.71	0.23	0.25	0.45	3.07	5.61	-1.32	U	33.68	2.25	-5.79	0	U	-5.71	-0.75	1.46	0
	mean concentration per fraction $\bar{c}_{j,k}$										0.21	0.24	0.43	3.11				C	A	G	U		C	A	G	U
3	C	80.02	160.9	453.03	3774.96	1.4	2.82	7.93	66.07	19.55	0.07	0.14	0.41	3.38	-28.09	5.58	C	0	-29.79	-39.4	-32.8	C	0	6.25	9.38	7.15
	A	238.28	470.59	1206.02	7428.57	1.73	3.42	8.76	53.98	16.97	0.1	0.2	0.52	3.18	2.42	-0.63	A	42.43	0	-13.69	-4.28	A	-5.88	0	2.95	0.85
	G	229.2	444.43	1093.1	5990.42	2.33	4.51	11.1	60.81	19.69	0.12	0.23	0.56	3.09	18.67	-3.48	G	65.02	15.86	0	10.9	G	-8.58	-2.87	0	-2.04
	U	153.36	301.69	763.24	4537.76	1.48	2.91	7.37	43.83	13.9	0.11	0.21	0.53	3.15	7	-1.47	U	48.8	4.48	-9.83	0	U	-6.67	-0.84	2.09	0
	mean concentration per fraction $\bar{c}_{j,k}$										0.1	0.2	0.5	3.2				C	A	G	U		C	A	G	U

Supplementary Table 59*: Raw data Extended Data Fig. 5q – cytidine and CMPs in water, 170µm, 18h*

repeat k	species	measured values (µAU*s)				concentration (µM) $[A]_{j,k,HPLC}$ calibration acc. to Supplementary Table 3				c_0 (µM)	Normalization (Eq. 1) concentration (x c_0)				Ratio species vs mean (Eq. 4) $[A]_{j,k}/\bar{c}_{j,k}-1$ (%)		Ratio species against species (shown in heat maps), Eq. 2 $[A]_{j,k}/[B]_{j,k}-1$ (%)													
		top	II	III	bot	top	II	III	bot		top	II	III	bot	top	bot	top part (blue shade)							bot part (red shade)						
1	Cyd	42272	51612	96405	150254	11.92	14.56	27.19	42.38	24.01	0.5	0.61	1.13	1.76	38.55	-16.19	Cyd	0	40.71	70.98	74.9	71.78	70.85	Cyd	0	-15.76	-23.83	-21.86	-22.05	-23.86
	5'	27246	37613	82222	161771	9.82	13.56	29.65	58.33	27.84	0.35	0.49	1.06	2.1	-1.53	-0.5	5'	-28.93	0	21.51	24.3	22.08	21.42	5'	18.71	0	-9.58	-7.24	-7.46	-9.62
	2',3'	38228	54265	129048	305021	8.4	11.92	28.35	67.01	28.92	0.29	0.41	0.98	2.32	-18.96	10.04	2',3'	-41.51	-17.7	0	2.29	0.47	-0.08	2',3'	31.29	10.59	0	2.59	2.34	-0.04
	3'	21969	32865	79928	174786	8	11.97	29.11	63.66	28.19	0.28	0.42	1.03	2.26	-20.78	7.26	3'	-42.83	-19.55	-2.24	0	-1.78	-2.32	3'	27.97	7.8	-2.52	0	-0.24	-2.56
	2'	30647	45424	107994	240051	7.9	11.71	27.85	61.9	27.34	0.29	0.43	1.02	2.26	-19.34	7.52	2'	-41.79	-18.09	-0.47	1.82	0	-0.54	2'	28.28	8.06	-2.29	0.24	0	-2.33
	3',5'	28104	39954	94591	224158	8.2	11.65	27.59	65.39	28.21	0.29	0.41	0.98	2.32	-18.9	10.08	3',5'	-41.47	-17.64	0.08	2.37	0.55	0	3',5'	31.34	10.64	0.04	2.63	2.39	0
	mean concentration per fraction $\bar{c}_{j,k}$										0.33	0.46	1.03	2.17				Cyd	5'	2',3'	3'	2'	3'.5'		Cyd	5'	2',3'	3'	2'	3'.5'
2	Cyd	51275	66015	85799	127640	14.46	18.62	24.2	36	23.32	0.62	0.8	1.04	1.54	28.2	-15.99	Cyd	0	28.18	47.21	49.8	46.28	48.09	Cyd	0	-15.52	-23.4	-21.13	-21.13	-23.37
	5'	35711	51897	72768	134876	12.88	18.71	26.24	48.64	26.62	0.48	0.7	0.99	1.83	0.02	-0.56	5'	-21.99	0	14.84	16.86	14.12	15.53	5'	18.37	0	-9.33	-6.65	-6.65	-9.29
	2',3'	52960	79518	117025	253354	11.64	17.47	25.71	55.66	27.62	0.42	0.63	0.93	2.02	-12.91	9.68	2',3'	-32.07	-12.92	0	1.76	-0.63	0.6	2',3'	30.55	10.29	0	2.96	2.96	0.04
	3'	30308	48361	70868	143297	11.04	17.61	25.81	52.19	26.66	0.41	0.66	0.97	1.96	-14.41	6.52	3'	-33.24	-14.43	-1.73	0	-2.35	-1.14	3'	26.79	7.12	-2.87	0	0	-2.83
	2'	42779	66345	96993	197515	11.03	17.11	25.01	50.93	26.02	0.42	0.66	0.96	1.96	-12.36	6.52	2'	-31.64	-12.37	0.63	2.4	0	1.24	2'	26.79	7.12	-2.87	0	0	-2.83
	3'.5'	38507	58379	85695	185237	11.23	17.03	25	54.03	26.82	0.42	0.63	0.93	2.01	-13.43	9.63	3',5'	-32.47	-13.44	-0.6	1.15	-1.22	0	3',5'	30.49	10.24	-0.04	2.92	2.92	0
	mean concentration per fraction $\bar{c}_{j,k}$										0.46	0.68	0.97	1.89				Cyd	5'	2',3'	3'	2'	3'.5'		Cyd	5'	2',3'	3'	2'	3'.5'
	Cyd	31562	52630	111168	128850	8.9	14.84	31.36	36.34	22.86	0.39	0.65	1.37	1.59	49.43	-15.12	Cyd	0	53.5	98.74	109.27	101.86	98.9	Cyd	0	-15.08	-22.19	-20.38	-20.44	-22.29
	5'	18328	35547	99873	135250	6.61	12.82	36.01	48.77	26.05	0.25	0.49	1.38	1.87	-2.65	-0.05	5'	-34.85	0	29.47	36.33	31.5	29.58	5'	17.76	0	-8.38	-6.24	-6.31	-8.5
	2',3'	24293	49911	168414	253322	5.34	10.97	37	55.65	27.24	0.2	0.4	1.36	2.04	-24.81	9.09	2',3'	-49.68	-22.76	0	5.3	1.57	0.08	2',3'	28.52	9.14	0	2.33	2.26	-0.13
	3'	13258	29386	100092	142260	4.83	10.7	36.46	51.82	25.95	0.19	0.41	1.4	2	-28.59	6.61	3'	-52.21	-26.65	-5.03	0	-3.54	-4.95	3'	25.6	6.66	-2.28	0	-0.07	-2.4
	2'	19069	41558	137263	197511	4.92	10.72	35.39	50.93	25.49	0.19	0.42	1.39	2	-25.97	6.68	2'	-50.46	-23.96	-1.54	3.67	0	-1.46	2'	25.69	6.74	-2.21	0.07	0	-2.33
	3'.5'	17852	36472	123870	186550	5.21	10.64	36.13	54.42	26.6	0.2	0.4	1.36	2.05	-24.87	9.23	3',5'	-49.72	-22.82	-0.08	5.21	1.49	0	3',5'	28.69	9.29	0.13	2.46	2.39	0
	mean concentration per fraction $\bar{c}_{j,k}$										0.24	0.46	1.38	1.93				Cyd	5'	2',3'	3'	2'	3'.5'		Cyd	5'	2',3'	3'	2'	3'.5'

***Supplementary Table 60**: Raw data Extended Data Fig. 5r – Deoxy- vs 5'-Ribonucleotides in water, 170µm, 18h*

repeat k	species	measured values (µAU*min)				concentration (µM) calibration acc. to Supplementary Table 3				c_0 (µM)	Normalization (Eq. 1) concentration (x c_0)			
		top	II	III	bot	top	II	III	bot		top	II	III	bot
1	5'CMP	169.74	424.53	839.37	3350.67	3.67	9.19	18.16	72.49	25.88	0.14	0.35	0.7	2.8
	dCMP	175.41	461.99	958.96	4073.06	3.32	8.74	18.14	77.06	26.81	0.12	0.33	0.68	2.87
	5'GMP	346.97	784.07	1329.61	4453.89	4.13	9.33	15.81	52.97	20.56	0.2	0.45	0.77	2.58
	dGMP	511.85	1208.13	2131.29	7815.32	4.87	11.5	20.29	74.4	27.77	0.18	0.41	0.73	2.68
	5'AMP	1317.97	1984.17	3279.2	7064.98	12.62	19	31.4	67.64	32.66	0.39	0.58	0.96	2.07
	dAMP	571.02	858.51	1432.45	3071.21	10.07	15.14	25.26	54.16	26.16	0.38	0.58	0.97	2.07
	5'UMP	549.3	837.71	1430.5	3220.96	10.81	16.49	28.16	63.41	29.72	0.36	0.55	0.95	2.13
	dTMP	390.43	618.95	1109.22	2846.79	5.23	8.29	14.86	38.14	16.63	0.31	0.5	0.89	2.29
	mean concentration per fraction $\bar{c}_{j,k}$										0.26	0.47	0.83	2.44
2	5'CMP	347.18	704.67	1116	3159.95	7.51	15.25	24.15	68.37	28.82	0.26	0.53	0.84	2.37
	dCMP	398.3	819.88	1312.14	3827.4	7.54	15.51	24.82	72.41	30.07	0.25	0.52	0.83	2.41
	5'GMP	540.73	1015.7	1718.89	4146.49	6.43	12.08	20.44	49.31	22.07	0.29	0.55	0.93	2.23
	dGMP	845.22	1671.92	2820.86	7391.98	8.05	15.92	26.86	70.37	30.3	0.27	0.53	0.89	2.32
	5'AMP	1534.83	1588.98	2664.24	5930.75	14.7	15.21	25.51	56.78	28.05	0.52	0.54	0.91	2.02
	dAMP	696.47	699.71	1161.9	2576.55	12.28	12.34	20.49	45.44	22.64	0.54	0.55	0.91	2.01
	5'UMP	671.53	686.52	1147.4	2710.09	13.22	13.52	22.59	53.35	25.67	0.52	0.53	0.88	2.08
	dTMP	485.15	489.83	853.87	2383.49	6.5	6.56	11.44	31.94	14.11	0.46	0.47	0.81	2.26
	mean concentration per fraction $\bar{c}_{j,k}$										0.39	0.52	0.87	2.21
3	5'CMP	1127.31	1294.99	1410.79	1684.55	24.39	28.02	30.52	36.45	29.84	0.82	0.94	1.02	1.22
	dCMP	1335.34	1545.51	1689.73	2018.81	25.26	29.24	31.97	38.19	31.16	0.81	0.94	1.03	1.23
	5'GMP	1560.59	1729.83	1894.02	2331.29	18.56	20.57	22.53	27.73	22.35	0.83	0.92	1.01	1.24
	dGMP	2661.51	2988.6	3285.33	4048.55	25.34	28.45	31.28	38.54	30.9	0.82	0.92	1.01	1.25
	5'AMP	1819.02	2778.42	3823.17	6974.22	17.42	26.6	36.6	66.77	36.85	0.47	0.72	0.99	1.81

	dAMP	776.81	1200.06	1677.78	3036.55	13.7	21.16	29.59	53.55	29.5	0.46	0.72	1	1.82
	5'UMP	776.61	1210.37	1702.05	3175.12	15.29	23.83	33.51	62.51	33.78	0.45	0.71	0.99	1.85
	dTMP	585.64	951.06	1401.87	2761.97	7.85	12.74	18.78	37.01	19.09	0.41	0.67	0.98	1.94
	mean concentration per fraction $\bar{c}_{j,k}$										0.63	0.82	1.01	1.54

***Supplementary Table 61**: Raw data Extended Data Fig. 5s-u – Chirality of amino acids and nucleoside in water, 170µm, 18h*
Each compound was analysed in a separate run. Due to technical problems, for the third replicate of cytidine chiral separation the temperature gradient was not applied for the entire duration of the experiment, which is why we do not take it into account for the calculation of the heat flow-driven enrichment.

		Raw data (counts*min for Amino acids, µAU*min for cytidine)			concentrations (µM)			Normalization (Eq. 1) concentration (x c_0)		
		Repeat 1	Repeat 2	Repeat 3	Repeat 1	Repeat 2	Repeat 3	Repeat 1	Repeat 2	Repeat 3
L-Serine	top	357762.18	389775.31	392169.21	72.55	79.01	79.49	0.88	0.91	0.95
	II	399559.36	425184.41	392717.48	80.99	86.16	79.60	0.98	0.99	0.95
	III	439297.54	435742.96	429217.61	89.01	88.29	86.97	1.08	1.02	1.04
	bottom	437473.30	465881.68	443719.48	88.64	94.37	89.90	1.07	1.09	1.07
	c_0 (µM)				82.79	86.96	83.99			
D-Serine	top	361647.23	378348.22	414610.32	73.33	76.70	84.02	0.93	0.93	1.04
	II	385201.82	399703.98	405301.24	78.09	81.01	82.14	0.99	0.98	1.01
	III	389921.03	422252.97	347992.72	79.04	85.57	70.57	1.00	1.03	0.87
	bottom	422312.42	433740.64	430845.06	85.58	87.89	87.30	1.08	1.06	1.08
	c_0 (µM)				79.01	82.79	81.01			
L-Threonine	top	387210.64	364486.05	390897.41	70.63	66.51	71.30	0.92	0.90	0.90
	II	412346.73	399252.18	405525.21	75.20	72.82	73.96	0.98	0.98	0.93
	III	432729.36	407077.01	460181.63	78.90	74.24	83.88	1.03	1.00	1.06
	bottom	451308.93	456273.54	480216.74	82.27	83.17	87.51	1.07	1.12	1.11
	c_0 (µM)				76.75	74.18	79.16			
D-Threonine	top	343706.88	419771.78	370256.49	62.74	76.54	67.56	0.81	0.98	0.82
	II	407149.61	431178.24	440857.08	74.25	78.61	80.37	0.96	1.01	0.97
	III	509280.53	436664.40	484323.39	82.78	79.61	88.26	1.07	1.02	1.07
	bottom	487535.27	425516.43	518873.29	88.84	77.59	94.52	1.15	0.99	1.14
	c_0 (µM)				77.15	78.09	82.68			
L-Cytidine	top	628.48	603.99		10.64	10.22		0.36	0.41	
	II	950.74	994.42		16.09	16.83		0.54	0.68	
	III	2251.66	1231.55		38.11	20.84		1.27	0.84	
	bottom	3248.43	3007.97		54.97	50.90		1.84	2.06	
	c_0 (µM)				29.95	24.70				
D-Cytidine	top	796.67	619.49		13.48	10.48		0.55	0.44	
	II	791.50	788.39		13.39	13.34		0.55	0.57	
	III	1609.61	1485.40		27.24	25.14		1.11	1.07	
	bottom	2594.78	2681.85		43.91	45.39		1.79	1.92	
	c_0 (µM)				24.51	23.59				

Supplementary Table 62: Raw data Supplementary Fig. 1a – nucleobases in water, 170µm, 6h

repeat k	species	measured values (µAU*min)				concentration (µM) $[A]_{j,k,HPLC}$ calibration acc. to Supplementary Table 3				c_0 (µM)	Normalization (Eq. 1) concentration (x c_0)				Ratio species vs mean (Eq. 4) $[A]_{j,k}/\bar{c}_{j,k}-1$ (%)		Ratio species against species (shown in heat maps), Eq. 2 $[A]_{j,k}/[B]_{j,k}-1$ (%)											
		top	II	III	bot	top	II	III	bot		top	II	III	bot	top	bot	top part (blue shade)						bot part (red shade)					
1	C	864.79	975.25	1002.41	1259.33	19.01	21.44	22.04	27.69	22.54	0.84	0.95	0.98	1.23	-7.05	6.16	C	0	-11.12	-11.61	-9.5	-1.73	C	0	11.98	10.79	8.93	0.39
	A	2412.8	2528.86	2441.54	2788.88	21.24	22.26	21.49	24.55	22.38	0.95	0.99	0.96	1.1	4.58	-5.2	A	12.51	0	-0.56	1.82	10.56	A	-10.7	0	-1.06	-2.72	-10.36
	G	605.61	606.41	623.29	703.54	28.06	28.09	28.88	32.59	29.41	0.95	0.96	0.98	1.11	5.17	-4.18	G	13.14	0.56	0	2.39	11.18	G	-9.74	1.07	0	-1.68	-9.39
	U	1504.64	1580.34	1553.3	1820.32	20.84	21.89	21.52	25.21	22.37	0.93	0.98	0.96	1.13	2.71	-2.54	U	10.5	-1.78	-2.34	0	8.59	U	-8.2	2.8	1.71	0	-7.85
	T	1284.43	1420.57	1450.77	1831.01	20.17	22.31	22.78	28.76	23.51	0.86	0.95	0.97	1.22	-5.41	5.76	T	1.76	-9.55	-10.06	-7.91	0	T	-0.38	11.55	10.37	8.51	0
	mean concentration per fraction $\bar{c}_{j,k}$										0.91	0.97	0.97	1.16				C	A	G	U	T		C	A	G	U	T
2	C	816.79	851.82	1066.63	1345.31	17.96	18.73	23.45	29.58	22.43	0.8	0.84	1.05	1.32	-4.81	5.25	C	0	-8.14	-8.55	-6.09	-0.58	C	0	11.06	8.18	7.1	0.81
	A	2273.86	2305.06	2758.81	3097.88	20.01	20.29	24.28	27.26	22.96	0.87	0.88	1.06	1.19	3.62	-5.23	A	8.86	0	-0.46	2.23	8.22	A	-9.96	0	-2.59	-3.57	-9.23
	G	659.46	651.61	783.55	918.13	30.55	30.19	36.3	42.54	34.89	0.88	0.87	1.04	1.22	4.09	-2.71	G	9.35	0.46	0	2.7	8.71	G	-7.56	2.66	0	-1	-6.82
	U	1404.32	1421.75	1734.34	2028.18	19.45	19.69	24.02	28.09	22.82	0.85	0.86	1.05	1.23	1.36	-1.73	U	6.48	-2.18	-2.63	0	5.86	U	-6.63	3.7	1.01	0	-5.88
	T	1197.28	1252.66	1551.7	1944.81	18.8	19.67	24.37	30.54	23.35	0.81	0.84	1.04	1.31	-4.25	4.41	T	0.59	-7.6	-8.02	-5.54	0	T	-0.8	10.17	7.32	6.24	0
	mean concentration per fraction $\bar{c}_{j,k}$										0.84	0.86	1.05	1.25				C	A	G	U	T		C	A	G	U	T
3	C	768.53	1011.98	1147.02	1443.66	16.9	22.25	25.22	31.74	24.03	0.7	0.93	1.05	1.32	-9.37	7.51	C	0	-17.5	-13.24	-12.59	-0.79	C	0	18.81	9.21	11.86	0.04
	A	2322.49	2709.67	2836.66	3029.33	20.44	23.85	24.97	26.66	23.98	0.85	0.99	1.04	1.11	9.86	-9.52	A	21.21	0	5.16	5.95	20.25	A	-15.84	0	-8.08	-5.85	-15.8
	G	551.09	624.69	721.21	822.36	25.53	28.94	33.41	38.1	31.5	0.81	0.92	1.06	1.21	4.47	-1.56	G	15.26	-4.91	0	0.76	14.35	G	-8.43	8.79	0	2.43	-8.4
	U	1405.7	1703.18	1816.61	2063.46	19.47	23.59	25.16	28.58	24.2	0.8	0.97	1.04	1.18	3.68	-3.89	U	14.4	-5.62	-0.75	0	13.49	U	-10.6	6.22	-2.37	0	-10.57
	T	1140.79	1492	1679.14	2125.05	17.92	23.43	26.37	33.37	25.27	0.71	0.93	1.04	1.32	-8.64	7.46	T	0.8	-16.84	-12.55	-11.89	0	T	-0.04	18.77	9.17	11.82	0
	mean concentration per fraction $\bar{c}_{j,k}$										0.78	0.95	1.05	1.23				C	A	G	U	T		C	A	G	U	T

***Supplementary Table 63**: Raw data Supplementary Fig. 1b – Ribonucleosides in water, 170µm, 6h*

repeat k	species	measured values (µAU*s)				concentration (µM) $[A]_{j,k,HPLC}$ calibration acc. to Supplementary Table 3				c_0 (µM)	Normalization (Eq. 1) concentration (x c_0)				Ratio species vs mean (Eq. 4) $[A]_{j,k}/\bar{c}_{j,k}-1$ (%)		Ratio species against species (shown in heat maps), Eq. 2 $[A]_{j,k}/[B]_{j,k}-1$ (%)									
		top	II	III	bot	top	II	III	bot		top	II	III	bot	top	bot	top part (blue shade)					bot part (red shade)				
1	C	43670	60769	79848	103677	12.32	17.14	22.52	29.24	20.31	0.61	0.84	1.11	1.44	-6.32	4.53	C	0	-6.21	-9.11	-9.36	C	0	6.03	7.72	4.69
	U	72563	98382	125445	152384	13.35	18.1	23.08	28.04	20.64	0.65	0.88	1.12	1.36	-0.12	-1.42	U	6.62	0	-3.09	-3.36	U	-5.69	0	1.59	-1.27
	A	116192	153813	193612	232747	13.7	18.14	22.84	27.45	20.53	0.67	0.88	1.11	1.34	3.07	-2.96	A	10.03	3.19	0	-0.28	A	-7.17	-1.57	0	-2.81
	G	94862	119535	157580	194984	11.94	15.04	19.83	24.53	17.83	0.67	0.84	1.11	1.38	3.36	-0.15	G	10.33	3.48	0.28	0	G	-4.48	1.28	2.9	0
	mean concentration per fraction $\bar{c}_{j,k}$										0.65	0.86	1.11	1.38				C	U	A	G		C	U	A	G
2	C	45386	53015	83308	102582	12.8	14.95	23.5	28.93	20.05	0.64	0.75	1.17	1.44	-8.69	5.21	C	0	-7.68	-10.75	-15.06	C	0	6.44	8.84	5.96
	U	77828	87844	131826	152584	14.32	16.16	24.25	28.07	20.7	0.69	0.78	1.17	1.36	-1.1	-1.16	U	8.31	0	-3.33	-8	U	-6.05	0	2.26	-0.44
	A	124498	137146	203619	230750	14.68	16.18	24.02	27.22	20.52	0.72	0.79	1.17	1.33	2.3	-3.34	A	12.04	3.44	0	-4.83	A	-8.12	-2.21	0	-2.64
	G	108841	111938	161113	197196	13.69	14.08	20.27	24.81	18.22	0.75	0.77	1.11	1.36	7.49	-0.71	G	17.73	8.69	5.08	0	G	-5.63	0.45	2.71	0
	mean concentration per fraction $\bar{c}_{j,k}$										0.7	0.77	1.16	1.37				C	U	A	G		C	U	A	G
3	C	54968	80890	95863	100879	15.5	22.82	27.04	28.45	23.45	0.66	0.97	1.15	1.21	-11.1	5.45	C	0	-8.75	-12.33	-20.83	C	0	5.53	7.7	9.01
	U	94953	130666	147991	150686	17.47	24.04	27.23	27.72	24.11	0.72	1	1.13	1.15	-2.58	-0.08	U	9.58	0	-3.93	-13.24	U	-5.24	0	2.05	3.3
	A	152403	202532	225824	227672	17.98	23.89	26.64	26.85	23.84	0.75	1	1.12	1.13	1.4	-2.09	A	14.07	4.09	0	-9.69	A	-7.15	-2.01	0	1.22
	G	136090	162275	172172	181384	17.12	20.42	21.66	22.82	20.51	0.84	1	1.06	1.11	12.29	-3.27	G	26.31	15.27	10.73	0	G	-8.27	-3.19	-1.2	0
	mean concentration per fraction $\bar{c}_{j,k}$										0.74	0.99	1.11	1.15				C	U	A	G		C	U	A	G

***Supplementary Table 64**: Raw data Supplementary Fig. 1c – 5'-Ribonucleotides in water, 170µm, 6h*

repeat k	species	measured values (µAU*min)				concentration (µM) $[A]_{j,k,HPLC}$ calibration acc. to Supplementary Table 3				c_0 (µM)	Normalization (Eq. 1) concentration (x c_0)				Ratio species vs mean (Eq. 4) $[A]_{j,k}/\bar{c}_{j,k}-1$ (%)		Ratio species against species (shown in heat maps), Eq. 2 $[A]_{j,k}/[B]_{j,k}-1$ (%)									
		top	II	III	bot	top	II	III	bot		top	II	III	bot	top	bot	top part (blue shade)					bot part (red shade)				
1	C	696.45	845.29	1434.23	1860	15.07	18.29	31.03	40.24	26.16	0.58	0.7	1.19	1.54	-2.26	1.28	C	0	-4.86	-4.1	0.11	C	0	3.95	3.22	-1.84
	A	1708.37	2052.63	3349.07	4175.74	16.36	19.65	32.07	39.98	27.01	0.61	0.73	1.19	1.48	2.73	-2.57	A	5.11	0	0.8	5.23	A	-3.8	0	-0.7	-5.58
	U	759.45	902.05	1511.3	1884.44	14.95	17.76	29.75	37.1	24.89	0.6	0.71	1.2	1.49	1.91	-1.88	U	4.28	-0.79	0	4.39	U	-3.12	0.71	0	-4.91
	G	1047.5	1269.42	2111.62	2853.39	12.46	15.1	25.11	33.94	21.65	0.58	0.7	1.16	1.57	-2.38	3.18	G	-0.11	-4.97	-4.21	0	G	1.88	5.9	5.16	0
	mean concentration per fraction $\bar{c}_{j,k}$										0.59	0.71	1.18	1.52				C	A	U	G		C	A	U	G
2	C	615.85	823.69	1033.93	1937.44	13.32	17.82	22.37	41.92	23.86	0.56	0.75	0.94	1.76	-3.86	2.32	C	0	-5.31	-5.85	-4.05	C	0	4.15	4.44	0.85
	A	1512.3	1991.69	2426.84	4325.17	14.48	19.07	23.24	41.41	24.55	0.59	0.78	0.95	1.69	1.54	-1.76	A	5.61	0	-0.57	1.34	A	-3.99	0	0.28	-3.18
	U	677.73	883.36	1087	1921.89	13.34	17.39	21.4	37.84	22.49	0.59	0.77	0.95	1.68	2.12	-2.03	U	6.22	0.57	0	1.92	U	-4.26	-0.28	0	-3.45
	G	941.98	1204.25	1507.81	2819.62	11.2	14.32	17.93	33.53	19.25	0.58	0.74	0.93	1.74	0.2	1.46	G	4.22	-1.32	-1.88	0	G	-0.84	3.28	3.57	0
	mean concentration per fraction $\bar{c}_{j,k}$										0.58	0.76	0.94	1.72				C	A	U	G		C	A	U	G
3	C	805.06	795.1	1085.33	1504.84	17.42	17.2	23.48	32.56	22.67	0.77	0.76	1.04	1.44	-0.99	0.53	C	0	-1.69	-4.05	2	C	0	1.51	2.52	-1.8
	A	1934.27	1919.71	2542.18	3501.43	18.52	18.38	24.34	33.52	23.69	0.78	0.78	1.03	1.42	0.72	-0.97	A	1.72	0	-2.4	3.75	A	-1.49	0	0.99	-3.27
	U	874.1	846.43	1115.75	1529.18	17.21	16.66	21.97	30.1	21.49	0.8	0.78	1.02	1.4	3.19	-1.94	U	4.22	2.46	0	6.3	U	-2.46	-0.98	0	-4.22
	G	1182.45	1194.79	1604.51	2295.72	14.06	14.21	19.08	27.3	18.66	0.75	0.76	1.02	1.46	-2.92	2.38	G	-1.96	-3.61	-5.93	0	G	1.83	3.38	4.4	0
	mean concentration per fraction $\bar{c}_{j,k}$										0.78	0.77	1.03	1.43				C	A	U	G		C	A	U	G

Supplementary Table 65*: Raw data Supplementary Fig. 1d – 2',3'-Ribonucleotides in water, 170µm, 6h*

rep eat k	spe cies	measured values (mAU*min)				concentration (µM) $[A]_{j,k,HPLC}$ calibration acc. to Supplementary Table 3				c_0 (µM)	Normalization (Eq. 1) concentration (x c_0)				Ratio species vs mean (Eq. 4) $[A]_{j,k}/\bar{c}_{j,k}-1$ (%)		Ratio species against species (shown in heat maps), Eq. 2 $[A]_{j,k}/[B]_{j,k}-1$ (%)									
		top	II	III	bot	top	II	III	bot		top	II	III	bot	top	bot	top part (blue shade)					bot part (red shade)				
	C	0.991	1.289	1.631	4.296	13.07	17	21.5	56.63	27.05	0.48	0.63	0.79	2.09	-8.1	6.25	C	0	-8.2	-11.82	-11.43	C	0	6.41	9.26	9.94
	U	1.254	1.599	1.988	4.687	14.27	18.2	22.63	53.34	27.11	0.53	0.67	0.83	1.97	0.11	-0.15	U	8.93	0	-3.94	-3.52	U	-6.02	0	2.68	3.32
1	A	1.533	1.919	2.378	5.361	14.63	18.32	22.7	51.18	26.71	0.55	0.69	0.85	1.92	4.22	-2.76	A	13.4	4.11	0	0.44	A	-8.48	-2.61	0	0.62
	G	2.027	2.555	3.206	7.079	15.33	19.31	24.24	53.51	28.1	0.55	0.69	0.86	1.9	3.76	-3.35	G	12.9	3.65	-0.44	0	G	-9.04	-3.21	-0.61	0
	mean concentration per fraction $\bar{c}_{j,k}$										0.53	0.67	0.84	1.97				C	U	A	G		C	U	A	G
	C	0.723	1.252	1.722	3.291	9.53	16.51	22.69	43.38	23.03	0.41	0.72	0.99	1.88	-10.8	6.35	C	0	-11.55	-16	-14.03	C	0	6.74	9.91	9.34
	U	0.961	1.552	2.081	3.628	10.94	17.66	23.69	41.29	23.4	0.47	0.75	1.01	1.76	0.84	-0.36	U	13.05	0	-5.04	-2.81	U	-6.31	0	2.98	2.44
2	A	1.197	1.877	2.484	4.167	11.43	17.92	23.71	39.78	23.21	0.49	0.77	1.02	1.71	6.2	-3.24	A	19.05	5.31	0	2.35	A	-9.02	-2.89	0	-0.52
	G	1.574	2.551	3.324	5.636	11.9	19.28	25.13	42.61	24.73	0.48	0.78	1.02	1.72	3.76	-2.74	G	16.32	2.89	-2.29	0	G	-8.54	-2.38	0.52	0
	mean concentration per fraction $\bar{c}_{j,k}$										0.46	0.76	1.01	1.77				C	U	A	G		C	U	A	G
	C	1.101	1.270	1.767	2.004	14.51	16.73	23.29	26.42	20.24	0.72	0.83	1.15	1.31	-1.91	3.04	C	0	-2.82	-4.24	-0.47	C	0	3.38	4.93	4
	U	1.387	1.586	2.174	2.374	15.78	18.05	24.74	27.02	21.4	0.74	0.84	1.16	1.26	0.93	-0.33	U	2.9	0	-1.46	2.42	U	-3.27	0	1.49	0.6
3	A	1.686	1.912	2.608	2.801	16.09	18.25	24.9	26.74	21.49	0.75	0.85	1.16	1.24	2.43	-1.8	A	4.43	1.48	0	3.94	A	-4.7	-1.47	0	-0.88
	G	2.224	2.592	3.658	3.876	16.81	19.6	27.65	29.3	23.34	0.72	0.84	1.18	1.26	-1.45	-0.92	G	0.47	-2.36	-3.79	0	G	-3.85	-0.59	0.89	0
	mean concentration per fraction $\bar{c}_{j,k}$										0.73	0.84	1.16	1.27				C	U	A	G		C	U	A	G

Supplementary Table 66: *Raw data Supplementary Fig. 15*

	area (counts * min)			deviation from mean (%)		
	repeat 1	repeat 2	repeat 3	repeat 1	repeat 2	repeat 3
S	585228	589553	600901	-1.13	-0.40	1.52
E	511421	519610	523440	-1.30	0.28	1.02
T	608092	615182	623499	-1.22	-0.07	1.28
A	585935	590815	594969	-0.79	0.04	0.74
P	168516	169837	171658	-0.88	-0.10	0.97
C	121011	121676	123355	-0.82	-0.28	1.10
K	477430	486495	485441	-1.18	0.70	0.48
V	758800	768624	780076	-1.35	-0.07	1.42
I	773793	784323	789932	-1.14	0.21	0.93
L	740480	757193	760091	-1.61	0.61	1.00
F	600381	608858	612207	-1.11	0.28	0.83

Supplementary references

References 41, 42 and 49 are also referenced in the main text.

41. Keil, L., Hartmann, M., Lanzmich, S. & Braun, D. Probing of molecular replication and accumulation in shallow heat gradients through numerical simulations. *Phys. Chem. Chem. Phys.* **18**, 20153–20159 (2016).

42. Matreux, T. *et al.* Heat flows in rock cracks naturally optimize salt compositions for ribozymes. *Nat. Chem.* **13**, 1038–1045 (2021).

49. Bai, L., Baker, D. R. & Hill, R. J. Permeability of vesicular Stromboli basaltic glass: Lattice Boltzmann simulations and laboratory measurements. *J. Geophys. Res.* **115**, B07201 (2010).

55. Viswanathan, H. S. *et al.* From Fluid Flow to Coupled Processes in Fractured Rock: Recent Advances and New Frontiers. *Reviews of Geophysics* **60**, (2022).

Calibration data amino acids (Fig. 2)

Fluorescence channel for all amino acids except for tryptophan and tyrosine, where the UV-channel is used (see Methods for details).

Calibration

Calibration Details	Histidine			
Calibration Type	Lin		*Offset (C0)*	0.0000
Evaluation Type	Area		*Slope (C1)*	3299.4061
Number of Calibration Points	18		*Curve (C2)*	0.0000
Number of disabled Calibration Points	0		*R-Square*	0.9905

Calibration Plot Histidine

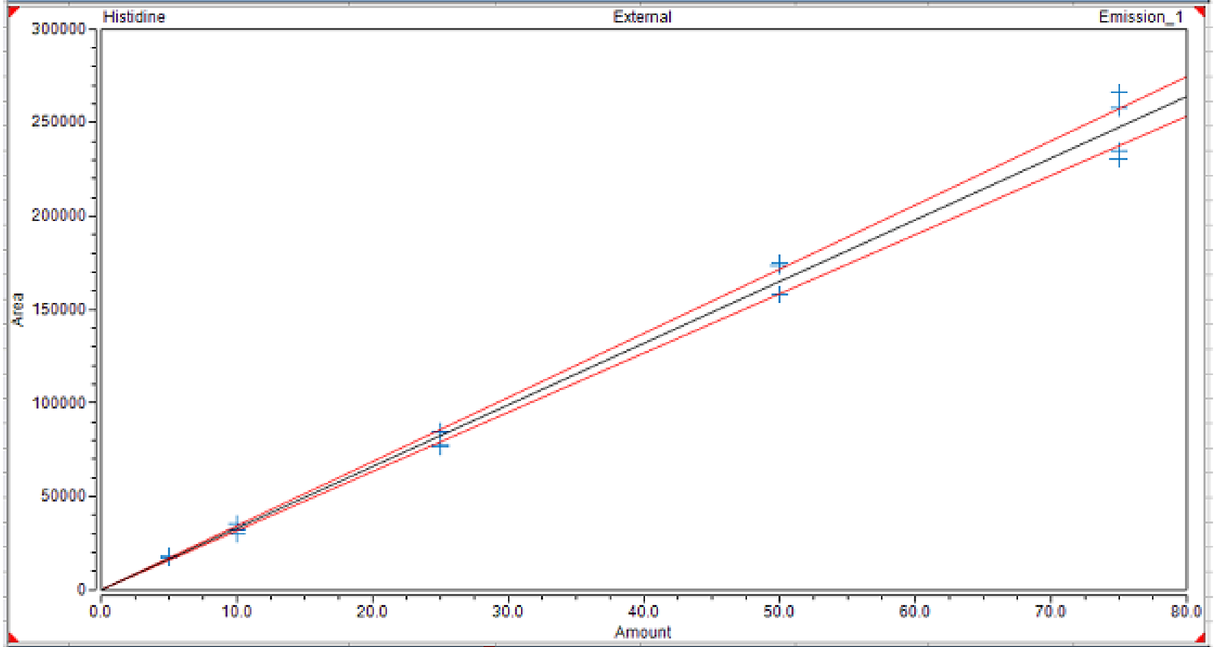

Calibration Results Histidine

No.	Injection Name	Calibration Level	X Value Emission_1 Histidine	Y Value Emission_1 Histidine	Y Value Emission_1 Histidine	Area counts*min Emission_1 Histidine	Height counts Emission_1 Histidine
1	5uM	1	5.0000	17149.2988	17149.2988	17149.299	430658.892
2	10uM	2	10.0000	29464.4402	29464.4402	29464.440	788256.385
3	10uM	2	10.0000	31875.9191	31875.9191	31875.919	837970.702
4	25uM	3	25.0000	77952.7894	77952.7894	77952.789	2104023.616
5	25uM	3	25.0000	76497.9025	76497.9025	76497.903	2045344.949
6	50uM	4	50.0000	158357.9651	158357.9651	158357.965	4324671.565
7	50uM	4	50.0000	157788.6897	157788.6897	157788.690	4223094.251
8	75uM	5	75.0000	230633.3556	230633.3556	230633.356	6301319.471
9	75uM	5	75.0000	234568.4690	234568.4690	234568.469	6263984.138
10	5uM	1	5.0000	18135.3581	18135.3581	18135.358	468986.120
11	10uM	2	10.0000	32753.8240	32753.8240	32753.824	896269.025
12	10uM	2	10.0000	35001.9738	35001.9738	35001.974	930218.238
13	25uM	3	25.0000	84781.3231	84781.3231	84781.323	2370673.924
14	25uM	3	25.0000	84392.0926	84392.0926	84392.093	2333925.080
15	50uM	4	50.0000	174672.5049	174672.5049	174672.505	4840254.790
16	50uM	4	50.0000	173453.5585	173453.5585	173453.559	4774156.498
17	75uM	5	75.0000	258060.8098	258060.8098	258060.810	7118714.942
18	75uM	5	75.0000	265968.3396	265968.3396	265968.340	7309306.574

Calibration

Calibration Details	Asparagine			
Calibration Type	Lin		*Offset (C0)*	0.0000
Evaluation Type	Area		*Slope (C1)*	5075.6814
Number of Calibration Points	18		*Curve (C2)*	0.0000
Number of disabled Calibration Points	0		*R-Square*	0.9880

Calibration Plot **Asparagine**

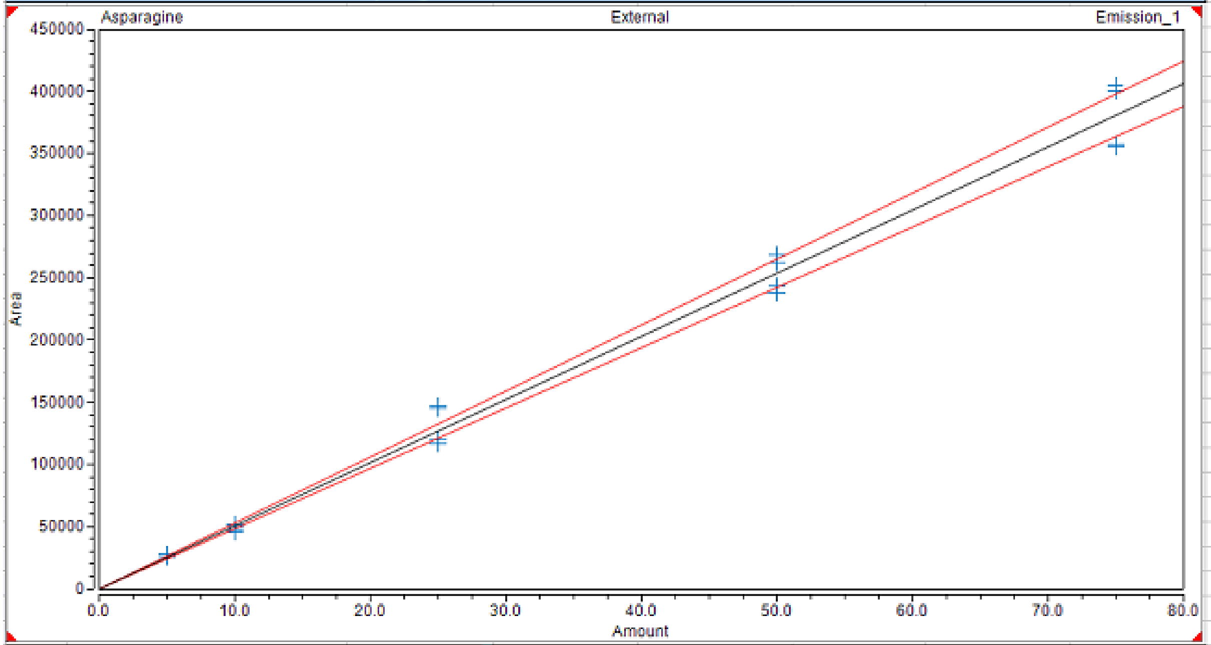

Calibration Results **Asparagine**

No.	Injection Name	Calibration Level	X Value<br><br>Emission_1<br>Asparagine	Y Value<br><br>Emission_1<br>Asparagine	Y Value<br><br>Emission_1<br>Asparagine	Area<br>counts*min<br>Emission_1<br>Asparagine	Height<br>counts<br>Emission_1<br>Asparagine
1	5uM	1	5.0000	25762.2891	25762.2891	25762.289	641442.650
2	10uM	2	10.0000	45954.3632	45954.3632	45954.363	1185387.769
3	10uM	2	10.0000	47572.5953	47572.5953	47572.595	1228981.681
4	25uM	3	25.0000	120201.2486	120201.2486	120201.249	3155436.568
5	25uM	3	25.0000	117003.6724	117003.6724	117003.672	3028569.594
6	50uM	4	50.0000	238203.7590	238203.7590	238203.759	6166540.513
7	50uM	4	50.0000	243481.6461	243481.6461	243481.646	6331679.837
8	75uM	5	75.0000	355416.1261	355416.1261	355416.126	9160881.995
9	75uM	5	75.0000	357369.5200	357369.5200	357369.520	9054709.212
10	5uM	1	5.0000	27458.7923	27458.7923	27458.792	709697.161
11	10uM	2	10.0000	49709.5324	49709.5324	49709.532	1309930.103
12	10uM	2	10.0000	51627.1407	51627.1407	51627.141	1389434.632
13	25uM	3	25.0000	146893.8582	146893.8582	146893.858	3487863.489
14	25uM	3	25.0000	144939.2143	144939.2143	144939.214	3428339.373
15	50uM	4	50.0000	262134.7327	262134.7327	262134.733	7054946.346
16	50uM	4	50.0000	268604.7874	268604.7874	268604.787	7126169.717
17	75uM	5	75.0000	400454.8912	400454.8912	400454.891	10577787.414
18	75uM	5	75.0000	405039.0288	405039.0288	405039.029	10707786.281

Calibration

Calibration Details	Serine			
Calibration Type	Lin		*Offset (C0)*	0.0000
Evaluation Type	Area		*Slope (C1)*	4926.4059
Number of Calibration Points	18		*Curve (C2)*	0.0000
Number of disabled Calibration Points	0		*R-Square*	0.9895

Calibration Plot **Serine**

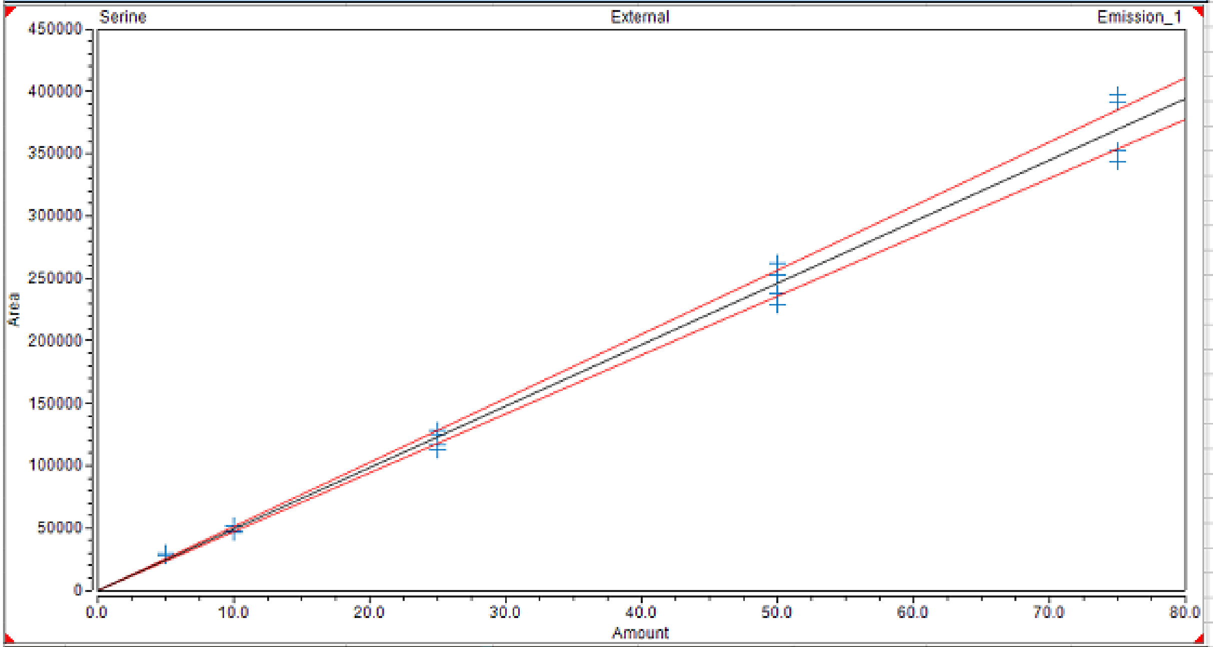

Calibration Results **Serine**

No.	Injection Name	Calibration Level	X Value<br><br>Emission_1<br>Serine	Y Value<br><br>Emission_1<br>Serine	Y Value<br><br>Emission_1<br>Serine	Area<br>counts*min<br>Emission_1<br>Serine	Height<br>counts<br>Emission_1<br>Serine
1	5uM	1	5.0000	27417.4351	27417.4351	27417.435	662566.444
2	10uM	2	10.0000	47616.9767	47616.9767	47616.977	1145061.727
3	10uM	2	10.0000	46916.6617	46916.6617	46916.662	1157511.057
4	25uM	3	25.0000	116434.4453	116434.4453	116434.445	2849513.951
5	25uM	3	25.0000	112175.2418	112175.2418	112175.242	2792481.863
6	50uM	4	50.0000	228387.5648	228387.5648	228387.565	5571502.027
7	50uM	4	50.0000	237795.9300	237795.9300	237795.930	5838791.692
8	75uM	5	75.0000	352061.1027	352061.1027	352061.103	8636249.000
9	75uM	5	75.0000	343940.6489	343940.6489	343940.649	8416270.339
10	5uM	1	5.0000	30053.7546	30053.7546	30053.755	731130.892
11	10uM	2	10.0000	51379.0783	51379.0783	51379.078	1272849.946
12	10uM	2	10.0000	51801.4751	51801.4751	51801.475	1280076.432
13	25uM	3	25.0000	127964.2238	127964.2238	127964.224	3157439.762
14	25uM	3	25.0000	125397.4698	125397.4698	125397.470	3091349.853
15	50uM	4	50.0000	252733.3695	252733.3695	252733.369	6296144.203
16	50uM	4	50.0000	261960.9359	261960.9359	261960.936	6458904.513
17	75uM	5	75.0000	397125.7800	397125.7800	397125.780	9819279.471
18	75uM	5	75.0000	390651.3788	390651.3788	390651.379	9698667.743

Calibration

Calibration Details	Glutamine			
Calibration Type	Lin		*Offset (C0)*	0.0000
Evaluation Type	Area		*Slope (C1)*	4726.9730
Number of Calibration Points	18		*Curve (C2)*	0.0000
Number of disabled Calibration Points	0		*R-Square*	0.9899

Calibration Plot **Glutamine**

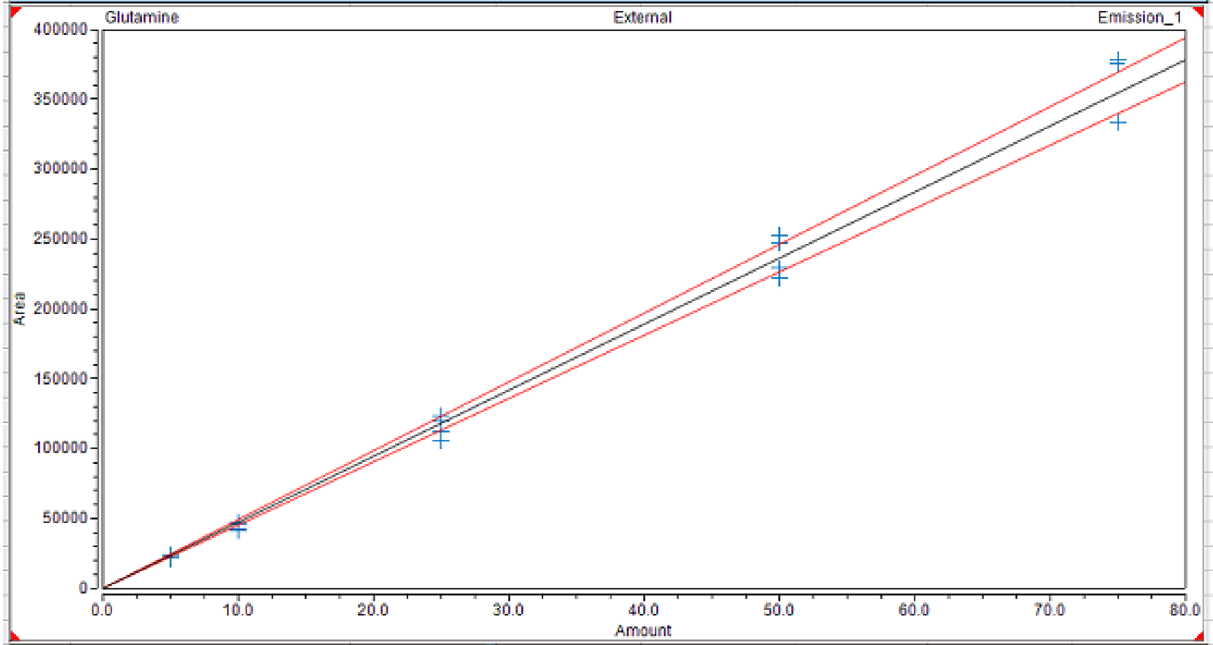

Calibration Results **Glutamine**

No.	Injection Name	Calibration Level	X Value<br><br>Emission_1<br>Glutamine	Y Value<br><br>Emission_1<br>Glutamine	Y Value<br><br>Emission_1<br>Glutamine	Area<br>counts*min<br>Emission_1<br>Glutamine	Height<br>counts<br>Emission_1<br>Glutamine
1	5uM	1	5.0000	21477.8419	21477.8419	21477.842	532478.205
2	10uM	2	10.0000	41773.5583	41773.5583	41773.558	1025529.273
3	10uM	2	10.0000	42389.2969	42389.2969	42389.297	1052775.233
4	25uM	3	25.0000	112310.5195	112310.5195	112310.520	2724048.798
5	25uM	3	25.0000	105972.1685	105972.1685	105972.168	2644465.707
6	50uM	4	50.0000	221985.1735	221985.1735	221985.174	5393799.013
7	50uM	4	50.0000	228906.9276	228906.9276	228906.928	5537778.105
8	75uM	5	75.0000	333535.7774	333535.7774	333535.777	8206516.582
9	75uM	5	75.0000	333377.1280	333377.1280	333377.128	8051091.554
10	5uM	1	5.0000	23937.7436	23937.7436	23937.744	596136.581
11	10uM	2	10.0000	45946.9984	45946.9984	45946.998	1149424.707
12	10uM	2	10.0000	47012.5231	47012.5231	47012.523	1163389.570
13	25uM	3	25.0000	123338.2113	123338.2113	123338.211	3064441.666
14	25uM	3	25.0000	119934.3002	119934.3002	119934.300	2942234.216
15	50uM	4	50.0000	246611.1784	246611.1784	246611.178	6079037.020
16	50uM	4	50.0000	252988.2700	252988.2700	252988.270	6185574.765
17	75uM	5	75.0000	374951.7286	374951.7286	374951.729	9290024.087
18	75uM	5	75.0000	378261.1552	378261.1552	378261.155	9347791.438

Calibration

Calibration Details	Arginine			
Calibration Type	Lin		*Offset (C0)*	0.0000
Evaluation Type	Area		*Slope (C1)*	3134.4265
Number of Calibration Points	18		*Curve (C2)*	0.0000
Number of disabled Calibration Points	0		*R-Square*	0.9905

Calibration Plot **Arginine**

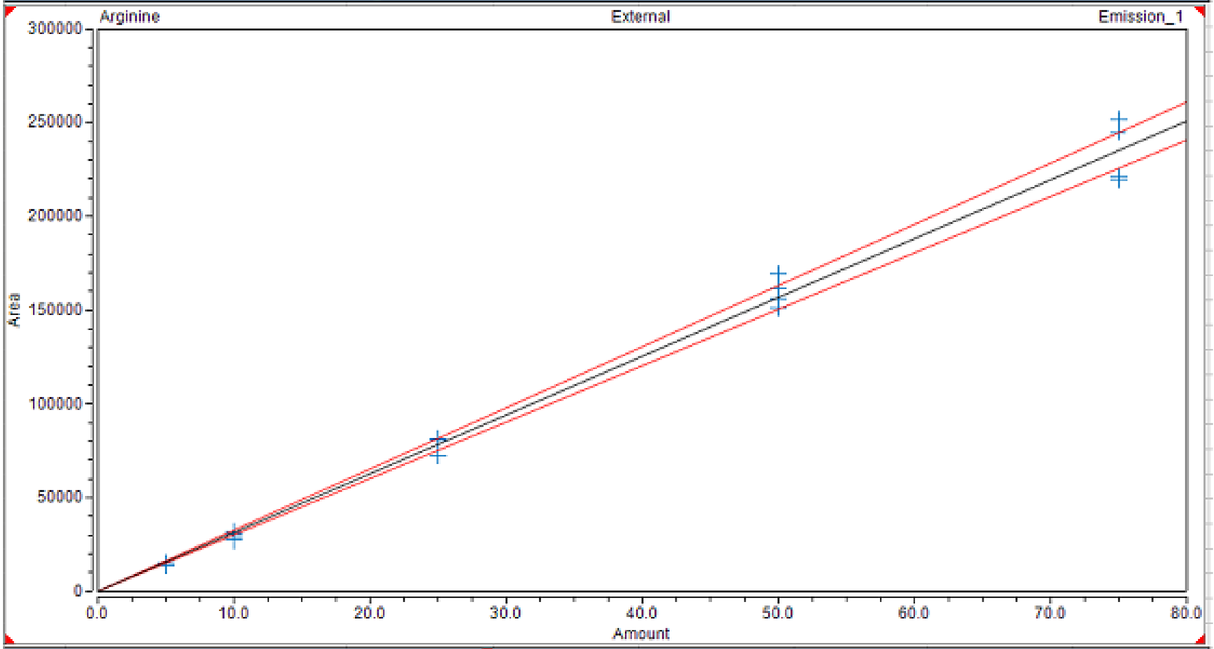

Calibration Results **Arginine**

No.	Injection Name	Calibration Level	X Value<br><br>Emission_1<br>Arginine	Y Value<br><br>Emission_1<br>Arginine	Y Value<br><br>Emission_1<br>Arginine	Area<br>counts*min<br>Emission_1<br>Arginine	Height<br>counts<br>Emission_1<br>Arginine
1	5uM	1	5.0000	13771.4041	13771.4041	13771.404	339971.091
2	10uM	2	10.0000	27322.7586	27322.7586	27322.759	650665.108
3	10uM	2	10.0000	29320.3371	29320.3371	29320.337	684238.419
4	25uM	3	25.0000	72361.7482	72361.7482	72361.748	1751333.683
5	25uM	3	25.0000	72017.2018	72017.2018	72017.202	1729384.273
6	50uM	4	50.0000	155914.1662	155914.1662	155914.166	3656195.038
7	50uM	4	50.0000	150867.7804	150867.7804	150867.780	3602518.773
8	75uM	5	75.0000	219444.5239	219444.5239	219444.524	5294251.189
9	75uM	5	75.0000	220708.6935	220708.6935	220708.693	5272280.536
10	5uM	1	5.0000	14925.6205	14925.6205	14925.621	367929.422
11	10uM	2	10.0000	30117.7691	30117.7691	30117.769	706680.954
12	10uM	2	10.0000	31765.6071	31765.6071	31765.607	766911.416
13	25uM	3	25.0000	81555.3230	81555.3230	81555.323	1925368.711
14	25uM	3	25.0000	80924.7733	80924.7733	80924.773	1917851.946
15	50uM	4	50.0000	169073.1977	169073.1977	169073.198	4062712.203
16	50uM	4	50.0000	161350.2043	161350.2043	161350.204	3990073.630
17	75uM	5	75.0000	244893.8660	244893.8660	244893.866	5927414.634
18	75uM	5	75.0000	251685.2446	251685.2446	251685.245	6068835.275

Calibration

Calibration Details	Glycine		
Calibration Type	Lin	*Offset (C0)*	0.0000
Evaluation Type	Area	*Slope (C1)*	5555.0666
Number of Calibration Points	18	*Curve (C2)*	0.0000
Number of disabled Calibration Points	0	*R-Square*	0.9886

Calibration Plot **Glycine**

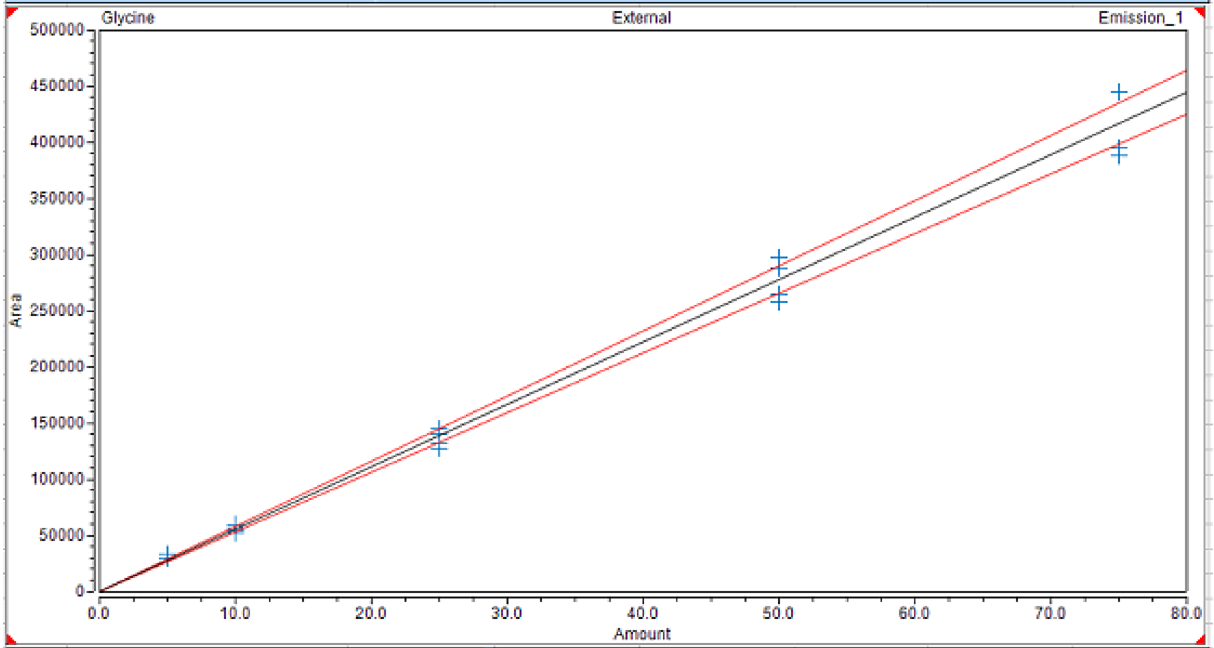

Calibration Results **Glycine**

No.	Injection Name	Calibration Level	X Value Emission_1 Glycine	Y Value Emission_1 Glycine	Y Value Emission_1 Glycine	Area counts*min Emission_1 Glycine	Height counts Emission_1 Glycine
1	5uM	1	5.0000	30186.5560	30186.5560	30186.556	649393.753
2	10uM	2	10.0000	51791.0517	51791.0517	51791.052	1131570.307
3	10uM	2	10.0000	54021.5994	54021.5994	54021.599	1152095.903
4	25uM	3	25.0000	132563.5050	132563.5050	132563.505	2886673.528
5	25uM	3	25.0000	127179.0845	127179.0845	127179.085	2767070.951
6	50uM	4	50.0000	257747.2491	257747.2491	257747.249	5675142.666
7	50uM	4	50.0000	264140.5258	264140.5258	264140.526	5832264.688
8	75uM	5	75.0000	394532.6580	394532.6580	394532.658	8684638.975
9	75uM	5	75.0000	387794.8855	387794.8855	387794.885	8473400.398
10	5uM	1	5.0000	32937.9049	32937.9049	32937.905	702161.245
11	10uM	2	10.0000	56102.7189	56102.7189	56102.719	1225057.800
12	10uM	2	10.0000	59348.9235	59348.9235	59348.923	1250629.350
13	25uM	3	25.0000	144558.2555	144558.2555	144558.256	3167493.256
14	25uM	3	25.0000	140680.7768	140680.7768	140680.777	3095069.594
15	50uM	4	50.0000	288339.5580	288339.5580	288339.558	6299793.771
16	50uM	4	50.0000	297443.7339	297443.7339	297443.734	6482479.389
17	75uM	5	75.0000	445180.9947	445180.9947	445180.995	9830685.703
18	75uM	5	75.0000	444368.1317	444368.1317	444368.132	9699299.832

Calibration

Calibration Details	Aspartic acid		
Calibration Type	Lin	*Offset (C0)*	0.0000
Evaluation Type	Area	*Slope (C1)*	4625.3724
Number of Calibration Points	18	*Curve (C2)*	0.0000
Number of disabled Calibration Points	0	*R-Square*	0.9731

Calibration Plot **Aspartic acid**

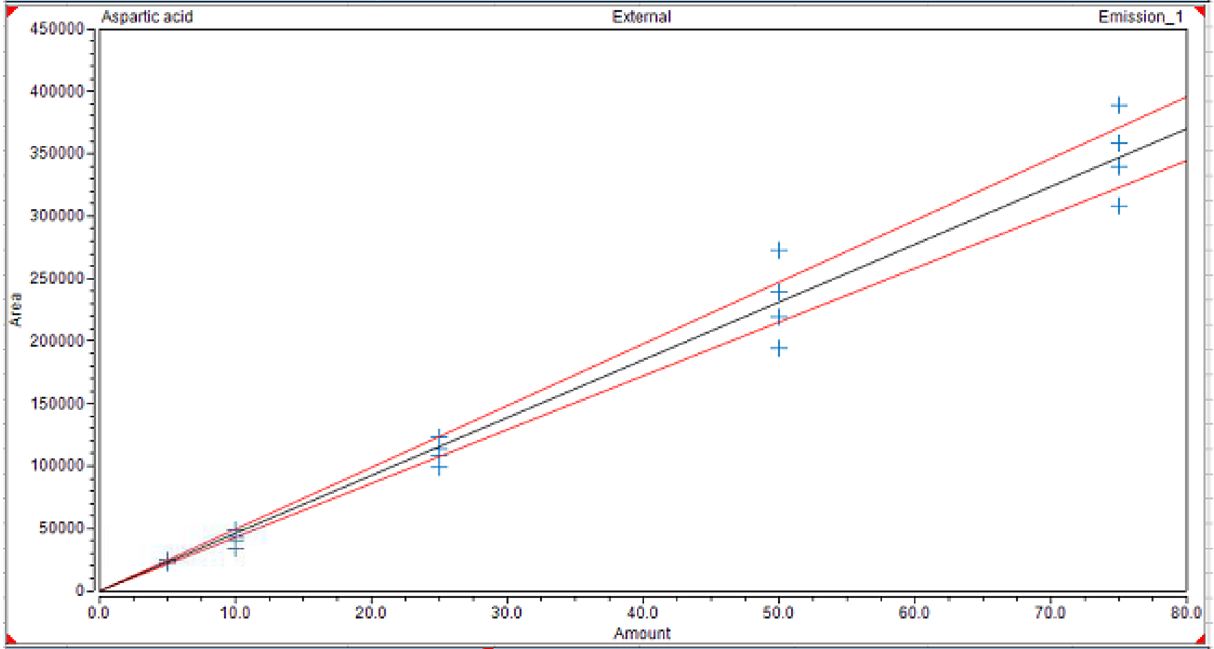

Calibration Results **Aspartic acid**

No.	Injection Name	Calibration Level	X Value<br><br>Emission_1<br>Aspartic acid	Y Value<br><br>Emission_1<br>Aspartic acid	Y Value<br><br>Emission_1<br>Aspartic acid	Area<br>counts*min<br>Emission_1<br>Aspartic acid	Height<br>counts<br>Emission_1<br>Aspartic acid
1	5uM	1	5.0000	22860.2950	22860.2950	22860.295	509271.691
2	10uM	2	10.0000	43028.9427	43028.9427	43028.943	942025.186
3	10uM	2	10.0000	34577.0927	34577.0927	34577.093	756492.869
4	25uM	3	25.0000	108406.5575	108406.5575	108406.557	2335110.521
5	25uM	3	25.0000	99444.9147	99444.9147	99444.915	2103952.088
6	50uM	4	50.0000	194300.6173	194300.6173	194300.617	4086469.652
7	50uM	4	50.0000	239157.9551	239157.9551	239157.955	5084295.169
8	75uM	5	75.0000	339319.2390	339319.2390	339319.239	7090759.239
9	75uM	5	75.0000	308235.3894	308235.3894	308235.389	6457870.308
10	5uM	1	5.0000	24839.5425	24839.5425	24839.543	560246.455
11	10uM	2	10.0000	48298.1528	48298.1528	48298.153	1030676.794
12	10uM	2	10.0000	40000.5331	40000.5331	40000.533	846877.935
13	25uM	3	25.0000	123073.8225	123073.8225	123073.822	2600507.300
14	25uM	3	25.0000	114084.5232	114084.5232	114084.523	2390116.331
15	50uM	4	50.0000	219595.5001	219595.5001	219595.500	4520206.589
16	50uM	4	50.0000	272651.3743	272651.3743	272651.374	5710213.015
17	75uM	5	75.0000	389134.6149	389134.6149	389134.615	8161976.894
18	75uM	5	75.0000	358795.9201	358795.9201	358795.920	7477551.395

Calibration

Calibration Details	Glutamic acid			
Calibration Type	Lin		*Offset (C0)*	0.0000
Evaluation Type	Area		*Slope (C1)*	4334.8757
Number of Calibration Points	18		*Curve (C2)*	0.0000
Number of disabled Calibration Points	0		*R-Square*	0.9817

Calibration Plot Glutamic acid

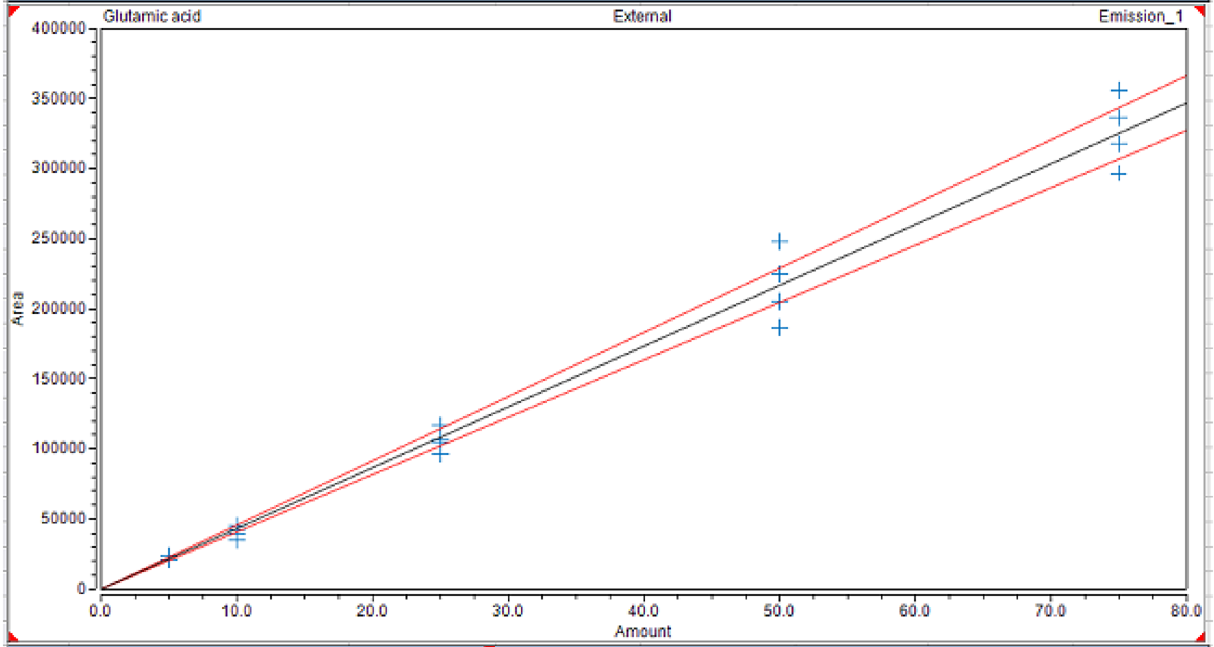

Calibration Results Glutamic acid

No.	Injection Name	Calibration Level	X Value<br><br>Emission_1 Glutamic acid	Y Value<br><br>Emission_1 Glutamic acid	Y Value<br><br>Emission_1 Glutamic acid	Area counts*min Emission_1 Glutamic acid	Height counts Emission_1 Glutamic acid
1	5uM	1	5.0000	21345.5310	21345.5310	21345.531	425848.844
2	10uM	2	10.0000	41940.6585	41940.6585	41940.659	840670.795
3	10uM	2	10.0000	35105.7740	35105.7740	35105.774	701644.676
4	25uM	3	25.0000	104990.7003	104990.7003	104990.700	2087257.329
5	25uM	3	25.0000	96375.7267	96375.7267	96375.727	1908361.368
6	50uM	4	50.0000	187002.8144	187002.8144	187002.814	3703203.364
7	50uM	4	50.0000	224776.3107	224776.3107	224776.311	4474950.508
8	75uM	5	75.0000	317847.0809	317847.0809	317847.081	6331361.438
9	75uM	5	75.0000	296762.1877	296762.1877	296762.188	5883059.567
10	5uM	1	5.0000	23805.6568	23805.6568	23805.657	480939.238
11	10uM	2	10.0000	45572.2633	45572.2633	45572.263	901152.587
12	10uM	2	10.0000	39285.2625	39285.2625	39285.263	771209.348
13	25uM	3	25.0000	116743.6065	116743.6065	116743.606	2292480.891
14	25uM	3	25.0000	106482.5056	106482.5056	106482.506	2091584.057
15	50uM	4	50.0000	204933.8821	204933.8821	204933.882	4021192.622
16	50uM	4	50.0000	247758.7909	247758.7909	247758.791	4914502.334
17	75uM	5	75.0000	355565.1034	355565.1034	355565.103	7037582.956
18	75uM	5	75.0000	336334.2417	336334.2417	336334.242	6641527.153

Calibration

Calibration Details	Threonine		
Calibration Type	Lin	*Offset (C0)*	0.0000
Evaluation Type	Area	*Slope (C1)*	5476.0653
Number of Calibration Points	18	*Curve (C2)*	0.0000
Number of disabled Calibration Points	0	*R-Square*	0.9906

Calibration Plot **Threonine**

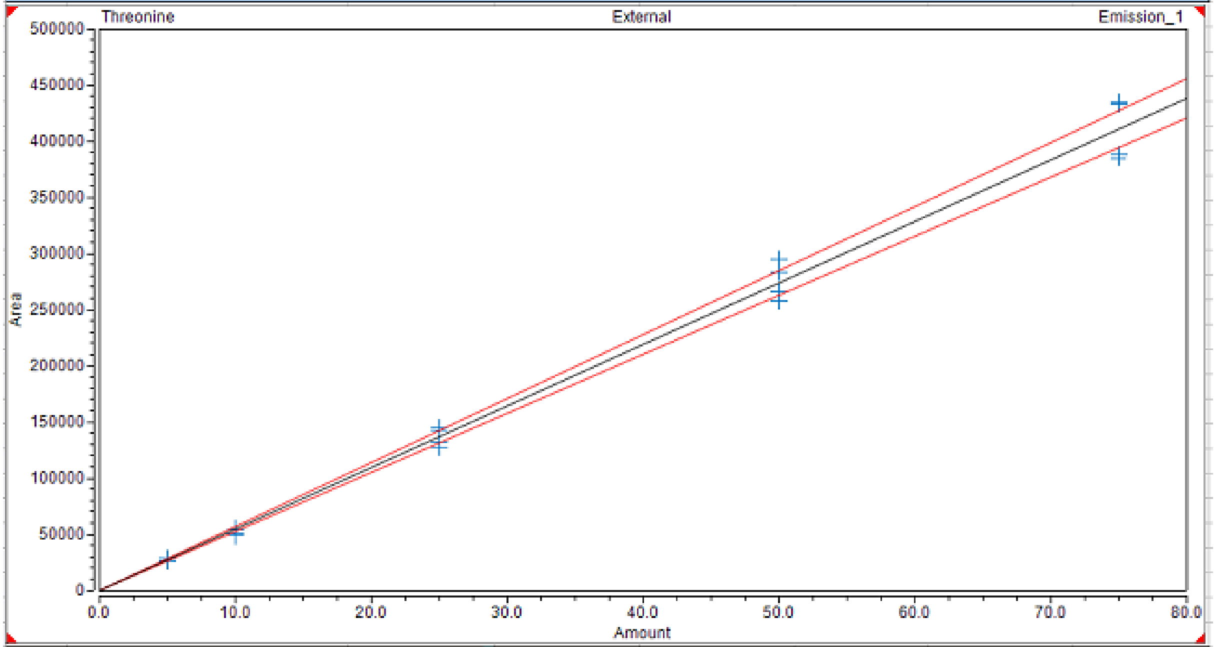

Calibration Results **Threonine**

No.	Injection Name	Calibration Level	X Value<br><br>Emission_1<br>Threonine	Y Value<br><br>Emission_1<br>Threonine	Y Value<br><br>Emission_1<br>Threonine	Area<br>counts*min<br>Emission_1<br>Threonine	Height<br>counts<br>Emission_1<br>Threonine
1	5uM	1	5.0000	25854.4364	25854.4364	25854.436	490518.424
2	10uM	2	10.0000	49041.1019	49041.1019	49041.102	938160.788
3	10uM	2	10.0000	50342.9024	50342.9024	50342.902	945580.472
4	25uM	3	25.0000	131676.1518	131676.1518	131676.152	2440868.896
5	25uM	3	25.0000	127201.2388	127201.2388	127201.239	2331828.695
6	50uM	4	50.0000	257165.2701	257165.2701	257165.270	4772436.161
7	50uM	4	50.0000	265333.9014	265333.9014	265333.901	4972327.798
8	75uM	5	75.0000	387783.3365	387783.3365	387783.336	7190740.409
9	75uM	5	75.0000	384887.5413	384887.5413	384887.541	7163603.550
10	5uM	1	5.0000	28304.5409	28304.5409	28304.541	538128.133
11	10uM	2	10.0000	54410.4664	54410.4664	54410.466	1021168.974
12	10uM	2	10.0000	54703.3390	54703.3390	54703.339	1028800.757
13	25uM	3	25.0000	145240.9607	145240.9607	145240.961	2665264.639
14	25uM	3	25.0000	142158.4315	142158.4315	142158.432	2589142.201
15	50uM	4	50.0000	283779.5221	283779.5221	283779.522	5242762.125
16	50uM	4	50.0000	294927.8015	294927.8015	294927.801	5443584.109
17	75uM	5	75.0000	434635.0029	434635.0029	434635.003	8012024.178
18	75uM	5	75.0000	433407.4139	433407.4139	433407.414	7991785.909

Calibration

Calibration Details	Alanine			
Calibration Type	Lin		*Offset (C0)*	0.0000
Evaluation Type	Area		*Slope (C1)*	4752.8095
Number of Calibration Points	18		*Curve (C2)*	0.0000
Number of disabled Calibration Points	0		*R-Square*	0.9840

Calibration Plot **Alanine**

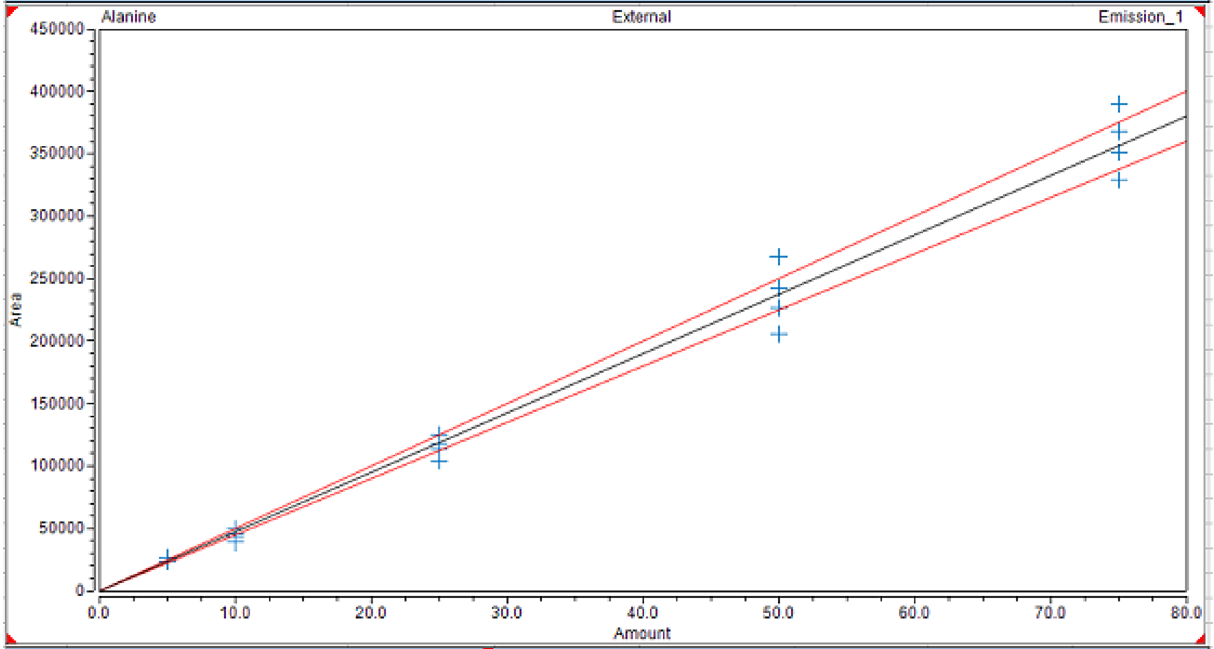

Calibration Results **Alanine**

No.	Injection Name	Calibration Level	X Value Emission_1 Alanine	Y Value Emission_1 Alanine	Y Value Emission_1 Alanine	Area counts*min Emission_1 Alanine	Height counts Emission_1 Alanine
1	5uM	1	5.0000	23552.7463	23552.7463	23552.746	403857.593
2	10uM	2	10.0000	45902.6890	45902.6890	45902.689	778783.906
3	10uM	2	10.0000	38863.4360	38863.4360	38863.436	671568.901
4	25uM	3	25.0000	113811.5433	113811.5433	113811.543	1938689.300
5	25uM	3	25.0000	104684.7293	104684.7293	104684.729	1772405.314
6	50uM	4	50.0000	205365.8416	205365.8416	205365.842	3488550.462
7	50uM	4	50.0000	242292.1438	242292.1438	242292.144	4048075.031
8	75uM	5	75.0000	351087.3456	351087.3456	351087.346	5901219.626
9	75uM	5	75.0000	328880.2561	328880.2561	328880.256	5559015.488
10	5uM	1	5.0000	26859.5903	26859.5903	26859.590	449012.383
11	10uM	2	10.0000	49668.9319	49668.9319	49668.932	835443.459
12	10uM	2	10.0000	43467.8764	43467.8764	43467.876	729160.324
13	25uM	3	25.0000	124913.4388	124913.4388	124913.439	2093564.979
14	25uM	3	25.0000	117441.1315	117441.1315	117441.131	1957492.708
15	50uM	4	50.0000	226220.9723	226220.9723	226220.972	3752545.014
16	50uM	4	50.0000	267500.8642	267500.8642	267500.864	4442354.543
17	75uM	5	75.0000	390254.8893	390254.8893	390254.889	6505670.661
18	75uM	5	75.0000	367987.4225	367987.4225	367987.423	6141096.317

Calibration

Calibration Details	Proline			
Calibration Type	Lin		*Offset (C0)*	0.0000
Evaluation Type	Area		*Slope (C1)*	1452.4449
Number of Calibration Points	18		*Curve (C2)*	0.0000
Number of disabled Calibration Points	0		*R-Square*	0.9892

Calibration Plot **Proline**

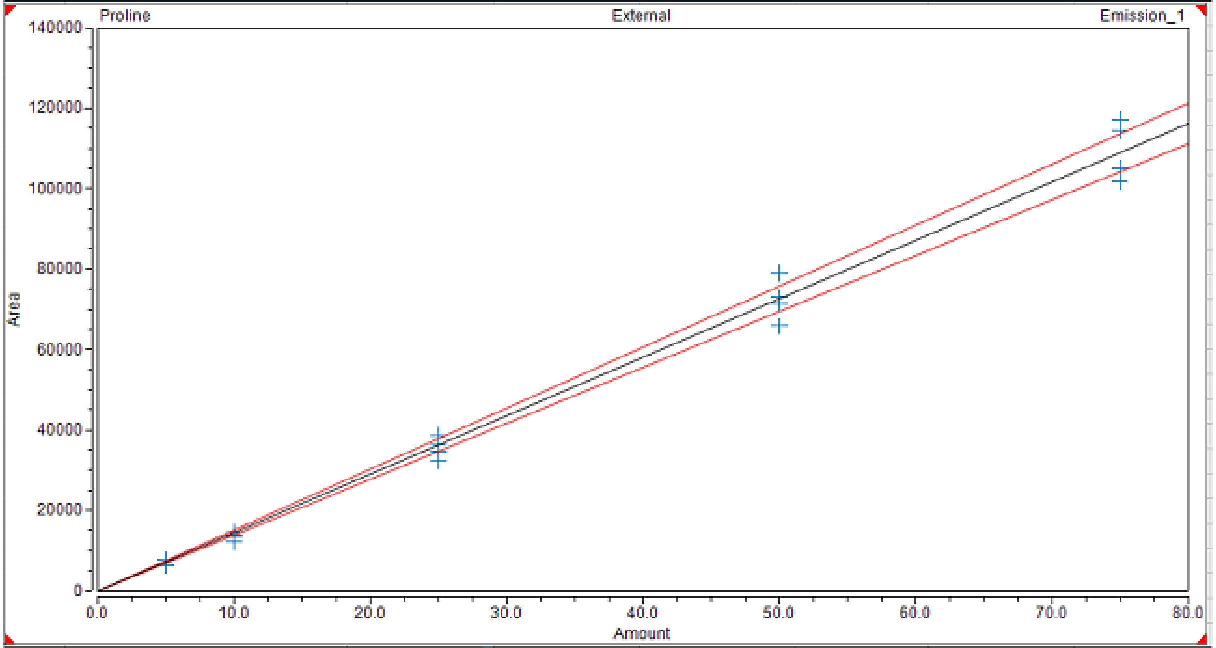

Calibration Results **Proline**

No.	Injection Name	Calibration Level	X Value<br><br>Emission_1 Proline	Y Value<br><br>Emission_1 Proline	Y Value<br><br>Emission_1 Proline	Area counts*min Emission_1 Proline	Height counts Emission_1 Proline
1	5uM	1	5.0000	6382.8285	6382.8285	6382.829	106026.328
2	10uM	2	10.0000	13622.0075	13622.0075	13622.008	212169.718
3	10uM	2	10.0000	12114.3152	12114.3152	12114.315	193487.207
4	25uM	3	25.0000	34612.3600	34612.3600	34612.360	536443.624
5	25uM	3	25.0000	32327.8353	32327.8353	32327.835	498072.002
6	50uM	4	50.0000	65798.8457	65798.8457	65798.846	1005963.498
7	50uM	4	50.0000	71596.4068	71596.4068	71596.407	1092256.079
8	75uM	5	75.0000	105149.9258	105149.9258	105149.926	1604543.430
9	75uM	5	75.0000	101611.4607	101611.4607	101611.461	1556146.818
10	5uM	1	5.0000	7671.5000	7671.5000	7671.500	119755.432
11	10uM	2	10.0000	14448.8987	14448.8987	14448.899	227690.233
12	10uM	2	10.0000	13776.3500	13776.3500	13776.350	213514.033
13	25uM	3	25.0000	38657.5741	38657.5741	38657.574	575889.745
14	25uM	3	25.0000	36448.3534	36448.3534	36448.353	544994.106
15	50uM	4	50.0000	72951.0697	72951.0697	72951.070	1097795.188
16	50uM	4	50.0000	79061.3791	79061.3791	79061.379	1196905.446
17	75uM	5	75.0000	117017.7342	117017.7342	117017.734	1764419.524
18	75uM	5	75.0000	114324.1618	114324.1618	114324.162	1754953.251

Calibration

Calibration Details	Cysteine			
Calibration Type	Lin		*Offset (C0)*	0.0000
Evaluation Type	Area		*Slope (C1)*	414.7629
Number of Calibration Points	18		*Curve (C2)*	0.0000
Number of disabled Calibration Points	0		*R-Square*	0.9843

Calibration Plot Cysteine

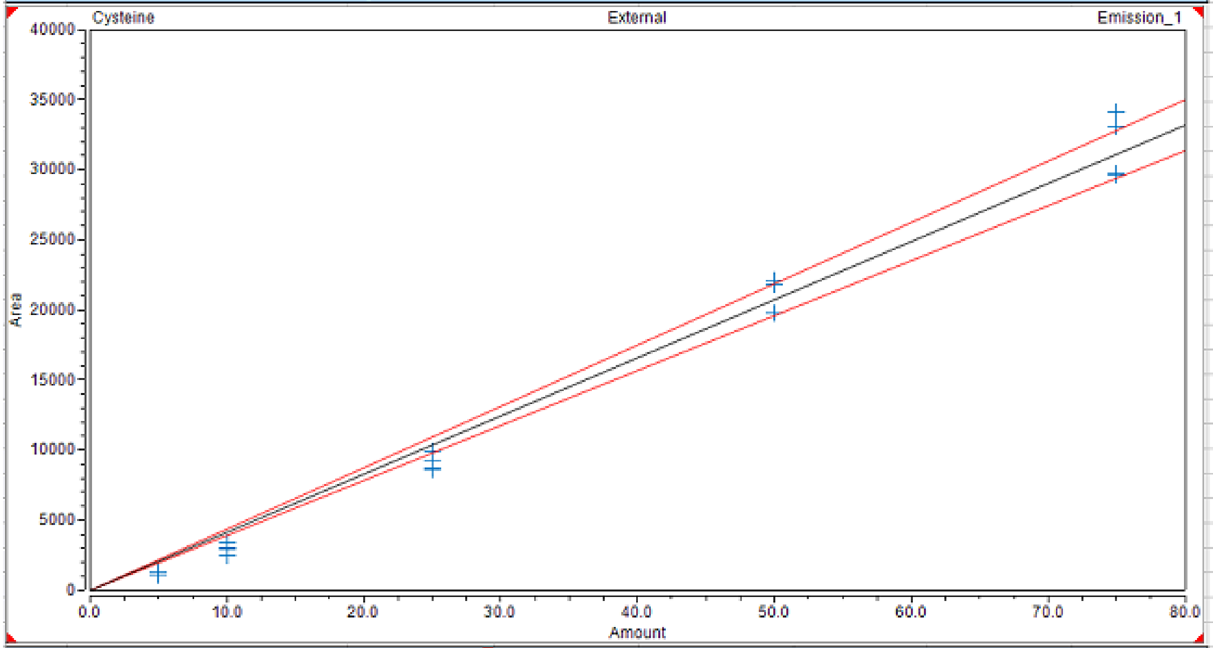

Calibration Results Cysteine

No.	Injection Name	Calibration Level	X Value<br><br>Emission_1 Cysteine	Y Value<br><br>Emission_1 Cysteine	Y Value<br><br>Emission_1 Cysteine	Area counts*min Emission_1 Cysteine	Height counts Emission_1 Cysteine
1	5uM	1	5.0000	1061.7898	1061.7898	1061.790	29690.915
2	10uM	2	10.0000	2446.7133	2446.7133	2446.713	66162.207
3	10uM	2	10.0000	2892.4442	2892.4442	2892.444	76582.298
4	25uM	3	25.0000	8638.4160	8638.4160	8638.416	212165.350
5	25uM	3	25.0000	8735.0820	8735.0820	8735.082	212477.324
6	50uM	4	50.0000	19783.9037	19783.9037	19783.904	492177.826
7	50uM	4	50.0000	19751.9238	19751.9238	19751.924	482459.710
8	75uM	5	75.0000	29611.7647	29611.7647	29611.765	729379.138
9	75uM	5	75.0000	29698.5815	29698.5815	29698.582	726856.550
10	5uM	1	5.0000	1289.8063	1289.8063	1289.806	36777.823
11	10uM	2	10.0000	3037.7095	3037.7095	3037.710	82815.669
12	10uM	2	10.0000	3382.6687	3382.6687	3382.669	85024.789
13	25uM	3	25.0000	9254.2378	9254.2378	9254.238	234231.158
14	25uM	3	25.0000	9940.4220	9940.4220	9940.422	248724.309
15	50uM	4	50.0000	22062.7980	22062.7980	22062.798	545630.061
16	50uM	4	50.0000	21817.5200	21817.5200	21817.520	537483.564
17	75uM	5	75.0000	33082.3268	33082.3268	33082.327	815033.809
18	75uM	5	75.0000	34127.0630	34127.0630	34127.063	835653.391

Calibration

Calibration Details	Lysine			
Calibration Type	Lin		*Offset (C0)*	0.0000
Evaluation Type	Area		*Slope (C1)*	3632.4287
Number of Calibration Points	18		*Curve (C2)*	0.0000
Number of disabled Calibration Points	0		*R-Square*	0.9797

Calibration Plot **Lysine**

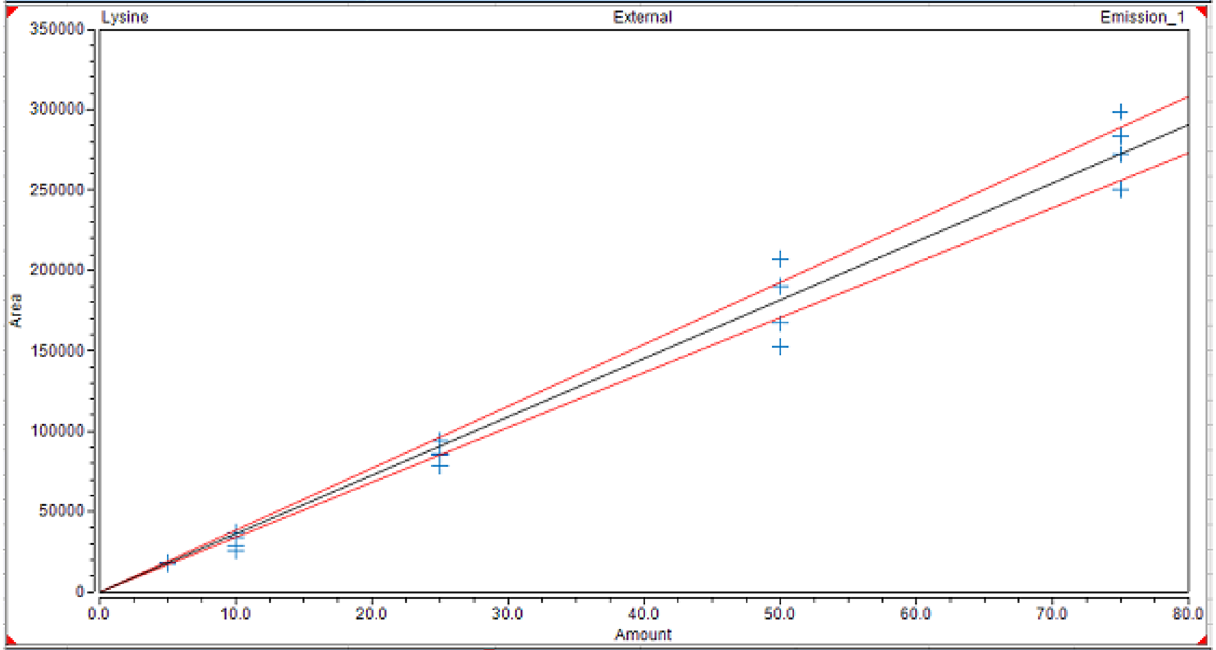

Calibration Results **Lysine**

No.	Injection Name	Calibration Level	X Value<br><br>Emission_1<br>Lysine	Y Value<br><br>Emission_1<br>Lysine	Y Value<br><br>Emission_1<br>Lysine	Area<br>counts*min<br>Emission_1<br>Lysine	Height<br>counts<br>Emission_1<br>Lysine
1	5uM	1	5.0000	17441.3499	17441.3499	17441.350	376002.800
2	10uM	2	10.0000	33524.3463	33524.3463	33524.346	745984.157
3	10uM	2	10.0000	25881.5792	25881.5792	25881.579	573391.668
4	25uM	3	25.0000	85199.9937	85199.9937	85199.994	1918905.971
5	25uM	3	25.0000	78440.5334	78440.5334	78440.533	1764268.543
6	50uM	4	50.0000	152791.6535	152791.6535	152791.654	3495323.075
7	50uM	4	50.0000	189959.3211	189959.3211	189959.321	4326111.027
8	75uM	5	75.0000	272081.9617	272081.9617	272081.962	6226551.462
9	75uM	5	75.0000	250671.2241	250671.2241	250671.224	5765972.320
10	5uM	1	5.0000	18552.6900	18552.6900	18552.690	405783.487
11	10uM	2	10.0000	36768.3285	36768.3285	36768.329	817772.730
12	10uM	2	10.0000	28974.6865	28974.6865	28974.686	651174.096
13	25uM	3	25.0000	94174.6727	94174.6727	94174.673	2107767.637
14	25uM	3	25.0000	85977.3390	85977.3390	85977.339	1938463.721
15	50uM	4	50.0000	166955.0650	166955.0650	166955.065	3849198.591
16	50uM	4	50.0000	207343.8636	207343.8636	207343.864	4732884.489
17	75uM	5	75.0000	298752.2228	298752.2228	298752.223	6825904.860
18	75uM	5	75.0000	283705.6442	283705.6442	283705.644	6537957.897

Calibration

Calibration Details	Methionine			
Calibration Type	Lin		*Offset (C0)*	0.0000
Evaluation Type	Area		*Slope (C1)*	1105.5433
Number of Calibration Points	18		*Curve (C2)*	0.0000
Number of disabled Calibration Points	0		*R-Square*	0.9929

Calibration Plot **Methionine**

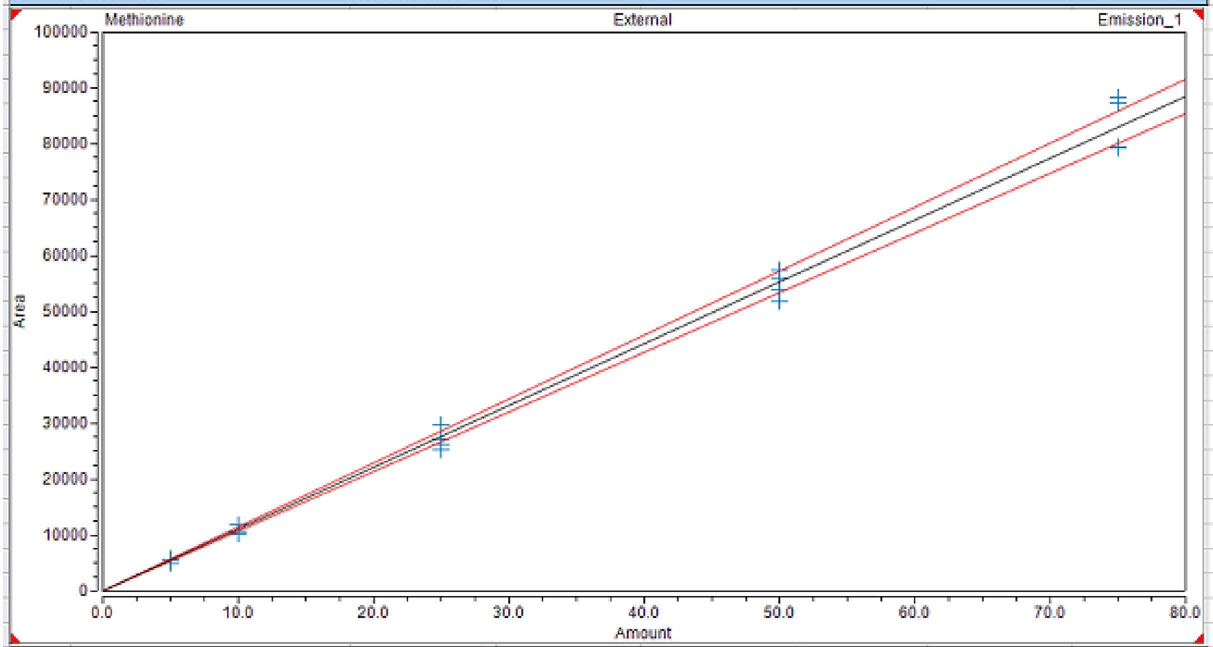

Calibration Results **Methionine**

No.	Injection Name	Calibration Level	X Value<br><br>Emission_1<br>Methionine	Y Value<br><br>Emission_1<br>Methionine	Y Value<br><br>Emission_1<br>Methionine	Area<br>counts*min<br>Emission_1<br>Methionine	Height<br>counts<br>Emission_1<br>Methionine
1	5uM	1	5.0000	5098.0456	5098.0456	5098.046	110019.971
2	10uM	2	10.0000	10511.6569	10511.6569	10511.657	201430.479
3	10uM	2	10.0000	10232.2403	10232.2403	10232.240	199323.351
4	25uM	3	25.0000	26251.9540	26251.9540	26251.954	512603.698
5	25uM	3	25.0000	25223.6479	25223.6479	25223.648	494846.933
6	50uM	4	50.0000	51690.8922	51690.8922	51690.892	1021201.939
7	50uM	4	50.0000	53901.4906	53901.4906	53901.491	1040198.984
8	75uM	5	75.0000	79415.9826	79415.9826	79415.983	1529069.915
9	75uM	5	75.0000	79220.8532	79220.8532	79220.853	1550990.692
10	5uM	1	5.0000	5586.3703	5586.3703	5586.370	117362.632
11	10uM	2	10.0000	10553.8852	10553.8852	10553.885	209674.174
12	10uM	2	10.0000	11788.3644	11788.3644	11788.364	222489.834
13	25uM	3	25.0000	29629.8008	29629.8008	29629.801	566360.137
14	25uM	3	25.0000	27187.5788	27187.5788	27187.579	537885.904
15	50uM	4	50.0000	55844.3828	55844.3828	55844.383	1078096.242
16	50uM	4	50.0000	57365.4061	57365.4061	57365.406	1113395.231
17	75uM	5	75.0000	87311.4506	87311.4506	87311.451	1655915.076
18	75uM	5	75.0000	88182.2796	88182.2796	88182.280	1717048.220

Calibration

Calibration Details	Valine			
Calibration Type	Lin		*Offset (C0)*	0.0000
Evaluation Type	Area		*Slope (C1)*	6528.6033
Number of Calibration Points	18		*Curve (C2)*	0.0000
Number of disabled Calibration Points	0		*R-Square*	0.9823

Calibration Plot **Valine**

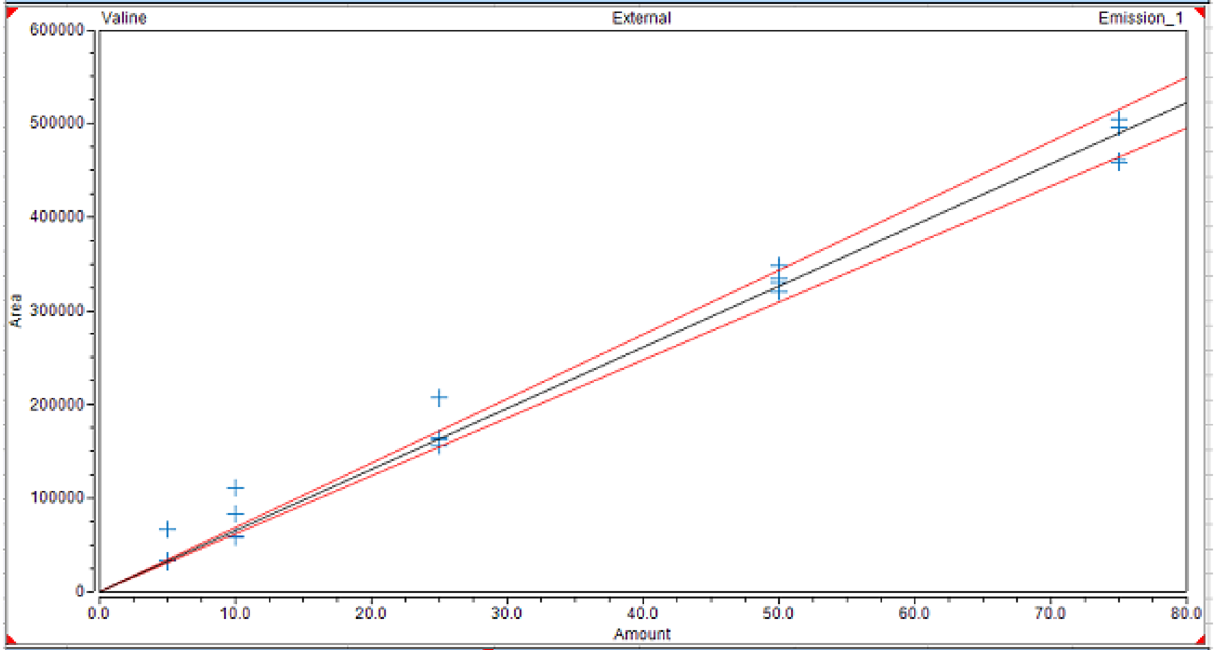

Calibration Results **Valine**

No.	Injection Name	Calibration Level	X Value Emission_1 Valine	Y Value Emission_1 Valine	Y Value Emission_1 Valine	Area counts*min Emission_1 Valine	Height counts Emission_1 Valine
1	5uM	1	5.0000	66269.9174	66269.9174	66269.917	1357845.371
2	10uM	2	10.0000	111169.6807	111169.6807	111169.681	2244080.227
3	10uM	2	10.0000	59194.3668	59194.3668	59194.367	1281581.664
4	25uM	3	25.0000	155606.1898	155606.1898	155606.190	3369708.171
5	25uM	3	25.0000	206658.3530	206658.3530	206658.353	4150363.777
6	50uM	4	50.0000	347730.7093	347730.7093	347730.709	7193131.871
7	50uM	4	50.0000	319820.5939	319820.5939	319820.594	6964954.385
8	75uM	5	75.0000	458284.5165	458284.5165	458284.516	9900749.710
9	75uM	5	75.0000	460638.8689	460638.8689	460638.869	9935189.562
10	5uM	1	5.0000	32250.9650	32250.9650	32250.965	695636.978
11	10uM	2	10.0000	83710.5384	83710.5384	83710.538	1759939.705
12	10uM	2	10.0000	58441.3643	58441.3643	58441.364	1248683.719
13	25uM	3	25.0000	163792.8088	163792.8088	163792.809	3517335.935
14	25uM	3	25.0000	162565.0984	162565.0984	162565.098	3455819.587
15	50uM	4	50.0000	330751.6918	330751.6918	330751.692	7024527.504
16	50uM	4	50.0000	334240.2451	334240.2451	334240.245	7161152.565
17	75uM	5	75.0000	496065.8883	496065.8883	496065.888	10613179.061
18	75uM	5	75.0000	504724.0784	504724.0784	504724.078	10818327.153

Calibration

Calibration Details	Isoleucine			
Calibration Type	Lin		*Offset (C0)*	0.0000
Evaluation Type	Area		*Slope (C1)*	6911.0469
Number of Calibration Points	18		*Curve (C2)*	0.0000
Number of disabled Calibration Points	0		*R-Square*	0.9894

Calibration Plot **Isoleucine**

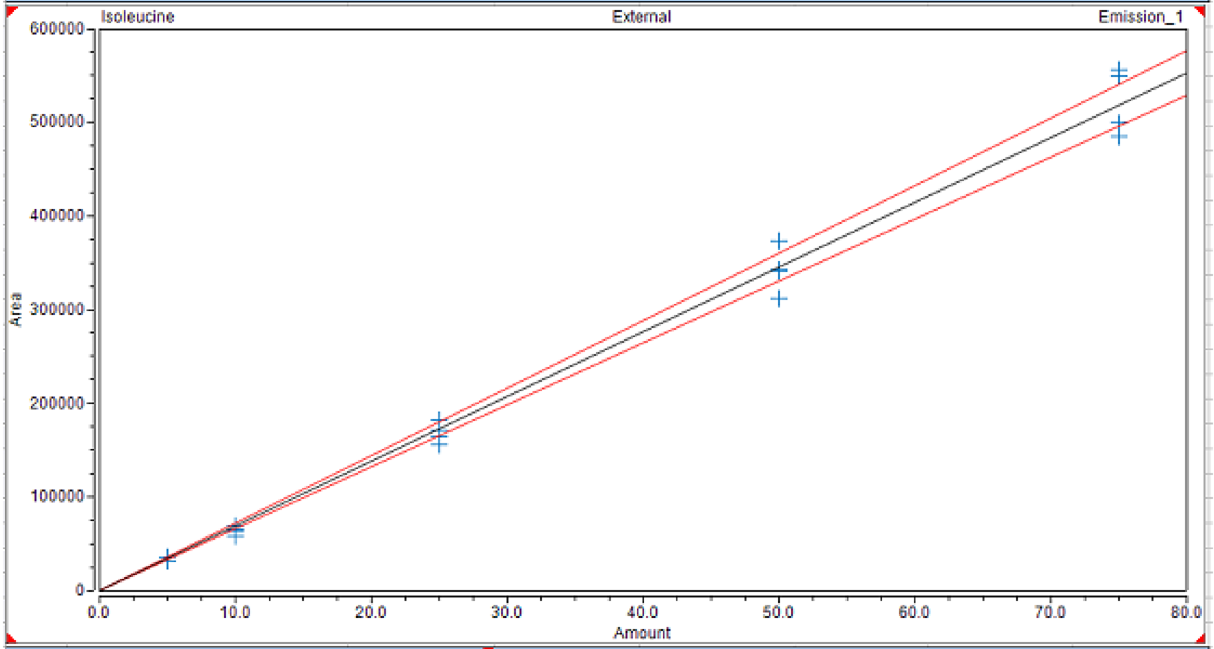

Calibration Results **Isoleucine**

No.	Injection Name	Calibration Level	X Value<br><br>Emission_1<br>Isoleucine	Y Value<br><br>Emission_1<br>Isoleucine	Y Value<br><br>Emission_1<br>Isoleucine	Area<br>counts*min<br>Emission_1<br>Isoleucine	Height<br>counts<br>Emission_1<br>Isoleucine
1	5uM	1	5.0000	31870.5027	31870.5027	31870.503	598415.400
2	10uM	2	10.0000	63834.0080	63834.0080	63834.008	1202138.824
3	10uM	2	10.0000	57539.2245	57539.2245	57539.225	1081223.260
4	25uM	3	25.0000	164688.9946	164688.9946	164688.995	3034946.201
5	25uM	3	25.0000	155147.5940	155147.5940	155147.594	2882122.640
6	50uM	4	50.0000	312314.2197	312314.2197	312314.220	5770715.402
7	50uM	4	50.0000	341921.7341	341921.7341	341921.734	6330442.289
8	75uM	5	75.0000	499791.0231	499791.0231	499791.023	9207837.827
9	75uM	5	75.0000	485257.3999	485257.3999	485257.400	8964762.922
10	5uM	1	5.0000	35873.8333	35873.8333	35873.833	654696.532
11	10uM	2	10.0000	68871.0317	68871.0317	68871.032	1259050.794
12	10uM	2	10.0000	64683.5480	64683.5480	64683.548	1199227.808
13	25uM	3	25.0000	181622.5008	181622.5008	181622.501	3333181.495
14	25uM	3	25.0000	171712.9427	171712.9427	171712.943	3128767.866
15	50uM	4	50.0000	343042.8347	343042.8347	343042.835	6246365.415
16	50uM	4	50.0000	373751.6167	373751.6167	373751.617	6839587.756
17	75uM	5	75.0000	555708.5092	555708.5092	555708.509	10184335.939
18	75uM	5	75.0000	548946.9387	548946.9387	548946.939	10057212.713

Calibration

Calibration Details	Leucine		
Calibration Type	Lin	*Offset (C0)*	0.0000
Evaluation Type	Area	*Slope (C1)*	5927.9748
Number of Calibration Points	18	*Curve (C2)*	0.0000
Number of disabled Calibration Points	0	*R-Square*	0.9894

Calibration Plot **Leucine**

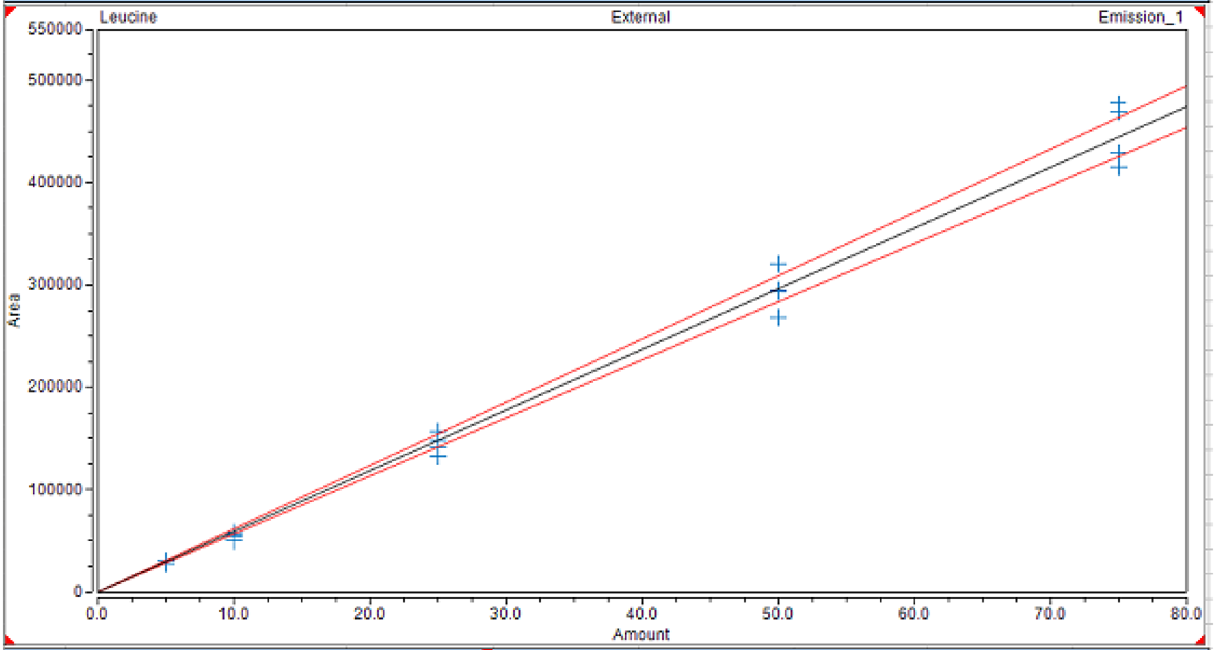

Calibration Results **Leucine**

No.	Injection Name	Calibration Level	X Value<br><br>Emission_1<br>Leucine	Y Value<br><br>Emission_1<br>Leucine	Y Value<br><br>Emission_1<br>Leucine	Area<br>counts*min<br>Emission_1<br>Leucine	Height<br>counts<br>Emission_1<br>Leucine
1	5uM	1	5.0000	27735.4727	27735.4727	27735.473	485707.571
2	10uM	2	10.0000	54415.0290	54415.0290	54415.029	972278.283
3	10uM	2	10.0000	50048.3960	50048.3960	50048.396	884099.820
4	25uM	3	25.0000	140844.7070	140844.7070	140844.707	2465334.372
5	25uM	3	25.0000	132553.5454	132553.5454	132553.545	2312812.540
6	50uM	4	50.0000	268427.0815	268427.0815	268427.081	4644794.941
7	50uM	4	50.0000	293064.9808	293064.9808	293064.981	5055540.789
8	75uM	5	75.0000	429819.5390	429819.5390	429819.539	7499567.478
9	75uM	5	75.0000	415316.3489	415316.3489	415316.349	7231652.014
10	5uM	1	5.0000	30187.7235	30187.7235	30187.724	532572.295
11	10uM	2	10.0000	58794.4675	58794.4675	58794.468	1023186.674
12	10uM	2	10.0000	55615.8285	55615.8285	55615.829	970347.073
13	25uM	3	25.0000	156391.9859	156391.9859	156391.986	2699587.145
14	25uM	3	25.0000	147541.2357	147541.2357	147541.236	2540832.078
15	50uM	4	50.0000	295062.9495	295062.9495	295062.949	5027631.581
16	50uM	4	50.0000	321074.9356	321074.9356	321074.936	5484660.337
17	75uM	5	75.0000	477203.6843	477203.6843	477203.684	8182784.403
18	75uM	5	75.0000	469041.3698	469041.3698	469041.370	8084412.148

Calibration

Calibration Details	Phenylalanine		
Calibration Type	Lin	*Offset (C0)*	0.0000
Evaluation Type	Area	*Slope (C1)*	5431.7345
Number of Calibration Points	18	*Curve (C2)*	0.0000
Number of disabled Calibration Points	0	*R-Square*	0.9915

Calibration Plot **Phenylalanine**

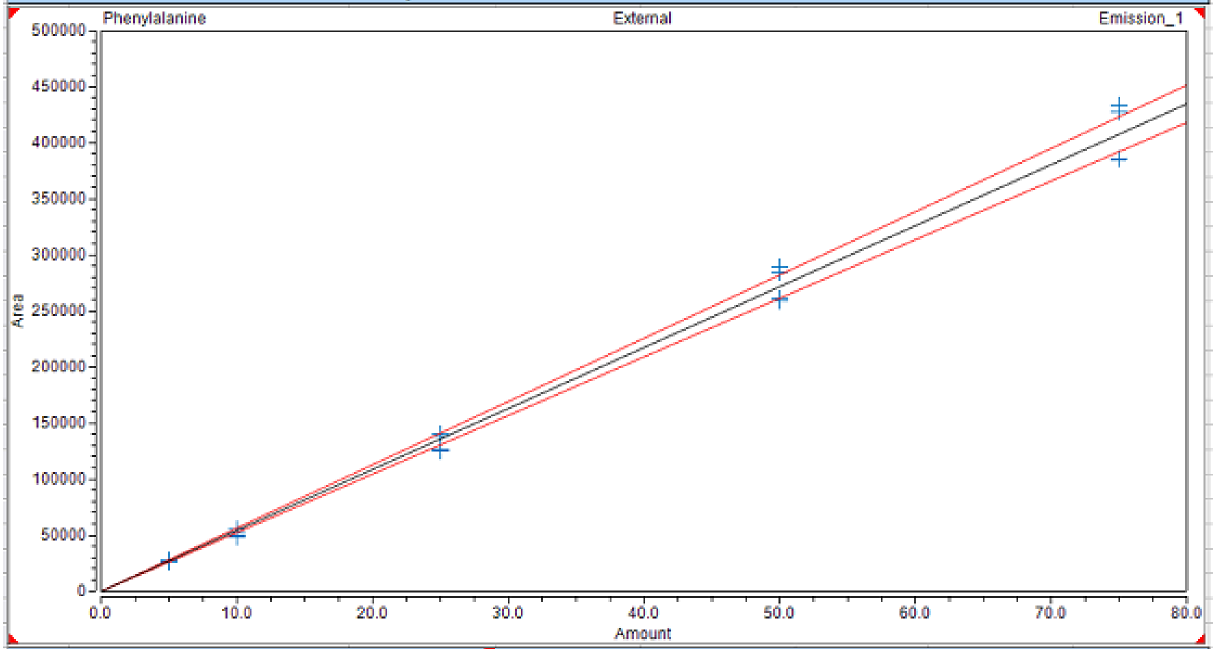

Calibration Results **Phenylalanine**

No.	Injection Name	Calibration Level	X Value<br><br>Emission_1 Phenylalanine	Y Value<br><br>Emission_1 Phenylalanine	Y Value<br><br>Emission_1 Phenylalanine	Area counts*min Emission_1 Phenylalanine	Height counts Emission_1 Phenylalanine
1	5uM	1	5.0000	25876.3929	25876.3929	25876.393	395625.222
2	10uM	2	10.0000	47943.1660	47943.1660	47943.166	762249.118
3	10uM	2	10.0000	49578.5310	49578.5310	49578.531	797035.042
4	25uM	3	25.0000	126474.8327	126474.8327	126474.833	2016880.040
5	25uM	3	25.0000	125024.9389	125024.9389	125024.939	1974477.582
6	50uM	4	50.0000	261505.5049	261505.5049	261505.505	4149760.129
7	50uM	4	50.0000	258971.1557	258971.1557	258971.156	4073124.208
8	75uM	5	75.0000	385174.6781	385174.6781	385174.678	6034586.696
9	75uM	5	75.0000	384904.0792	384904.0792	384904.079	5989712.115
10	5uM	1	5.0000	26834.9183	26834.9183	26834.918	432236.158
11	10uM	2	10.0000	52285.2691	52285.2691	52285.269	820386.506
12	10uM	2	10.0000	55329.1623	55329.1623	55329.162	863840.353
13	25uM	3	25.0000	140064.5370	140064.5370	140064.537	2198598.927
14	25uM	3	25.0000	137686.9579	137686.9579	137686.958	2142389.390
15	50uM	4	50.0000	289341.8195	289341.8195	289341.820	4491125.269
16	50uM	4	50.0000	284777.5390	284777.5390	284777.539	4448006.478
17	75uM	5	75.0000	427512.7195	427512.7195	427512.720	6639518.257
18	75uM	5	75.0000	432795.0101	432795.0101	432795.010	6729497.833

Calibration

Calibration Details	Tyrosine			
Calibration Type	Lin		*Offset (C0)*	0.0000
Evaluation Type	Area		*Slope (C1)*	0.0080
Number of Calibration Points	18		*Curve (C2)*	0.0000
Number of disabled Calibration Points	0		*R-Square*	0.9907

Calibration Plot **Tyrosine**

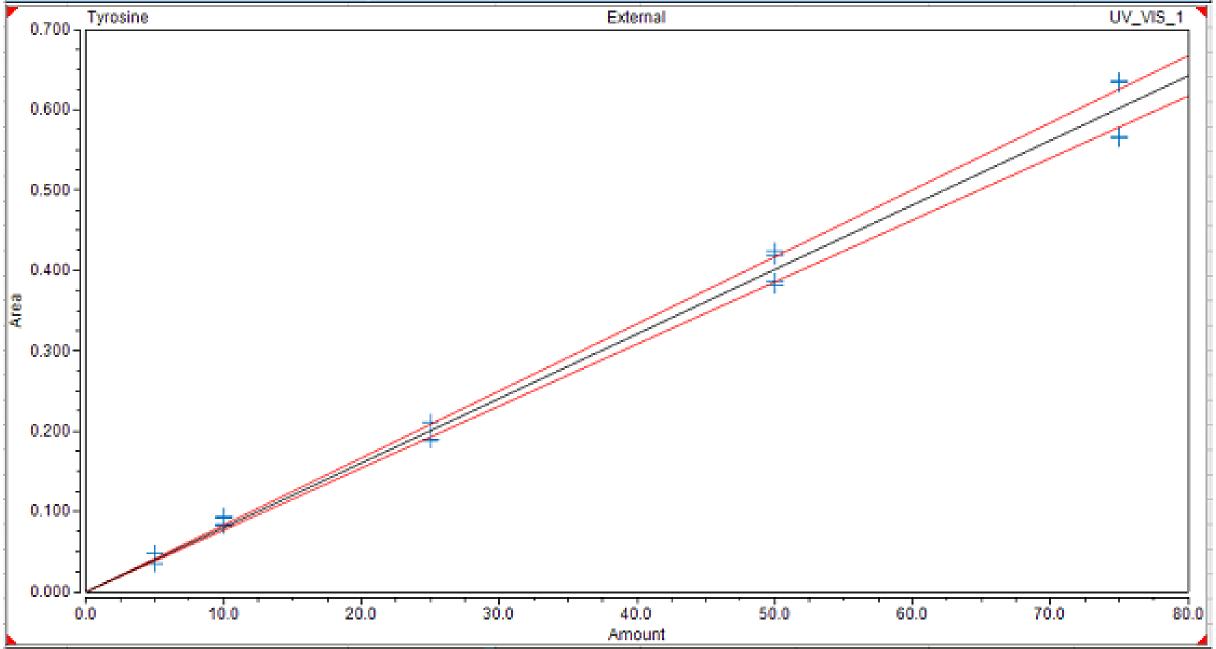

Calibration Results **Tyrosine**

No.	Injection Name	Calibration Level	X Value<br><br>UV_VIS_1<br>Tyrosine	Y Value<br><br>UV_VIS_1<br>Tyrosine	Y Value<br><br>UV_VIS_1<br>Tyrosine	Area<br>mAU*min<br>UV_VIS_1<br>Tyrosine	Height<br>mAU<br>UV_VIS_1<br>Tyrosine
1	5uM	1	5.0000	0.0485	0.0485	0.049	1.004
2	10uM	2	10.0000	0.0845	0.0845	0.085	1.929
3	10uM	2	10.0000	0.0830	0.0830	0.083	1.963
4	25uM	3	25.0000	0.1889	0.1889	0.189	4.833
5	25uM	3	25.0000	0.1899	0.1899	0.190	4.786
6	50uM	4	50.0000	0.3873	0.3873	0.387	9.985
7	50uM	4	50.0000	0.3824	0.3824	0.382	9.757
8	75uM	5	75.0000	0.5672	0.5672	0.567	14.555
9	75uM	5	75.0000	0.5640	0.5640	0.564	14.427
10	5uM	1	5.0000	0.0354	0.0354	0.035	0.969
11	10uM	2	10.0000	0.0929	0.0929	0.093	2.088
12	10uM	2	10.0000	0.0932	0.0932	0.093	2.175
13	25uM	3	25.0000	0.2117	0.2117	0.212	5.300
14	25uM	3	25.0000	0.2103	0.2103	0.210	5.216
15	50uM	4	50.0000	0.4243	0.4243	0.424	10.846
16	50uM	4	50.0000	0.4185	0.4185	0.418	10.683
17	75uM	5	75.0000	0.6334	0.6334	0.633	15.977
18	75uM	5	75.0000	0.6360	0.6360	0.636	16.296

Calibration

Calibration Details	Tryptophan		
Calibration Type	Lin	*Offset (C0)*	0.0000
Evaluation Type	Area	*Slope (C1)*	0.0076
Number of Calibration Points	18	*Curve (C2)*	0.0000
Number of disabled Calibration Points	0	*R-Square*	0.9867

Calibration Plot **Tryptophan**

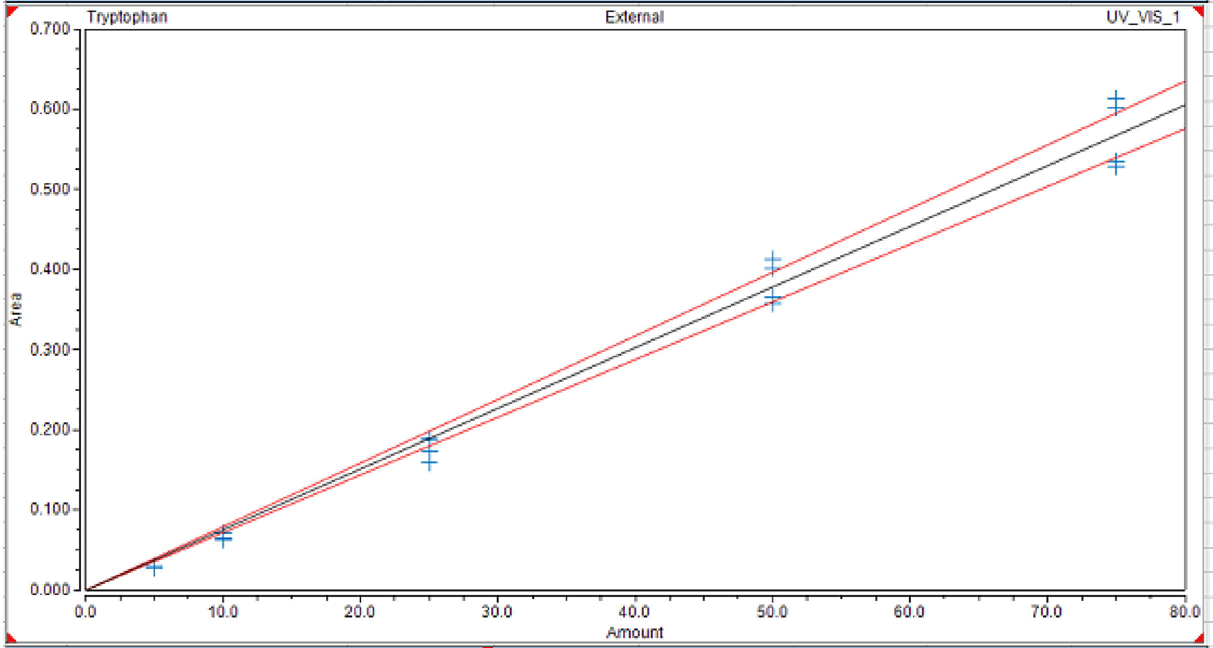

Calibration Results **Tryptophan**

No.	Injection Name	Calibration Level	X Value<br><br>UV_VIS_1<br>Tryptophan	Y Value<br><br>UV_VIS_1<br>Tryptophan	Y Value<br><br>UV_VIS_1<br>Tryptophan	Area<br>mAU*min<br>UV_VIS_1<br>Tryptophan	Height<br>mAU<br>UV_VIS_1<br>Tryptophan
1	5uM	1	5.0000	0.0267	0.0267	0.027	0.500
2	10uM	2	10.0000	0.0638	0.0638	0.064	1.051
3	10uM	2	10.0000	0.0624	0.0624	0.062	1.057
4	25uM	3	25.0000	0.1739	0.1739	0.174	2.764
5	25uM	3	25.0000	0.1595	0.1595	0.159	2.626
6	50uM	4	50.0000	0.3654	0.3654	0.365	5.706
7	50uM	4	50.0000	0.3575	0.3575	0.358	5.597
8	75uM	5	75.0000	0.5347	0.5347	0.535	8.320
9	75uM	5	75.0000	0.5290	0.5290	0.529	8.216
10	5uM	1	5.0000	0.0299	0.0299	0.030	0.546
11	10uM	2	10.0000	0.0640	0.0640	0.064	1.089
12	10uM	2	10.0000	0.0705	0.0705	0.070	1.176
13	25uM	3	25.0000	0.1890	0.1890	0.189	2.959
14	25uM	3	25.0000	0.1868	0.1868	0.187	2.954
15	50uM	4	50.0000	0.4123	0.4123	0.412	6.256
16	50uM	4	50.0000	0.4011	0.4011	0.401	6.181
17	75uM	5	75.0000	0.6014	0.6014	0.601	9.156
18	75uM	5	75.0000	0.6126	0.6126	0.613	9.315

Calibration data amino acids (Fig. 4 – 0 K and 16 K)

Fluorescence channel for all amino acids (see Methods for details).

Calibration

Calibration Details	Asparagine			
Calibration Type	Lin		*Offset (C0)*	0.0000
Evaluation Type	Area		*Slope (C1)*	13337.5252
Number of Calibration Points	30		*Curve (C2)*	0.0000
Number of disabled Calibration Points	0		*R-Square*	0.9947

Calibration Plot **Asparagine**

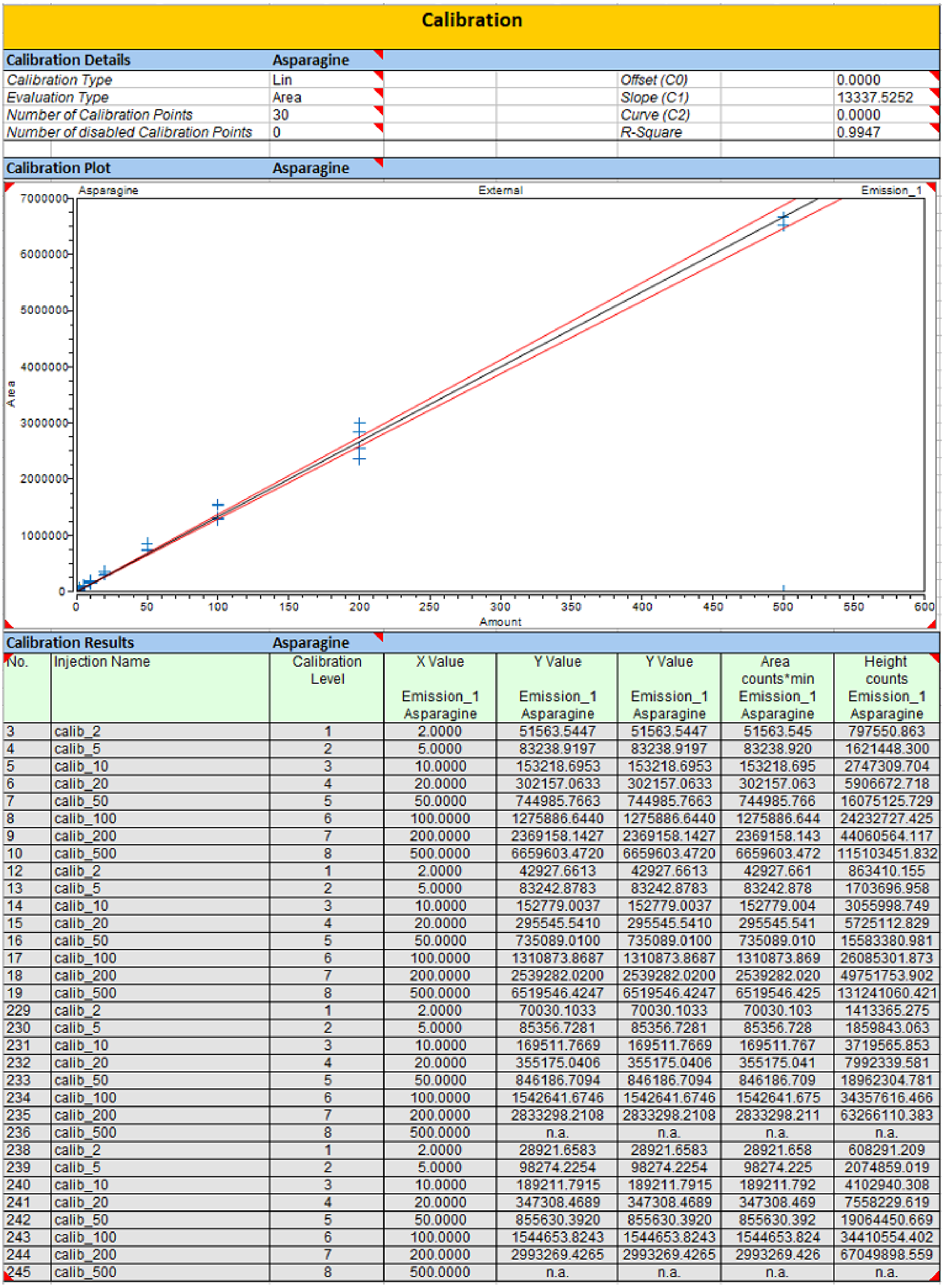

Calibration Results **Asparagine**

No.	Injection Name	Calibration Level	X Value<br><br>Emission_1 Asparagine	Y Value<br><br>Emission_1 Asparagine	Y Value<br><br>Emission_1 Asparagine	Area counts*min Emission_1 Asparagine	Height counts Emission_1 Asparagine
3	calib_2	1	2.0000	51563.5447	51563.5447	51563.545	797550.863
4	calib_5	2	5.0000	83238.9197	83238.9197	83238.920	1621448.300
5	calib_10	3	10.0000	153218.6953	153218.6953	153218.695	2747309.704
6	calib_20	4	20.0000	302157.0633	302157.0633	302157.063	5906672.718
7	calib_50	5	50.0000	744985.7663	744985.7663	744985.766	16075125.729
8	calib_100	6	100.0000	1275886.6440	1275886.6440	1275886.644	24232727.425
9	calib_200	7	200.0000	2369158.1427	2369158.1427	2369158.143	44060564.117
10	calib_500	8	500.0000	6659603.4720	6659603.4720	6659603.472	115103451.832
12	calib_2	1	2.0000	42927.6613	42927.6613	42927.661	863410.155
13	calib_5	2	5.0000	83242.8783	83242.8783	83242.878	1703696.958
14	calib_10	3	10.0000	152779.0037	152779.0037	152779.004	3055998.749
15	calib_20	4	20.0000	295545.5410	295545.5410	295545.541	5725112.829
16	calib_50	5	50.0000	735089.0100	735089.0100	735089.010	15583380.981
17	calib_100	6	100.0000	1310873.8687	1310873.8687	1310873.869	26085301.873
18	calib_200	7	200.0000	2539282.0200	2539282.0200	2539282.020	49751753.902
19	calib_500	8	500.0000	6519546.4247	6519546.4247	6519546.425	131241060.421
229	calib_2	1	2.0000	70030.1033	70030.1033	70030.103	1413365.275
230	calib_5	2	5.0000	85356.7281	85356.7281	85356.728	1859843.063
231	calib_10	3	10.0000	169511.7669	169511.7669	169511.767	3719565.853
232	calib_20	4	20.0000	355175.0406	355175.0406	355175.041	7992339.581
233	calib_50	5	50.0000	846186.7094	846186.7094	846186.709	18962304.781
234	calib_100	6	100.0000	1542641.6746	1542641.6746	1542641.675	34357616.466
235	calib_200	7	200.0000	2833298.2108	2833298.2108	2833298.211	63266110.383
236	calib_500	8	500.0000	n.a.	n.a.	n.a.	n.a.
238	calib_2	1	2.0000	28921.6583	28921.6583	28921.658	608291.209
239	calib_5	2	5.0000	98274.2254	98274.2254	98274.225	2074859.019
240	calib_10	3	10.0000	189211.7915	189211.7915	189211.792	4102940.308
241	calib_20	4	20.0000	347308.4689	347308.4689	347308.469	7558229.619
242	calib_50	5	50.0000	855630.3920	855630.3920	855630.392	19064450.669
243	calib_100	6	100.0000	1544653.8243	1544653.8243	1544653.824	34410554.402
244	calib_200	7	200.0000	2993269.4265	2993269.4265	2993269.426	67049898.559
245	calib_500	8	500.0000	n.a.	n.a.	n.a.	n.a.

Calibration

Calibration Details	Serine			
Calibration Type	Lin		*Offset (C0)*	0.0000
Evaluation Type	Area		*Slope (C1)*	14064.3520
Number of Calibration Points	31		*Curve (C2)*	0.0000
Number of disabled Calibration Points	0		*R-Square*	0.9920

Calibration Plot **Serine**

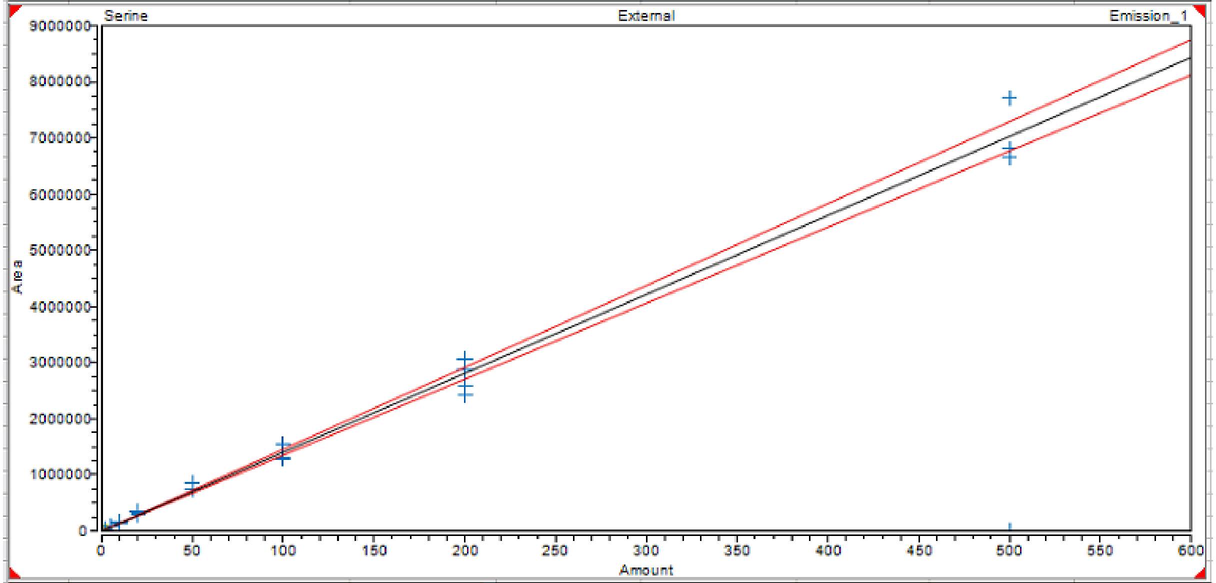

Calibration Results **Serine**

No.	Injection Name	Calibration Level	X Value Emission_1 Serine	Y Value Emission_1 Serine	Y Value Emission_1 Serine	Area counts*min Emission_1 Serine	Height counts Emission_1 Serine
3	calib_2	1	2.0000	31868.4987	31868.4987	31868.499	505004.906
4	calib_5	2	5.0000	73610.0303	73610.0303	73610.030	1420819.023
5	calib_10	3	10.0000	142131.6597	142131.6597	142131.660	2577621.356
6	calib_20	4	20.0000	303110.2633	303110.2633	303110.263	5729301.178
7	calib_50	5	50.0000	743062.0060	743062.0060	743062.006	14893515.835
8	calib_100	6	100.0000	1282522.1617	1282522.1617	1282522.162	24041981.023
9	calib_200	7	200.0000	2410932.6990	2410932.6990	2410932.699	44917205.329
10	calib_500	8	500.0000	6806335.2653	6806335.2653	6806335.265	119244010.120
12	calib_2	1	2.0000	29550.4383	29550.4383	29550.438	608108.393
13	calib_5	2	5.0000	69372.5113	69372.5113	69372.511	1368365.997
14	calib_10	3	10.0000	139633.7917	139633.7917	139633.792	2736163.320
15	calib_20	4	20.0000	284569.7750	284569.7750	284569.775	5435425.560
16	calib_50	5	50.0000	745040.1453	745040.1453	745040.145	14837208.019
17	calib_100	6	100.0000	1313098.8690	1313098.8690	1313098.869	25246652.949
18	calib_200	7	200.0000	2595737.4113	2595737.4113	2595737.411	49142206.229
19	calib_500	8	500.0000	6650657.9947	6650657.9947	6650657.995	129273244.783
229	calib_2	1	2.0000	39051.8220	39051.8220	39051.822	806312.007
230	calib_5	2	5.0000	74893.8830	74893.8830	74893.883	1543078.573
231	calib_10	3	10.0000	154976.1945	154976.1945	154976.195	3198100.982
232	calib_20	4	20.0000	349572.3397	349572.3397	349572.340	7186692.712
233	calib_50	5	50.0000	839859.4523	839859.4523	839859.452	17376636.019
234	calib_100	6	100.0000	1543838.4663	1543838.4663	1543838.466	31698387.456
235	calib_200	7	200.0000	2872107.8563	2872107.8563	2872107.856	59729665.396
236	calib_500	8	500.0000	n.a.	n.a.	n.a.	n.a.
238	calib_2	1	2.0000	20406.1412	20406.1412	20406.141	414667.096
239	calib_5	2	5.0000	78895.2633	78895.2633	78895.263	1636313.772
240	calib_10	3	10.0000	169420.3780	169420.3780	169420.378	3435895.688
241	calib_20	4	20.0000	330792.1680	330792.1680	330792.168	6764578.000
242	calib_50	5	50.0000	860400.9453	860400.9453	860400.945	17751924.978
243	calib_100	6	100.0000	1547493.2630	1547493.2630	1547493.263	31538756.976
244	calib_200	7	200.0000	3059222.1947	3059222.1947	3059222.195	62705792.019
245	calib_500	8	500.0000	7708359.8210	7708359.8210	7708359.821	157061764.909

Calibration

Calibration Details	Glycine			
Calibration Type	Lin		*Offset (C0)*	0.0000
Evaluation Type	Area		*Slope (C1)*	15001.9783
Number of Calibration Points	32		*Curve (C2)*	0.0000
Number of disabled Calibration Points	0		*R-Square*	0.9873

Calibration Plot **Glycine**

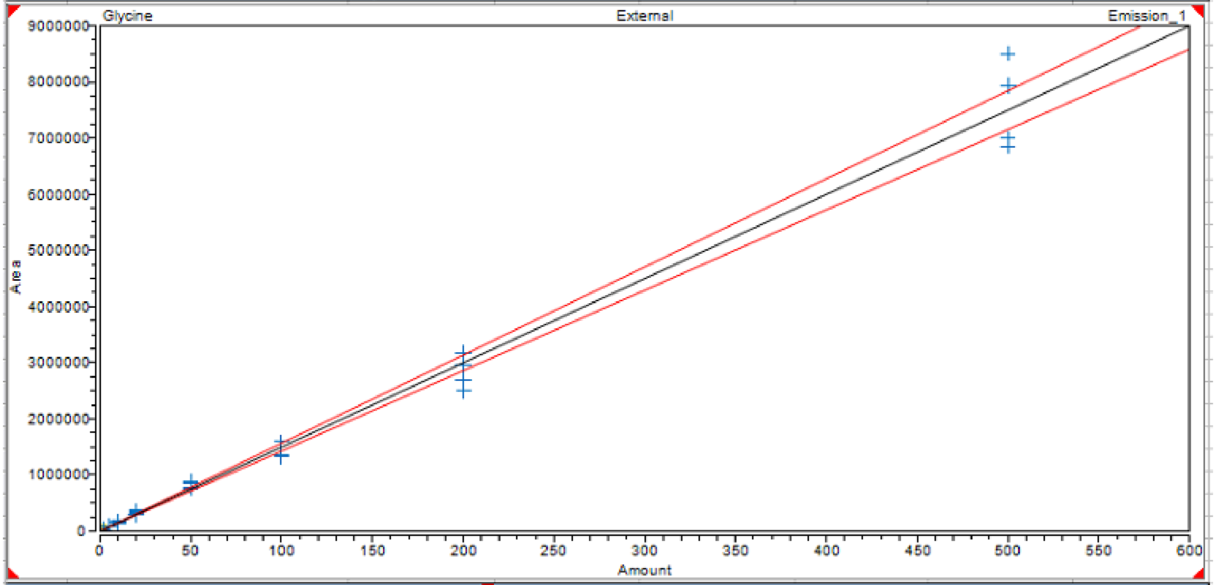

Calibration Results **Glycine**

No.	Injection Name	Calibration Level	X Value<br><br>Emission_1<br>Glycine	Y Value<br><br>Emission_1<br>Glycine	Y Value<br><br>Emission_1<br>Glycine	Area<br>counts*min<br>Emission_1<br>Glycine	Height<br>counts<br>Emission_1<br>Glycine
3	calib_2	1	2.0000	33572.3747	33572.3747	33572.375	466083.655
4	calib_5	2	5.0000	80750.8180	80750.8180	80750.818	1326952.947
5	calib_10	3	10.0000	148408.6003	148408.6003	148408.600	2495277.730
6	calib_20	4	20.0000	312465.4670	312465.4670	312465.467	5473943.725
7	calib_50	5	50.0000	756864.9732	756864.9732	756864.973	13801003.274
8	calib_100	6	100.0000	1326982.6405	1326982.6405	1326982.641	23450711.909
9	calib_200	7	200.0000	2492021.3030	2492021.3030	2492021.303	43699892.481
10	calib_500	8	500.0000	6990602.7444	6990602.7444	6990602.744	119784136.195
12	calib_2	1	2.0000	30422.1679	30422.1679	30422.168	557184.721
13	calib_5	2	5.0000	73036.3713	73036.3713	73036.371	1250444.319
14	calib_10	3	10.0000	145954.9823	145954.9823	145954.982	2532743.719
15	calib_20	4	20.0000	291428.2933	291428.2933	291428.293	5154974.100
16	calib_50	5	50.0000	764659.0542	764659.0542	764659.054	13846505.314
17	calib_100	6	100.0000	1353354.6444	1353354.6444	1353354.644	24323280.650
18	calib_200	7	200.0000	2683943.8939	2683943.8939	2683943.894	47629345.530
19	calib_500	8	500.0000	6848553.6312	6848553.6312	6848553.631	123572071.834
229	calib_2	1	2.0000	35709.2439	35709.2439	35709.244	653075.978
230	calib_5	2	5.0000	82115.0067	82115.0067	82115.007	1401951.234
231	calib_10	3	10.0000	162195.0245	162195.0245	162195.025	2914798.293
232	calib_20	4	20.0000	365648.4980	365648.4980	365648.498	6678139.418
233	calib_50	5	50.0000	856028.6128	856028.6128	856028.613	15991953.054
234	calib_100	6	100.0000	1593169.5199	1593169.5199	1593169.520	29636596.910
235	calib_200	7	200.0000	2966820.1247	2966820.1247	2966820.125	55740588.693
236	calib_500	8	500.0000	8498581.7957	8498581.7957	8498581.796	158401218.313
238	calib_2	1	2.0000	20533.4632	20533.4632	20533.463	370274.504
239	calib_5	2	5.0000	84960.7383	84960.7383	84960.738	1440680.952
240	calib_10	3	10.0000	175534.8013	175534.8013	175534.801	3154266.623
241	calib_20	4	20.0000	340443.4148	340443.4148	340443.415	6330921.630
242	calib_50	5	50.0000	887381.9566	887381.9566	887381.957	16187939.720
243	calib_100	6	100.0000	1592080.5177	1592080.5177	1592080.518	29121015.067
244	calib_200	7	200.0000	3170533.7331	3170533.7331	3170533.733	58912460.850
245	calib_500	8	500.0000	7936868.3085	7936868.3085	7936868.309	146582328.362

Calibration

Calibration Details	Threonine			
Calibration Type	Lin		*Offset (C0)*	0.0000
Evaluation Type	Area		*Slope (C1)*	16080.3907
Number of Calibration Points	32		*Curve (C2)*	0.0000
Number of disabled Calibration Points	0		*R-Square*	0.9871

Calibration Plot **Threonine**

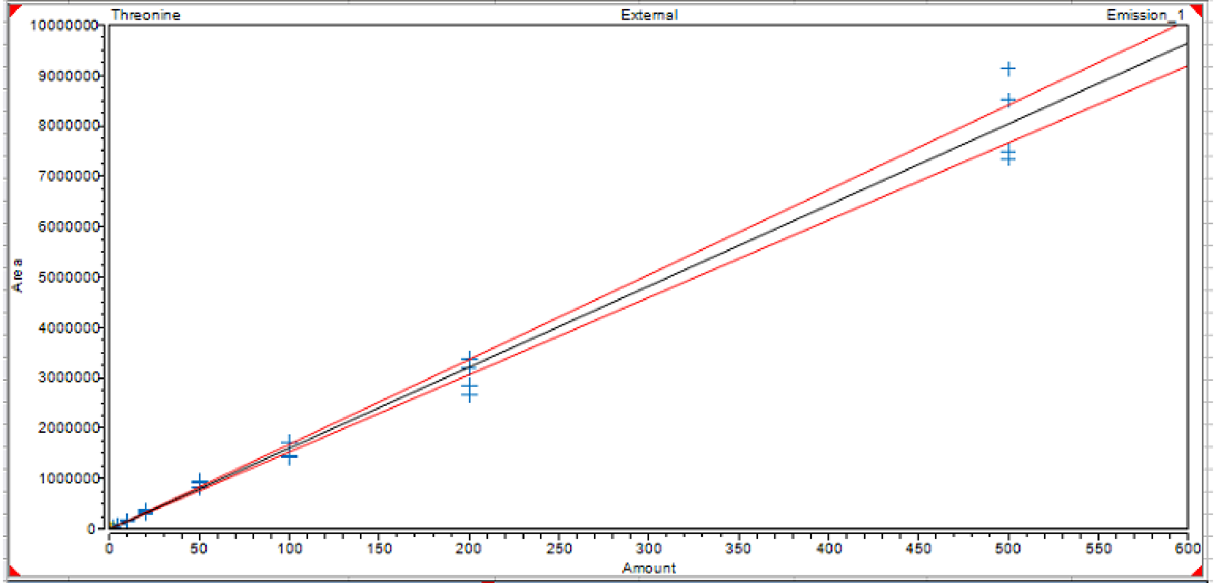

Calibration Results **Threonine**

No.	Injection Name	Calibration Level	X Value<br><br>Emission_1<br>Threonine	Y Value<br><br>Emission_1<br>Threonine	Y Value<br><br>Emission_1<br>Threonine	Area<br>counts*min<br>Emission_1<br>Threonine	Height<br>counts<br>Emission_1<br>Threonine
3	calib_2	1	2.0000	27618.9133	27618.9133	27618.913	450825.883
4	calib_5	2	5.0000	79596.8910	79596.8910	79596.891	1216121.795
5	calib_10	3	10.0000	160579.6613	160579.6613	160579.661	2424873.020
6	calib_20	4	20.0000	326574.1670	326574.1670	326574.167	5188397.869
7	calib_50	5	50.0000	820116.0190	820116.0190	820116.019	13150116.391
8	calib_100	6	100.0000	1417673.6968	1417673.6968	1417673.697	22597460.005
9	calib_200	7	200.0000	2666373.5864	2666373.5864	2666373.586	42350476.861
10	calib_500	8	500.0000	7495430.1708	7495430.1708	7495430.171	116475513.477
12	calib_2	1	2.0000	29570.1898	29570.1898	29570.190	500727.443
13	calib_5	2	5.0000	78236.2792	78236.2792	78236.279	1180638.440
14	calib_10	3	10.0000	159533.8532	159533.8532	159533.853	2433827.190
15	calib_20	4	20.0000	308196.5086	308196.5086	308196.509	5016307.098
16	calib_50	5	50.0000	821660.7823	821660.7823	821660.782	13069128.125
17	calib_100	6	100.0000	1451372.0347	1451372.0347	1451372.035	23183001.203
18	calib_200	7	200.0000	2840836.7273	2840836.7273	2840836.727	45406016.702
19	calib_500	8	500.0000	7340972.1353	7340972.1353	7340972.135	117722433.805
229	calib_2	1	2.0000	33233.6778	33233.6778	33233.678	571971.525
230	calib_5	2	5.0000	75594.3845	75594.3845	75594.384	1262616.629
231	calib_10	3	10.0000	162201.9935	162201.9935	162201.993	2715408.751
232	calib_20	4	20.0000	376664.2090	376664.2090	376664.209	6107391.158
233	calib_50	5	50.0000	929945.7460	929945.7460	929945.746	14874064.785
234	calib_100	6	100.0000	1704551.4313	1704551.4313	1704551.431	27446767.297
235	calib_200	7	200.0000	3182579.6043	3182579.6043	3182579.604	50900549.668
236	calib_500	8	500.0000	9128418.9373	9128418.9373	9128418.937	146950443.123
238	calib_2	1	2.0000	19986.7067	19986.7067	19986.707	334625.454
239	calib_5	2	5.0000	82015.4349	82015.4349	82015.435	1366842.673
240	calib_10	3	10.0000	178919.6398	178919.6398	178919.640	2949885.333
241	calib_20	4	20.0000	356659.5096	356659.5096	356659.510	5825430.560
242	calib_50	5	50.0000	946222.9163	946222.9163	946222.916	15339768.261
243	calib_100	6	100.0000	1713172.9780	1713172.9780	1713172.978	27656972.081
244	calib_200	7	200.0000	3372987.8487	3372987.8487	3372987.849	53287807.796
245	calib_500	8	500.0000	8511972.1437	8511972.1437	8511972.144	136756897.127

Calibration

Calibration Details	Alanine			
Calibration Type	Lin		*Offset (C0)*	0.0000
Evaluation Type	Area		*Slope (C1)*	11963.3366
Number of Calibration Points	32		*Curve (C2)*	0.0000
Number of disabled Calibration Points	0		*R-Square*	0.9849

Calibration Plot **Alanine**

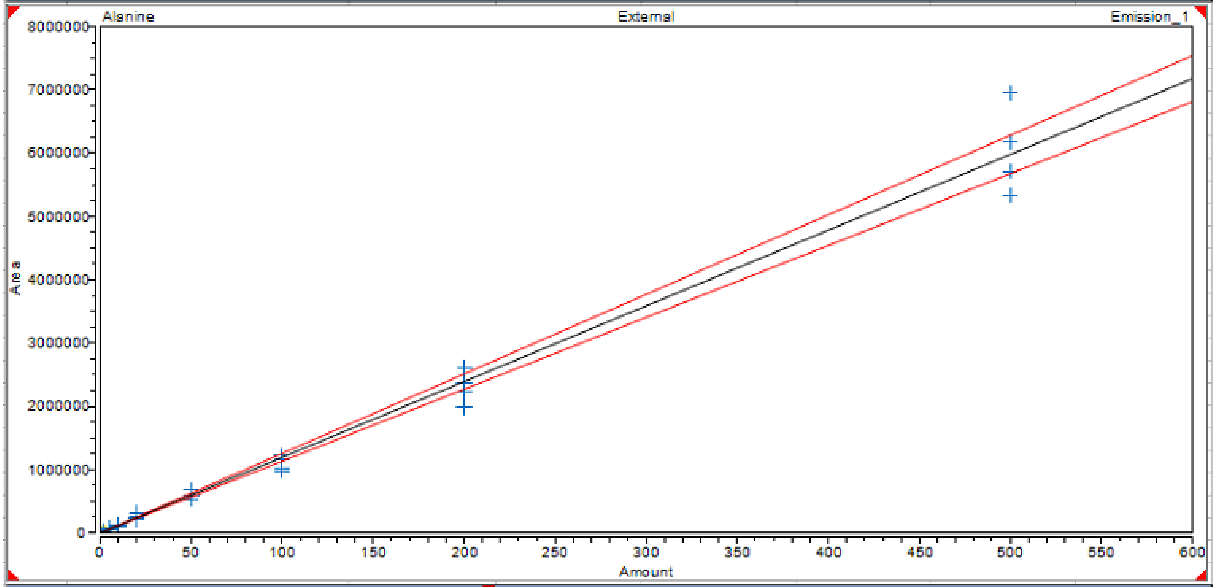

Calibration Results **Alanine**

No.	Injection Name	Calibration Level	X Value<br><br>Emission_1<br>Alanine	Y Value<br><br>Emission_1<br>Alanine	Y Value<br><br>Emission_1<br>Alanine	Area<br>counts*min<br>Emission_1<br>Alanine	Height<br>counts<br>Emission_1<br>Alanine
3	calib_2	1	2.0000	24240.6790	24240.6790	24240.679	368890.538
4	calib_5	2	5.0000	66248.0067	66248.0067	66248.007	979024.959
5	calib_10	3	10.0000	113043.3585	113043.3585	113043.359	1640222.851
6	calib_20	4	20.0000	271378.4453	271378.4453	271378.445	3992301.367
7	calib_50	5	50.0000	528455.3100	528455.3100	528455.310	7609934.352
8	calib_100	6	100.0000	1029543.1523	1029543.1523	1029543.152	14861332.000
9	calib_200	7	200.0000	1981519.3790	1981519.3790	1981519.379	28476800.851
10	calib_500	8	500.0000	5713180.8420	5713180.8420	5713180.842	81339277.733
12	calib_2	1	2.0000	17933.6183	17933.6183	17933.618	275159.598
13	calib_5	2	5.0000	43755.3293	43755.3293	43755.329	675525.585
14	calib_10	3	10.0000	100450.2657	100450.2657	100450.266	1463253.709
15	calib_20	4	20.0000	204824.3197	204824.3197	204824.320	2977679.671
16	calib_50	5	50.0000	590735.5205	590735.5205	590735.521	8665779.457
17	calib_100	6	100.0000	979799.6783	979799.6783	979799.678	14139369.742
18	calib_200	7	200.0000	2215131.6483	2215131.6483	2215131.648	31840488.711
19	calib_500	8	500.0000	5329316.6637	5329316.6637	5329316.664	75815962.674
229	calib_2	1	2.0000	31148.7475	31148.7475	31148.748	462853.049
230	calib_5	2	5.0000	66650.3937	66650.3937	66650.394	994534.147
231	calib_10	3	10.0000	122635.8333	122635.8333	122635.833	1815388.953
232	calib_20	4	20.0000	315276.8470	315276.8470	315276.847	4568330.021
233	calib_50	5	50.0000	599924.6190	599924.6190	599924.619	8560736.783
234	calib_100	6	100.0000	1232459.5367	1232459.5367	1232459.537	17642929.082
235	calib_200	7	200.0000	2360737.8732	2360737.8732	2360737.873	33979228.889
236	calib_500	8	500.0000	6946726.9783	6946726.9783	6946726.978	100194411.353
238	calib_2	1	2.0000	10912.9245	10912.9245	10912.925	178288.401
239	calib_5	2	5.0000	52204.9143	52204.9143	52204.914	771485.620
240	calib_10	3	10.0000	121816.6627	121816.6627	121816.663	1780346.724
241	calib_20	4	20.0000	237029.4883	237029.4883	237029.488	3434276.083
242	calib_50	5	50.0000	678074.9927	678074.9927	678074.993	9770168.440
243	calib_100	6	100.0000	1156638.5433	1156638.5433	1156638.543	16505549.365
244	calib_200	7	200.0000	2614759.5340	2614759.5340	2614759.534	37170202.974
245	calib_500	8	500.0000	6171487.4317	6171487.4317	6171487.432	87883733.356

Calibration

Calibration Details	Proline			
Calibration Type	Lin		*Offset (C0)*	0.0000
Evaluation Type	Area		*Slope (C1)*	3897.8384
Number of Calibration Points	32		*Curve (C2)*	0.0000
Number of disabled Calibration Points	0		*R-Square*	0.9869

Calibration Plot **Proline**

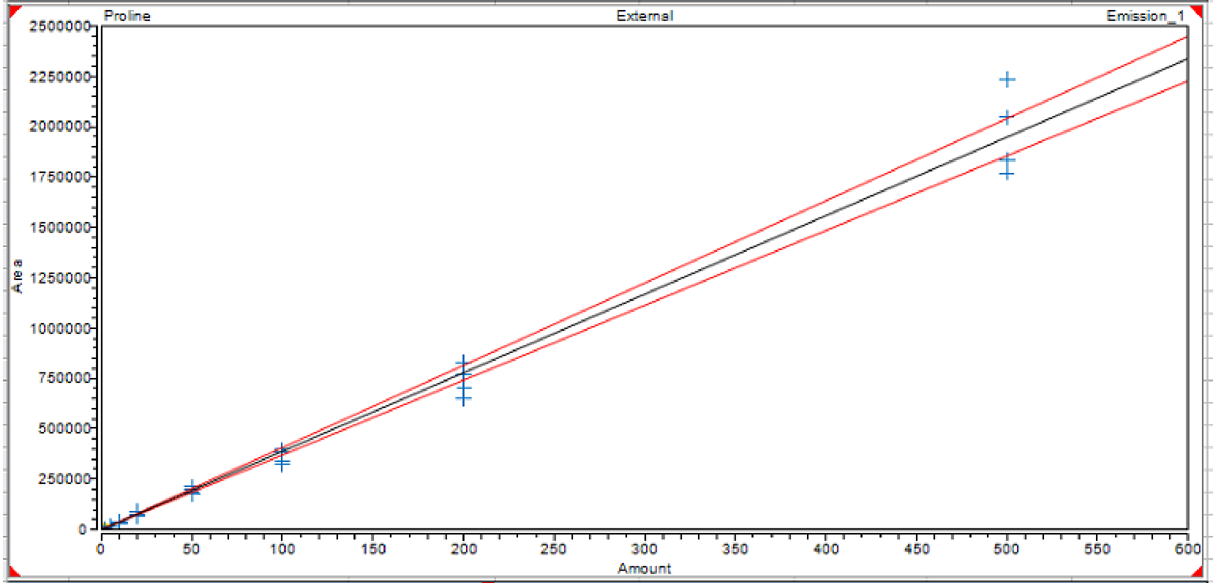

Calibration Results **Proline**

No.	Injection Name	Calibration Level	X Value<br><br>Emission_1<br>Proline	Y Value<br><br>Emission_1<br>Proline	Y Value<br><br>Emission_1<br>Proline	Area counts*min<br>Emission_1<br>Proline	Height counts<br>Emission_1<br>Proline
3	calib_2	1	2.0000	4875.3897	4875.3897	4875.390	83067.333
4	calib_5	2	5.0000	18446.5308	18446.5308	18446.531	270975.780
5	calib_10	3	10.0000	32706.8850	32706.8850	32706.885	453931.881
6	calib_20	4	20.0000	77918.9063	77918.9063	77918.906	1063447.371
7	calib_50	5	50.0000	177406.2853	177406.2853	177406.285	2351746.553
8	calib_100	6	100.0000	339212.7175	339212.7175	339212.718	4446060.500
9	calib_200	7	200.0000	651865.5727	651865.5727	651865.573	8459277.084
10	calib_500	8	500.0000	1834640.1463	1834640.1463	1834640.146	23772676.227
12	calib_2	1	2.0000	4970.4370	4970.4370	4970.437	82944.107
13	calib_5	2	5.0000	12833.3105	12833.3105	12833.311	197703.462
14	calib_10	3	10.0000	30643.2473	30643.2473	30643.247	428241.698
15	calib_20	4	20.0000	66029.4548	66029.4548	66029.455	891004.538
16	calib_50	5	50.0000	188429.0287	188429.0287	188429.029	2486428.152
17	calib_100	6	100.0000	324253.0625	324253.0625	324253.063	4337133.033
18	calib_200	7	200.0000	706227.1095	706227.1095	706227.110	9210701.116
19	calib_500	8	500.0000	1769375.3642	1769375.3642	1769375.364	22707906.851
229	calib_2	1	2.0000	5986.2973	5986.2973	5986.297	99517.639
230	calib_5	2	5.0000	15963.1668	15963.1668	15963.167	240387.704
231	calib_10	3	10.0000	35002.4700	35002.4700	35002.470	494608.380
232	calib_20	4	20.0000	88695.4173	88695.4173	88695.417	1220600.413
233	calib_50	5	50.0000	199005.2553	199005.2553	199005.255	2655117.274
234	calib_100	6	100.0000	398631.4580	398631.4580	398631.458	5279250.944
235	calib_200	7	200.0000	768056.1078	768056.1078	768056.108	9939748.267
236	calib_500	8	500.0000	2233860.1500	2233860.1500	2233860.150	28430080.429
238	calib_2	1	2.0000	3059.8040	3059.8040	3059.804	52243.673
239	calib_5	2	5.0000	15416.9698	15416.9698	15416.970	225962.917
240	calib_10	3	10.0000	36050.4403	36050.4403	36050.440	512582.494
241	calib_20	4	20.0000	74972.5047	74972.5047	74972.505	1030502.249
242	calib_50	5	50.0000	214066.6395	214066.6395	214066.640	2840207.592
243	calib_100	6	100.0000	384965.6442	384965.6442	384965.644	5033694.139
244	calib_200	7	200.0000	829917.8577	829917.8577	829917.858	10777317.584
245	calib_500	8	500.0000	2045963.9843	2045963.9843	2045963.984	26197378.556

Calibration

Calibration Details	Cysteine			
Calibration Type	Lin		*Offset (C0)*	0.0000
Evaluation Type	Area		*Slope (C1)*	1507.2410
Number of Calibration Points	32		*Curve (C2)*	0.0000
Number of disabled Calibration Points	0		*R-Square*	0.9773

Calibration Plot **Cysteine**

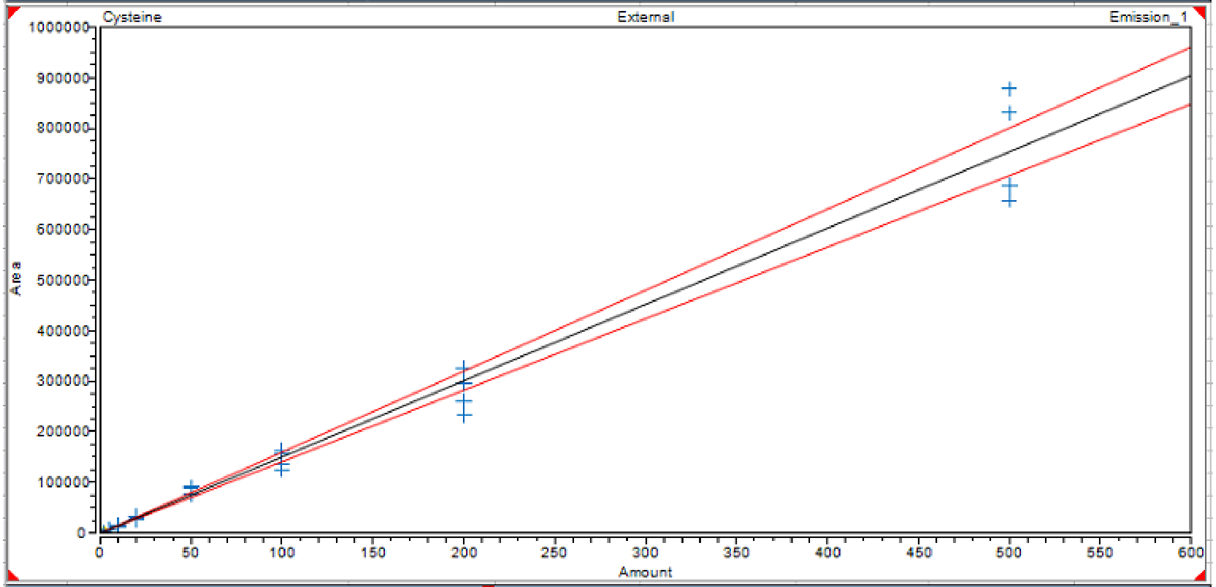

Calibration Results **Cysteine**

No.	Injection Name	Calibration Level	X Value Emission_1 Cysteine	Y Value Emission_1 Cysteine	Y Value Emission_1 Cysteine	Area counts*min Emission_1 Cysteine	Height counts Emission_1 Cysteine
3	calib_2	1	2.0000	1102.1100	1102.1100	1102.110	28055.045
4	calib_5	2	5.0000	4638.0837	4638.0837	4638.084	107031.355
5	calib_10	3	10.0000	11903.7348	11903.7348	11903.735	277671.108
6	calib_20	4	20.0000	28262.0570	28262.0570	28262.057	633857.577
7	calib_50	5	50.0000	76472.7277	76472.7277	76472.728	1692267.380
8	calib_100	6	100.0000	125839.6167	125839.6167	125839.617	2691777.025
9	calib_200	7	200.0000	232535.6346	232535.6346	232535.635	5044007.275
10	calib_500	8	500.0000	686452.0785	686452.0785	686452.078	14812152.371
12	calib_2	1	2.0000	2556.7190	2556.7190	2556.719	55881.222
13	calib_5	2	5.0000	5183.3035	5183.3035	5183.304	118933.599
14	calib_10	3	10.0000	12404.2820	12404.2820	12404.282	268409.188
15	calib_20	4	20.0000	26964.0860	26964.0860	26964.086	593156.193
16	calib_50	5	50.0000	76921.6386	76921.6386	76921.639	1676543.020
17	calib_100	6	100.0000	134989.6377	134989.6377	134989.638	2947535.509
18	calib_200	7	200.0000	260190.7079	260190.7079	260190.708	5650836.750
19	calib_500	8	500.0000	657974.9629	657974.9629	657974.963	14099349.986
229	calib_2	1	2.0000	1381.6043	1381.6043	1381.604	33776.298
230	calib_5	2	5.0000	5219.9623	5219.9623	5219.962	118734.599
231	calib_10	3	10.0000	13522.1995	13522.1995	13522.200	302336.234
232	calib_20	4	20.0000	33993.9525	33993.9525	33993.953	734529.426
233	calib_50	5	50.0000	88266.6470	88266.6470	88266.647	1906604.764
234	calib_100	6	100.0000	156271.8337	156271.8337	156271.834	3394575.761
235	calib_200	7	200.0000	295458.8226	295458.8226	295458.823	6261327.244
236	calib_500	8	500.0000	878294.0600	878294.0600	878294.060	19030927.836
238	calib_2	1	2.0000	1381.8030	1381.8030	1381.803	34501.675
239	calib_5	2	5.0000	6015.3403	6015.3403	6015.340	140712.532
240	calib_10	3	10.0000	14460.1077	14460.1077	14460.108	327820.665
241	calib_20	4	20.0000	32372.0466	32372.0466	32372.047	698867.468
242	calib_50	5	50.0000	91475.8407	91475.8407	91475.841	1956195.002
243	calib_100	6	100.0000	163042.1713	163042.1713	163042.171	3504229.050
244	calib_200	7	200.0000	325929.9988	325929.9988	325929.999	6905241.136
245	calib_500	8	500.0000	830046.4910	830046.4910	830046.491	17725602.474

Calibration

Calibration Details	Valine		
Calibration Type	Lin	*Offset (C0)*	0.0000
Evaluation Type	Area	*Slope (C1)*	16030.4318
Number of Calibration Points	31	*Curve (C2)*	0.0000
Number of disabled Calibration Points	0	*R-Square*	0.9921

Calibration Plot **Valine**

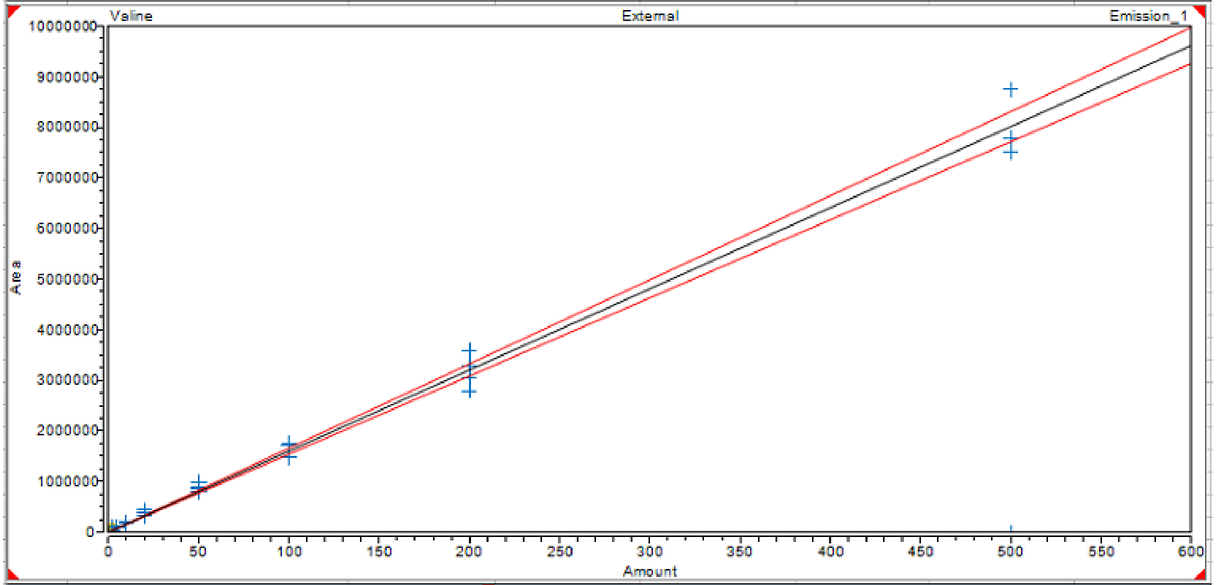

Calibration Results **Valine**

No.	Injection Name	Calibration Level	X Value<br><br>Emission_1<br>Valine	Y Value<br><br>Emission_1<br>Valine	Y Value<br><br>Emission_1<br>Valine	Area<br>counts*min<br>Emission_1<br>Valine	Height<br>counts<br>Emission_1<br>Valine
3	calib_2	1	2.0000	76672.1925	76672.1925	76672.192	1148672.830
4	calib_5	2	5.0000	116461.3563	116461.3563	116461.356	1941551.255
5	calib_10	3	10.0000	175209.9621	175209.9621	175209.962	3153996.488
6	calib_20	4	20.0000	373315.9421	373315.9421	373315.942	6868616.583
7	calib_50	5	50.0000	797416.9941	797416.9941	797416.994	14962396.064
8	calib_100	6	100.0000	1491921.6430	1491921.6430	1491921.643	27700216.429
9	calib_200	7	200.0000	2789606.1200	2789606.1200	2789606.120	52576269.403
10	calib_500	8	500.0000	7785698.6883	7785698.6883	7785698.688	146856901.568
12	calib_2	1	2.0000	38604.9360	38604.9360	38604.936	672974.612
13	calib_5	2	5.0000	79262.5128	79262.5128	79262.513	1417331.399
14	calib_10	3	10.0000	165896.9682	165896.9682	165896.968	2997054.130
15	calib_20	4	20.0000	320746.8763	320746.8763	320746.876	5960927.519
16	calib_50	5	50.0000	854805.3313	854805.3313	854805.331	16198444.766
17	calib_100	6	100.0000	1471232.3173	1471232.3173	1471232.317	27547515.678
18	calib_200	7	200.0000	3043575.8223	3043575.8223	3043575.822	57486145.970
19	calib_500	8	500.0000	7507809.3612	7507809.3612	7507809.361	142253447.906
229	calib_2	1	2.0000	93618.9737	93618.9737	93618.974	1448131.647
230	calib_5	2	5.0000	115358.2120	115358.2120	115358.212	1948555.261
231	calib_10	3	10.0000	188808.7110	188808.7110	188808.711	3385284.578
232	calib_20	4	20.0000	430973.3548	430973.3548	430973.355	7982417.061
233	calib_50	5	50.0000	898961.3120	898961.3120	898961.312	16848418.767
234	calib_100	6	100.0000	1760831.2848	1760831.2848	1760831.285	33086084.099
235	calib_200	7	200.0000	3297554.6235	3297554.6235	3297554.624	61607834.377
236	calib_500	8	500.0000	n.a.	n.a.	n.a.	n.a.
238	calib_2	1	2.0000	24690.2802	24690.2802	24690.280	438526.906
239	calib_5	2	5.0000	91711.7340	91711.7340	91711.734	1652165.173
240	calib_10	3	10.0000	197170.4405	197170.4405	197170.441	3636432.736
241	calib_20	4	20.0000	374797.8749	374797.8749	374797.875	6843712.495
242	calib_50	5	50.0000	983083.7150	983083.7150	983083.715	18025946.900
243	calib_100	6	100.0000	1712291.7151	1712291.7151	1712291.715	31673019.150
244	calib_200	7	200.0000	3592881.9949	3592881.9949	3592881.995	65708515.343
245	calib_500	8	500.0000	8743134.9226	8743134.9226	8743134.923	161538498.151

Calibration

Calibration Details	Isoleucine			
Calibration Type	Lin		*Offset (C0)*	0.0000
Evaluation Type	Area		*Slope (C1)*	17735.9883
Number of Calibration Points	31		*Curve (C2)*	0.0000
Number of disabled Calibration Points	0		*R-Square*	0.9927

Calibration Plot **Isoleucine**

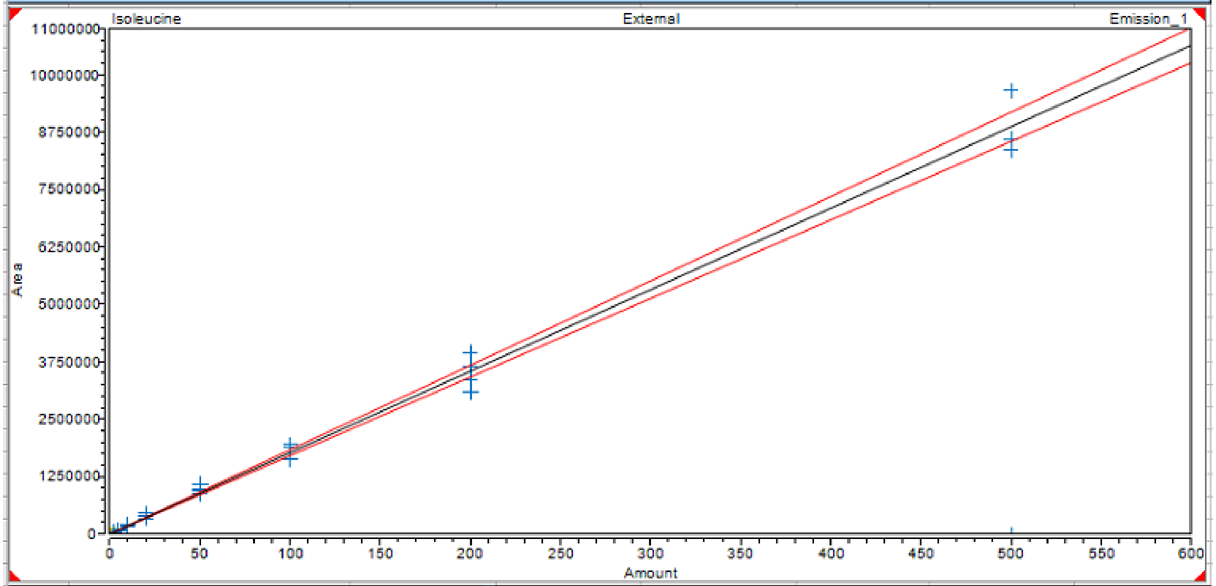

Calibration Results **Isoleucine**

No.	Injection Name	Calibration Level	X Value Emission_1 Isoleucine	Y Value Emission_1 Isoleucine	Y Value Emission_1 Isoleucine	Area counts*min Emission_1 Isoleucine	Height counts Emission_1 Isoleucine
3	calib_2	1	2.0000	35091.7193	35091.7193	35091.719	582280.558
4	calib_5	2	5.0000	90518.0840	90518.0840	90518.084	1522334.774
5	calib_10	3	10.0000	172068.2837	172068.2837	172068.284	2866486.572
6	calib_20	4	20.0000	394448.7349	394448.7349	394448.735	6418596.111
7	calib_50	5	50.0000	871610.1974	871610.1974	871610.197	14335491.750
8	calib_100	6	100.0000	1641930.0060	1641930.0060	1641930.006	26644729.456
9	calib_200	7	200.0000	3088634.7760	3088634.7760	3088634.776	50312562.024
10	calib_500	8	500.0000	8599762.5605	8599762.5605	8599762.561	140682843.015
12	calib_2	1	2.0000	33815.7867	33815.7867	33815.787	554765.182
13	calib_5	2	5.0000	75699.0044	75699.0044	75699.004	1252021.249
14	calib_10	3	10.0000	170651.2900	170651.2900	170651.290	2782993.479
15	calib_20	4	20.0000	342668.1381	342668.1381	342668.138	5616620.174
16	calib_50	5	50.0000	931514.8635	931514.8635	931514.864	15248650.092
17	calib_100	6	100.0000	1625951.9276	1625951.9276	1625951.928	26412976.366
18	calib_200	7	200.0000	3365283.8870	3365283.8870	3365283.887	55560659.820
19	calib_500	8	500.0000	8354231.7951	8354231.7951	8354231.795	137777885.097
229	calib_2	1	2.0000	40683.7840	40683.7840	40683.784	703127.982
230	calib_5	2	5.0000	91510.4908	91510.4908	91510.491	1531482.304
231	calib_10	3	10.0000	189932.5893	189932.5893	189932.589	3116752.758
232	calib_20	4	20.0000	457243.7063	457243.7063	457243.706	7586603.368
233	calib_50	5	50.0000	985136.3750	985136.3750	985136.375	16199224.386
234	calib_100	6	100.0000	1937625.6906	1937625.6906	1937625.691	31856282.478
235	calib_200	7	200.0000	3660547.3883	3660547.3883	3660547.388	59983295.951
236	calib_500	8	500.0000	n.a.	n.a.	n.a.	n.a.
238	calib_2	1	2.0000	21429.9593	21429.9593	21429.959	355757.877
239	calib_5	2	5.0000	87082.0797	87082.0797	87082.080	1458128.816
240	calib_10	3	10.0000	204031.2647	204031.2647	204031.265	3358691.011
241	calib_20	4	20.0000	398309.8654	398309.8654	398309.865	6542776.997
242	calib_50	5	50.0000	1073050.4195	1073050.4195	1073050.419	17639318.315
243	calib_100	6	100.0000	1876279.4986	1876279.4986	1876279.499	30668277.840
244	calib_200	7	200.0000	3949631.5061	3949631.5061	3949631.506	64770368.369
245	calib_500	8	500.0000	9663621.5695	9663621.5695	9663621.570	158727715.872

Calibration

Calibration Details	Leucine		Offset (C0)	0.0000
Calibration Type	Lin		*Offset (C0)*	0.0000
Evaluation Type	Area		*Slope (C1)*	18439.7022
Number of Calibration Points	32		*Curve (C2)*	0.0000
Number of disabled Calibration Points	0		*R-Square*	0.9873

Calibration Plot Leucine

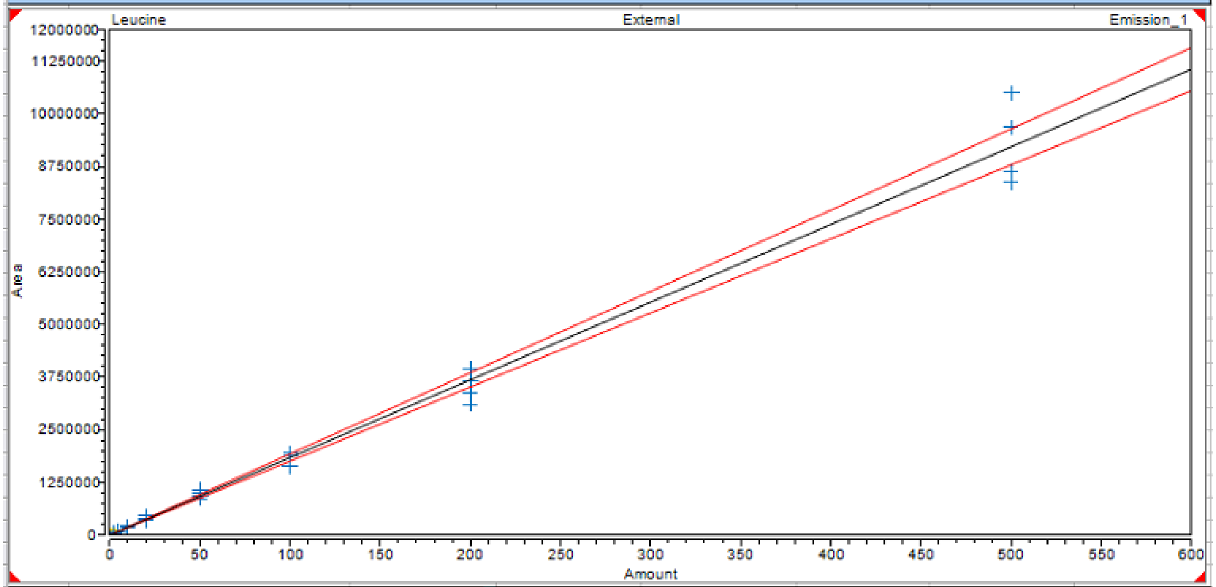

Calibration Results Leucine

No.	Injection Name	Calibration Level	X Value<br><br>Emission_1<br>Leucine	Y Value<br><br>Emission_1<br>Leucine	Y Value<br><br>Emission_1<br>Leucine	Area<br>counts*min<br>Emission_1<br>Leucine	Height<br>counts<br>Emission_1<br>Leucine
3	calib_2	1	2.0000	34008.5567	34008.5567	34008.557	544332.949
4	calib_5	2	5.0000	89692.0357	89692.0357	89692.036	1440645.600
5	calib_10	3	10.0000	170247.4910	170247.4910	170247.491	2668348.588
6	calib_20	4	20.0000	390062.1041	390062.1041	390062.104	6031401.037
7	calib_50	5	50.0000	868449.6716	868449.6716	868449.672	13489452.627
8	calib_100	6	100.0000	1636962.4220	1636962.4220	1636962.422	25013268.946
9	calib_200	7	200.0000	3097022.2664	3097022.2664	3097022.266	47122123.725
10	calib_500	8	500.0000	8633040.6295	8633040.6295	8633040.630	131545202.983
12	calib_2	1	2.0000	32185.1320	32185.1320	32185.132	519529.841
13	calib_5	2	5.0000	74994.3409	74994.3409	74994.341	1172773.772
14	calib_10	3	10.0000	167766.2417	167766.2417	167766.242	2615452.974
15	calib_20	4	20.0000	340319.2012	340319.2012	340319.201	5243697.492
16	calib_50	5	50.0000	927008.9190	927008.9190	927008.919	14186241.301
17	calib_100	6	100.0000	1630178.0088	1630178.0088	1630178.009	24906205.373
18	calib_200	7	200.0000	3363791.0513	3363791.0513	3363791.051	51688358.052
19	calib_500	8	500.0000	8373842.6176	8373842.6176	8373842.618	128691667.424
229	calib_2	1	2.0000	41769.3203	41769.3203	41769.320	670027.589
230	calib_5	2	5.0000	89532.2463	89532.2463	89532.246	1449149.641
231	calib_10	3	10.0000	187663.1597	187663.1597	187663.160	2935050.914
232	calib_20	4	20.0000	454614.8424	454614.8424	454614.842	7072202.339
233	calib_50	5	50.0000	981407.8187	981407.8187	981407.819	15165755.802
234	calib_100	6	100.0000	1937271.3399	1937271.3399	1937271.340	29654481.129
235	calib_200	7	200.0000	3654677.9612	3654677.9612	3654677.961	55842746.586
236	calib_500	8	500.0000	10507837.4629	10507837.4629	10507837.463	160504800.451
238	calib_2	1	2.0000	20316.6127	20316.6127	20316.613	336937.294
239	calib_5	2	5.0000	87139.6868	87139.6868	87139.687	1373293.580
240	calib_10	3	10.0000	202812.0551	202812.0551	202812.055	3151404.291
241	calib_20	4	20.0000	396939.0266	396939.0266	396939.027	6081527.286
242	calib_50	5	50.0000	1069269.5857	1069269.5857	1069269.586	16619054.023
243	calib_100	6	100.0000	1868614.0422	1868614.0422	1868614.042	29025970.868
244	calib_200	7	200.0000	3951369.0265	3951369.0265	3951369.026	60963249.936
245	calib_500	8	500.0000	9679658.6136	9679658.6136	9679658.614	147447603.363

Calibration

Calibration Details	Phenylalanine			
Calibration Type	Lin		*Offset (C0)*	0.0000
Evaluation Type	Area		*Slope (C1)*	15084.0472
Number of Calibration Points	32		*Curve (C2)*	0.0000
Number of disabled Calibration Points	0		*R-Square*	0.9864

Calibration Plot **Phenylalanine**

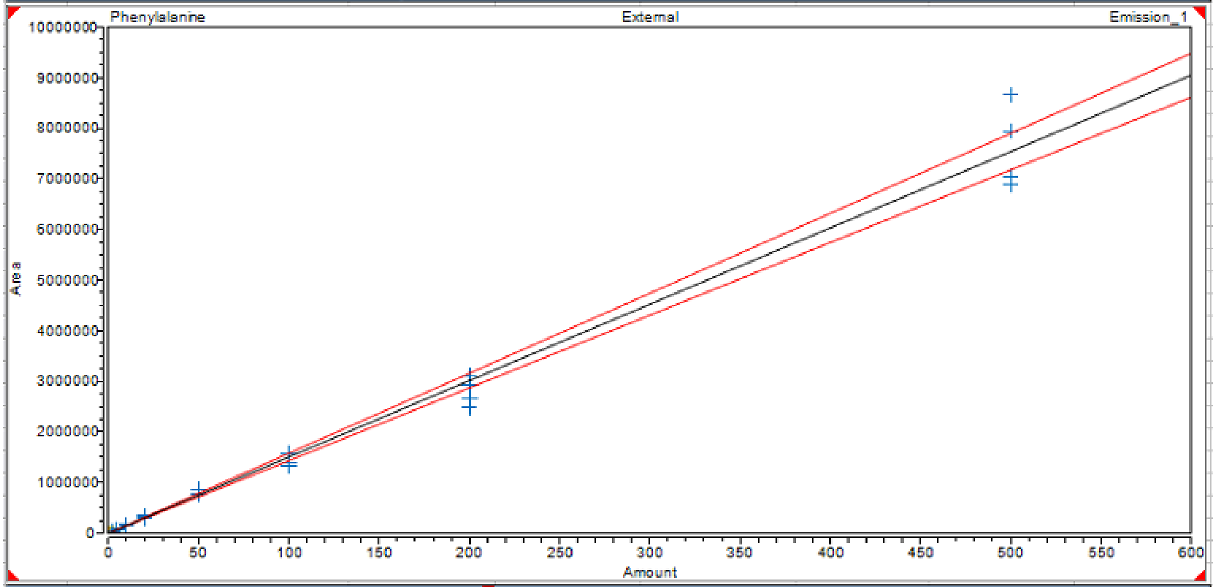

Calibration Results **Phenylalanine**

No.	Injection Name	Calibration Level	X Value<br><br>Emission_1<br>Phenylalanine	Y Value<br><br>Emission_1<br>Phenylalanine	Y Value<br><br>Emission_1<br>Phenylalanine	Area<br>counts*min<br>Emission_1<br>Phenylalanine	Height<br>counts<br>Emission_1<br>Phenylalanine
3	calib_2	1	2.0000	27547.8266	27547.8266	27547.827	376264.357
4	calib_5	2	5.0000	71032.3257	71032.3257	71032.326	981331.836
5	calib_10	3	10.0000	138770.5540	138770.5540	138770.554	1941273.344
6	calib_20	4	20.0000	306110.2539	306110.2539	306110.254	4177769.975
7	calib_50	5	50.0000	766559.2634	766559.2634	766559.263	10565590.833
8	calib_100	6	100.0000	1323619.1514	1323619.1514	1323619.151	18266815.904
9	calib_200	7	200.0000	2475468.6084	2475468.6084	2475468.608	34203020.813
10	calib_500	8	500.0000	7046137.5664	7046137.5664	7046137.566	96649072.932
12	calib_2	1	2.0000	28325.5822	28325.5822	28325.582	408930.188
13	calib_5	2	5.0000	62834.5477	62834.5477	62834.548	919598.675
14	calib_10	3	10.0000	143235.0217	143235.0217	143235.022	1995552.316
15	calib_20	4	20.0000	292658.3315	292658.3315	292658.331	4034777.880
16	calib_50	5	50.0000	765239.1929	765239.1929	765239.193	10491351.193
17	calib_100	6	100.0000	1382151.5857	1382151.5857	1382151.586	18820396.102
18	calib_200	7	200.0000	2667295.7228	2667295.7228	2667295.723	37077172.098
19	calib_500	8	500.0000	6891200.1795	6891200.1795	6891200.179	94470887.403
229	calib_2	1	2.0000	29875.5350	29875.5350	29875.535	440051.048
230	calib_5	2	5.0000	70652.7599	70652.7599	70652.760	975756.229
231	calib_10	3	10.0000	152466.2358	152466.2358	152466.236	2135840.211
232	calib_20	4	20.0000	347866.7685	347866.7685	347866.768	4826530.733
233	calib_50	5	50.0000	861763.2933	861763.2933	861763.293	11944721.145
234	calib_100	6	100.0000	1572286.8927	1572286.8927	1572286.893	21514377.152
235	calib_200	7	200.0000	2938524.9089	2938524.9089	2938524.909	40462762.992
236	calib_500	8	500.0000	8653282.2387	8653282.2387	8653282.239	118085855.032
238	calib_2	1	2.0000	18337.7997	18337.7997	18337.800	268798.816
239	calib_5	2	5.0000	77898.7647	77898.7647	77898.765	1105523.000
240	calib_10	3	10.0000	169275.3410	169275.3410	169275.341	2355214.959
241	calib_20	4	20.0000	336916.6573	336916.6573	336916.657	4694700.161
242	calib_50	5	50.0000	871346.8015	871346.8015	871346.801	12075253.431
243	calib_100	6	100.0000	1579040.9070	1579040.9070	1579040.907	22078199.306
244	calib_200	7	200.0000	3119716.3082	3119716.3082	3119716.308	42710880.571
245	calib_500	8	500.0000	7931639.6582	7931639.6582	7931639.658	108368924.824

Calibration data dimerization of glycine (Fig. 5)
Detection with mass spectrometry (see Methods for details).

Calibration

Calibration Details	Gly		
Calibration Type	Poly2(vx)	*Offset (C0)*	0.0000
Evaluation Type	Area	*Slope (C1)*	255808.8699
Number of Calibration Points	12	*Curve (C2)*	103098.4066
Number of disabled Calibration Points	0	*R-Square*	0.9980

Calibration Plot Gly

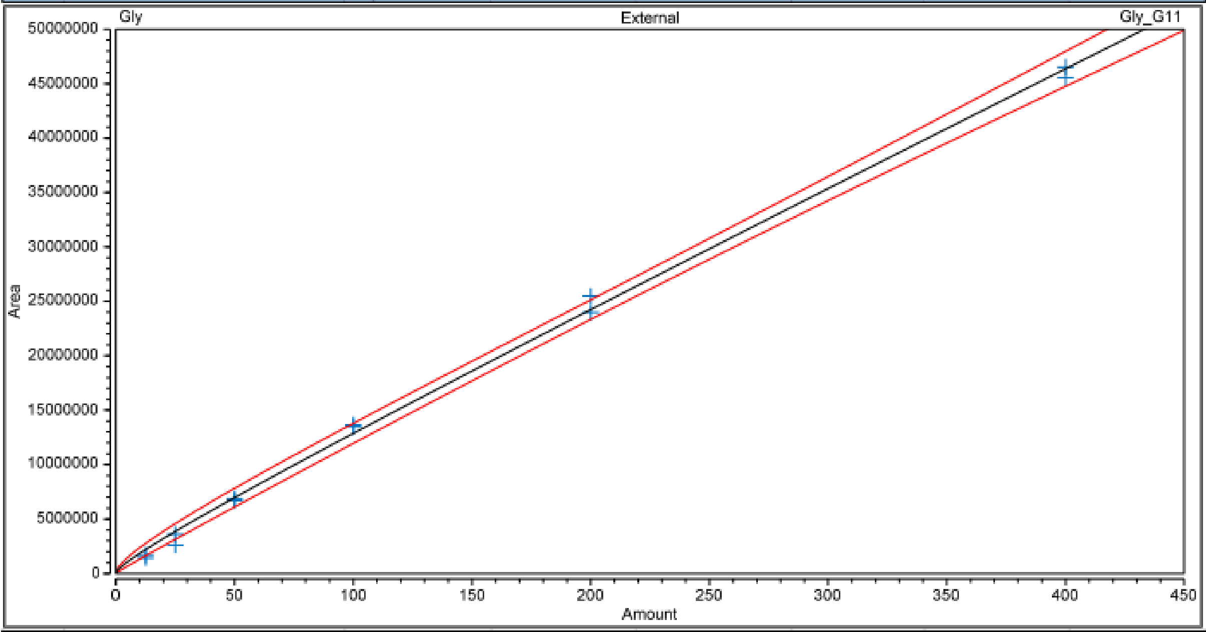

Calibration Results Gly

No.	Injection Name	Calibration Level	X Value Gly_G11 Gly	Y Value Gly_G11 Gly	Y Value Gly_G11 Gly	Area counts*min Gly_G11 Gly	Height counts Gly_G11 Gly
26	G_12_20230603230415	1	12.5000	1429600.5087	1429600.5087	1429600.509	25421419.364
27	GG_12_20230603233016	2	n.a.	n.a.	n.a.	n.a.	n.a.
28	G_25_20230604002216	3	25.0000	3609468.0838	3609468.0838	3609468.084	60049744.651
29	GG_25_20230604004816	4	n.a.	n.a.	n.a.	n.a.	n.a.
30	G_50_20230604014016	5	50.0000	6869809.4147	6869809.4147	6869809.415	108988931.025
31	GG_50_20230604020616	6	n.a.	n.a.	n.a.	n.a.	n.a.
32	G_100_20230604025816	7	100.0000	13649601.4530	13649601.4530	13649601.453	196445789.055
33	GG_100_20230604032419	8	n.a.	n.a.	n.a.	n.a.	n.a.
34	G_200_20230604041624	9	200.0000	23965615.8745	23965615.8745	23965615.874	314934089.504
35	G_400_20230604053442	10	400.0000	45546623.0062	45546623.0062	45546623.006	325386583.819
36	G_12	1	12.5000	1707597.0642	1707597.0642	1707597.064	28465515.208
37	GG_12	2	n.a.	n.a.	n.a.	n.a.	n.a.
38	G_25	3	25.0000	2611172.5877	2611172.5877	2611172.588	47785014.301
39	GG_25	4	n.a.	n.a.	n.a.	n.a.	n.a.
40	G_50	5	50.0000	6696047.7232	6696047.7232	6696047.723	105748371.377
41	GG_50	6	n.a.	n.a.	n.a.	n.a.	n.a.
42	G_100	7	100.0000	13453655.3226	13453655.3226	13453655.323	165961338.227
43	GG_100	8	n.a.	n.a.	n.a.	n.a.	n.a.
44	G_200	9	200.0000	25486425.9110	25486425.9110	25486425.911	301718905.539
45	G_400	10	400.0000	46511467.6787	46511467.6787	46511467.679	327162328.940

Calibration

Calibration Details	GlyGly		
Calibration Type	Poly2(vx)	*Offset (C0)*	0.0000
Evaluation Type	Area	*Slope (C1)*	139176.2585
Number of Calibration Points	8	*Curve (C2)*	427965.4347
Number of disabled Calibration Points	0	*R-Square*	0.9921

Calibration Plot **GlyGly**

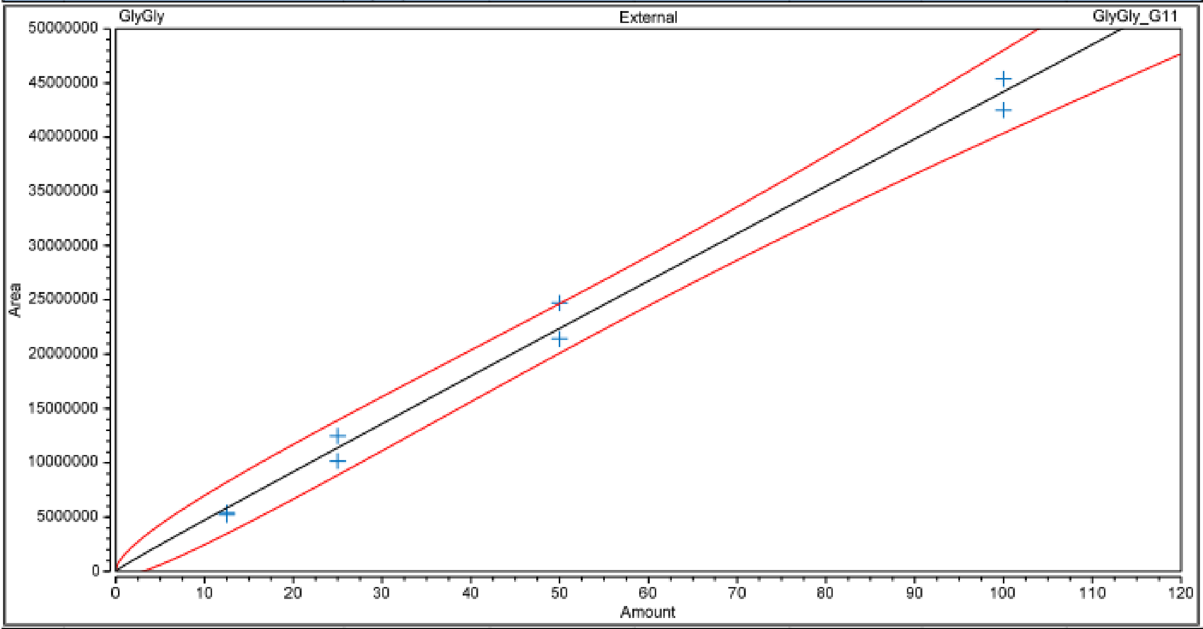

Calibration Results **GlyGly**

No.	Injection Name	Calibration Level	X Value<br><br>GlyGly_G11<br>GlyGly	Y Value<br><br>GlyGly_G11<br>GlyGly	Y Value<br><br>GlyGly_G11<br>GlyGly	Area<br>counts*min<br>GlyGly_G11<br>GlyGly	Height<br>counts<br>GlyGly_G11<br>GlyGly
26	G_12_20230603230415	1	n.a.	n.a.	n.a.	n.a.	n.a.
27	GG_12_20230603233016	2	12.5000	5384754.0942	5384754.0942	5384754.094	97637773.424
28	G_25_20230604002216	3	n.a.	n.a.	n.a.	n.a.	n.a.
29	GG_25_20230604004816	4	25.0000	12477607.2745	12477607.2745	12477607.275	196759190.800
30	G_50_20230604014016	5	n.a.	n.a.	n.a.	n.a.	n.a.
31	GG_50_20230604020616	6	50.0000	21412660.7238	21412660.7238	21412660.724	356168404.774
32	G_100_20230604025816	7	n.a.	n.a.	n.a.	n.a.	n.a.
33	GG_100_20230604032419	8	100.0000	42500409.6149	42500409.6149	42500409.615	677670184.695
34	G_200_20230604041624	9	n.a.	n.a.	n.a.	n.a.	n.a.
35	G_400_20230604053442	10	n.a.	n.a.	n.a.	n.a.	n.a.
36	G_12	1	n.a.	n.a.	n.a.	n.a.	n.a.
37	GG_12	2	12.5000	5219763.2798	5219763.2798	5219763.280	90770186.825
38	G_25	3	n.a.	n.a.	n.a.	n.a.	n.a.
39	GG_25	4	25.0000	10165642.4339	10165642.4339	10165642.434	199402806.172
40	G_50	5	n.a.	n.a.	n.a.	n.a.	n.a.
41	GG_50	6	50.0000	24719767.7495	24719767.7495	24719767.749	438024122.537
42	G_100	7	n.a.	n.a.	n.a.	n.a.	n.a.
43	GG_100	8	100.0000	45363923.8196	45363923.8196	45363923.820	646217894.684
44	G_200	9	n.a.	n.a.	n.a.	n.a.	n.a.
45	G_400	10	n.a.	n.a.	n.a.	n.a.	n.a.
