# Supplementary figures and images for: Heat flows enrich prebiotic building blocks and enhance their reactivity
Source: Nature. 2024 Apr 3;628(8006):110–6. doi: 10.1038/s41586-024-07193-7 (PMC10990939; doi:10.1038/s41586-024-07193-7)

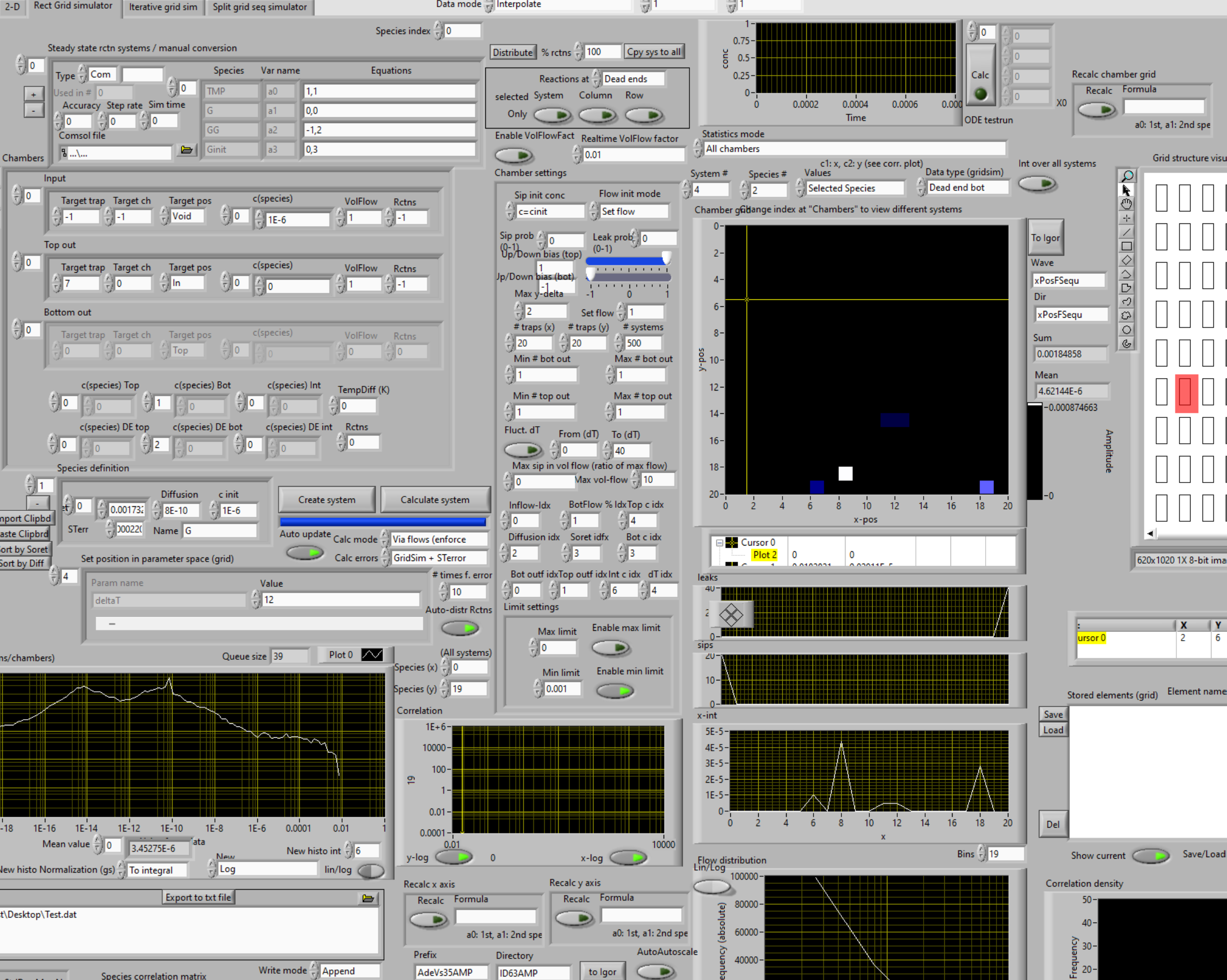

Supplement: Supplementary file 3 — This file Contains LabVIEW programs (llb), data files (dat) and COMSOL models (mph) used for numeric modelling, fitting of Soret coefficients and the modelling of the network of heat flow chambers as described in Methods. [file 41586_2024_7193_MOESM3_ESM.zip › AdditionalSubmissionFiles/NetworkSimulation/GridSimSettings.png]
